# Sequence-definition in stiff conjugated oligomers

**DOI:** 10.1038/s41598-018-35933-z

**Published:** 2018-11-30

**Authors:** Rebekka V. Schneider, Kevin A. Waibel, Andreas P. Arndt, Mathias Lang, Rebecca Seim, Dmitry Busko, Stefan Bräse, Uli Lemmer, Michael A. R. Meier

**Affiliations:** 1 Laboratory of Applied Chemistry, Institute of Organic Chemistry (IOC), Karlsruhe Institute of Technology (KIT), Straße am Forum 7, 76131 Karlsruhe, Germany; 2 Light Technology Institute (LTI), Karlsruhe Institute of Technology (KIT), Engesserstraße 13, 76131 Karlsruhe, Germany; 3 Institute of Organic Chemistry (IOC), Karlsruhe Institute of Technology (KIT), Fritz-Haber-Weg 6, 76131 Karlsruhe, Germany; 4 Institute of Microstructure Technology (IMT), Karlsruhe Institute of Technology (KIT), Hermann-von-Helmholtz-Platz 1, 76344 Eggenstein-Leopoldshafen, Germany

## Abstract

The concept of sequence-definition in the sense of polymer chemistry is introduced to conjugated, rod-like oligo(phenylene ethynylene)s via an iterative synthesis procedure. Specifically, monodisperse sequence-defined trimers and pentamers were prepared via iterative Sonogashira cross-coupling and deprotection. The reaction procedure was extended to tetra- and pentamers for the first time yielding a monodisperse pentamer with 18% and a sequence-defined pentamer with 3.2% overall yield. Furthermore, three novel trimers with a 9*H-*fluorene building block at predefined positions within the phenylene ethynylene chain were synthesised in 23–52% overall yields. Hence, it was confirmed that a functionality of interest can be incorporated selectively at a pre-defined position of these monodisperse oligomers. All respective intermediate structures were fully characterised by proton and carbon NMR, mass spectrometry, size-exclusion chromatography, and IR spectroscopy. Additionally, thermal and optical transitions are reported for the different oligomers.

## Introduction

The synthesis of monodisperse macromolecules with a defined monomer sequence came into the focus of polymer science lately^1–3^. In nature, sequence-defined macromolecules or oligomers, such as peptides and proteins, are produced in a highly precise manner^4^. The latter are based on a building set of more than 20 amino acids, enabling diverse secondary and tertiary structures^5^. Theoretically, an infinite number of monomer units can be utilised in polymer chemistry, but the extent of perfection observed in nature is not yet achieved. Several synthetic methods to sequence-defined macromolecules are described, including solution phase^6–9^, solid phase^10–13^, as well as fluorous-^14,15^ and polymer-tethered^16,17^ approaches. Procedures based on solution phase chemistry are often associated with complex purification procedures, but usually offer high yields and the possibility to characterise and optimise every step. For solid phase procedures, the purification process is drastically facilitated, but the yields as well as scales are rather restricted. Fluorous-tethered approaches use a fluorous tag for a simplified purification process with perfluorinated columns and the polymer-tethered options are purified by simple precipitation^2^.

In the field of conjugated polymers, monodisperse oligomers are investigated as model systems for potential applications^18^. For rod-like molecules, such as oligo(1,4-phenylene ethynylene)s, various procedures to monodisperse oligomers were pursued. In a recent approach, Bunz *et al*. synthesised water-soluble oligo(1,4-phenylene ethynylene)s (monomer to tetramer) by non-iterative procedures^19^. Many iterative procedures, which in principle allow to obtain sequence-defined macromolecules, were published by the group of Tour, for example a divergent approach to a 16mer with dodecyl side groups or a bidirectional approach to a molecule exhibiting seven benzene units^20–22^. Non-iterative as well as divergent or bidirectional procedures, however, do not provide full molecular control over each repeating unit. Only via iterative synthesis procedures, the structure and more specifically the sequence of building blocks can be controlled. Therefore, gradual changes in the electronic level of the conjugated oligomer backbone can be achieved^23^. So far, only few studies focus on the incorporation of diverse monomers^17,23–26^. For instance, Lutz *et al*. prepared oligo(1,4-arylene ethynylene)s iteratively with the aid of a soluble polystyrene support by Sonogashira cross-coupling and subsequent deprotection^17^. Unfunctionalised benzene and pyridine building blocks enabled the positioning within the sequence. UV/Vis and fluorescence spectra of the polymers exhibiting one to four aromatic units were measured and revealed a bathochromic shift with increasing length and pyridine content. The Tour group established another linear synthesis procedure to oligo(1,4-phenylene ethynylene)s up to the trimer stage^25^.

The Sonogashira reaction is also the method of choice for preparing rod-like polymers in form of poly(arylene ethynylene)s by converting aryl diiodides or dibromides with aromatic diynes^27–29^. Poly(arylene ethynylene)s are mainly investigated in applications for molecular wires and sensors, but also as transistors^27,30–32^. Usually, these polymers exhibit structural defects due to butadiyne formation related to Glaser coupling^33,34^. Monodisperse oligo(1,4-phenylene ethynylene)s are free from structural defects, since chromatographic procedures are applied for purification.

Our approach towards sequence-defined oligo(1,4-phenylene ethynylene)s is based on the Sonogashira cross-coupling as well^29^. Although short monodisperse rod-like oligomers obtained by Sonogashira cross-coupling were published before (see above), an extension to sequence-defined pentamers was not achieved yet. Furthermore, novel molecules with a targeted incorporation of a fluorene unit were synthesised, demonstrating the possibility to systematically tune the structure of such highly defined macromolecules. All oligomers are molecularly fully characterised and demonstrate the success as well as limitations of this approach (see electronic supplementary information with all details). Furthermore, optical and thermal properties are reported to demonstrate the influence of the sequence on the properties of the obtained macromolecules.

## Results and Discussion

### Synthesis strategy

**Figure 1:**
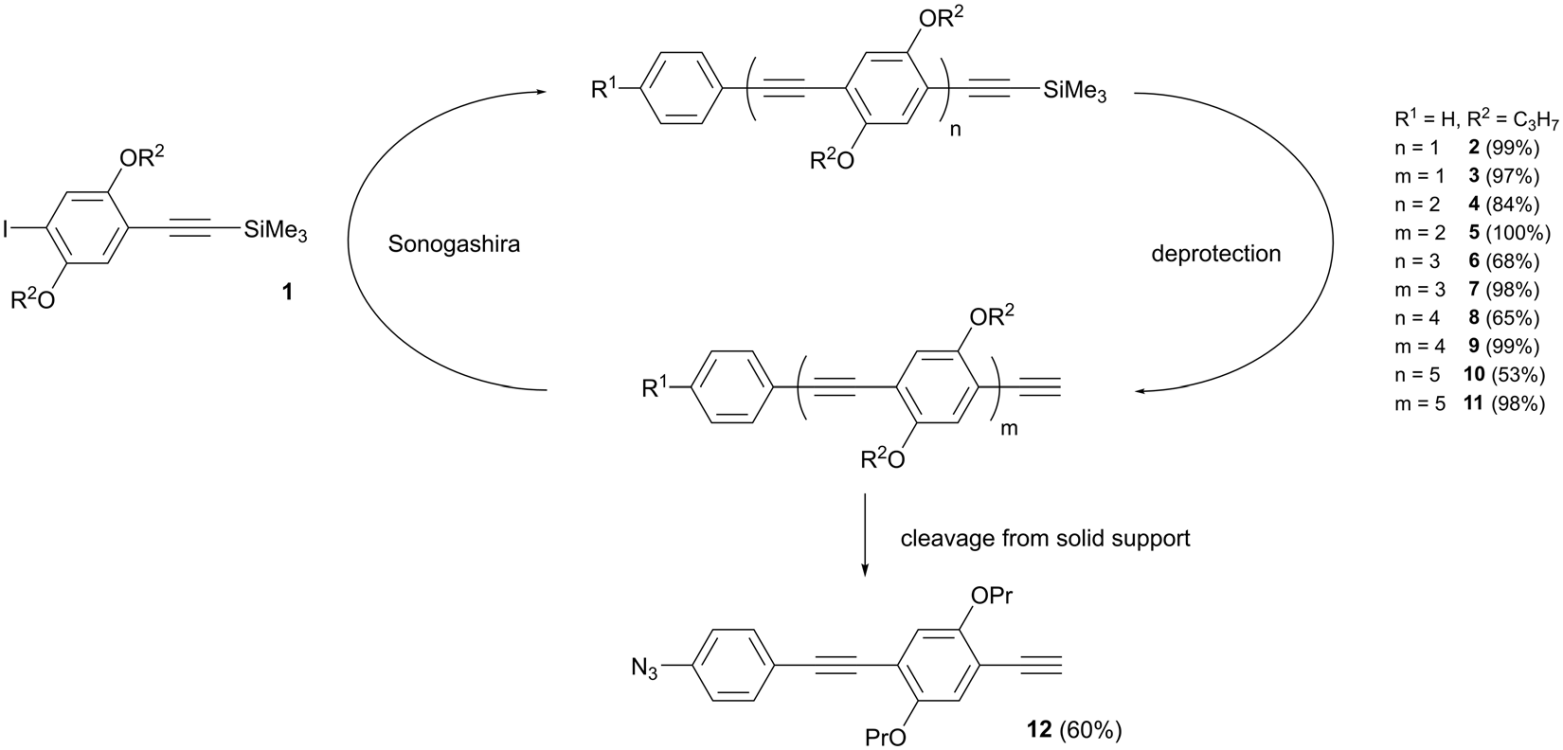
Synthesis procedure towards monodisperse oligo(1,4-phenylene ethynylene)s through Sonogashira cross-coupling with the building block **1** and subsequent deprotection. The approach in solution yields the protected monomer **2**, the deprotected monomer **3**, the respective versions of the dimers **4** and **5**, the trimers **6** and **7**, the tetramers **8** and **9** as well as the pentamers **10** and **11**. Cleavage from the resin in SPOS results in the azide functionalised monomer **12**.

For the synthesis of sequence-defined rod-like oligomers, five building blocks were synthesised starting from hydroquinone. By applying Williamson ether synthesis with 1-bromopropane, 2-bromopropane, bromocyclohexane and 1-bromooctane, four 1,4-dialkoxybenzenes were obtained^35^. An iodination of 1,4-dimethoxybenzene and the obtained 1,4-dialkoxybenzenes with periodic acid yielded the 1,4-diiodo-2,5-dialkoxybenzenes^36^. These were converted with trimethylsilylacetylene into protected bifunctional building blocks via Sonogashira mono-coupling^37^. First, building block **1** with propoxy side groups was used for establishing the synthesis procedure in a solution and a solid phase organic synthesis (SPOS) approach (Fig. 1). For SPOS, a triazene linker was applied^38^.

The synthesis in solution started with a Sonogashira reaction of the building block 1 with phenylacetylene and proceeded smoothly to yield the respective monomer **2** in 99% yield. The deprotection was performed with potassium carbonate and the deprotected monomer **3** was received with 97%. After ten iterative steps of Sonogashira reaction with **1** and subsequent deprotection, the deprotected monodisperse pentamer **11** was obtained in an overall yield of 18% and in a scale of 116 mg. All intermediate steps are fully characterised in the Supplementary Information (^1^H and ^13^C NMR, SEC, and MS), unambiguously confirming the success of the synthesis. The purification was challenging, since Glaser coupling due to traces of oxygen could not be suppressed completely. Thus, this side reaction constitutes the major limiting factor for better yields in the Sonogashira cross-coupling.

The SPOS approach was initially performed in analogy to the solution approach, however product formation was not observed. The acquired conditions for the solution approach are therefore not directly transferable to SPOS and the above-mentioned Glaser coupling side reaction is even more problematic in this case, as it leads to further cross-linking of the resin. Further investigations enabled the cleavage of azide functionalised monomer **12** (Fig. 1) in an overall yield of 7 mg (60%). However, the initial concept of a fast and direct synthesis strategy via SPOS could not sustain, and the yield and scale are significantly lower as for the solution approach to deprotected monomer **3** with 3.15 g (96%). Therefore, SPOS was not further investigated here.

### Polycondensation with chain stopper

**Figure 2:**
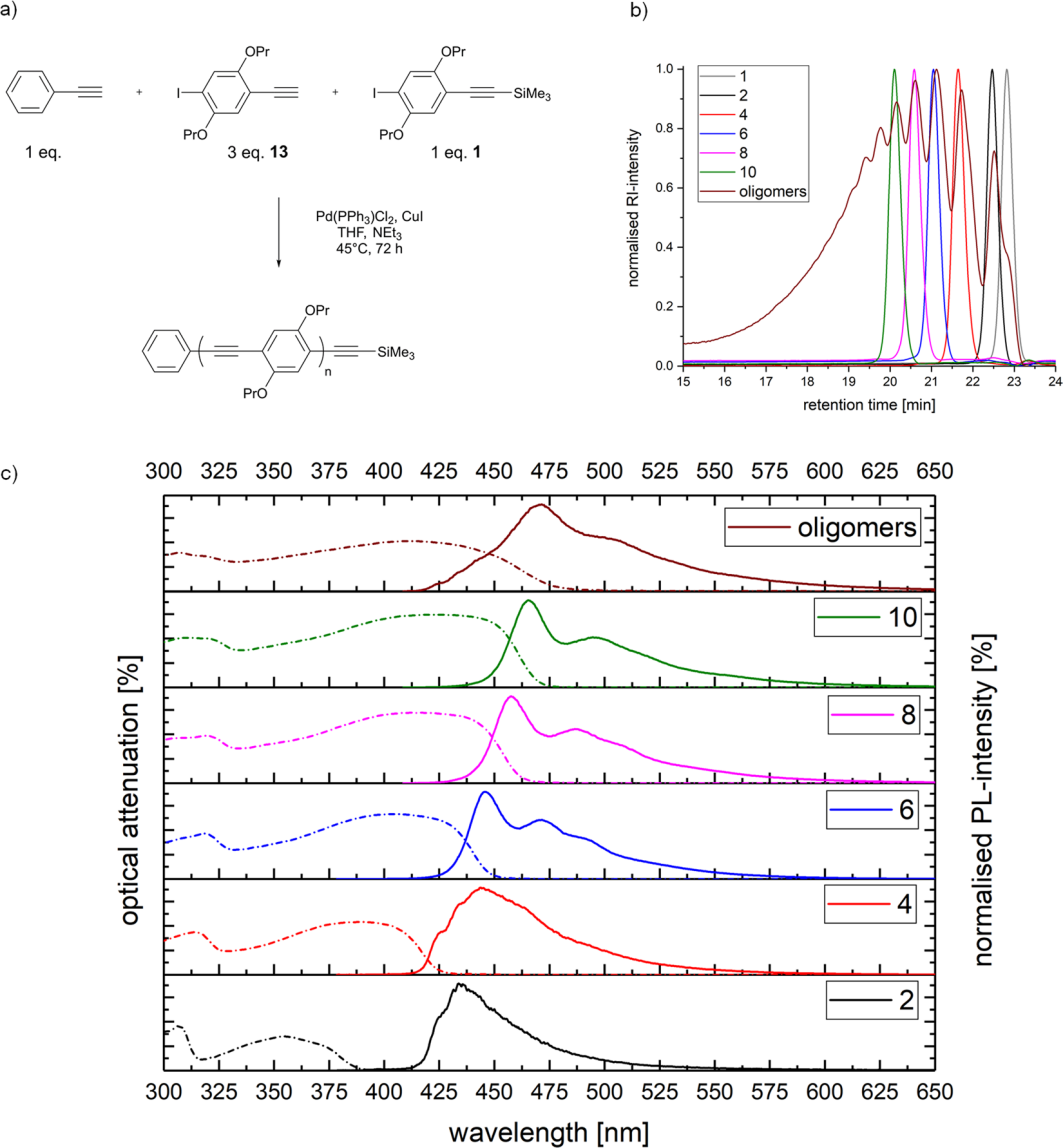
The polycondensation approach and its comparison with monodisperse oligomers. (**a**) Schematic representation of the polycondensation approach with starting unit (phenylacetylene), monomer **13** and chain stopper **1**. (**b)** SEC traces of a step-growth approach with monofunctional monomers (brown) in comparison to the monodisperse protected oligomers - monomer **2** (black), dimer **4** (red), trimer **6** (blue), tetramer **8** (pink) and pentamer **10** (green) and the building block **1** (grey). The trimer **4** is most abundant in the mixture. (**c**) Absorption (dotted) and photoluminescence (dotted) spectra of the polycondensation approach (brown), in comparison to the oligomers.

A further direct oligomerisation approach in solution was performed as depicted in Fig. 2. A deprotected version of **1** exhibiting an unprotected tripled bond served as monomer (**13**, Fig. 2a), phenylacetylene and building block **1** act as starting unit and chain stopper, respectively. By applying the ratio 1:3:1 (starting unit, monofunctional monomer **13**, chain stopper 1), short chain oligomers like the tetramer **8** were expected to form preferentially within the oligomer mixture. The comparative size exclusion chromatography (SEC) traces (Fig. 2b) confirm the formation of short chain oligomers, but also clearly demonstrate the high dispersity within the product mixture. Thus, the SEC trace clearly outlines the necessity of the herein described iterative approach towards defined oligomers (Fig. 1).

Furthermore, absorption and emission properties of the oligomer approach in comparison to the monodisperse representatives were investigated (Fig. 2c). The absorption maximum for the trimer **6** is observed at 402 nm, for the tetramer **8** at 410 nm and for the oligomer mixture at 406 nm. Therefore, the most abundant oligomer in the mixture should be the trimer **6** or the tetramer **8**, which is in accordance with the SEC trace (Fig. 2b). Generally, the maximum of the photoluminescence intensity increases from the monomer **2** (400 nm) to the pentamer **10** (465 nm) and the oligomer mixture exhibits its maximum at 471 nm. Thus, in order to achieve defined photophysical properties, monodisperse oligomers offer unique advantages.

### A sequence-defined rod-like pentamer

**Figure 3:**
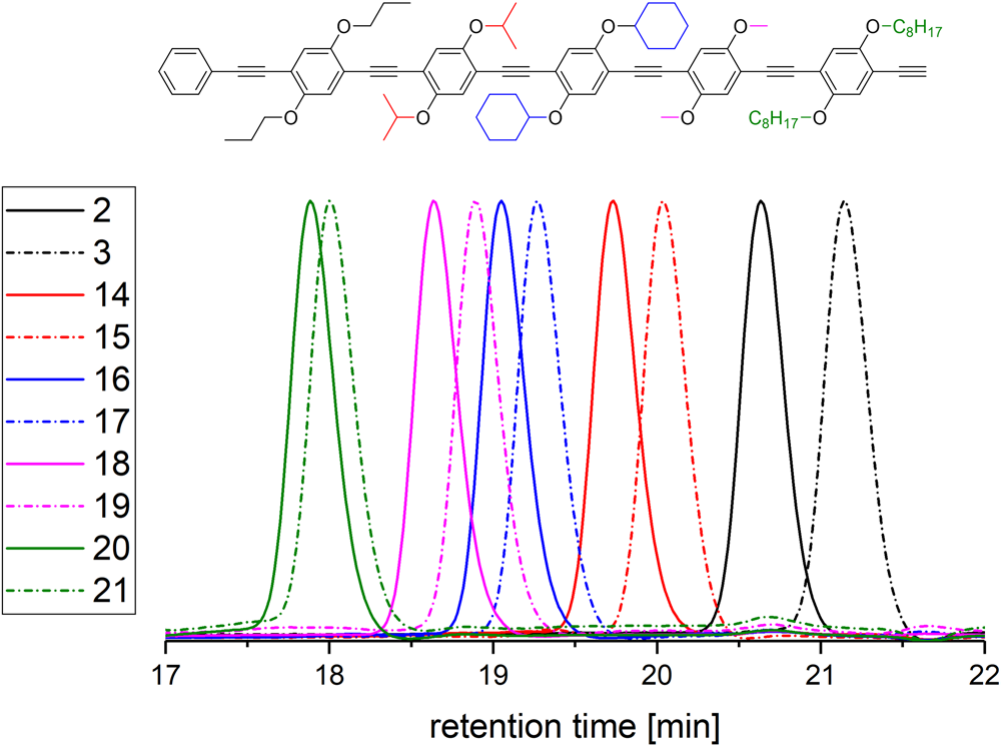
SEC traces of the sequence-defined oligomers. The protected versions are displayed as bold, the deprotected representatives as dotted lines: protected monomer **2** (black, bold), deprotected monomer **3** (black, dotted), the respective versions of the dimers **14** and **15** (red), trimers **16** and **17** (blue), tetramers **18** and **19** (pink) as well as pentamers **20** and **21** (green). The colour code of the SEC traces corresponds to the coloured side chains of the structure displayed above.

With the previously synthesised building blocks in hand and the experience gained in the synthesis of pentamer **10**, subsequently sequence-defined oligomers were synthesised starting from deprotected monomer **3**. The protected, sequence-defined dimer **14** exhibits propoxy side groups at position **1** and isopropoxy side groups at position **2**. Deprotection yields deprotected, sequence-defined dimer **15** (Fig. 3).

Eventually, sequence-defined deprotected pentamer **21** (compare structure depicted in Fig. 3) was synthesised iteratively according to Fig. 1 in a lower overall yield of 3.2% and in a scale of 73.6 mg. The sterically demanding moieties (i.e. cyclohexyl) restricted better yields in the Sonogashira reaction. Also, the side reaction is favoured as the respective Glaser coupling products are sterically less hindered compared to the desired respective oligomers. In Fig. 3, the SEC traces of the sequence-defined oligomers and their respective protected precursors are depicted confirming the high purities of the compounds and intermediates. All other characterisation data can be found in the supporting information.

The absorption and photoluminescence spectra of the sequence-defined oligomers in comparison to the monodisperse compounds differ only slightly, since the respective electronic properties do not change significantly. The thermal properties differ however, since the monodisperse and the sequence-defined oligomers exhibit distinct molecular weight and side groups (Table 1). The sequence-defined representatives have higher melting points (apart from the dimers) and higher glass transition temperatures compared to the monodisperse oligomers. For instance, the monodisperse trimer **6** shows a *T*_g_ of 34.4 °C and a *T*_m_ of 125.5 °C, whereas the sequence-defined trimer **16** shows a *T*_g_ of 52.7 °C and a *T*_m_ of 189.2 °C. Higher melting points result from the higher molecular weight of the molecules and the higher glass transition temperatures from the sterically more demanding side groups (especially cyclohexyl), which leads to high rotation barriers^39^.

**Table 1:** Overview of melting points (*T*_m_) and glass transition temperatures (*T*_g_). The monodisperse trimer **6**, tetramer **8** and pentamer **10** exhibit lower melting points and glass transition temperatures compared to the sequence-defined representatives

Monodisperse oligomers	*T*_g_	*T*_m_	Sequence-defined oligomers	*T*_g_	*T*_m_
dimer **4**	19.3 °C	129.2 °C	dimer **14**	22.9 °C	123.2 °C
trimer **6**	34.4 °C	125.5 °C	trimer **16**	52.7 °C	189.2 °C
tetramer **8**	45.6 °C	160.5 °C	tetramer **18**	67.7 °C	176.6 °C
pentamer **10**	—	186.8 °C	pentamer **20**	—	—

### Novel trimers with pre-defined incorporation of a fluorene moiety

**Figure 4:**
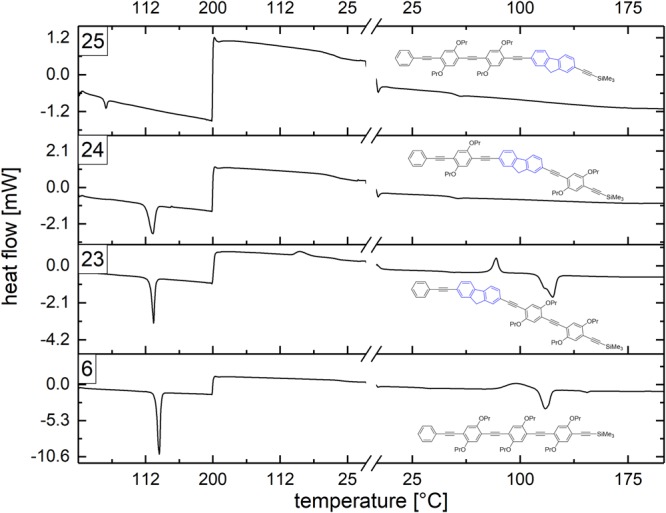
DSC traces of the sequence-defined trimers **23**, **24** and **25** and the monodisperse trimer **6** with the respective molecular structures. The molecules were heated up to 200 °C and then cooled to 0 °C (left). The temperature was retained at 0 °C for 10 minutes (not depicted) and then a further heating cycle to 200 °C was performed (right).

The confirmation of the synthesis strategy inspired us to synthesise a 9*H*-fluorene building block **22** derived from 2,7-diiodofluorene. This novel building block, exhibiting again an iodine moiety as well as a trimethylsilyl-protected triple bond, was applied for the sequence-specific incorporation into oligo(1,4-phenylene ethynylene)s. In this way, three trimers (see Fig. 4 for respective structures) with systematically altered position of the fluorene unit were obtained (**23**, **24**, **25**). The overall yields and scales amounted to 21% and 188 mg (**23**), 36% and 319 mg (**24**), as well as 52% and 426 mg (**25**). All characterisation data of these trimers is provided in the supporting information, confirming their structure and high purity. In order to investigate the influence of the fluorene position, differential scanning calorimetry (DSC) was performed for the three trimers and the monodisperse (1,4-phenylene ethynylene) trimer **6** as depicted in Fig. 4.

Interestingly, the melting points of the trimers incorporating the fluorene units at position 1 (**23**) with 118.3 °C and at position 2 (**24**) with 112.6 °C resemble the melting point of monodisperse trimer **6** with 125.5 °C. Trimer **25** does not exhibit a melting point, but a glass transition (*T*_g_) at 57.3 °C. Performing the DSC measurement of trimer **24** again, no melting point, but a *T*_g_ at 54.2 °C is detected as well. In further measurements, exclusively the *T*_g_ was detectable. Annealing attempts at 75 °C for 1 hour and altered heating and cooling rates did not lead to crystallisation of **24**; the same applies to trimer **25**. The monodisperse trimer **6** with its rod-like structure can obviously arrange comparably easy into a crystal structure, also **23** is able to recrystallise. Both representatives exhibit a solid-solid transition in the second heating cycle^40^. Trimer **24** and **25** do not recrystallise easily proving that the position of the fluorene unit has a relevant impact on the thermal properties.

**Figure 5:**
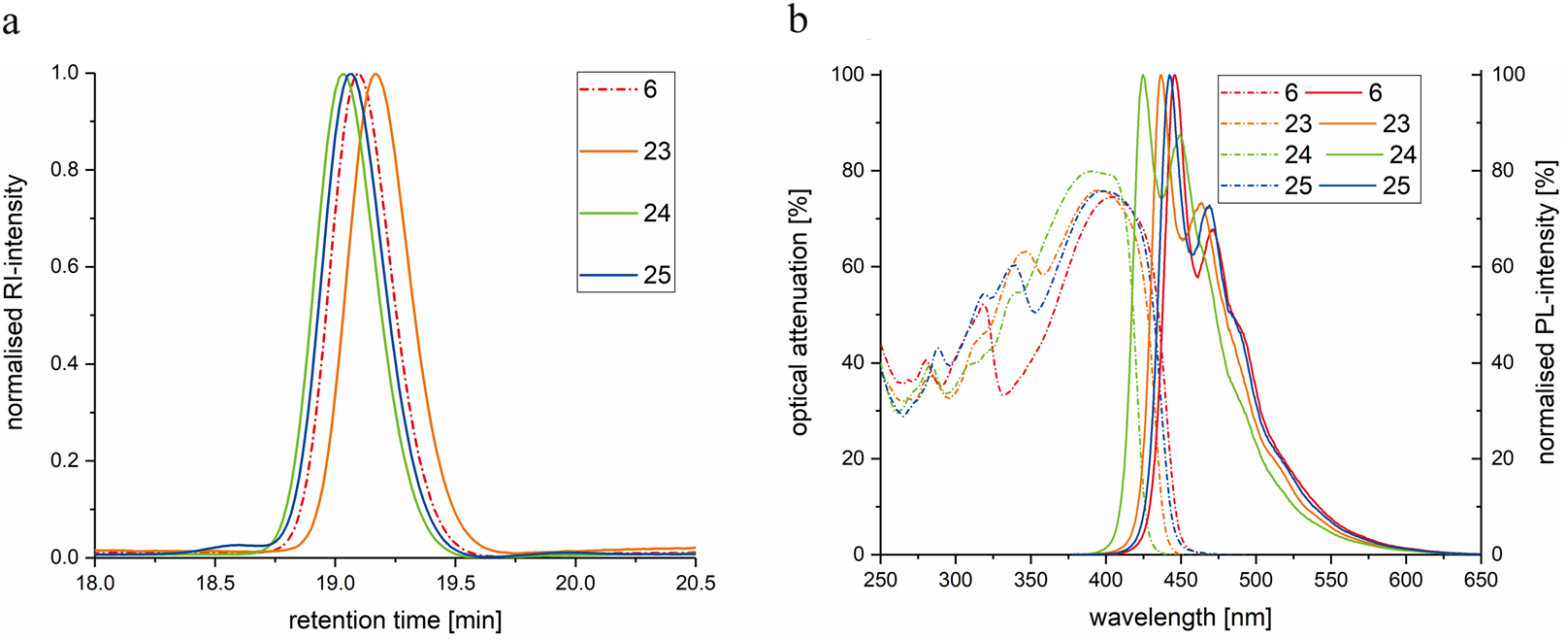
Properties of novel sequence-defined trimers with one fluorene unit and monodisperse trimer **6**. (**a**) SEC traces of trimer **23** with the fluorene unit at position 1 (orange), **24** with the fluorene unit at position 2 (green) and **25** with the fluorene unit at position 3 (blue) in comparison to monodisperse trimer **6** (red, dotted). (**b**) Absorption spectra (dotted) of monodisperse and sequence-defined trimers **6** (red), **23** (orange) **24** (green) **25** (blue) and photoluminescence spectra of **6**, **23**–**25** (bold).

According to SEC, **24** with the fluorene unit in the middle exhibits the largest hydrodynamic volume (Fig. 5a), probably since the different bond angle in the middle of the molecule has the largest influence within the series of stiff trimers. However, one would assume that **23** and **25** elute similar, but **23** exhibits a relatively smaller hydrodynamic radius. Thus, the interchanged phenyl and trimethylsilyl groups lead to a significantly altered hydrodynamic volume.

Furthermore, the maximum absorption and photoluminescence intensities differed (Fig. 5b): **24** displayed the lowest wavelengths. Moreover, the Stokes shift is significantly smaller for **24** compared to the other trimers. The exact wavelengths of the trimers are depicted in Table 2. Trimers **23** and **25** exhibit similar wavelengths of maximum absorption intensities, maximum photoluminescence intensities and similar Stokes shifts. Monodisperse trimer **6** exhibits the highest wavelength of maximum absorption and photoluminescence intensities. The Stokes shift is similar to **23** and **25**. The latter results indicate that the conjugated system especially in trimer **24** is slightly disturbed if compared to the non-fluorene containing trimer **6**. This effect seems to be less pronounced for fluorene units placed at positions 1 and 3 of trimers **23** and **25**, respectively.

**Table 2:** Overview of maximum absorption and photoluminescence intensities and the Stokes shift. The monodisperse trimer **6** exhibits the highest wavelengths of maximum absorption and photoluminescence intensities. The Stokes shift is similar to fluorene-containing trimers **23** and **25**. Trimer **24** with the fluorene unit at position 2 has lower maximum absorption and photoluminescence intensity wavelengths; also the Stokes-shift is significantly smaller

Oligomer	λ_max_ absorption	λ_max_ photoluminescence	Stokes-shift
**6**	404 nm	446 nm	42 nm
**23**	395 nm	437 nm	42 nm
**24**	392 nm	425 nm	33 nm
**25**	398 nm	442 nm	44 nm

Additionally, time-resolved emission spectra were recorded, and the transient characteristic of the photoluminescence was fitted by a biexponential decay function. All transients are strongly dominated by the shorter-lived decay component with a lifetime of 1.15 ns for the monodisperse trimer **6** and only 0.97 ns for trimer **24**. The trimers **23** and **25** show similar decay times of 1.03 ns and 1.01 ns, respectively. Photoluminescence quantum yield (PLQY) experiments at an excitation wavelength of 405 nm showed that trimer **25** exhibits the lowest PLQY of 83.3%. Trimers **6**, **23** and **24** exhibit a similar PLQY of 88.5%, 88.7% and 89.3%, respectively. Longer lifetimes usually relate to lower quantum yields^41^, which cannot explain the significantly lower PLQY value for trimer **25** in this case.

## Conclusion

To conclude, a monodisperse as well as a sequence-defined pentamer were synthesised in solution. So far, only trimers have been synthesised in an iterative fashion, thus significantly extending existing concepts. In parallel to the solution approach, SPOS and a direct oligomerisation were tested as fast alternatives but could not compete. The concept of sequence-definition was transferred to the synthesis of three novel sequence-defined trimers incorporating one fluorene moiety at a pre-defined position. These molecules were not only analysed by ^1^H and ^13^C NMR, MS, IR, and SEC, but also with DSC and several photophysical methods. The photophysical properties of the monodisperse and sequence-defined trimers differed only slightly, but the sequence had an impact on the thermal properties and the hydrodynamic volume. However, no clear trend of the properties according to the sequence could be observed. In the future, iterative synthesis procedures could be applied for the investigation and understanding of structure-property relationships by synthesising further structures with more distinct sequences.

## Methods

### General Synthetic Procedures

General procedure for the Williamson ether synthesis to 1,4-dialkoxybenzenes according to H. Meier *et al*.^35^.

Hydroquinone (1.00 eq., typically 275 mmol) was dissolved in 250 mL absolute ethanol. Potassium hydroxide (2.50 eq.) was added and the mixture was stirred for 30 minutes under reflux. Subsequently, the bromoalkane (2.20 eq.) was slowly added over a 1 h time period and stirred under reflux for another 2 h. Ethanol was removed with a rotary evaporator and the residue was taken up in dichloromethane. The organic phase was washed with water three times and once more with saturated NaHCO_3_ solution. It was then dried over Na_2_SO_4_, filtered and the solvent was removed under reduced pressure. The crude product was either recrystallised from methanol (if solid) or column chromatography was performed (cyclohexane/ethyl acetate 20:1). Typical yields: 28–76%.

General procedure for the iodination to 1,4-diiodo-2,5-dialkoxybenzenes after Park *et al*.^36^.

Periodic acid (0.636 eq., typically 14.0 mmol) was dissolved in 25 mL methanol and stirred for 10 minutes. Subsequently, iodine (1.23 eq.) was added and after an additional stirring time of 10 minutes, 1,4-dialkoxybenzene (1.00 eq.) was added. The reaction mixture was stirred at 70 °C for 4 h. The residue was carefully poured into 50 mL water containing potassium disulfite. The precipitate was washed with methanol and dissolved in dichloromethane. The solution was filtered, and the filtrate was concentrated under reduced pressure. The residue was purified by recrystallisation from methanol or ethanol. Typical yields: 64–92%).

General procedure for the Sonogashira mono-coupling to the dialkoxy-substituted building blocks (*e.g*. 1) after Tour *et al*.^37^.

All Sonogashira reactions were performed under argon atmosphere.

1,4-Diiodo-2,5-dialkoxybenzene (1.00 eq., typically 22.4 mmol), 2.5 mol% *bis*(triphenylphosphine)palladium(II) dichloride and 5 mol% copper(I) iodide were placed into a Schlenk flask and degassed. Under continuous argon flow, 400 mL dry THF and 30 mL dry triethylamine were added, and the mixture was stirred for 10 minutes. Subsequently, trimethylsilylacetylene (1.10 eq.) with 5 mL dry THF was added dropwise with a syringe. The reaction mixture was stirred for 20 h at room temperature, taken up in dichloromethane and washed with saturated NH_4_Cl solution. The aqueous phase was extracted three times with dichloromethane. The combined organic layers were dried over Na_2_SO_4_, filtered and concentrated under reduced pressure. The residue was purified by silica column chromatography (cyclohexane/dichloromethane 9:1 or 5:1). Typical yields: 33–46%.

Procedure for the Sonogashira mono-coupling to the 9*H*-fluorene building block **22**.

2,7-Diiodo-9*H*-fluorene (1.00 eq., typically 12.0 mmol), 2.5 mol% bis(triphenylphosphine)palladium(II) dichloride and 5 mol% copper(I)iodide were placed into a Schlenk flask and degassed. Under continuous argon flow, 200 mL dry THF and 17 mL dry triethylamine were added, and the mixture was stirred for 10 minutes. Subsequently, trimethylsilylacetylene (0.603 eq) was added dropwise with a syringe. The reaction mixture was stirred for 20 h at room temperature, taken up in dichloromethane and washed with saturated NH_4_Cl solution. The aqueous phase was extracted three times with dichloromethane. The combined organic layers were dried over Na_2_SO_4_, filtered and concentrated under reduced pressure. The residue was purified by silica column chromatography (cyclohexane/dichloromethane 30:1) to yield the product as a white solid (43%).

General procedure for the Sonogashira cross-coupling to monomers (**2** and fluorene-containing monomer).

**1** or **22** (1.00 eq., typically 12.0 mmol), 2.5 mol% bis(triphenylphosphine) palladium(II) dichloride and 5 mol% copper(I) iodide were placed into a Schlenk flask and degassed three times. Under continuous argon flow, 150 mL dry THF and dry triethylamine (10.0 eq.) were added and the mixture was stirred for 10 minutes. Subsequently, phenylacetylene (3.00 eq.) in 5 mL THF was added dropwise with a syringe. The reaction mixture was stirred for 48 h at room temperature, taken up in dichloromethane and washed with saturated NH_4_Cl solution. The aqueous phase was extracted three times with dichloromethane. The combined organic layers were dried over Na_2_SO_4_, filtered and concentrated under reduced pressure. The residue was purified by silica column chromatography twice (cyclohexane/dichloromethane 4:1 and cyclohexane/ethyl acetate 20:1). Typical yields: 92–99%.

General procedure for the deprotection of the trimethylsilyl group (**3, 5, 7, 9, 11, 13, 15, 17, 19, 21**).

The protected oligomer (1.00 eq., typically 10 mmol) and two equivalents of potassium carbonate were placed into a Schlenk flask and degassed three times. Under continuous argon flow, 200 mL dry dichloromethane and 200 mL dry methanol were added. The reaction mixture was stirred overnight at room temperature under argon atmosphere and quenched with distilled water. The aqueous phase was extracted three times with dichloromethane, dried over Na_2_SO_4_, filtered and concentrated under reduced pressure. The residue was purified by flash silica column chromatography (either cyclohexane/ethyl acetate or dichloromethane/cyclohexane). Typical yields: 85–100%.

General procedure for the Sonogashira cross-coupling to dimers and higher molecular weight oligomers (**4, 6, 8, 10, 12, 14, 16, 18, 20, 23, 24, 25**).

The building block (*e.g*. 1 or 22) (3.00–5.00 eq.), 5 mol% bis(triphenylphosphine) palladium(II) dichloride and 5 mol% copper(I) iodide were placed into a Schlenk flask and degassed three times. Under continuous argon flow, 100 mL dry THF and dry triethylamine (10.0 eq.) were added and the mixture was stirred for 10 minutes. Subsequently, rod-like oligomer (*e.g*. **3, 5, 7, 9, 13, 15, 17, 19**) (1.00 eq.) in 20 mL dry THF was added dropwise with a syringe. The reaction mixture was stirred for 72 h (3 d) at 45 °C, taken up in dichloromethane and washed with saturated NH_4_Cl solution. The aqueous phase was extracted three times with dichloromethane. The combined organic layers were dried over Na_2_SO_4_, filtered and concentrated under reduced pressure. The residue was purified by silica column chromatography (cyclohexane/dichloromethane) and a flash silica column (cyclohexane/ethyl acetate) or by recrystallisation from cyclohexane/ethyl acetate. Typical yields: 33–84%.

## Electronic supplementary material

SUPPLEMENTARY INFO
