# Supplementary material for: Sequence-definition in stiff conjugated oligomers
Source: Sci Rep. 2018 Nov 30;8:17483. doi: 10.1038/s41598-018-35933-z (PMC6269511; doi:10.1038/s41598-018-35933-z)
Supplement: Supplementary file 1 — SUPPLEMENTARY INFO [file 41598_2018_35933_MOESM1_ESM.pdf]

Supplementary Material for:

Sequence definition in stiff conjugated oligomers

R. V. Schneider,[a] K. A. Waibel,[a] A. P. Arndt,[b] M. Lang,[c] R. Seim,[a] D. Busko,[d] S. Bräse,[c] U. Lemmer,[b] and M. A. R. Meier[a]*

[a] Institute of Organic Chemistry (IOC), Karlsruhe Institute of Technology (KIT), Straße am Forum 7, 76131 Karlsruhe (Germany); [b] Light Technology Institute (LTI), Karlsruhe Institute of Technology (KIT), Engesserstraße 13, 76131 Karlsruhe (Germany), [c] Institute of Organic Chemistry (IOC), Karlsruhe Institute of Technology (KIT), Fritz-Haber-Weg 6, 76131 Karlsruhe (Germany), [d] Institute of Microstructure Technology (IMT), Karlsruhe Institute of Technology (KIT), Hermann-von-Helmholtz-Platz 1, 76344 Eggenstein-Leopoldshafen (Germany)

Table of Contents

Table of Contents 2
1. Experimental Procedures 3
1.1 Materials 3
1.2 Characterisation 3
1.3 Synthesis of the Building Blocks 5
1.4 Synthesis of the Monodisperse Pentamer 50
1.5 Solid Phase Organic Synthesis (SPOS) Approaches 80
1.6 Oligomerization Approach 84
1.7 Synthesis of the Sequence-Defined Pentamer 87
1.8 Synthesis of Sequence-Defined Trimers with one Fluorene Unit 111
2. Overview of overall yields 138
3. SEC Traces 139
3.1 Monodisperse Oligomers 139
3.2 Sequence-Defined Trimers with one Fluorene Unit 140
4. Optical properties 141
4.1 Sequence-Defined Oligomers 141
4.2 Sequence-Defined Trimers with one Fluorene Unit 143
References 146

1. Experimental Procedures

1.1 Materials

The following chemicals were used as received: hydroquinone (≥99%, Bayer), 1-bromopropane (99%, Fluka), 2-bromopropane (99%, TCI), bromocyclohexane (98%, Sigma-Aldrich), 1-bromooctane (98%, TCI), potassium hydroxide (≥99.97%, Sigma Aldrich), 1,4-dimethoxybenzene (98%, Alfa Aesar), iodine (≥99.8%, VWR Chemicals), periodic acid (99%, Fisher Bioreagents), potassium metabisulfite (≥96%, Roth), 2,7-diiodo-9H-fluorene (97%, Sigma-Aldrich/TCI), bis(triphenylphosphine)palladium(II) dichloride (≥99%, Sigma-Aldrich), copper(I) iodide (≥99.5%, Sigma-Aldrich), trimethylsilylacetylene (98%, abcr), ammonium chloride (≥99%, BASF), phenylacetylene (98%, Sigma-Aldrich), potassium carbonate (≥99.5%, Evonik, Tetrakis(triphenylphosphine)palladium(0) (Carbolution, 98%), tetra-n-butylammonium fluoride (Sigma Aldrich, 1 M in THF), trimethylsilyl azide (abcr, 95%), cupral solution (Sodium diethyldithiocarbamate trihydrate, Sigma Aldrich, p.a.), trifluoroacetic acid (abcr, 99%), anhydrous dichloromethane (≥99.8%, Sigma-Aldrich), anhydrous methanol (≥99.8%, Sigma-Aldrich), anhydrous tetrahydrofuran (≥99.9%, Sigma-Aldrich), sodium sulfate (>99%, Sigma Aldrich), toluene (99.7%, Bernd Kraft), ethanol (HPLC-grade, VWR Chemicals), methanol (HPLC-grade, VWR Chemicals), isopropanol (HPLC-grade, VWR Chemicals), dichloromethane (HPLC-grade, VWR Chemicals), anhydrous dimethylformamide (Sigma Aldrich, 99.8%), chloroform-d (99.8 atom% D, Euriso-top), dimethylsulfoxide-d6 (99.8 atom% D, Euriso-top).

Cyclohexane and ethyl acetate in technical grade were distilled before use.

Triethylamine (≥99.5%, Roth) was dried over calcium hydride and subsequently distilled under argon.

Tetrahydrofuran in HPLC grade (≥99.7%, VWR Chemicals) was dried over sodium and subsequently distilled under argon. Benzophenone was used to indicate the abstinence of water and oxygen.

(*E*)-3-benzyl-3-M*-1-(4-iodophenyl)triaz-1-ene was kindly provided by Nicolai Wippert (KIT).

1.2 Characterisation

NMR spectra were recorded on a Bruker AVANCE DPX spectrometer operating at 300 MHz for ^{1}H and 75 MHz for ^{13}C and a Bruker AVANCE DRX operating at 400 MHz for ^{1}H and 100 MHz for ^{13}C. $CDCl_3$ was used as solvent and the resonance signal 7.26 ppm (^{1}H, $CDCl_3$) and 77.16 ppm (^{13}C, $CDCl_3$) served as reference for the chemical shift δ. All measurements were performed at room temperature. For the different multiplicity of the NMR-signals the following abbreviations were used: s = singlet, d = doublet, t = triplet, p = quintet, sex = sextet, h = septet, m = multiplet.

Size exclusion chromatography (**SEC**) was performed on a Varian 390-LC gel permeation chromatography (GPC) system equipped with a LC-290 pump (Varian), refractive index detector (24 °C), PL AS RT GPC-autosampler (Polymer laboratories) and a Varian Pro Star column oven Model 510, operating at 40 °C. For separation, two systems were used. System A consisted of two SDV 5 µm linear

S columns (8 x 300 mm) and a guard column (8 x 50 mm). System B consisted of two SDA 3 µm linear S columns (8 x 300 mm) and a guard column (8 x 50 mm).

Infrared spectra (**IR**) were recorded on a Bruker Alpha-p instrument in a frequency range from 3997.21 to 373.94 cm^{-1} applying ATR-technology.

Fast atom bombardment (**FAB**) mass spectra were recorded on a Finnigan MAT 95 instrument.

Orbitrap electrospray ionization mass spectra (**ESI-MS**) were recorded on a Q Excative (Orbitrap) mass spectrometer (Thermo Fisher Scientific, San Jose, CA, USA) equipped with an atmospheric pressure ionization source operating in the nebulizer assisted electrospray mode. The instrument was calibrated in the m/z-range 150-2000 using premixed calibration solutions. A constant spray voltage of 3.5 kV and a dimensionless sheath gas of 6. The capillary voltage and the S-lens RF level were set to 68.0 V and 320 °C, respectively.

Thin layer chromatography (**TLC**) was performed on silica gel coated aluminum foil (silica gel 60 F_{254}, Sigma-Aldrich). Compounds were visualized by UV at 256 and 365 nm.

Thermal properties were recorded via differential scanning calorimetry (**DSC**) with a Mettler Toledo DSC $star^{e}$ system operating under nitrogen atmosphere using approximately 3 mg of the respective sample. Following method was used: heating from 25 to 200 °C with a heating rate of 20 K/min, cooling from 200 to 0 °C with a cooling rate of 20 K/min, an isothermic segment at 0 °C for 10 min and a further heating cycle from 0 to 200 °C with a heating rate of 20 K/min.

Optical attenuation was recorded on a UV/Vis spectrophotometer (LAMBD A1050, PerkinElmer), which was additionally equipped with an integrating sphere.

Steady-state photoluminescence spectra were taken from a 100 µMol concentrated solution at an excitation wavelength of 355 nm and with an excitation power of 300 µW. The Photoluminescence was spectrally dissolved by a spectrometer (Acton SpectraPro SP-2300, Princeton Instruments) and detected by a CCD-camera (PI-MAX4, Princeton Instruments).

Time-resolved photoluminescence spectra were recorded from the same solutions with a streak camera (C10910-02, Hamamatsu) operated in photon counting mode. For excitation, we used the frequency-doubled laser pulses at a wavelength of 380 nm from an 80 MHz titanium:sapphire laser (Chameleon Ulltra II, Coherent) . Excitation power was 3 mW. The photoluminescence transients shown in this work are taken from the spectral regions of 420 to 620 nm.

Photoluminescence quantum yield (**PLQY**) values were measured in a home-built setup. The excitation laser (405 nm, DL5146-101S, Thorlabs Inc.) was directed in an integrating sphere (Labsphere) with a diameter of 15 cm. The luminescence was detected with a CCD spectrometer (AvaSpec-2048x64-TEC, Avantes). The measurement system (integrating sphere – optical fiber – spectrometer) was irradiance calibrated using a calibrating lamp (HL-3P, Ocean Optics). PLQY was measured according to a 3-measurement procedure published elsewhere.[1, 2] Measurement and calculation routine was automated using a Labview program. It should be pointed out that for the measured compounds 2-measurement and 3-measurement PLQY values were coinciding with 99% accuracy.

1.3 Synthesis of the Building Blocks

Synthesis of 1,4-dipropoxybenzene

The Williamson Ether syntheses are based on a procedure published by H. Meier *et al.*[3]

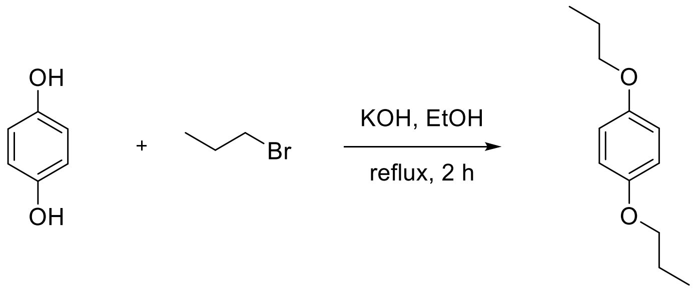

Hydroquinone (30.0 g, 272 mmol, 1.00 eq.) was dissolved in 250 mL absolute ethanol. Potassium hydroxide (38.2 g, 681 mmol, 2.50 eq.) was added and the mixture was stirred for 30 minutes under reflux. Subsequently, 1-bromopropane (54.7 mL, 73.8 g, 600 mmol, 2.20 eq.) was slowly added over a 1 h time period and stirred under reflux for another 2 h. Ethanol was removed with a rotary evaporator and the residue was taken up in dichloromethane. The organic phase was washed with water three times and once more with saturated $NaHCO_3$ solution. It was then dried over Na_2SO_4, filtered and the solvent was removed under reduced pressure and the crude product was recrystallised from methanol to yield colourless crystals (40.0 g, 76%). TLC (hexane / dichloromethane 9:1) R_f = 0.27; ^{1}H NMR ($CDCl_3$, 300 MHz): δ (ppm) = 6.83 (d, J = 0.9 Hz, 4 H, 4 C**H**$_{\textbf{aromatic}}$), 3.87 (t, J = 6.6 Hz, 4 H, 2 C**H₂**O), 1.79 (sex, J = 7.4 Hz, 4 H, 2 C**H₂**CH₃), 1.03 (t, J = 7.4 Hz, 6 H, 2 C**H₃**); ^{13}C NMR ($CDCl_3$, 75 MHz): δ (ppm) = 153.28, 115.41, 70.12, 22.77, 10.56; FAB of $C_{12}H_{18}O_2$ (M+H$^+$ = 195.1); HRMS (FAB) of $C_{12}H_{18}O_2$ [M+H$^+$] calc. 194.1301, found 194.1299; IR (ATR) v = 2962.6, 2935.5, 2874.7, 1504.8, 1460.9, 1391.7, 1275.2, 1218.5, 1115.5, 1068.7, 1049.5, 1025.8, 1005.1, 978.7, 824.2, 805.7, 770.2, 723.0, 531.2 cm^{-1}.

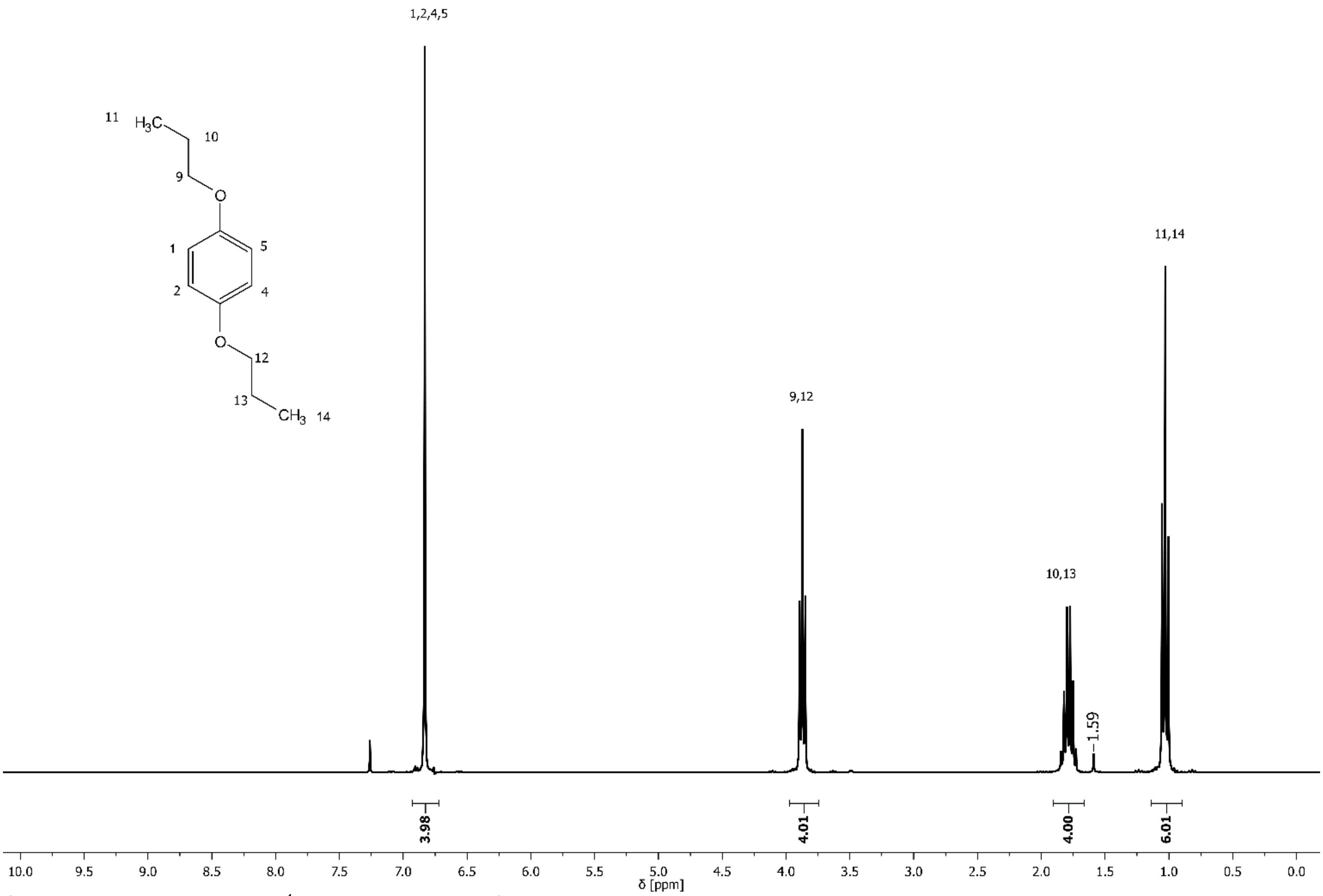

Supplementary Figure 1: ^1H NMR spectrum of 1,4-dipropoxybenzene with assigned signals.

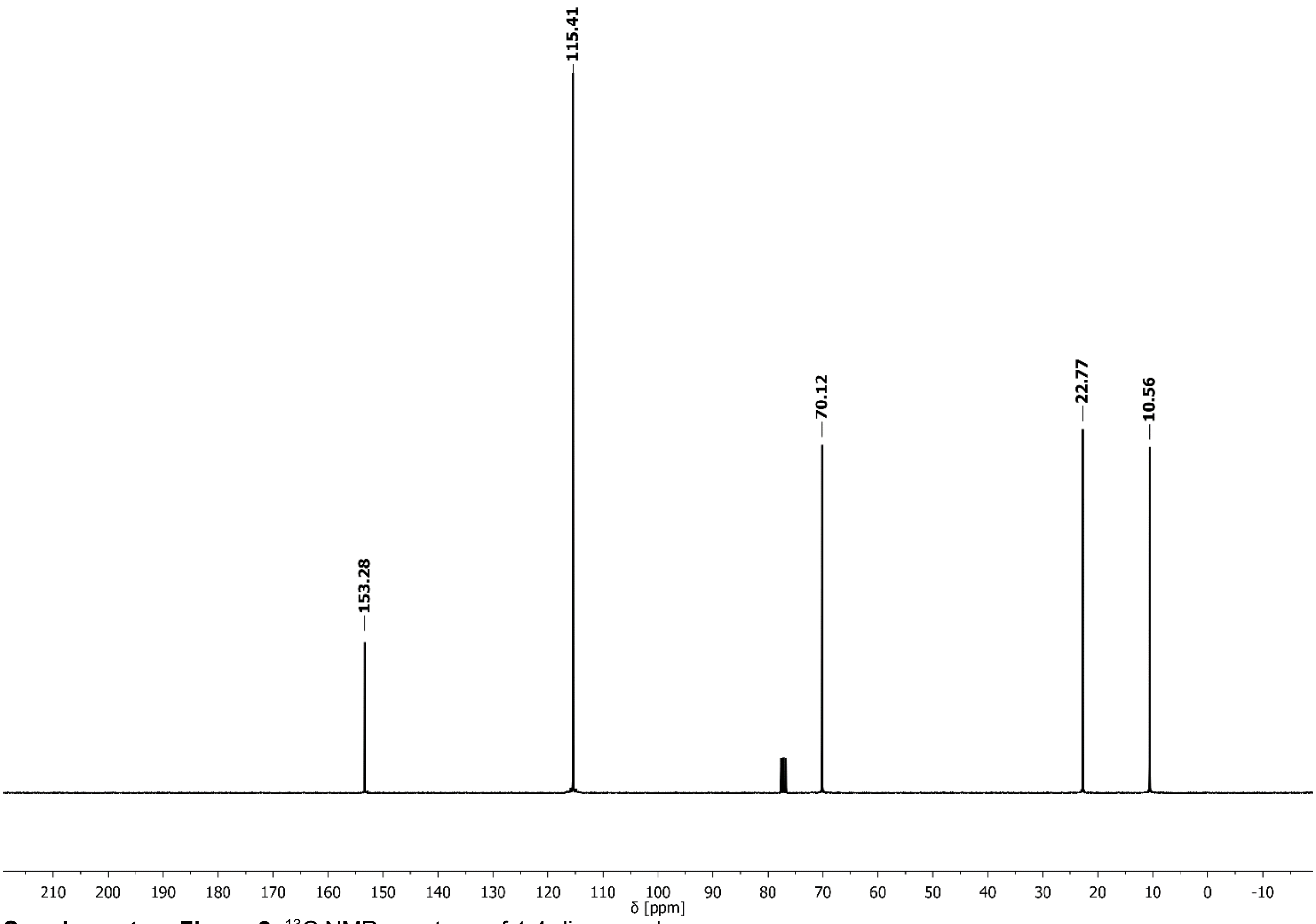

Supplementary Figure 2: ^{13}C NMR spectrum of 1,4-dipropoxybenzene.

Synthesis of 1,4-diiodo-2,5-dipropoxybenzene

The iodinations are based on a procedure published by Park *et al.*[4]

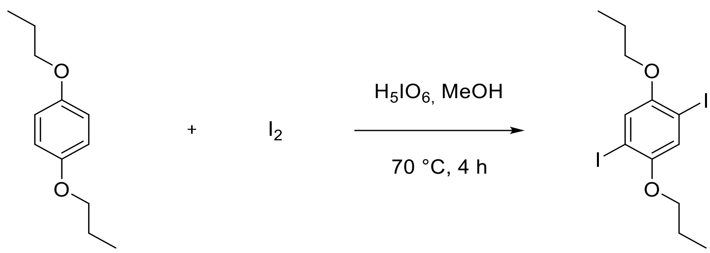

Periodic acid (3.20 g, 14.0 mmol, 0.636 eq.) was dissolved in 25 mL methanol and stirred for 10 minutes. Subsequently, iodine (6.97 g, 27.0 mmol, 1.23 eq.) was added and after an additional stirring time of 10 minutes, 1,4-dipropoxybenzene (4.27 g, 22.0 mmol, 1.00 eq.) was added. The reaction mixture was stirred at 70 °C for 4 h. The residue was carefully poured into 50 mL water containing potassium disulfite. The precipitate was washed with methanol and dissolved in dichloromethane. The solution was filtered, and the filtrate was concentrated under reduced pressure. The residue was purified by recrystallisation from methanol to yield the product as a white solid (8.20 g, 84%). TLC (hexane / dichloromethane 9:1) R_f = 0.38; ^{1}H NMR ($CDCl_3$, 300 MHz): δ (ppm) = 7.17 (s, 2 H, 2 C**H$_{aromatic}$**), 3.90 (t, J = 6.4 Hz, 4 H, 2 C**H$_2$**O), 1.83 (sex, J = 7.4 Hz, 4 H, 2 C**H$_2$**CH_3), 1.07 (t, J = 7.4 Hz, 6 H, 2 C**H$_3$**); ^{13}C NMR ($CDCl_3$, 75 MHz): δ (ppm) = 152.87, 122.83, 86.43, 71.88, 22.69, 10.82; FAB of $C_{12}H_{16}I_2O_2$ (M+H$^+$ = 446.9); HRMS (FAB) of $C_{12}H_{16}I_2O_2$ [M+H$^+$] calc. 445.9234, found 445.9234; IR (ATR) v = 2957.9, 2907.4, 2869.2, 1680.4, 1486.6, 1461.7, 1446.9, 1392.1, 1347.0, 1262.8, 1205.8, 1054.0, 1005.3, 909.4, 849.7, 795.2, 768.0, 621.1, 434.0, 394.9 cm^{-1}.

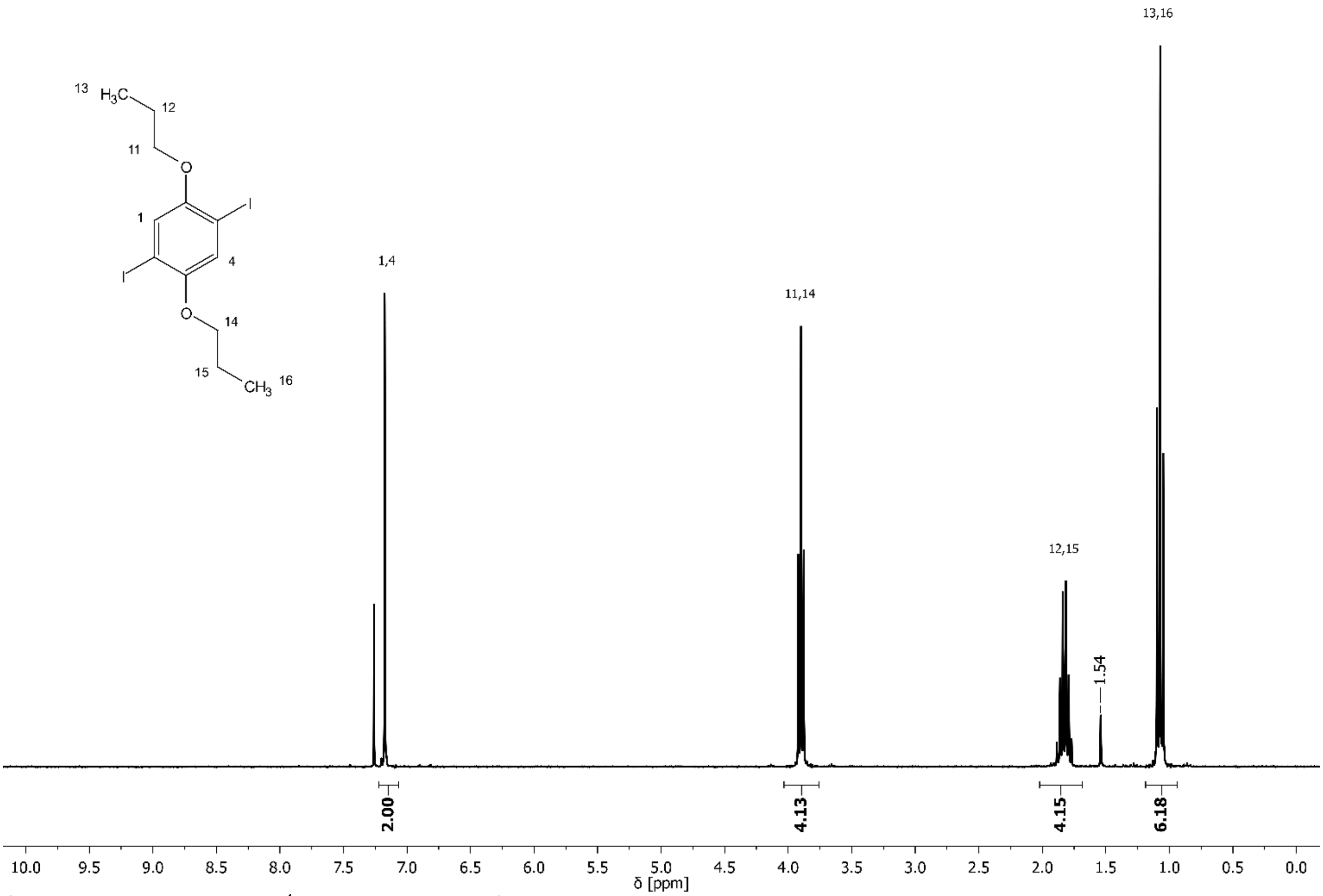

Supplementary Figure 3: ^{1}H NMR spectrum of 1,4-diodo-2,5-dipropoxybenzene with assigned signals.

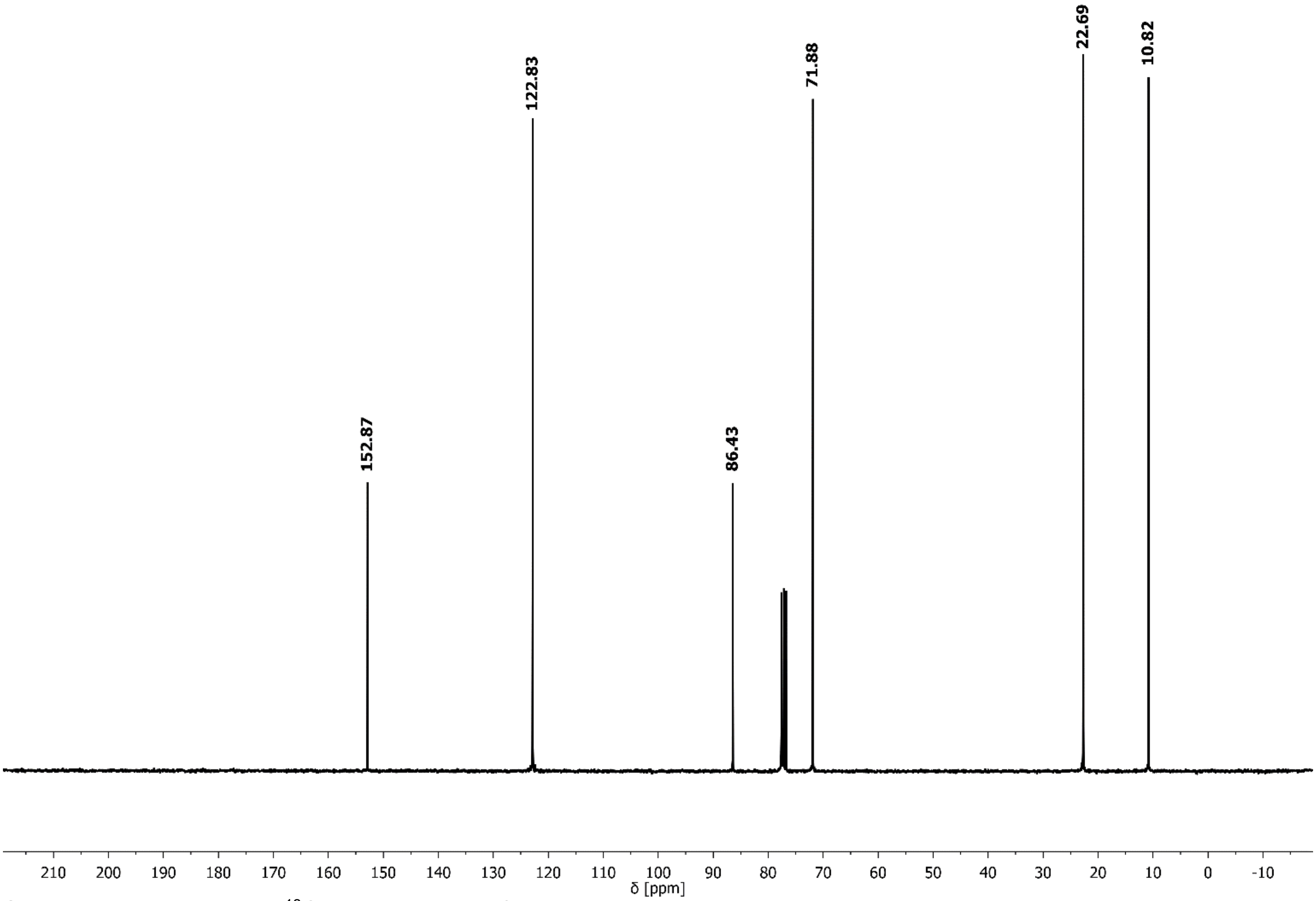

Supplementary Figure 4: ^{13}C NMR spectrum of 1,4-diodo-2,5-dipropoxybenzene.

Synthesis of 1,4-Bis(propyloxy)-2-iodo-5-trimethylsilylacetylenebenzene **1**

The Sonogashira reactions are based on a procedure published by Tour *et al.*[5] All Sonogashira reactions were performed under continuous argon atmosphere.

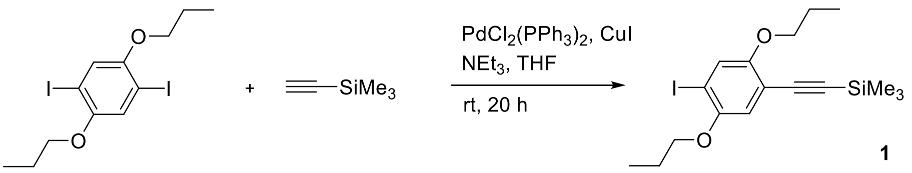

1,4-Diiodo-2,5-dipropoxybenzene (10.0 g, 22.4 mmol, 1.00 eq.), 2.5 mol% *bis*(triphenylphosphine)palladium(II) dichloride (393 mg, 0.560 mmol) and 5 mol% copper(I) iodide (214 mg, 1.12 mmol) were placed into a Schlenk flask and degassed. Under continuous argon flow, 400 mL dry THF and 31.1 mL dry triethylamine were added, and the mixture was stirred for 10 minutes. Subsequently, 3.41 mL trimethylsilylacetylene (2.42 g, 24.7 mmol, 1.10 eq.) with 5 mL dry THF was added dropwise with a syringe. The reaction mixture was stirred for 20 h at room temperature, taken up in dichloromethane and washed with saturated NH_4Cl solution. The aqueous phase was extracted three times with dichloromethane. The combined organic layers were dried over Na_2SO_4, filtered and concentrated under reduced pressure. The residue was purified by silica column chromatography (cyclohexane / dichloromethane 9:1) to yield the product as a yellow solid (4.26 g, 46%). TLC (hexane / dichloromethane 9:1) R_f = 0.25; ^{1}H NMR ($CDCl_3$, 300 MHz): δ (ppm) = 7.26 (s, 1 H, 1 C$\mathbf{H}_{\mathbf{aromatic}}$Cl), 6.84 (s, 1 H, 1 C$\mathbf{H}_{\mathbf{aromatic}}$C-C≡C), 3.91 (t, *J* = 6.4 Hz, 4 H, 2 C$\mathbf{H_2}$O), 1.65-2.00 (m, 4 H, 2 C**H**$_2$CH$_3$), 1.07 (t, *J* = 7.4 Hz, 6 H, 2 C$\mathbf{H_3}$), 0.25 (s, 9 H, 3 CH_3Si); ^{13}C NMR ($CDCl_3$, 75 MHz): δ (ppm) = 154.99, 151.81, 124.14, 116.38, 113.65, 100.91, 99.54, 88.02, 71.67, 71.45, 22.80, 22.73, 10.83, 10.60, 0.06; FAB of $C_{17}H_{25}IO_2Si$ ($M+H^+$ = 417.1); HRMS (FAB) of $C_{17}H_{25}IO_2Si$ [$M+H^+$] calc. 416.0663, found 416.0662; IR (ATR) v = 2952.6, 2872.0, 2157.4, 1498.6, 1485.5, 1456.6, 1369.2, 1288.9, 1253.9, 1244.0, 1213.8, 1162.9, 1030.7, 1014.4, 972.7, 906.2, 856.1, 833.5, 756.9, 696.3, 664.4, 636.8, 491.3, 395.9 cm^{-1}.

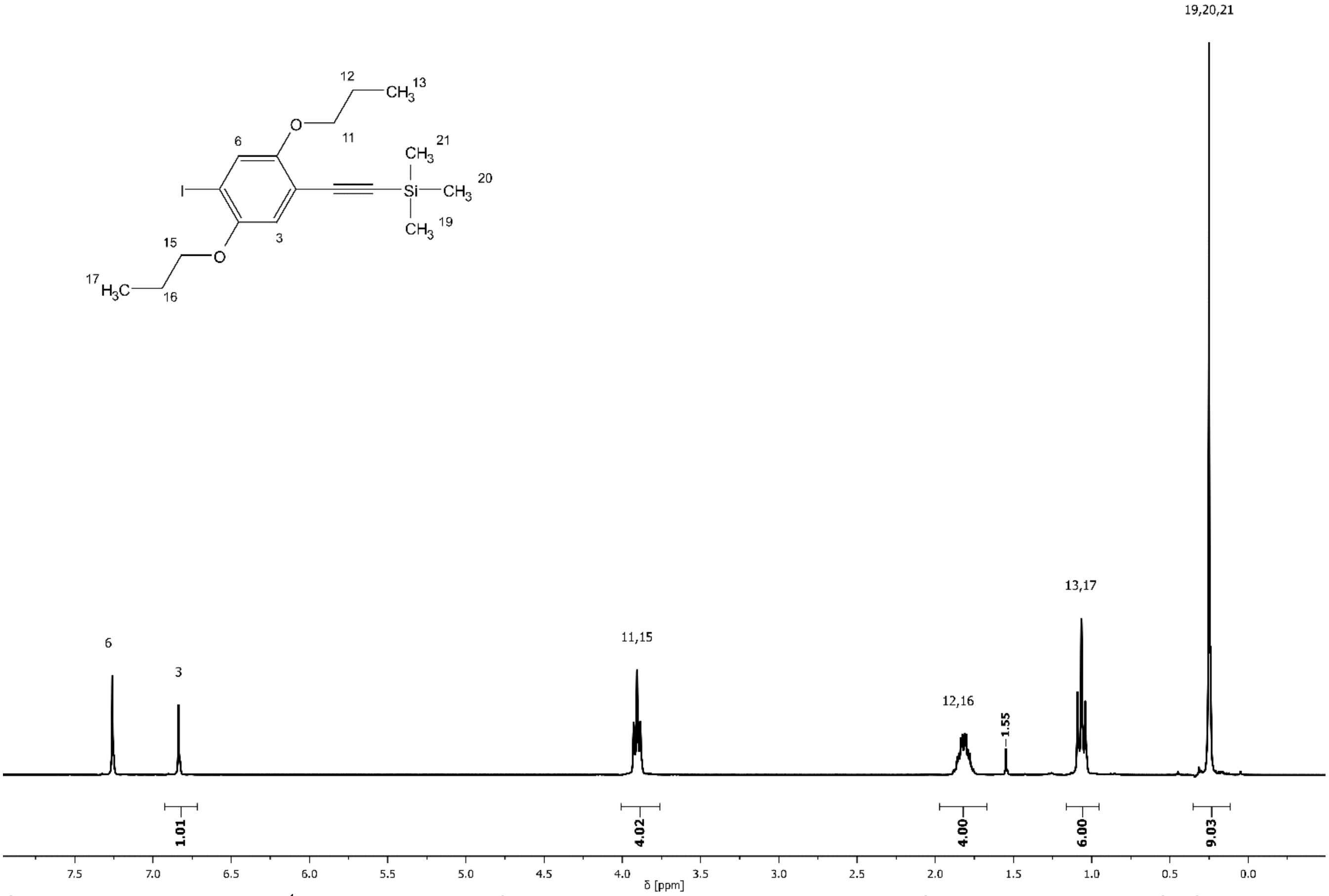

Supplementary Figure 5: ^{1}H NMR spectrum of building block **1** with assigned signals. Signal 6 overlays with $CDCl_3$.

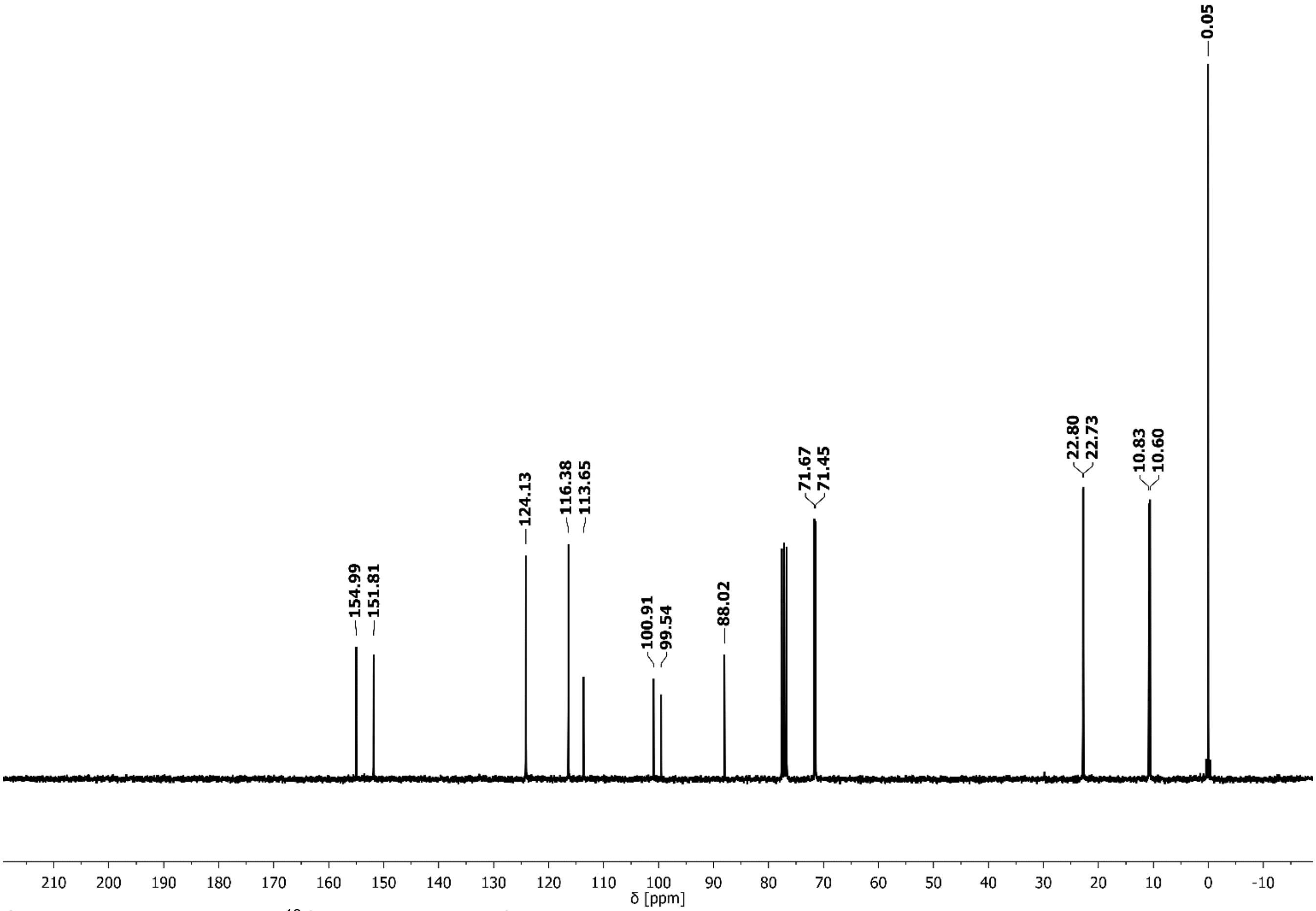

Supplementary Figure 6: ^{13}C NMR spectrum of building block **1**.

Synthesis of 1,4-diisopropoxybenzene

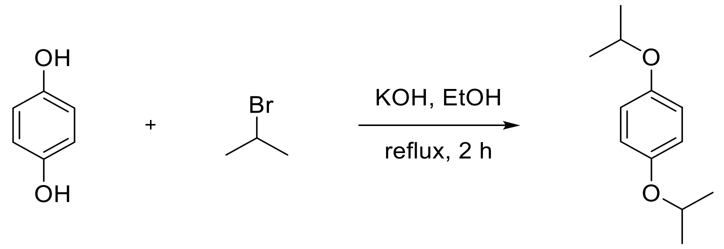

Hydroquinone (30.0 g, 272 mmol, 1.00 eq.) was dissolved in 250 mL absolute ethanol. Potassium hydroxide (38.2 g, 681 mmol, 2.50 eq.) was added and the mixture was stirred for 30 minutes under reflux. Subsequently, 2-bromopropane (54.7 mL, 73.8 g, 600 mmol, 2.20 eq.) was slowly added over a 1 h time period and stirred under reflux for another 2 h. Ethanol was removed with a rotary evaporator and the residue was taken up in dichloromethane. The organic phase was washed with water three times and once more with saturated $NaHCO_3$ solution. It was then dried over Na_2SO_4, filtered and the solvent was removed under reduced pressure. The crude product was purified by silica column chromatography (cyclohexane / ethyl acetate 20:1) to yield the product as a yellow oil (40.0 g, 76%). TLC (hexane / dichloromethane 4:1) R_f = 0.26; ^{1}H NMR ($CDCl_3$, 300 MHz): δ (ppm) = 6.81 (s, 4 H, 4 C**H**$_{\mathbf{aromatic}}$), 4.42 (hept, J = 6.1 Hz, 2 H, 2 C**H**CH_3), 1.31 (d, J = 6.1 Hz, 12 H, 4 C**H**$_{\mathbf{3}}$); ^{13}C NMR ($CDCl_3$, 75 MHz): δ (ppm) = 152.01, 117.36, 70.77, 22.19; FAB of $C_{12}H_{18}O_2$ ($M+H^+$ = 195.1); HRMS (FAB) of $C_{12}H_{18}O_2$ [$M+H^+$] calc. 194.1301, found 194.1301; IR (ATR) v = 2972.7, 1501.5, 1382.0, 1212.3, 1113.1, 957.1, 827.9, 750.4, 527.2 cm^{-1}.

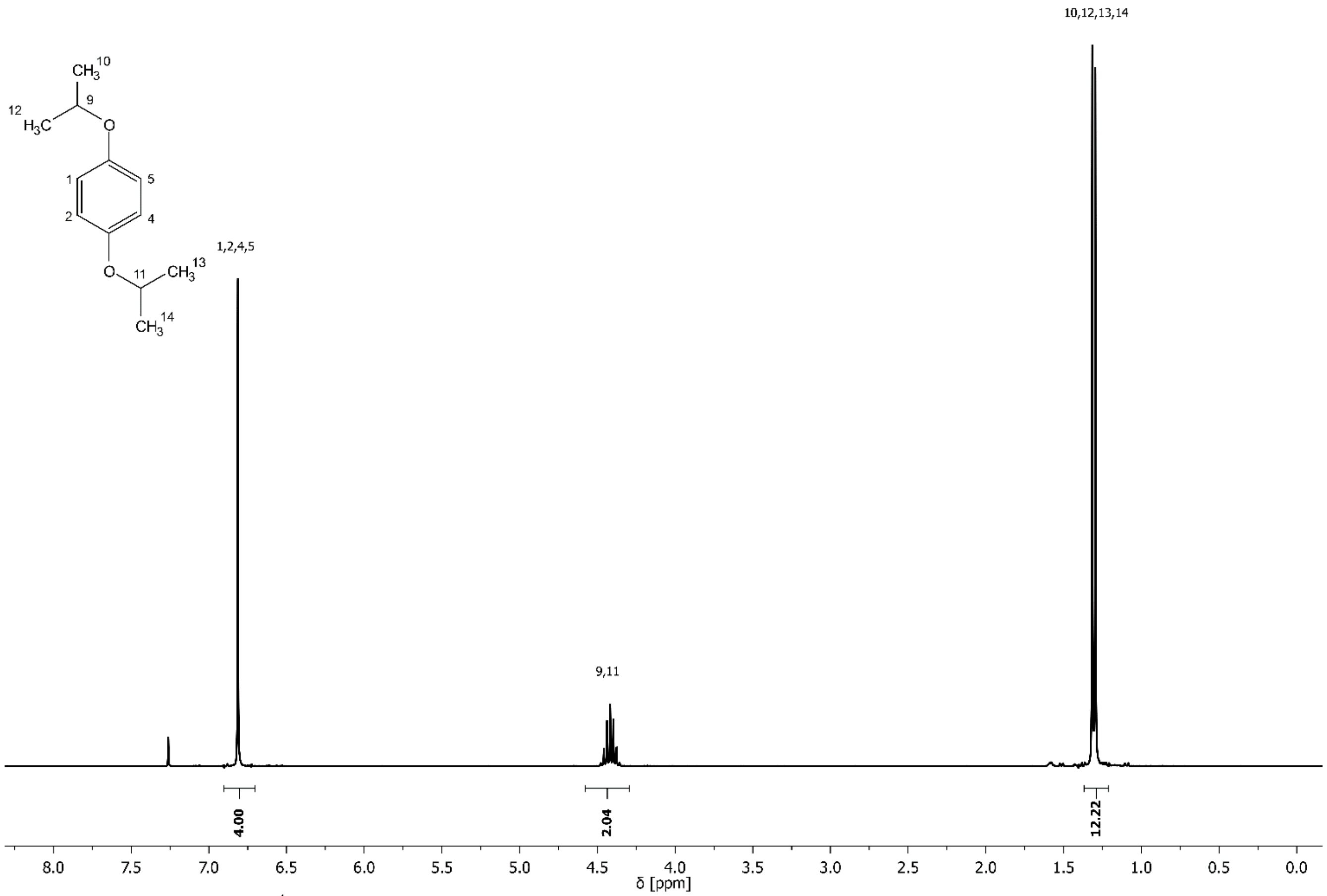

Supplementary Figure 7: ^1H NMR spectrum of 1,4-diisopropoxybenzene with assigned signals.

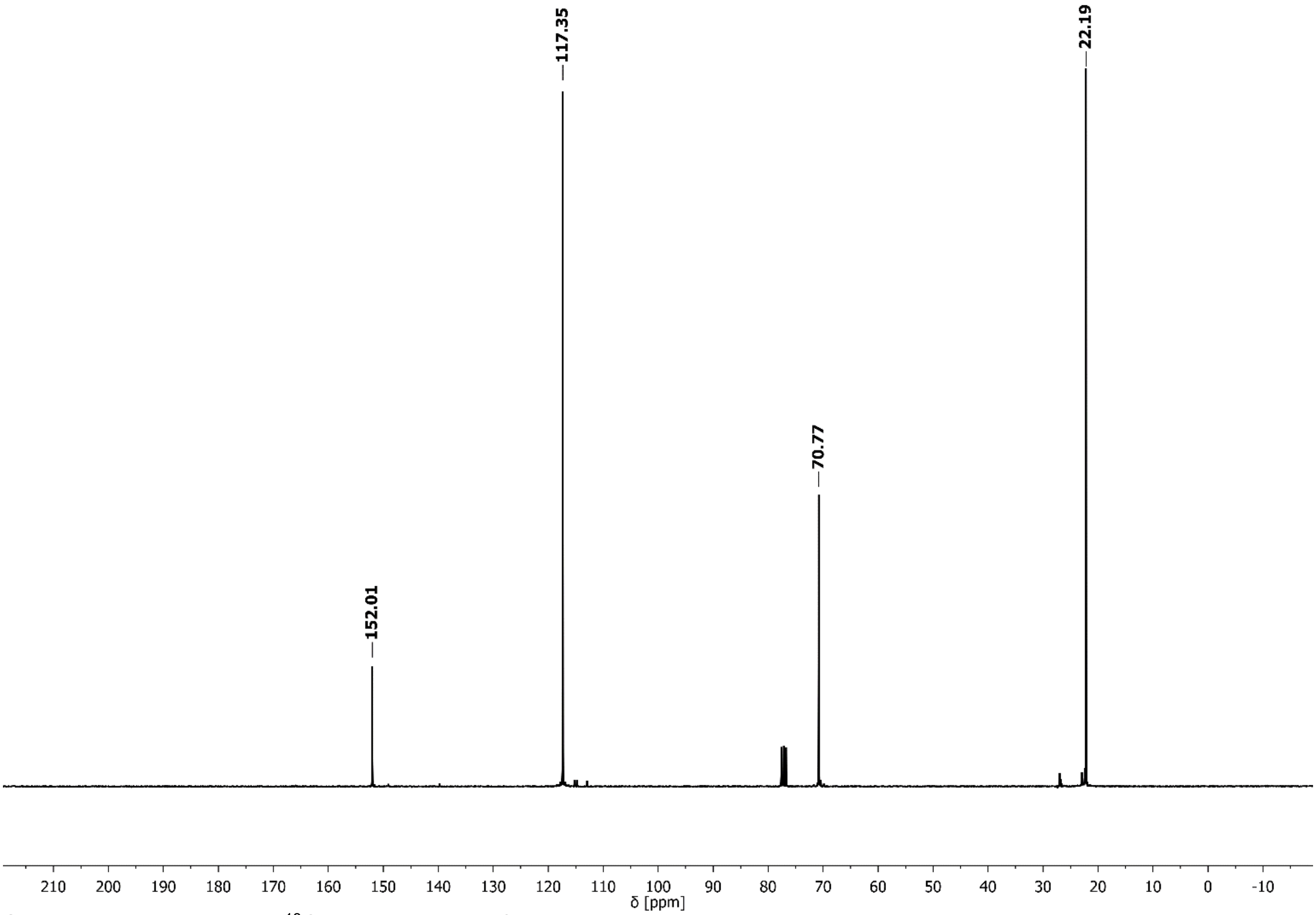

Supplementary Figure 8: ^{13}C NMR spectrum of 1,4-diisopropoxybenzene with assigned signals.

Synthesis of 1,4-diiodo-2,5-diisopropoxybenzene

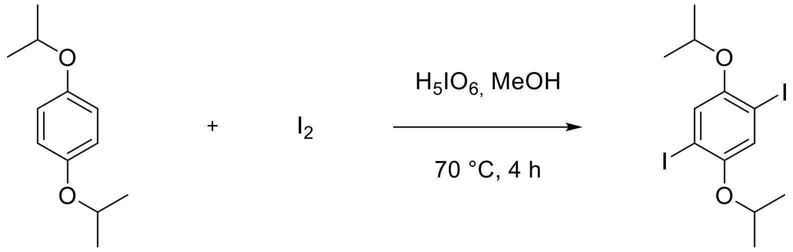

Periodic acid (6.40 g, 28.0 mmol, 0.636 mmol) was dissolved in 50 mL methanol and stirred for 10 minutes. Subsequently, iodine (13.9 g, 54.0 mmol, 1.23 mmol) was added and after an additional stirring time of 10 minutes, 1,4-diisopropoxybenzene (8.54 g, 44.0 mmol, 1.00 eq.) was added. The reaction mixture was stirred at 70 °C for 4 h. Subsequently, the residue was carefully poured into 50 mL water containing potassium disulfite. The aqueous phase was extracted three times with dichloromethane, dried over Na_2SO_4, filtered and the solvent was removed under reduced pressure. The crude product crystallised overnight and was purified by recrystallisation from methanol to yield the product as a white solid (12.5 g, 64%). TLC (cyclohexane / dichloromethane 4:1) R_f = 0.43; ^{1}H NMR ($CDCl_3$, 300 MHz): δ (ppm) = 7.21 (s, 2 H, 2 C**H$_{aromatic}$**), 4.41 (hept, J = 6.1 Hz, 2 H, 2 C**H**CH_3), 1.36 (d, J = 6.1 Hz, 12 H, 4 C**H$_3$**); ^{13}C NMR ($CDCl_3$, 75 MHz): δ (ppm) = 152.25, 125.55, 88.69, 73.65, 22.24; FAB of $C_{12}H_{16}I_2O_2$ (M+H$^+$ = 446.7); HRMS (FAB) of $C_{12}H_{16}I_2O_2$ [M+H$^+$] calc. 445.9234, found 445.9233; IR (ATR) ν = 2968.9, 2924.6, 1450.8, 1371.5, 1347.6, 1328.9, 1251.4, 1198.2, 1139.9, 1098.2, 1044.3, 944.2, 867.2, 854.1, 762.6, 626.3, 468.3, 423.8 cm^{-1}.

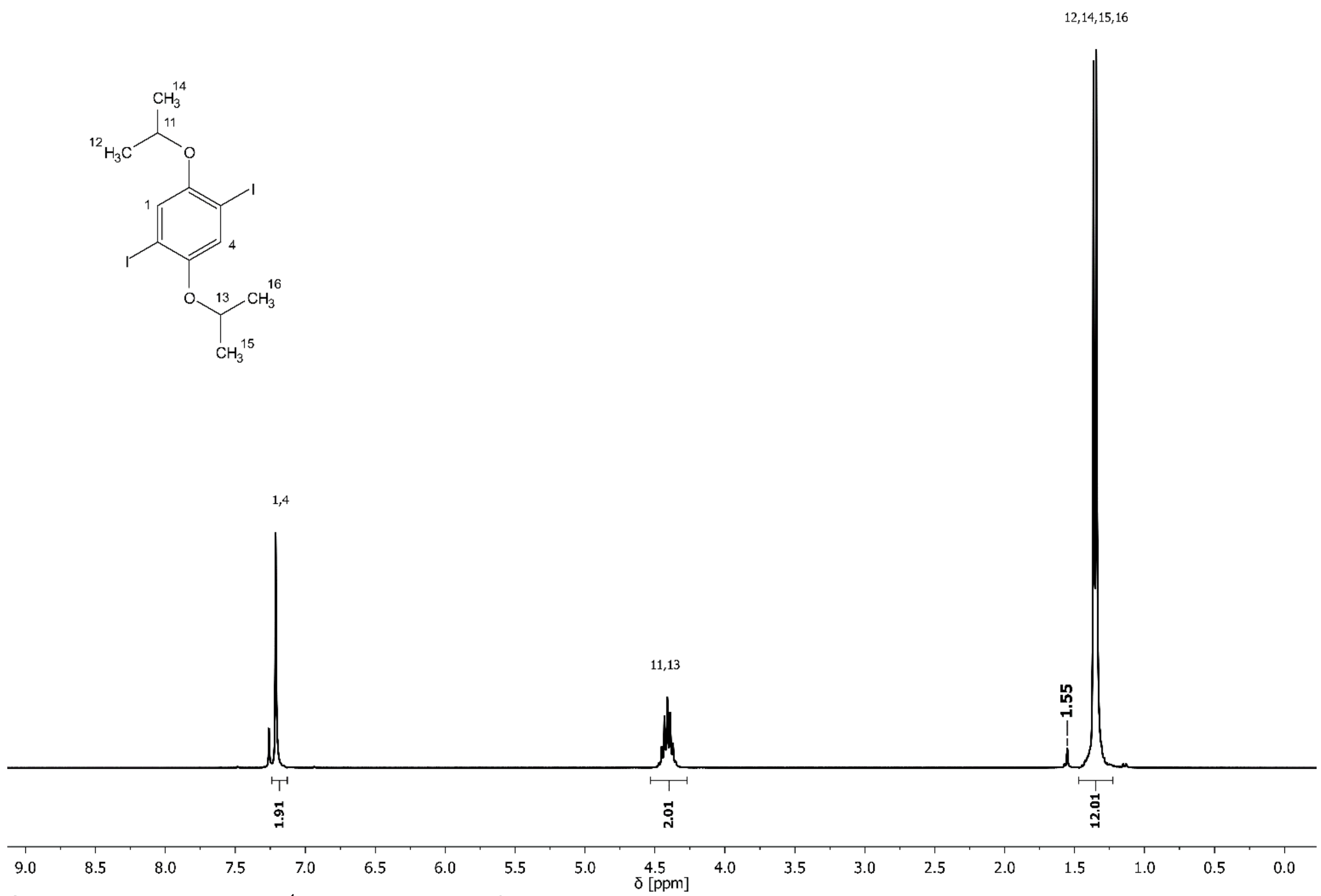

Supplementary Figure 9: ^{1}H NMR spectrum of 1,4-diodo-2,5-diisopropoxybenzene with assigned signals.

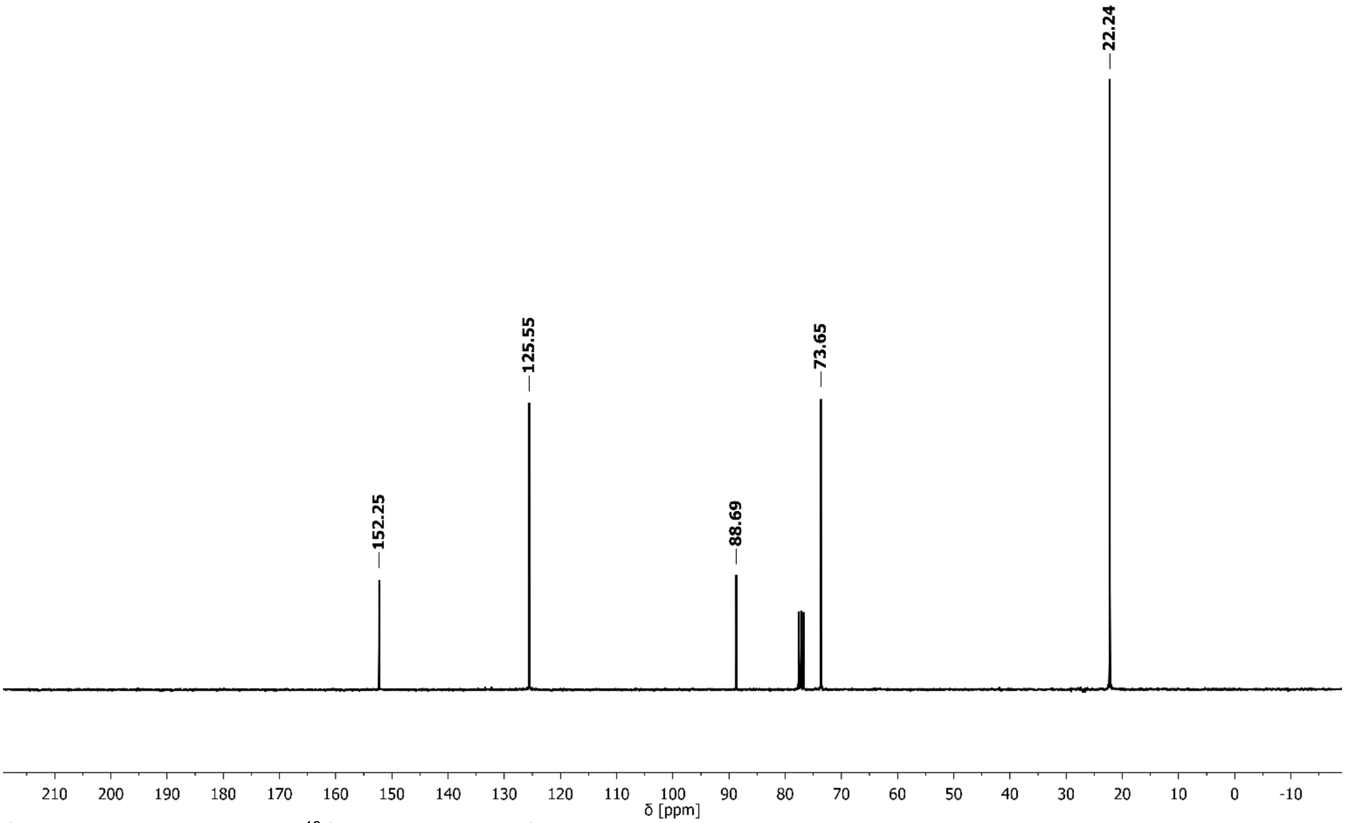

Supplementary Figure 10: ^{13}C NMR spectrum of 1,4-diodo-2,5-diisopropoxybenzene.

Synthesis of 1,4-Bis(isopropyloxy)-2-iodo-5-trimethylsilylacetylenebenzene

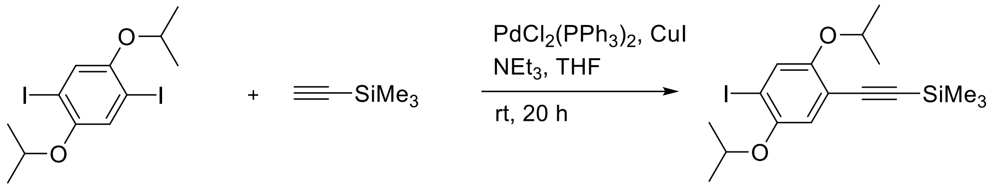

1,4-Diiodo-2,5-diisopropoxybenzene (500 mg, 1.12 mmol, 1.00 eq.), 2.5 mol% bis(triphenylphosphine)palladium(II) dichloride (19.7 mg, 28.0 µmol) and 5 mol% copper(I) iodide (10.7 mg, 56.0 µmol) were placed into a Schlenk flask. Under continuous argon flow, 20 mL dry THF and 1.55 mL dry triethylamine were added and the mixture was stirred for 0.5 h. Subsequently, trimethylsilylacetylene (191 µL, 132 mg, 1.34 mmol, 1.20 eq.) in 4 mL THF was added dropwise with a syringe. The reaction mixture was stirred for 24 h at room temperature, taken up in dichloromethane and washed with saturated NH_4Cl solution. The aqueous phase was extracted three times with dichloromethane. The combined organic layers were dried over Na_2SO_4, filtered and concentrated under reduced pressure. The residue was purified by silica column chromatography (cyclohexane / dichloromethane 5:1) to yield the product as a yellow liquid (165 mg, 35%). TLC (cyclohexane / dichloromethane 5:1) R_f = 0.29; ^{1}H NMR ($CDCl_3$, 300 MHz): δ (ppm) = 7.31 (s, 1 H, 1 C**H**$_{\textbf{aromatic}}$CI), 6.86 (s, 1 H, 1 C**H**$_{\textbf{aromatic}}$C-C≡C), 4.44 (hept, *J* = 6.2 Hz, 2 H, 2 C**H**CH_3), 1.34 (dd, *J* = 9.7, 6.1 Hz, 12 H, 4 C**H**$_{\textbf{3}}$), 0.25 (s, 9 H, 3 C**H**$_{\textbf{3}}$Si); ^{13}C NMR ($CDCl_3$, 75 MHz): δ (ppm) = 154.13, 151.51, 128.55, 118.92, 115.91, 101.24, 99.37, 90.11, 73.99, 73.27, 22.31, 22.27, 0.06; FAB of $C_{17}H_{25}IO_2Si$ (M+H$^+$ = 417.0); HRMS (FAB) of $C_{17}H_{25}IO_2Si$ [M+H$^+$] calc. 416.0663, found 416.0661; IR (ATR) ν = 2973.6, 2150.5, 1472.5, 1372.8, 1331.0, 1247.7, 1197.3, 1154.3, 1136.5, 1104.5, 1002.2, 944.1, 838.9, 757.9, 698.0, 661.4, 626.4, 417.4 cm^{-1}.

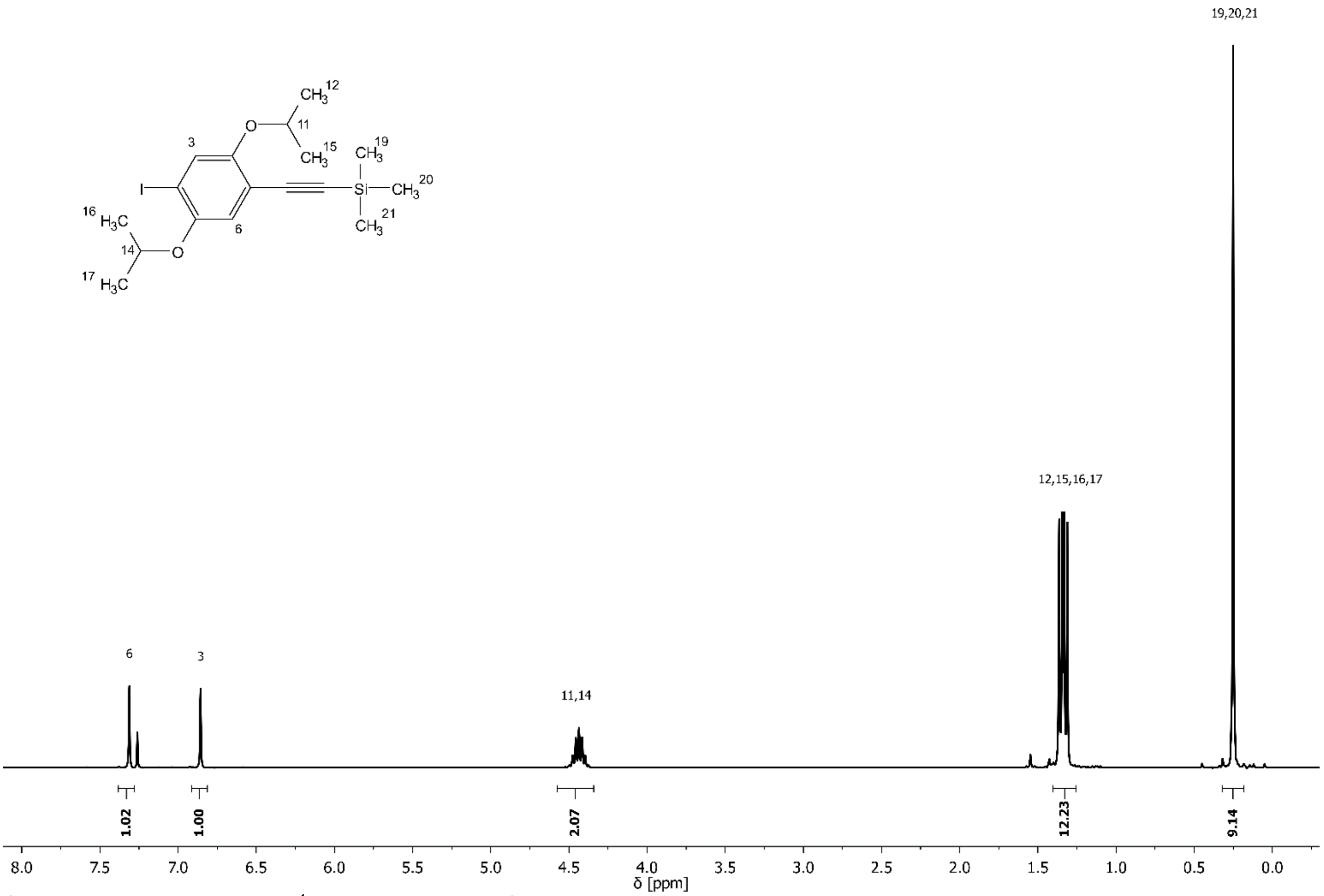

Supplementary Figure 11: ^{1}H NMR spectrum of the diisopropoxy-building block with assigned signals.

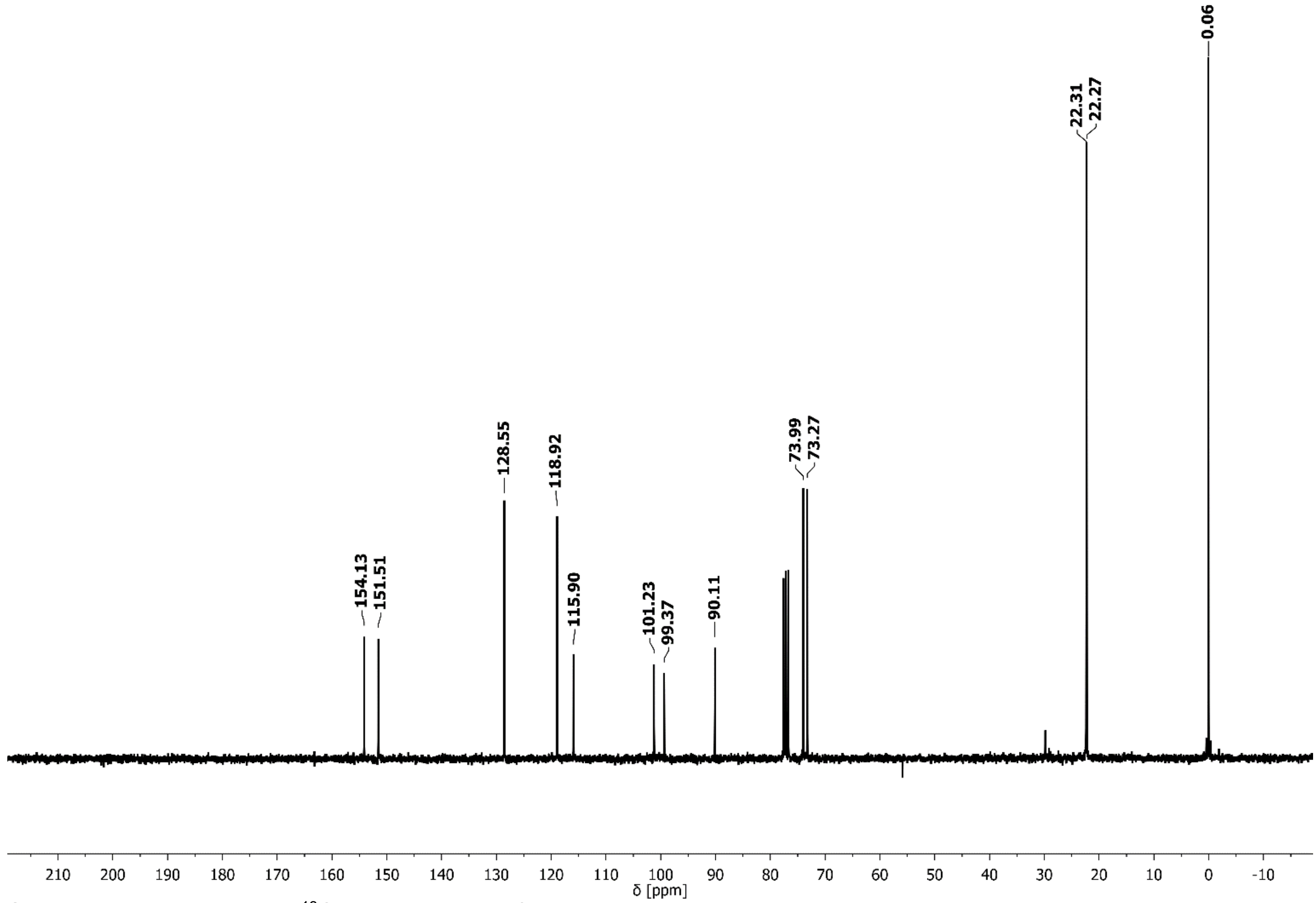

Supplementary Figure 12: ^{13}C NMR spectrum of the diisopropoxy-building block.

Synthesis of 1,4-bis(cyclohexyloxy)benzene

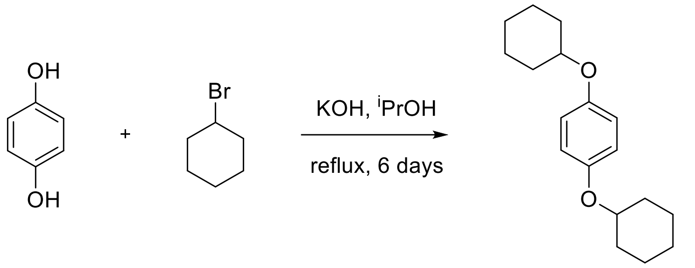

Hydroquinone (10.0 g, 90 8 mmol, 1.00 eq.) was dissolved in 100 mL isopropanol. Potassium hydroxide (12.7 g, 227 mmol, 1.25 eq. per hydroxy group) was added and the mixture was stirred for 30 minutes under reflux. Subsequently, bromocyclohexane (24.5 mL, 32.6 g, 200 mmol, 1.10 eq. per hydroxy group) was slowly added over a 1 h time period and stirred under reflux for another 4 h. The conversion was regularly monitored by GC-MS. If necessary, further potassium hydroxide (6.00 g, 107 mmol) or bromocyclohexane (12.0 mL, 15.9 g, 97.4 mmol) was added. After 6 days, the GC-MS confirmed full conversion and the isopropanol was removed with a rotary evaporator. The residue was taken up in dichloromethane, washed with water three times and once more with saturated $NaHCO_3$ solution. It was then dried over Na_2SO_4, filtered and the solvent was removed under reduced pressure and the crude product was recrystallised from methanol to yield colorless crystals (6.21 g, 28%), TLC (cyclohexane / dichloromethane 9:1) R_f = 0.30; ^{1}H NMR ($CDCl_3$, 300 MHz): δ (ppm) = 6.82 (s, 4 H, 4 C**H**$_{\textbf{aromatic}}$), 4.21-3.97 (m, 2 H, 2 C**H**O), 2.11-1.89 (m, 4 H, 4 C**H**$_{\textbf{equatorial}}$CHO), 1.89-1.68 (m, 4 H, 4 C**H**$_{\textbf{equatorial}}$$CH_2$CHO), 1.66-1.41 (m, 6 H, 4 C**H**$_{\textbf{axial}}$CHO, 2 C**H**$_{\textbf{equatorial}}$$CH_2CH_2$CHO), 1.41-1.20 (m, 6 H, 4 C**H**$_{\textbf{axial}}$$CH_2$CHO, 2 C**H**$_{\textbf{axial}}$$CH_2CH_2$CHO); ^{13}C NMR ($CDCl_3$, 75 MHz): δ (ppm) = 151.93, 117.57, 76.56, 32.09, 25.79, 23.95; FAB of $C_{18}H_{26}O_2$ (M+H$^+$ = 275.3); HRMS (FAB) of $C_{18}H_{26}O_2$ [M+H$^+$] calc. 274.1927, found 274.1926; IR (ATR) v = 2929.3, 2852.1, 1503.0, 1454.4, 1379.7, 1357.0, 1283.6, 1256.5, 1210.7, 1148.9, 1118.2, 1089.9, 1050.9, 1019.9, 970.3, 889.1, 847.4, 829.8, 815.3, 766.5, 518.2, 470.9 cm^{-1}.

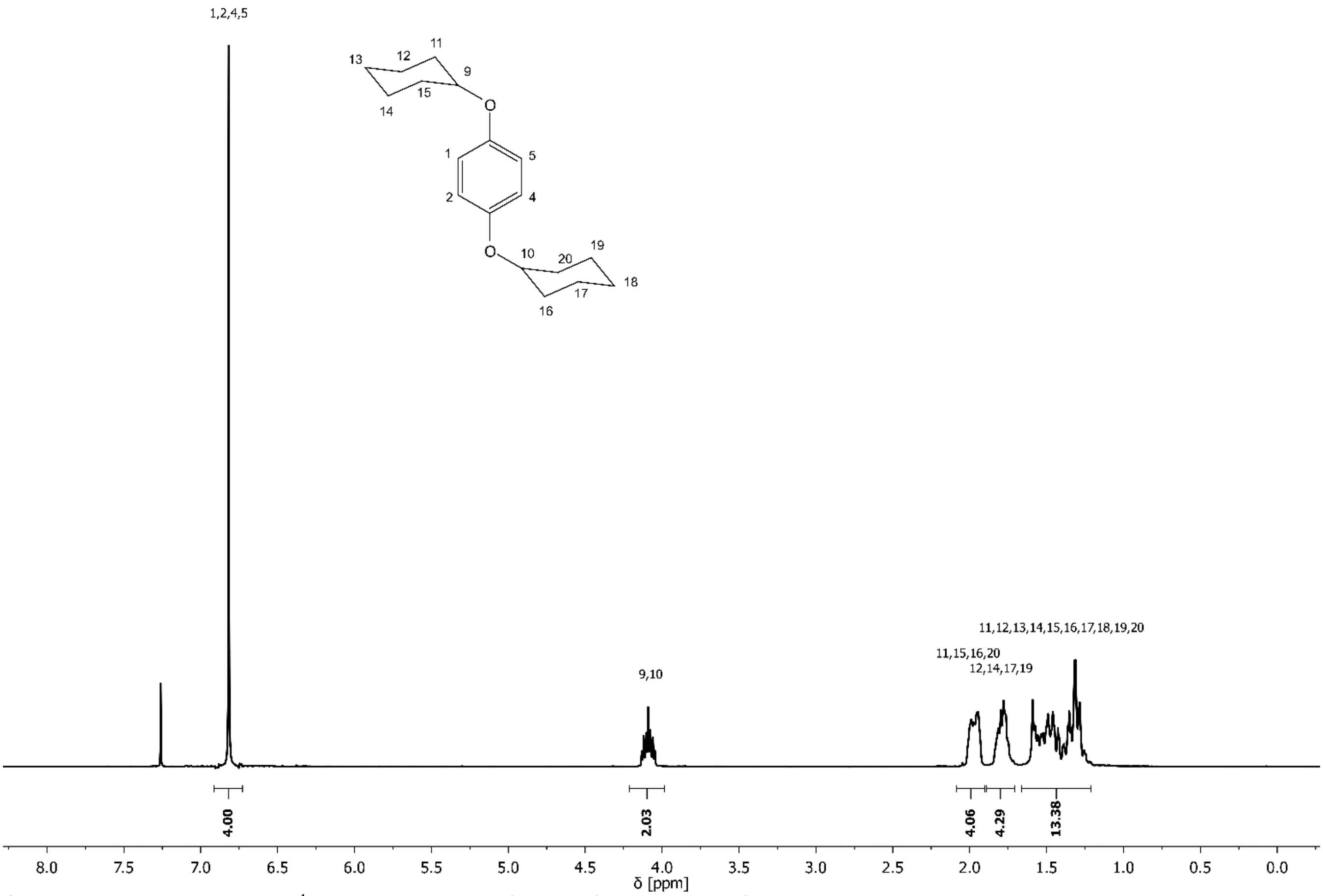

Supplementary Figure 13: ^{1}H NMR spectrum of 1,4-bis(cyclohexyloxy)benzene with assigned signals.

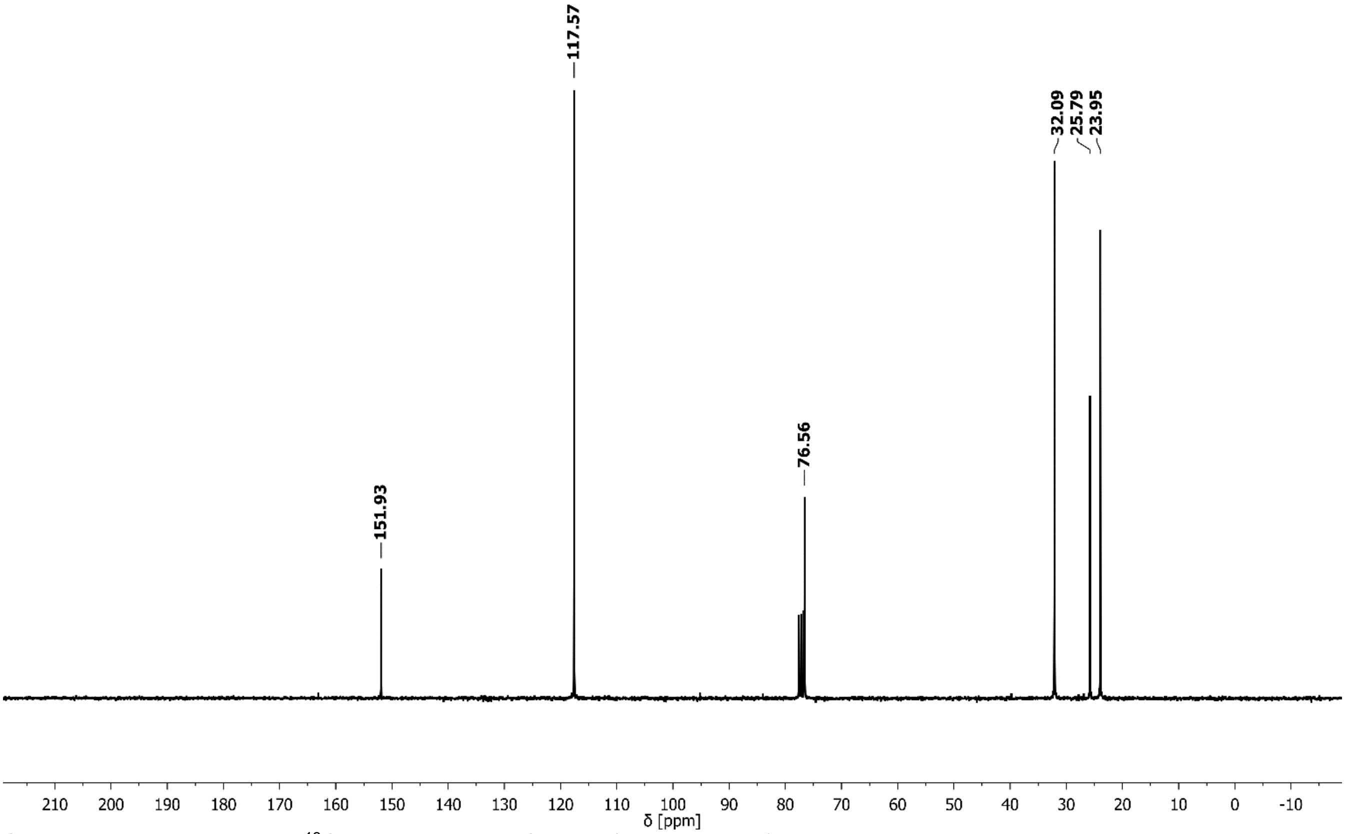

Supplementary Figure 14: ^{13}C NMR spectrum of 1,4-bis(cyclohexyloxy)benzene.

Synthesis of ((2,5-diiodo-1,4-phenylene)bis(oxy))dicyclohexane

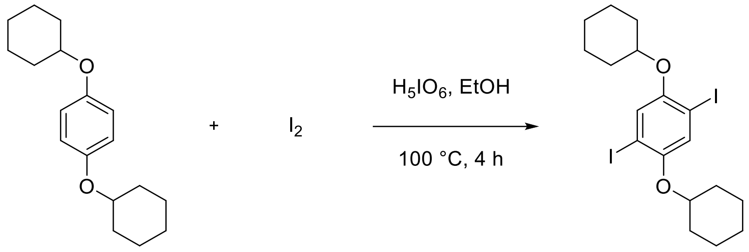

Periodic acid (1.69 g, 7.42 mmol, 0.636 eq.) was dissolved in 25 mL ethanol and stirred for 10 minutes. Subsequently, iodine (3.64 g, 14.3 mmol, 1.23 mmol) was added and after an additional stirring time of 10 minutes 1,4-bis(cyclohexyloxy)benzene (3.20 g, 11.7 mmol, 1.00 eq.) was added. The reaction mixture was stirred at 100 °C for 4 h. The residue was carefully poured into 50 mL water containing potassium disulfite. The precipitate was washed with ethanol and dissolved in dichloromethane. The solution was filtered, and the filtrate was concentrated under reduced pressure. The residue was purified by recrystallisation from ethanol to yield the product as a white solid (4.56 g, 74%). TLC (cyclohexane / dichloromethane 9:1) R_f = 0.64; ^{1}H NMR ($CDCl_3$, 300 MHz): δ (ppm) = 7.20 (s, 2 H, 2 C**H**$_{\textbf{aromatic}}$), 4.29-4.07 (m, 2 H, 2 C**H**O), 2.03-1.72 (m, 8 H, 4 C**H**$_{\textbf{equatorial}}$CHO, 4 C**H**$_{\textbf{equatorial}}$$CH_2$CHO), 1.72-1.57 (m, 4 H, 4 C**H**$_{\textbf{axial}}$CHO), 1.57-1.47 (m, 2 H, 2 C**H**$_{\textbf{equatorial}}$$CH_2CH_2$CHO), 1.47-1.21 (m, 6 H, 4 C**H**$_{\textbf{axial}}$$CH_2$CHO, 2 C**H**$_{\textbf{axial}}$$CH_2CH_2$CHO); ^{13}C NMR ($CDCl_3$, 75 MHz): δ (ppm) = 151.85, 125.27, 88.48, 78.09, 31.59, 25.68, 23.39; FAB of $C_{18}H_{24}I_2O_2$ ($M+H^+$ = 526.3); HRMS (FAB) of $C_{18}H_{24}I_2O_2$ [$M+H^+$] calc. 525.9860, found 525.9861; IR (ATR) v = 2925.5, 2845.8, 1472.2, 1452.6, 1364.2, 1342.1, 1315.5, 1259.3, 1234.7, 1200.3, 1153.7, 1120.2, 1047.1, 1024.2, 949.6, 864.0, 852.0, 802.8, 781.4, 650.6, 614.9, 501.5, 480.7, 451.7, 431.9 cm^{-1}.

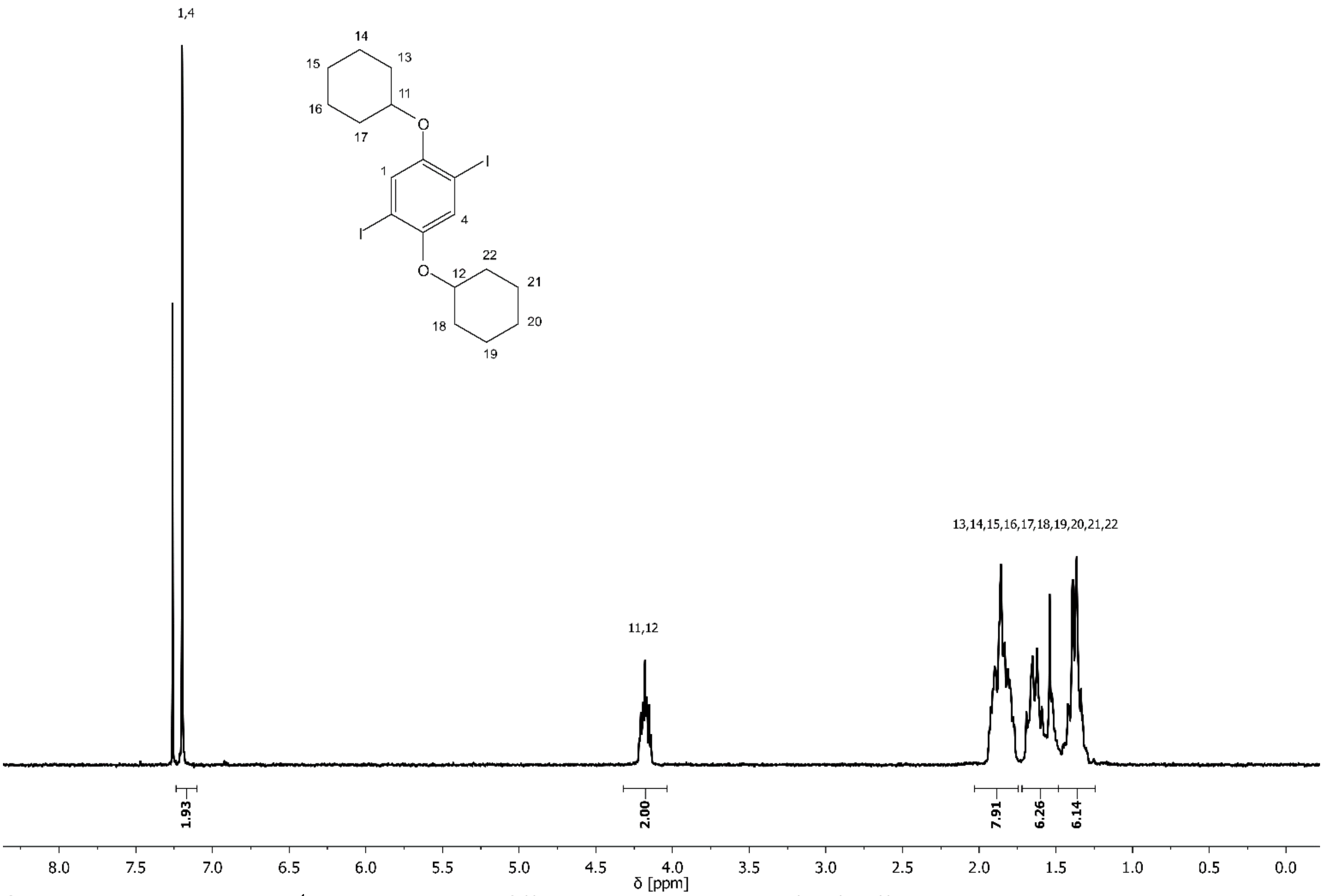

Supplementary Figure 15: ^{1}H NMR spectrum of ((2,5-diiodo-1,4-phenylene)bis(oxy))dicyclohexane with assigned signals.

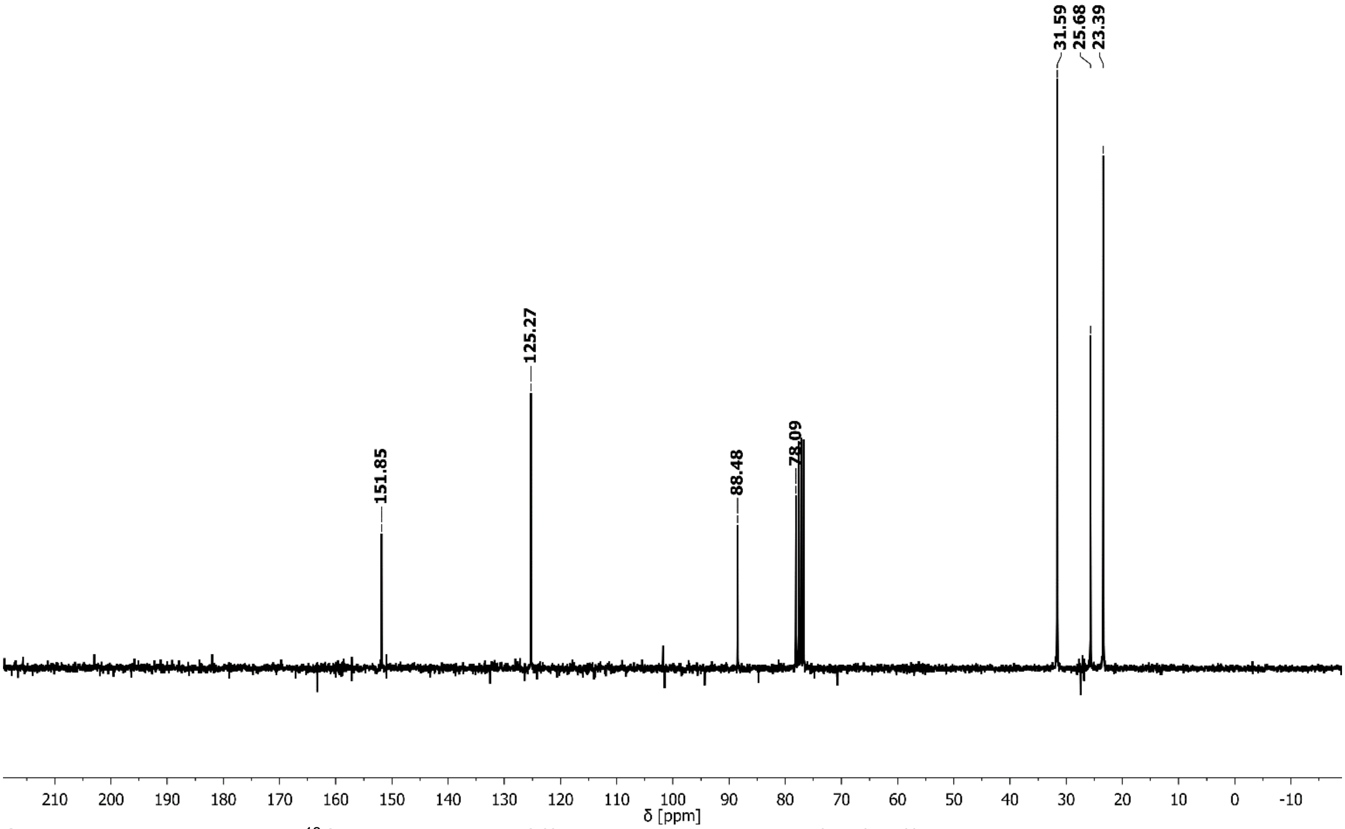

Supplementary Figure 16: ^{13}C NMR spectrum of ((2,5-diiodo-1,4-phenylene)bis(oxy))dicyclohexane.

Synthesis of ((2,5-bis(cyclohexyloxy)-4-iodophenyl)ethynyl)trimethylsilane

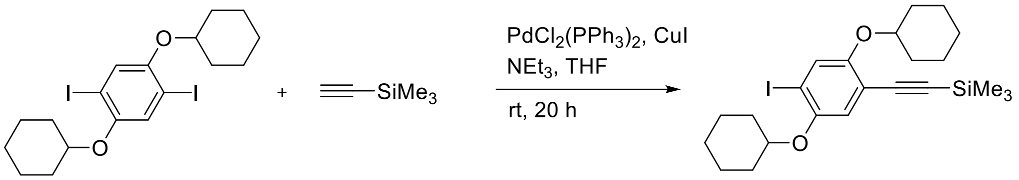

((2,5-Diiodo-1,4-phenylene)bis(oxy))dicyclohexane (3.50 g, 6.65 mmol, 1.00 eq.), 2.5 mol% bis(triphenylphosphine)palladium(II) dichloride (117 mg, 0.166 mmol) and 5 mol% copper(I) iodide (63.3 mg, 0.333 mmol) were placed into a Schlenk flask and degassed. Under continuous argon flow, 140 mL dry THF and 9.22 mL dry triethylamine were added, and the mixture was stirred for 10 minutes. Subsequently, 1.04 mL trimethylsilylacetylene (719 mg, 7.32 mmol, 1.10 eq.) with 5 mL dry THF were added dropwise with a syringe. The reaction mixture was stirred for 20 h at room temperature, taken up in dichloromethane and washed with saturated NH_4Cl solution. The aqueous phase was extracted three times with dichloromethane. The combined organic layers were dried over Na_2SO_4, filtered and concentrated under reduced pressure. The residue was purified by silica column chromatography (cyclohexane / dichloromethane 9:1) to yield the product as a yellow solid (1.40 g, 42%). TLC (cyclohexane / dichloromethane 9:1) R_f = 0.50; ^{1}H NMR ($CDCl_3$, 300 MHz): δ (ppm) = 7.31 (s, 1 H, 1 C**H**$_{\mathbf{aromatic}}$CI), 6.85 (s, 1 H, 1 C**H**$_{\mathbf{aromatic}}$C-C≡C), 4.30-4.11 (m, 2 H, 2 C**H**O), 2.05–1.74 (m, 8 H, 4 C**H**$_{\mathbf{equatorial}}$CHO, 4 C**H**$_{\mathbf{equatorial}}$$CH_2$CHO), 1.74–1.48 (m, 6 H, 4 C**H**$_{\mathbf{axial}}$CHO, 2 C**H**$_{\mathbf{equatorial}}$$CH_2CH_2$CHO), 1.43–1.18 (m, 6 H, 4 C**H**$_{\mathbf{axial}}$$CH_2$CHO, 2 C**H**$_{\mathbf{axial}}$$CH_2CH_2$CHO), 0.25 (s, 9 H, 3 C**H$_3$**Si); ^{13}C NMR ($CDCl_3$, 75 MHz): δ (ppm) = 153.71, 150.94, 128.10, 118.39, 115.61, 101.30, 98.97, 89.90, 77.90, 77.48, 31.56, 31.50, 25.70, 25.63, 23.27, 23.05, -0.03; FAB of $C_{23}H_{33}IO_2Si$ ($M+H^+$ = 497.2); HRMS (FAB) of $C_{23}H_{33}IO_2Si$ [$M+H^+$] calc. 496.1295, found 496.1294; IR (ATR) v = 2931.5, 2855.3, 2154.5, 1470.9, 1365.1, 1247.3, 1196.5, 1158.6, 1123.6, 1039.8, 1018.4, 960.9, 837.9, 757.7, 698.2, 671.6, 645.2, 481.4 cm^{-1}.

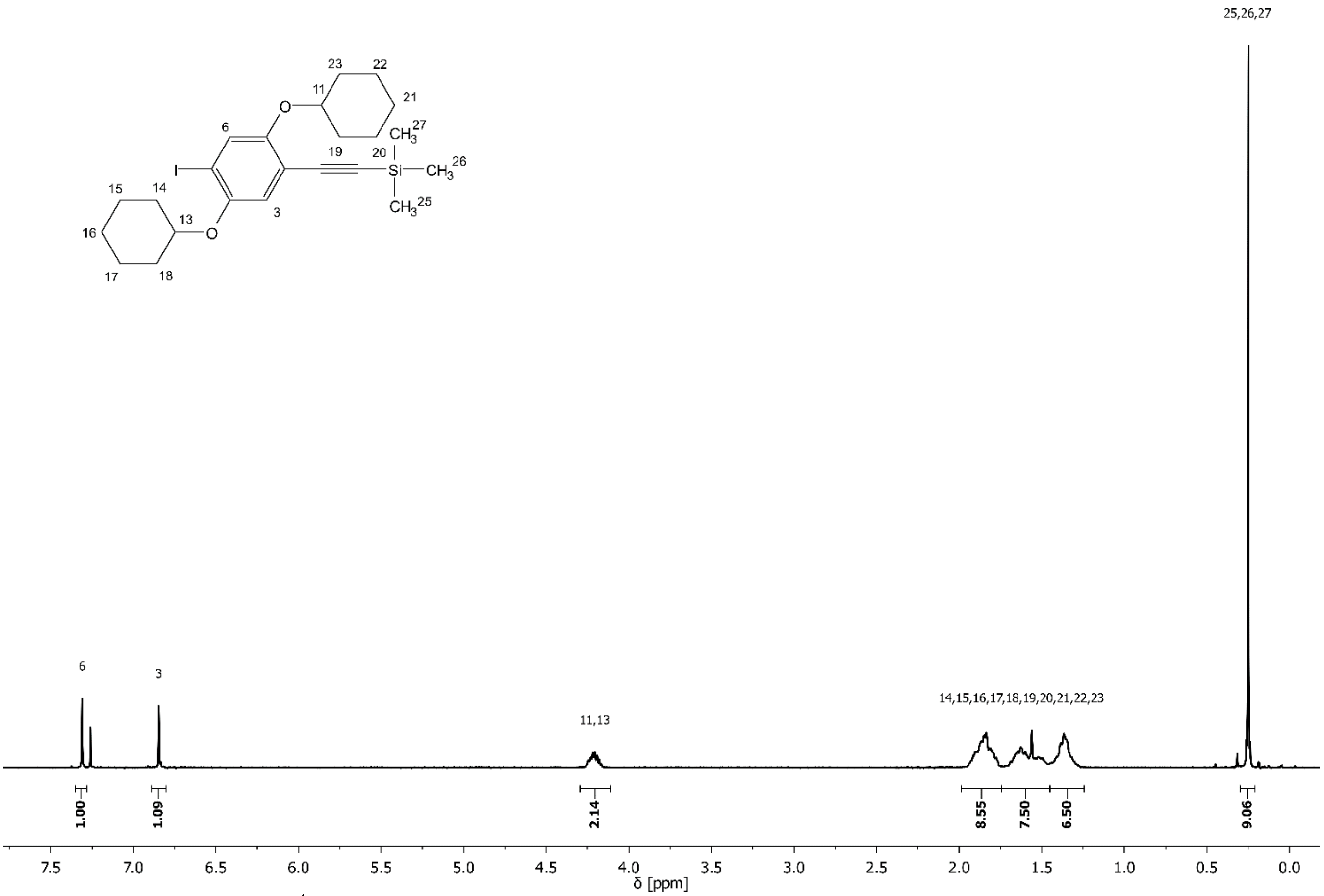

Supplementary Figure 17: ^{1}H NMR spectrum of the dicyclohexyloxy-building block with assigned signals.

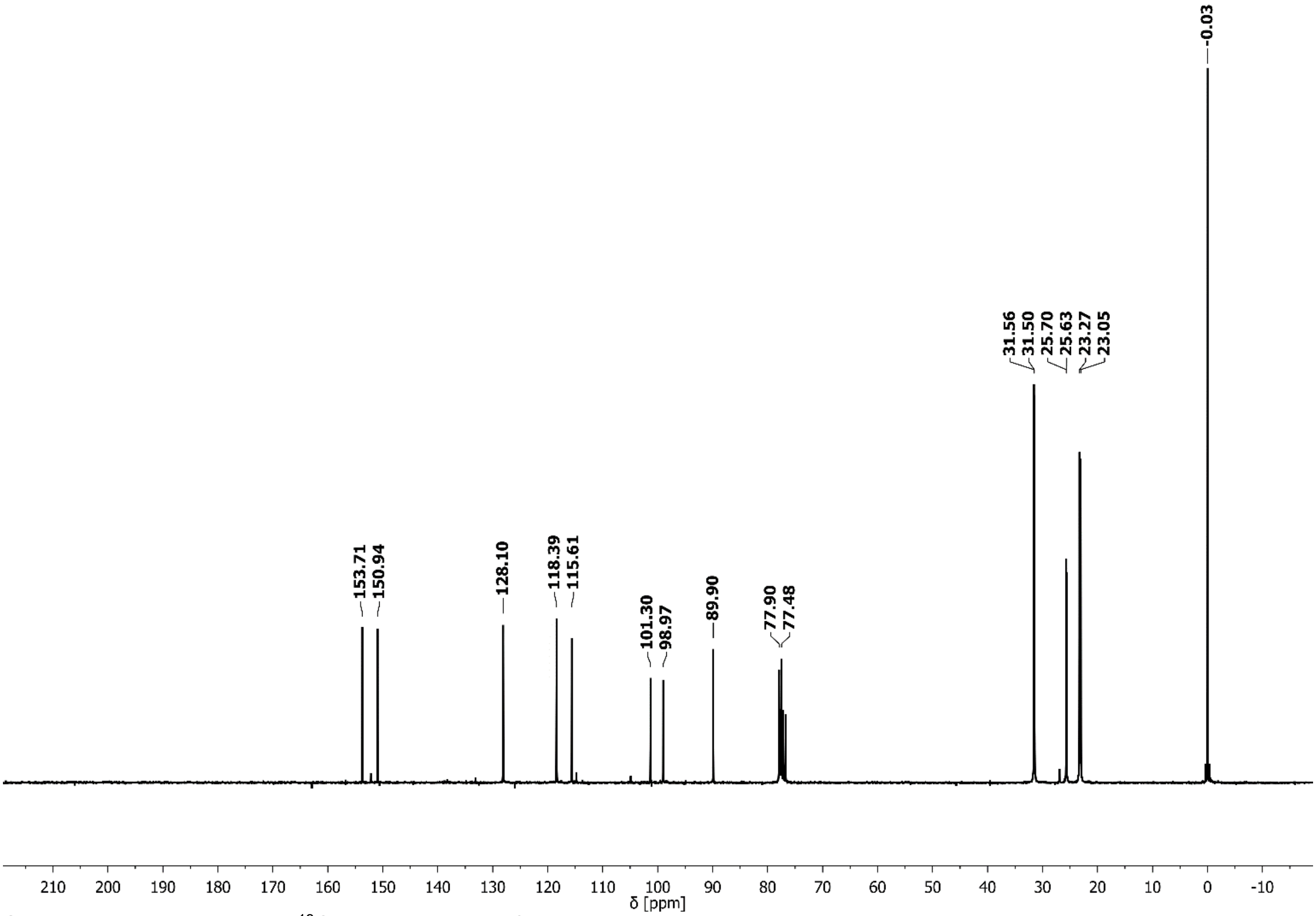

Supplementary Figure 18: ^{13}C NMR spectrum of the dicyclohexyloxy-building block.

Synthesis of 1,4-diiodo-2,5-dimethoxybenzene

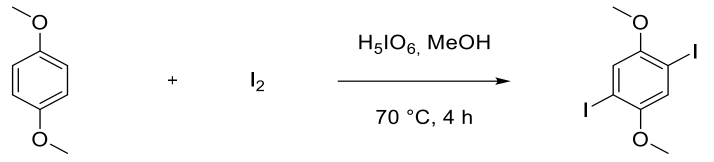

Periodic acid (3.20 g, 14.0 mmol, 0.636 eq.) was dissolved in 25 mL methanol and stirred for 10 minutes. Subsequently, iodine (6.97 g, 27.0 mmol, 1.23 eq.) was added and after an additional stirring time of 10 minutes 1,4-dimethoxybenzene (3.04 g, 22.0 mmol, 1.00 eq.) was added. The reaction mixture was stirred at 70 °C for 4 h. The residue was carefully poured into 50 mL water containing potassium disulfite. The precipitate was washed with methanol and dissolved in dichloromethane. The solution was filtered, and the filtrate was concentrated under reduced pressure. The residue was purified by recrystallisation from methanol to yield the product as a white solid (7.90 g, 92%). TLC (cyclohexane / dichloromethane 4:1) R_f = 0.48; ^{1}H NMR ($CDCl_3$, 300 MHz): δ (ppm) = 7.19 (s, 2 H, 2 C**H**$_{\mathbf{aromatic}}$), 3.83 (s, 6 H, 2 C**H**$_\mathbf{3}$); ^{13}C NMR ($CDCl_3$, 75 MHz): δ (ppm) = 153.47, 121.76, 85.61, 57.33; FAB of $C_8H_8I_2O_2$ (M+H$^+$ = 390.9); HRMS (FAB) of $C_8H_8I_2O_2$ [M+H$^+$] calc. 389.8608, found 389.8609; IR (ATR) v = 2927.6, 2829.6, 1681.0, 1480.0, 1443.7, 1432.1, 1345.3, 1270.9, 1200.3, 1057.4, 1014.2, 850.0, 836.7, 743.7, 615.8, 482.9, 431.8 cm^{-1}.

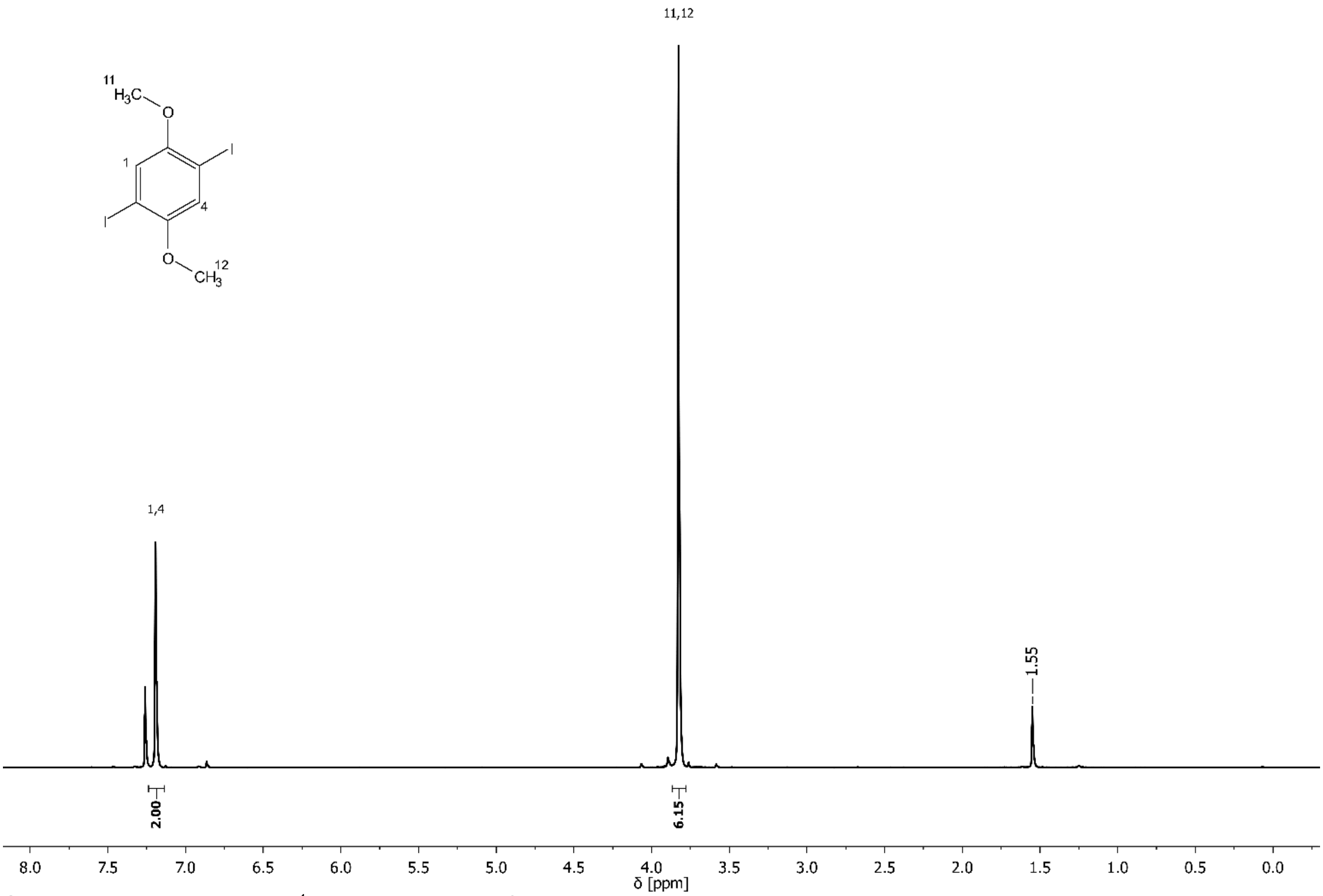

Supplementary Figure 19: ^{1}H NMR spectrum of 1,4-diodo-2,5-dimethoxybenzene with assigned signals.

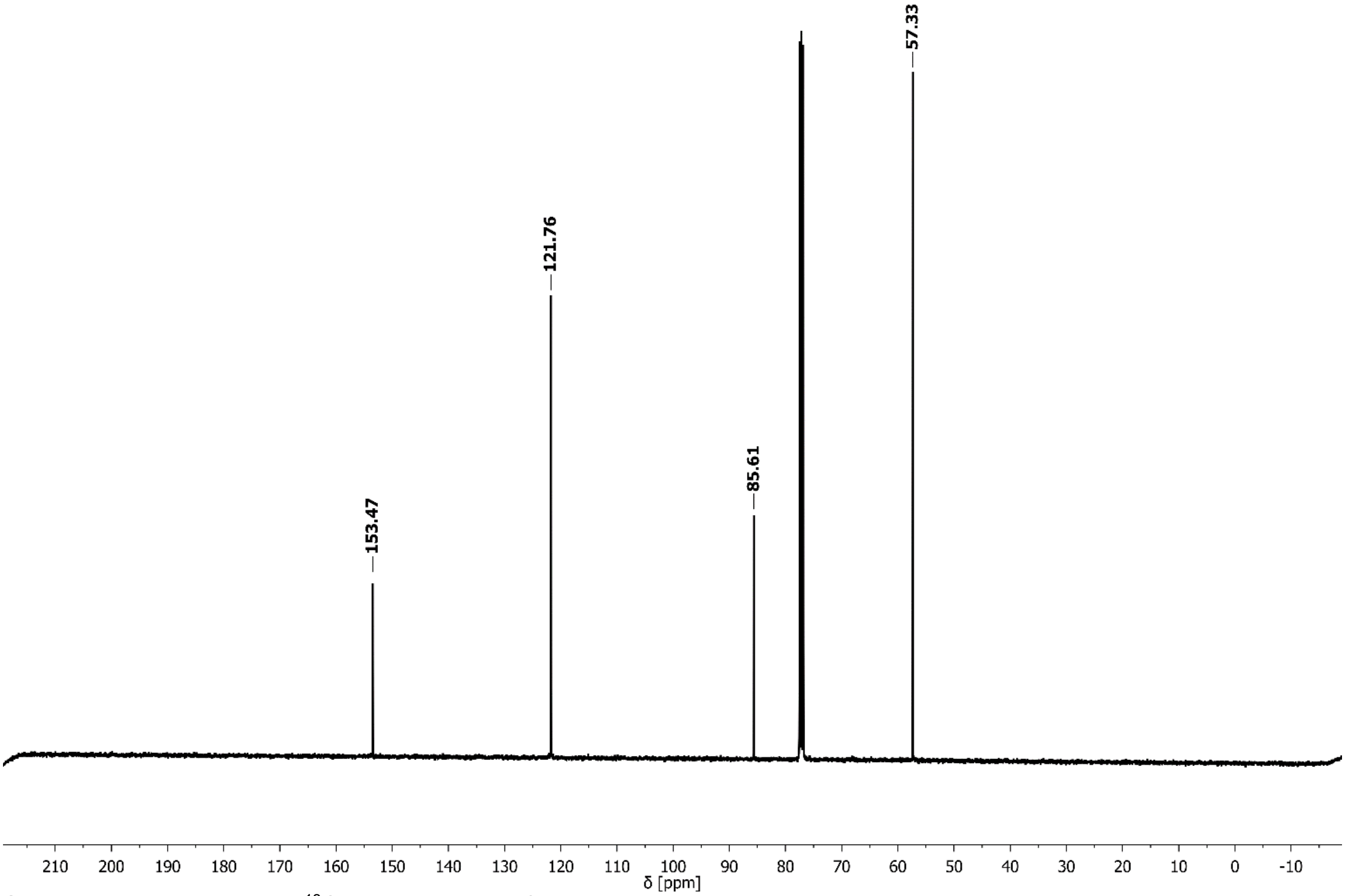

Supplementary Figure 20: ^{13}C NMR spectrum of 1,4-diodo-2,5-dimethoxybenzene.

Synthesis of ((4-iodo-2,5-dimethoxyphenyl)ethynyl)trimethylsilane

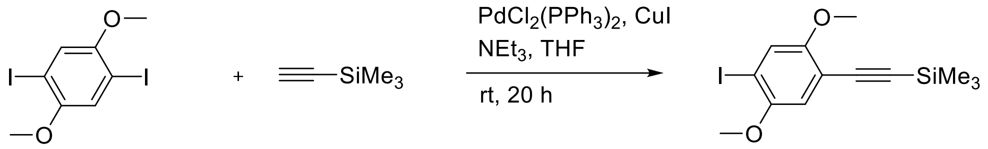

1,4-Diiodo-2,5-dimethoxybenzene (10.0 g, 25.6 mmol, 1.00 eq.), 2.5 mol% bis(triphenylphosphine)palladium(II) dichloride (450 mg, 0.641 mmol) and 5 mol% copper(I) iodide (244 mg, 1.28 mmol) were placed into a Schlenk flask and degassed. Under continuous argon flow, 400 mL dry THF and 35.5 mL dry triethylamine were added and the mixture was stirred for 10 minutes. Subsequently, 3.91 mL trimethylsilylacetylene (2.77 g, 28.2 mmol, 1.10 eq.) with 5 mL dry THF were added dropwise with a syringe. The reaction mixture was stirred for 20 h at room temperature, taken up in dichloromethane and washed with saturated NH_4Cl solution. The aqueous phase was extracted three times with dichloromethane. The combined organic layers were dried over Na_2SO_4, filtered and concentrated under reduced pressure. The residue was purified by silica column chromatography (cyclohexane / dichloromethane 9:1) to yield the product as a yellow solid (3.10 g, 34%). TLC (cyclohexane / dichloromethane 4:1) R_f = 0.42; ^{1}H NMR ($CDCl_3$, 300 MHz): δ (ppm) = 7.26 (s, 1 H, 1 C**H$_{aromatic}$**CI), 6.87 (s, 1 H, 1 C**H$_{aromatic}$**C-C≡C), 3.83 (s, 6 H, 2 C**H$_3$**), 0.27 (s, 9 H, 3 C**H$_3$**Si); ^{13}C NMR ($CDCl_3$, 75 MHz): δ (ppm) = 155.18, 152.29, 122.53, 115.65, 113.06, 100.65, 99.93, 87.17, 57.09, 56.82, 0.13; FAB of $C_{13}H_{17}IO_2Si$ (M+H$^+$ = 361.3); HRMS (FAB) of $C_{13}H_{17}IO_2Si$ [M+H$^+$] calc. 360.0037, found 360.0038; IR (ATR) v = 2955.2, 2839.8, 2149.5, 1489.8, 1437.0, 1370.9, 1279.2, 1247.0, 1214.5, 1186.9, 1157.2, 1039.1, 954.1, 836.0, 796.4, 756.0, 729.7, 696.4, 663.6, 639.4, 484.6 cm^{-1}.

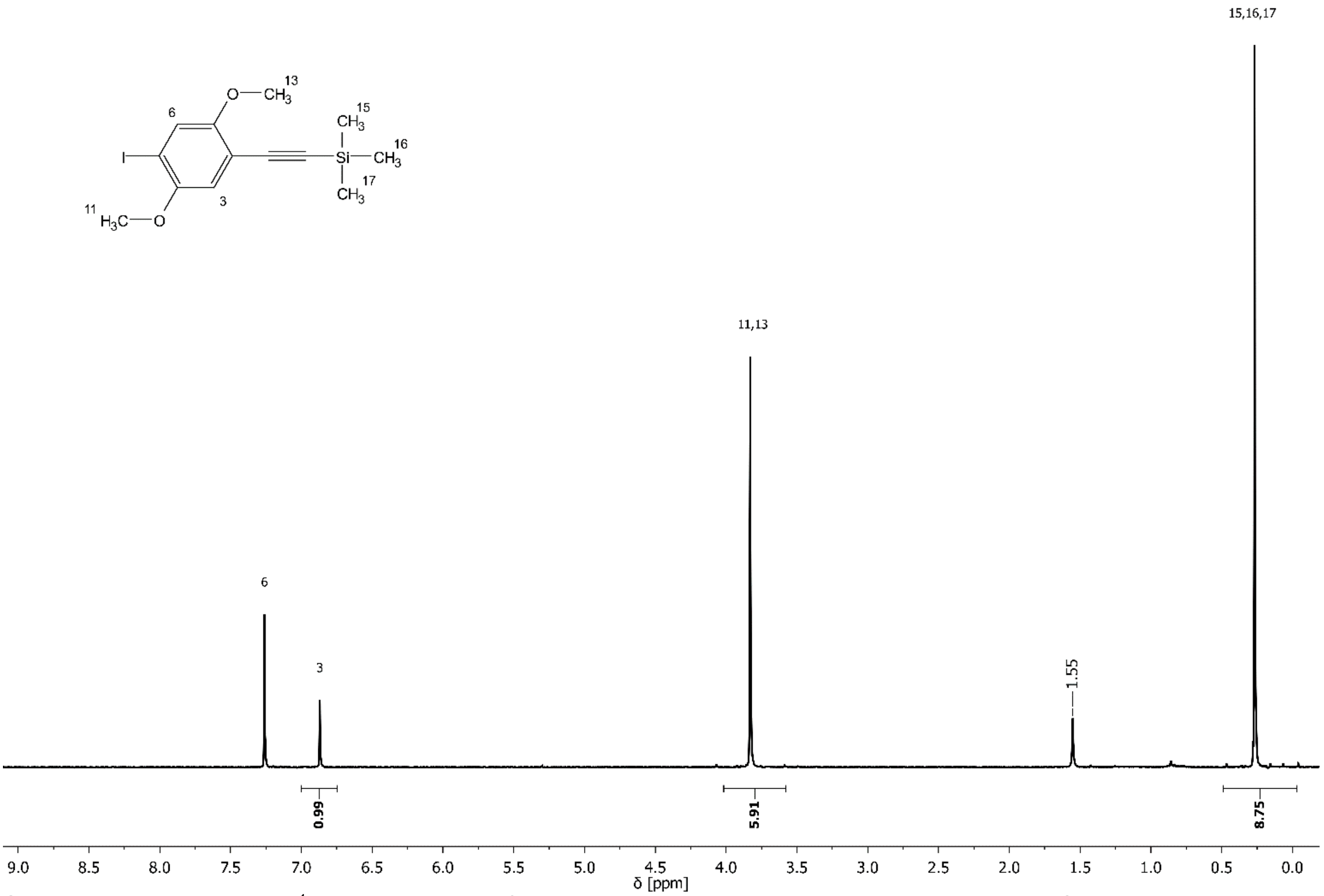

Supplementary Figure 21: ^{1}H NMR spectrum of the dimethoxy-building block with assigned signals. Signal 6 overlays with $CDCl_3$.

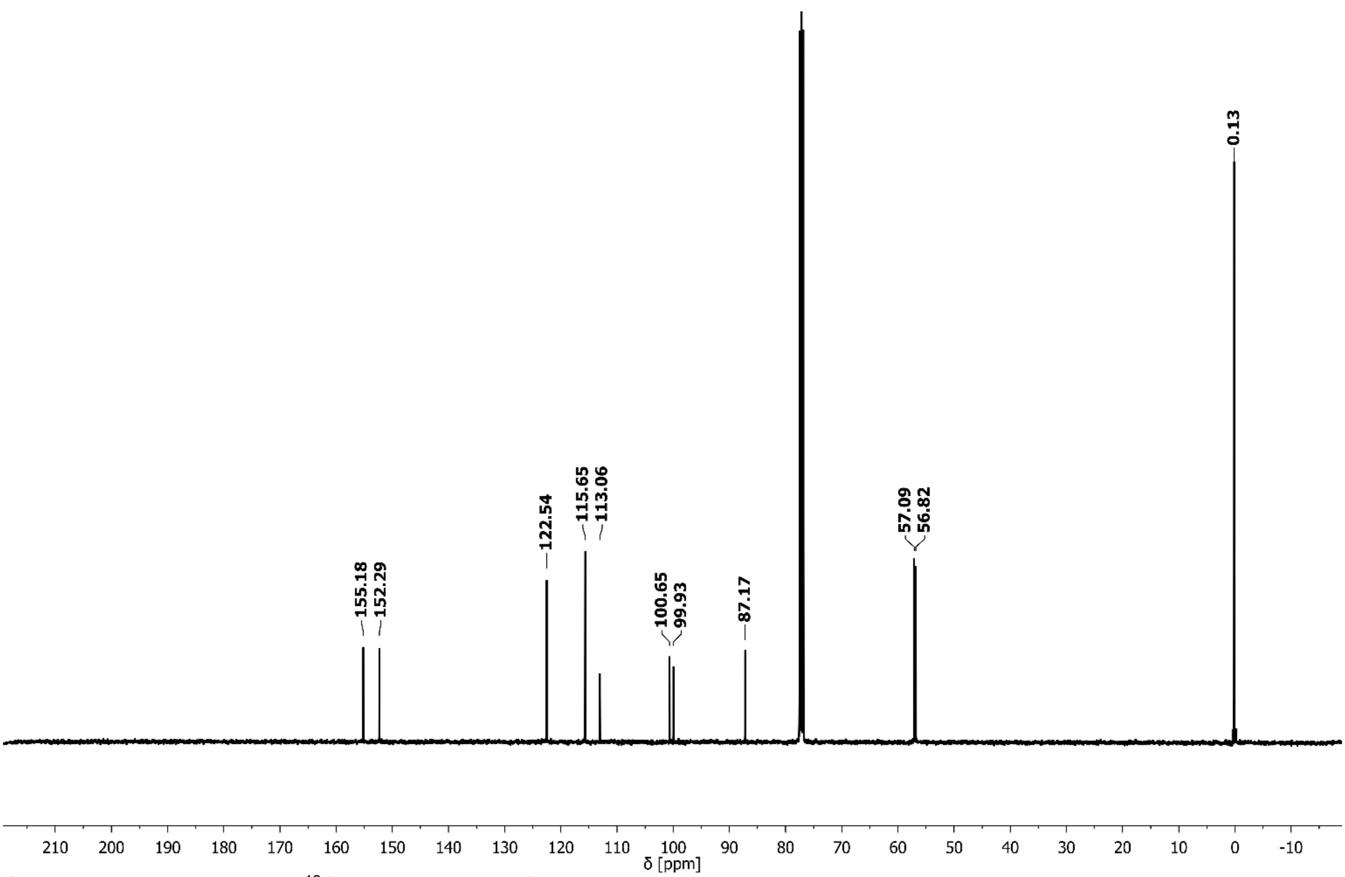

Supplementary Figure 22: ^{13}C NMR spectrum of the dimethoxy-building block.

Synthesis of 1,4-bis(octyloxy)benzene

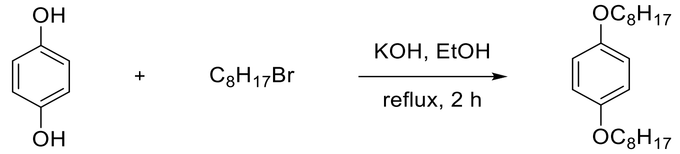

Hydroquinone (30.0 g, 272 mmol, 1.00 eq.) was dissolved in 250 mL absolute ethanol. Potassium hydroxide (38.2 g, 681 mmol, 2.50 eq.) was added and the mixture was stirred for 30 minutes under reflux. Subsequently, 1-bromooctane (104 mL, 116 g, 599 mmol, 2.20 eq.) was slowly added over a 1 h time period and stirred under reflux for another 2 h. Ethanol was removed with a rotary evaporator and the residue was taken up in dichloromethane. The organic phase was washed with water three times and once more with saturated $NaHCO_3$ solution. It was then dried over Na_2SO_4, filtered and the solvent was removed under reduced pressure and the crude product was recrystallised from methanol to yield colorless crystals (37.6 g, 41%). TLC (cyclohexane / dichloromethane 9:1) R_f = 0.54; ^{1}H NMR ($CDCl_3$, 300 MHz): δ (ppm) = 6.82 (d, J = 0.9 Hz, 4 H, 4 C**H$_{aromatic}$**), 3.90 (t, J = 6.6 Hz, 4 H, 2 C**H$_2$**O), 1.75 (p, J = 6.7 Hz, 4 H, 2 C**H$_2$**CH_2O), 1.51–1.18 (m, 20 H, 10 C**H$_2$**), 0.89 (t, J = 6.2 Hz, 6 H, 2 C**H$_3$**); ^{13}C NMR ($CDCl_3$, 75 MHz): δ (ppm) = 153.35, 115.53, 68.81, 31.97, 29.54, 29.40, 26.22, 22.81, 14.25; FAB of $C_{22}H_{38}O_2$ (M+H$^+$ = 335.3); HRMS (FAB) of $C_{22}H_{38}O_2$ [M+H$^+$] calc. 334.2866, found 334.2866; IR (ATR) v = 2954.3, 2920.0, 2870.3, 2852.9, 2022.5, 1507.1, 1472.7, 1463.7, 1416.8, 1393.5, 1288.1, 1223.7, 1114.7, 1043.6, 1028.2, 998.6, 942.5, 826.3, 770.1, 720.5, 534.9, 521.8, 507.2, 386.4 cm^{-1}.

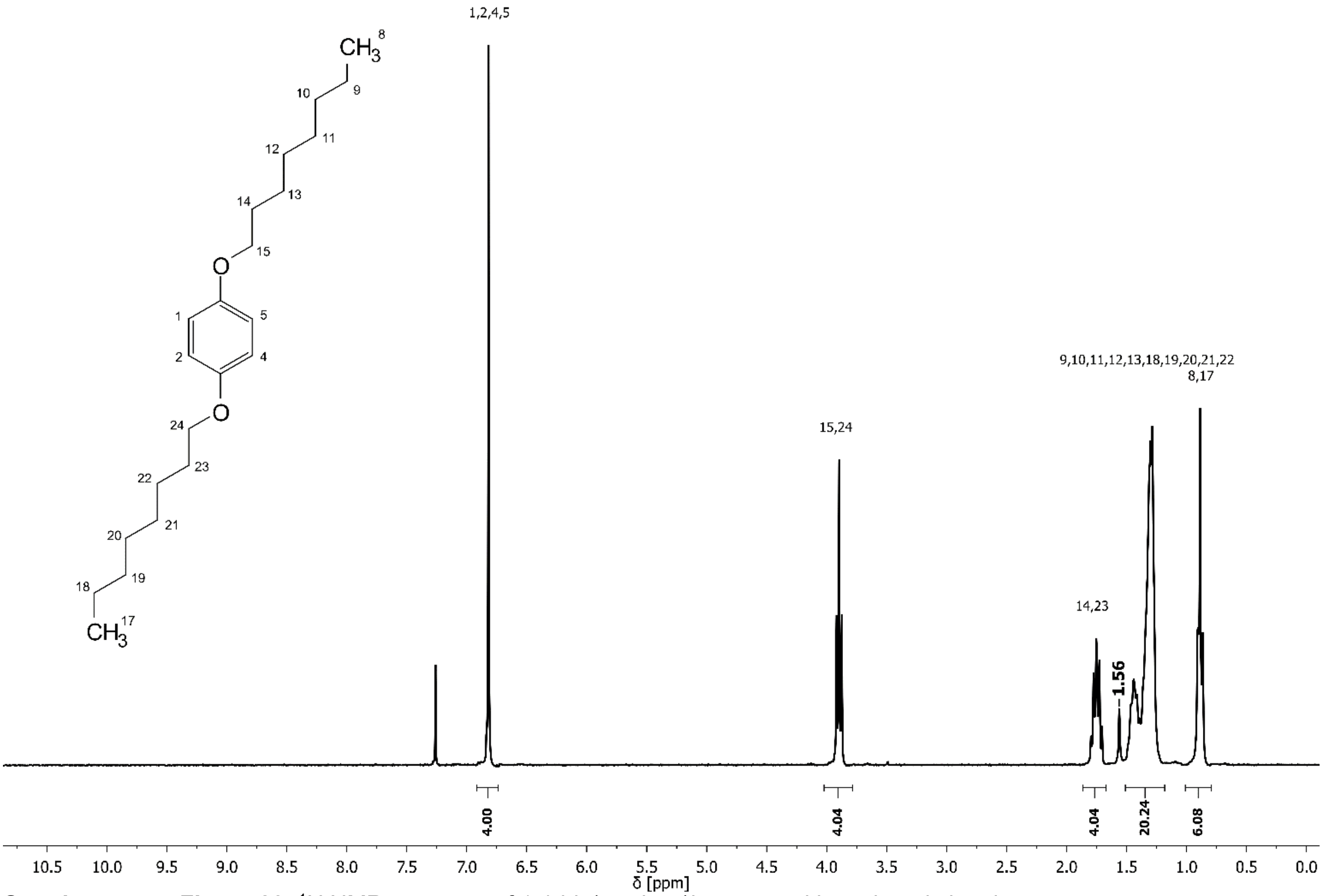

Supplementary Figure 23: ^{1}H NMR spectrum of 1,4-bis(octyloxy)benzene with assigned signals.

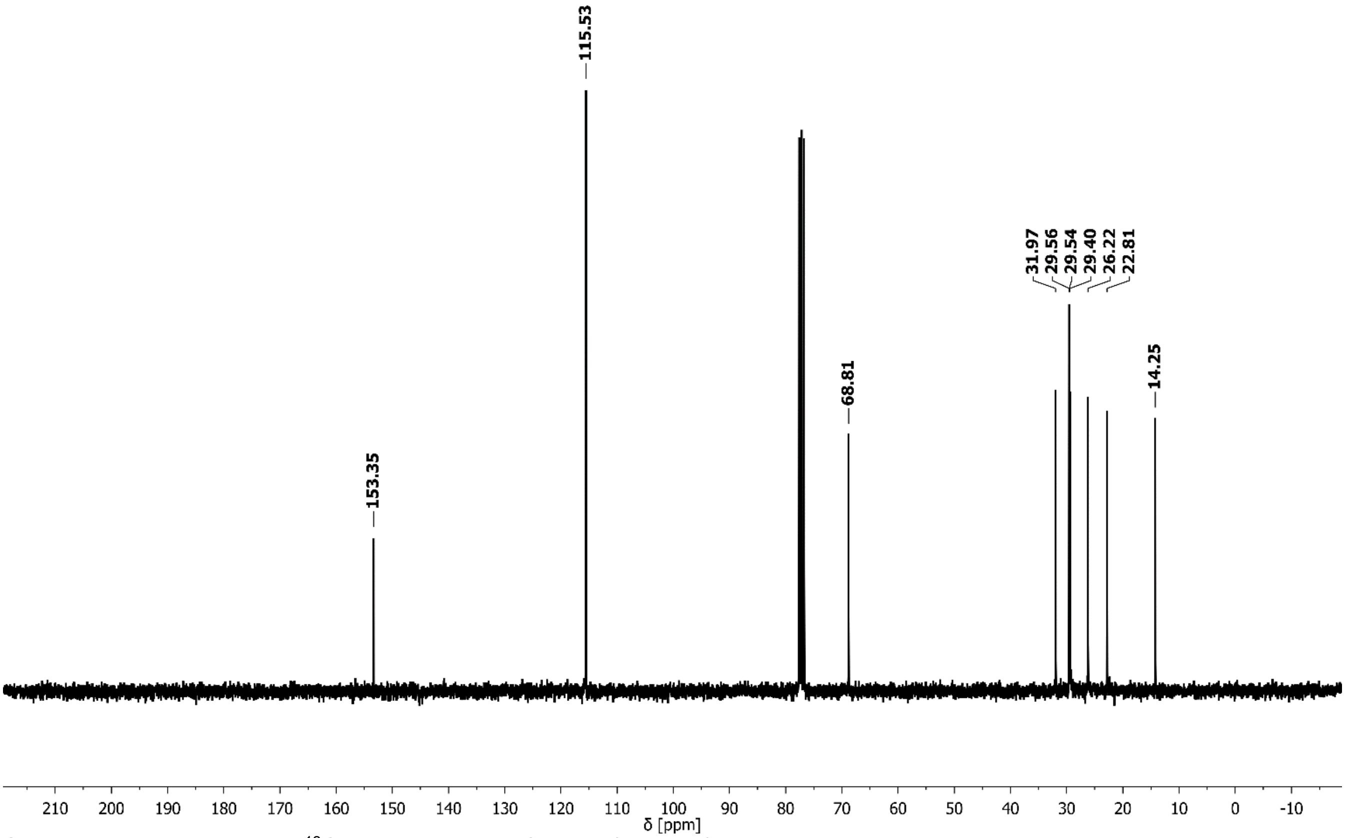

Supplementary Figure 24: ^{13}C NMR spectrum of 1,4-bis(octyloxy)benzene.

Synthesis of 1,4-diiodo-2,5-bis(octyloxy)benzene

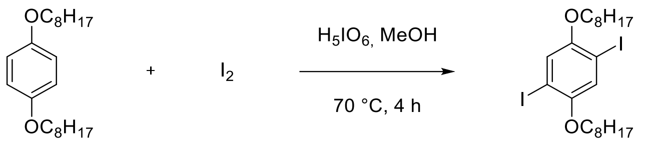

Periodic acid (16.0 g, 70.3 mmol, 0.636 eq.) was dissolved in 150 mL methanol and stirred for 10 minutes. Subsequently, iodine (34.5 g, 136 mmol, 1.23 eq.) was added and after an additional stirring time of 10 minutes, 1,4-bis(octyloxy)benzene (37.0 g, 111 mmol, 1.00 eq.) was added. The reaction mixture was stirred at 70 °C for 4 h. The residue was carefully poured into 300 mL water containing potassium disulfite. The precipitate was washed with methanol and dissolved in dichloromethane. The solution was filtered, and the filtrate was concentrated under reduced pressure. The residue was purified by recrystallisation from methanol to yield the product as a white solid (58.5 g, 90%). TLC (cyclohexane / dichloromethane 9:1) R_f = 0.72; ^{1}H NMR ($CDCl_3$, 300 MHz): δ (ppm) = 7.17 (s, 2 H, 2 C**H**$_{\mathbf{aromatic}}$), 3.92 (t, J = 6.4 Hz, 4 H, 2 C$\mathbf{H_2}$O), 1.90–1.70 (m, 4 H, 2 C**H**$_2$CH$_2$O), 1.66–1.17 (m, 20 H, 5 C$\mathbf{H_2}$), 0.89 (t, J = 6.7 Hz, 6 H, 2 C$\mathbf{H_3}$); ^{13}C NMR ($CDCl_3$, 75 MHz): δ (ppm) = 152.95, 122.87, 86.43, 70.46, 31.95, 29.39, 29.37, 29.28, 26.17, 22.82, 14.28; FAB of $C_{22}H_{36}I_2O_2$ ($M+H^+$ = 587.3); HRMS (FAB) of $C_{22}H_{36}I_2O_2$ [$M+H^+$] calc. 586.0799, found 586.0801; IR (ATR) ν = 2915.4, 2847.8, 1484.8, 1458.4, 1388.1, 1350.8, 1263.0, 1212.9, 1143.5, 1066.9, 1049.7, 1012.8, 961.6, 900.4, 845.7, 834.3, 785.7, 747.0, 720.1, 533.3, 435.1 cm^{-1}.

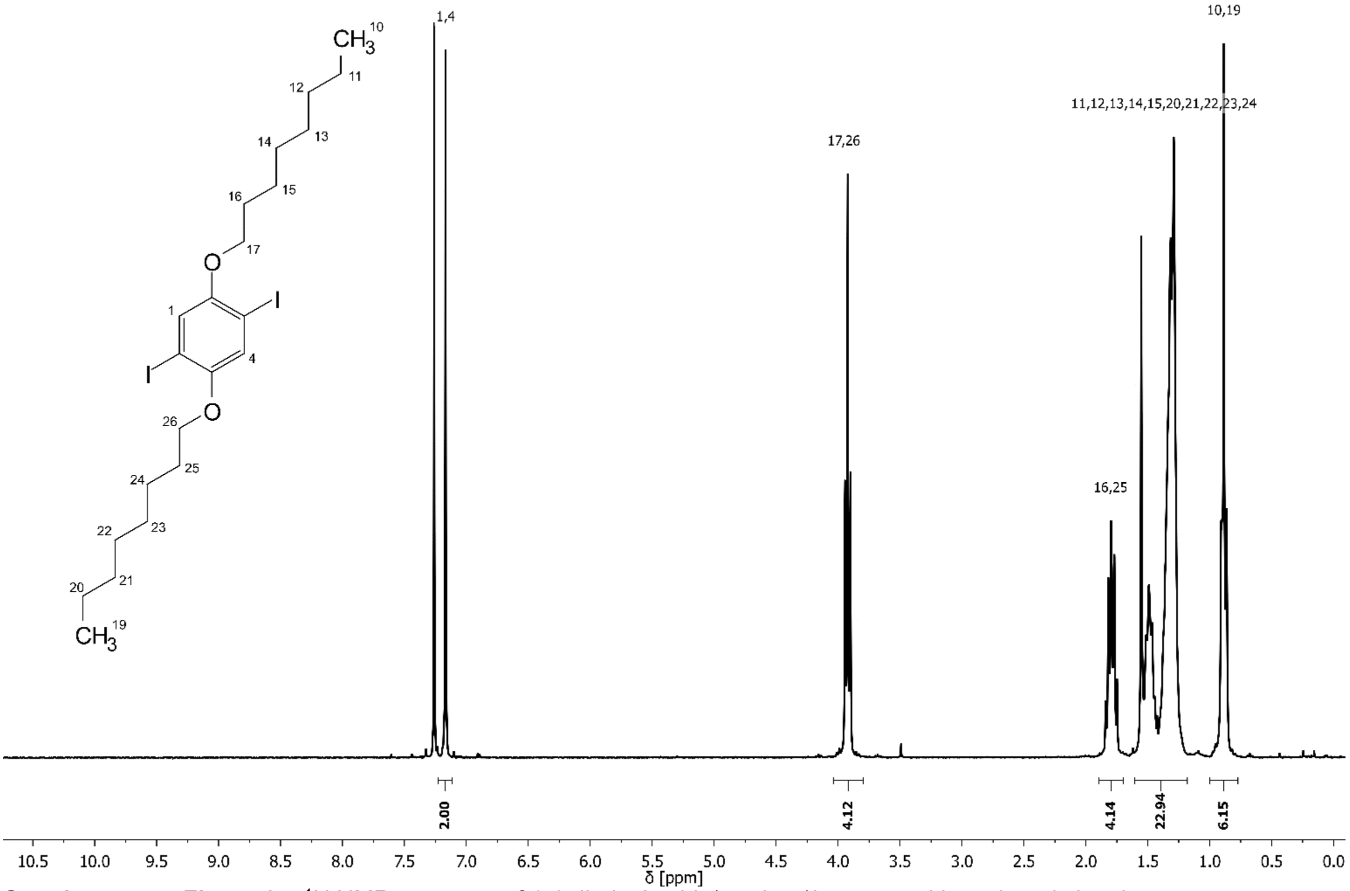

Supplementary Figure 25: ^{1}H NMR spectrum of 1,4-diodo-2,5-bis(octyloxy)benzene with assigned signals.

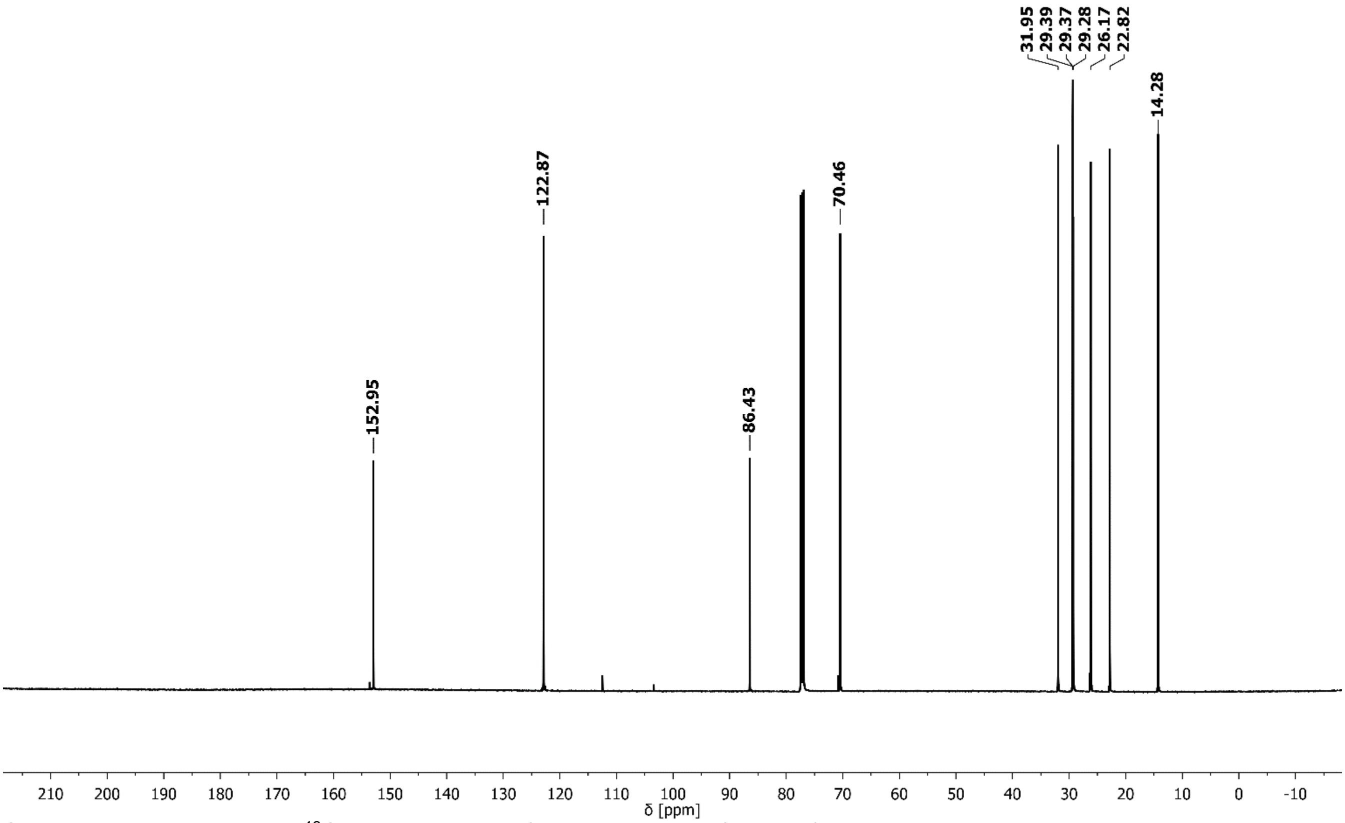

Supplementary Figure 26: ^{13}C NMR spectrum of 1,4-diodo-2,5-bis(octyloxy)benzene.

Synthesis of ((4-iodo-2,5-bis(octyloxy)phenyl)ethynyl)trimethylsilane

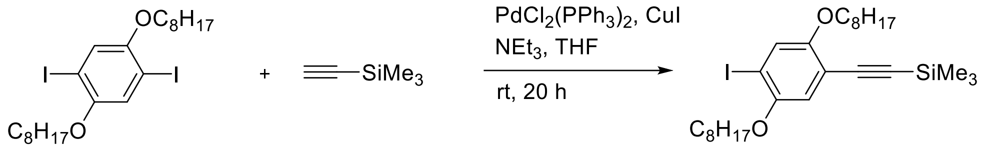

1,4-Diiodo-2,5-bis(octyloxy)benzene (10.0 g, 17.1 mmol, 1.00 eq.), 2.5 mol% bis(triphenylphosphine)palladium(II) dichloride (299 mg, 0.426 mmol) and 5 mol% copper(I) iodide (162 mg, 0.853 mmol) were placed into a Schlenk flask and degassed. Under continuous argon flow, 400 mL dry THF and 23.6 mL dry triethylamine were added, and the mixture was stirred for 10 minutes. Subsequently, 2.67 mL trimethylsilylacetylene (1.84 g, 18.8 mmol, 1.10 eq.) with 5 mL dry THF was added dropwise with a syringe. The reaction mixture was stirred for 20 h at room temperature, taken up in dichloromethane and washed with saturated NH_4Cl solution. The aqueous phase was extracted three times with dichloromethane. The combined organic layers were dried over Na_2SO_4, filtered and concentrated under reduced pressure. The residue was purified by silica column chromatography (cyclohexane / dichloromethane 9:1 and cyclohexane / ethyl acetate 20:1) to yield the product as a yellow liquid (3.17 g, 33%). TLC (cyclohexane / dichloromethane 9:1) R_f = 0.62; ^{1}H NMR ($CDCl_3$, 300 MHz): δ (ppm) = 7.25 (s, 1 H, 1 C**H**$_{\mathbf{aromatic}}$CI), 6.83 (s, 1 H, 1 C**H**$_{\mathbf{aromatic}}$C-C≡C), 3.93 (t, J = 6.3 Hz, 4 H, 2 C**H**$_\mathbf{2}$O), 1.89–1.70 (m, 4 H, 2 C**H**$_\mathbf{2}$CH$_3$), 1.54–1.41 (m, 4 H, 2 C**H**$_2$CH$_2$CH$_3$), 1.41–1.19 (m, 16 H, 8 C**H**$_\mathbf{2}$), 0.88 (t, J = 6.5 Hz, 6 H, 2 C**H**$_\mathbf{3}$), 0.25 (s, 9 H, 3 C**H**$_\mathbf{3}$Si); ^{13}C NMR ($CDCl_3$, 75 MHz): δ (ppm) = 155.01, 151.82, 123.94, 116.42, 113.53, 100.92, 99.55, 88.04, 70.21, 69.90, 31.98, 31.95, 29.52, 29.47, 29.43, 29.40, 29.37, 29.31, 27.05, 26.20, 26.14, 22.81, 14.26, 14.24, 0.08; FAB of $C_{27}H_{45}IO_2Si$ (M+H$^+$ = 557.3); HRMS (FAB) of $C_{27}H_{45}IO_2Si$ [M+H$^+$] calc. 556.2234, found 556.2233; IR (ATR) v = 2922.5, 2853.9, 2155.3, 1578.1, 1484.0, 1463.2, 1369.7, 1247.9, 1214.0, 1159.2, 1030.7, 841.1, 758.6, 721.9, 698.7, 664.2, 636.2 cm^{-1}.

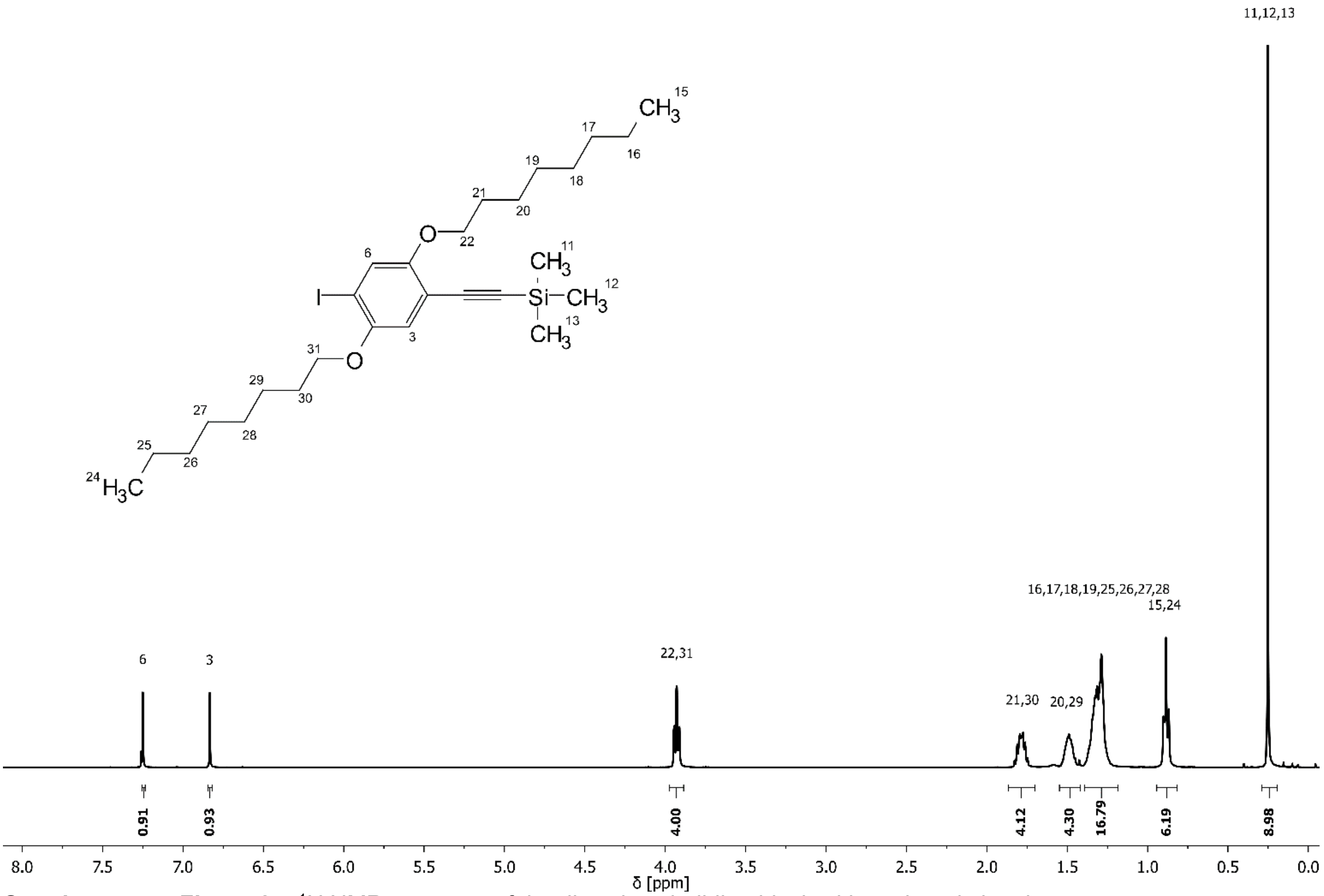

Supplementary Figure 27: ^{1}H NMR spectrum of the dioctyloxy-building block with assigned signals.

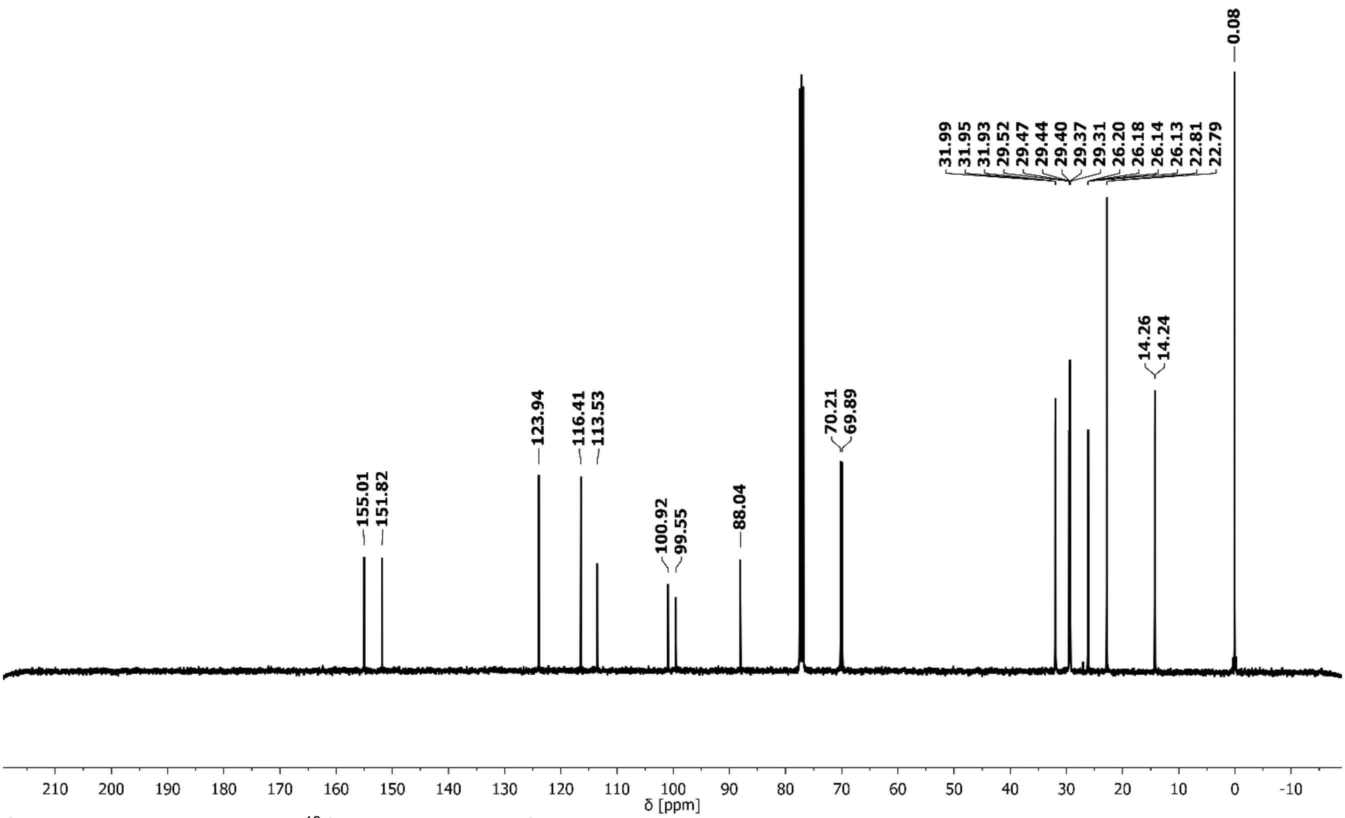

Supplementary Figure 28: ^{13}C NMR spectrum of the dioctyloxy-building block.

Synthesis of ((7-iodo-*9H*-fluoren-2-yl)ethynyl)trimethylsilane **22**

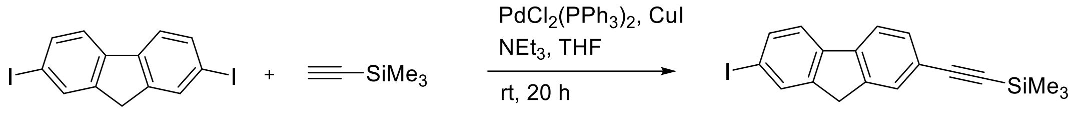

22

2,7-Diiodo-9*H*-fluorene (5.00 g, 12.0 mmol, 1.00 eq.), 2.5 mol% bis(triphenylphosphine)palladium(II) dichloride (212 mg, 0.302 mmol) and 5 mol% copper(I)iodide (114 mg, 0.599 mmol) were placed into a Schlenk flask and degassed. Under continuous argon flow, 200 mL dry THF and 16.6 mL dry triethylamine were added, and the mixture was stirred for 10 minutes. Subsequently, 1.00 mL trimethylsilylacetylene (736 mg, 7.23 mmol, 0.603 eq) was added dropwise with a syringe. The reaction mixture was stirred for 20 h at room temperature, taken up in dichloromethane and washed with saturated NH_4Cl solution. The aqueous phase was extracted three times with dichloromethane. The combined organic layers were dried over Na_2SO_4, filtered and concentrated under reduced pressure. The residue was purified by silica column chromatography (cyclohexane / dichloromethane 30:1) to yield the product as a white solid (1.20 g, 43%). TLC (cyclohexane / dichloromethane 9:1) R_f = 0.58; ^{1}H NMR (300 MHz, $CDCl_3$): δ (ppm) = 7.88 (s, 1 H, 1 CC$\mathbf{H}_{\mathbf{aromatic}}$CI), 7.72–7.64 (m, 3 H, 1 CHC$\mathbf{H}_{\mathbf{aromatic}}$CI, 2 C$\mathbf{H}_{\mathbf{aromatic}}$C-C≡C), 7.52–7.49 (m, 2 H, 2 C$\mathbf{H}_{\mathbf{aromatic}}$), 3.84 (s, 2 H, CC$\mathbf{H_2}$C), 0.27 (s, 9 H, 3 C$\mathbf{H_3}$Si); ^{13}C NMR (75 MHz, $CDCl_3$): δ (ppm) = 145.82, 142.56, 141.12, 140.70, 136.01, 134.30, 131.11, 128.59, 121.88, 121.85, 119.87, 105.73, 94.62, 92.56, 36.48, 0.18; HRMS (FAB) of $C_{18}H_{17}ISi[M+H^+]$ calc. 388.0144, found 388.0144; IR (ATR): v = 2955.9, 2897.5, 2181.0, 2150.9, 1595.1, 1556.4, 1449.7, 1400.7, 1325.6, 1276.1, 1242.9, 1166.5, 1135.7, 1043.0, 1002.0, 945.5, 928.0, 838.1, 808.8, 757.1, 731.4, 694.7, 646.6, 595.1, 521.6, 488.0, 470.4, 447.6, 409.4 cm^{-1}.

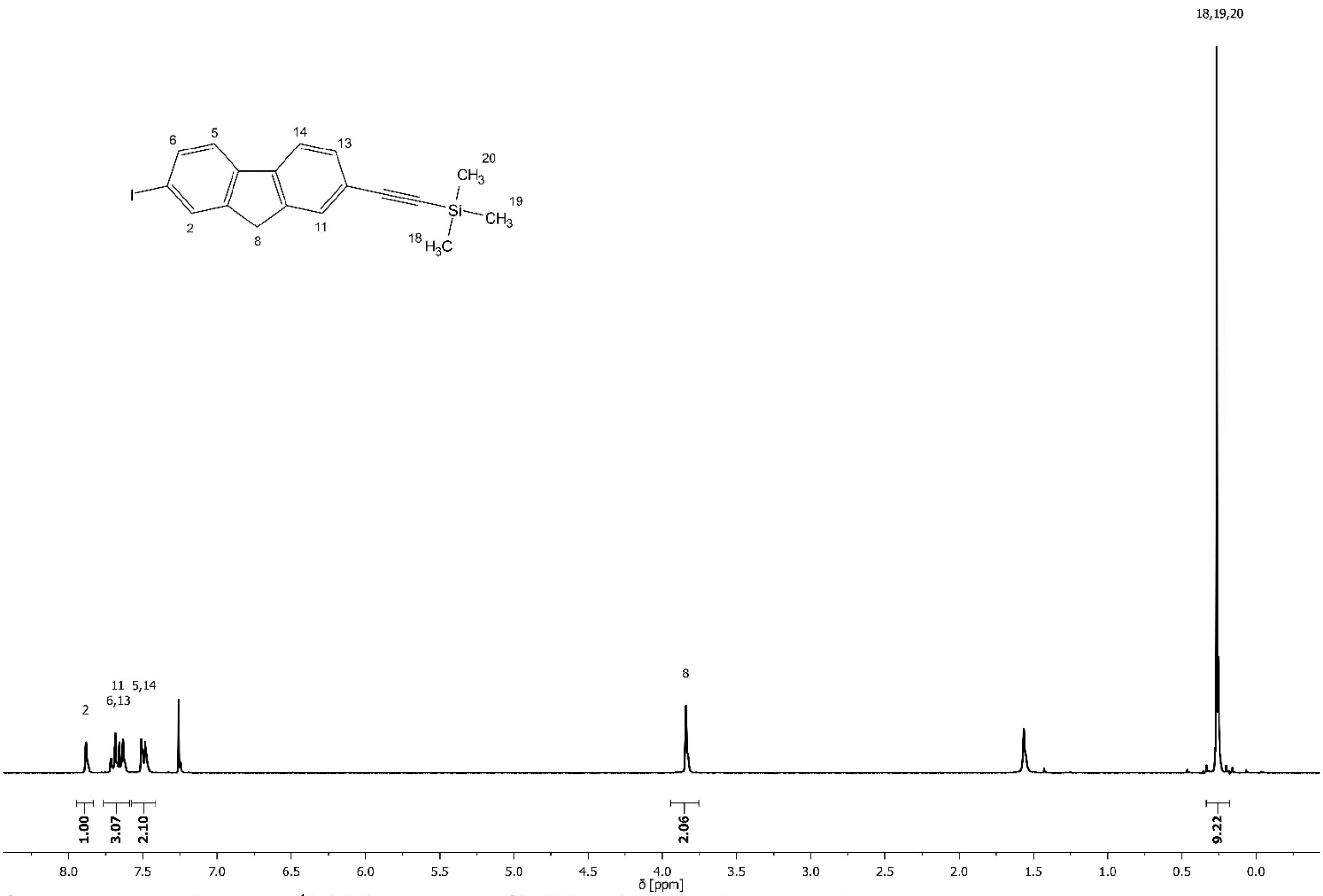

Supplementary Figure 29: ^1H NMR spectrum of building block **22** with assigned signals.

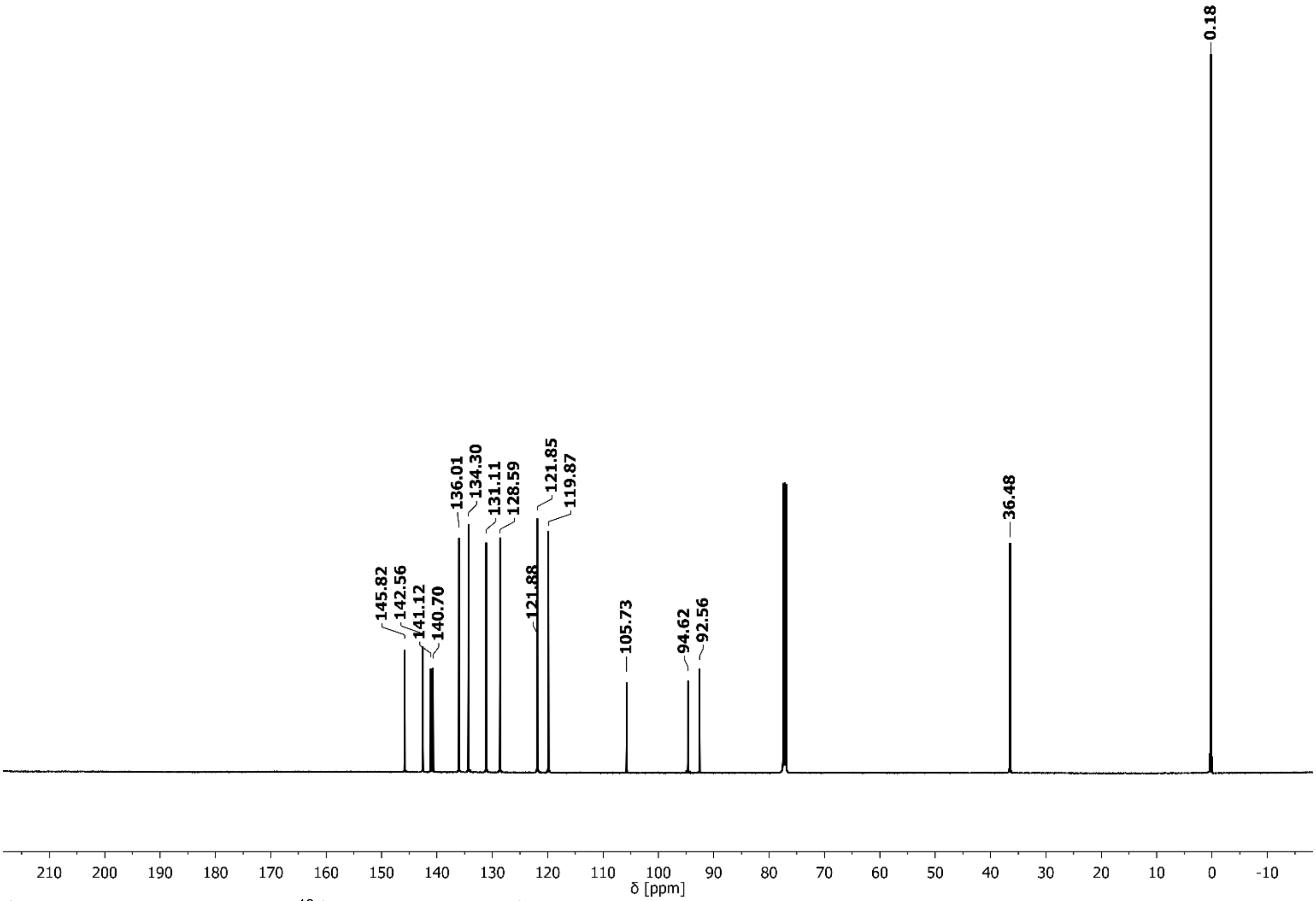

Supplementary Figure 30: ^{13}C NMR spectrum of building block **22**.

1.4 Synthesis of the Monodisperse Pentamer

Synthesis of trimethyl((4-(phenylethynyl)-2,5-dipropoxyphenyl)ethynyl)silane **2**

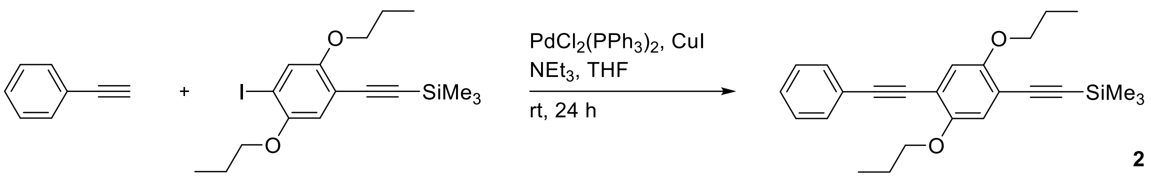

2

1,4-Bis(propyloxy)-2-iodo-5-trimethylsilylacetylenebenzene **1** (5.00 g, 12.0 mmol, 1.00 eq.), 2.5 mol% bis(triphenylphosphine) palladium(II) dichloride (210 mg, 0.300 mmol) and 5 mol% copper(I) iodide (114 mg, 0.600 mmol) were placed into a Schlenk flask and degassed three times. Under continuous argon flow, 150 mL dry THF and 16.6 mL dry triethylamine (12.2 g, 120 mmol, 10.0 eq.) were added and the mixture was stirred for 10 minutes. Subsequently, 3.96 mL phenylacetylene (3.68 g, 36.0 mmol, 3.00 eq.) in 5 mL THF were added dropwise with a syringe. The reaction mixture was stirred for 48 h at room temperature, taken up in dichloromethane and washed with saturated NH_4Cl solution. The aqueous phase was extracted three times with dichloromethane. The combined organic layers were dried over Na_2SO_4, filtered and concentrated under reduced pressure. The residue was purified by silica column chromatography twice (cyclohexane / dichloromethane 4:1 and cyclohexane / ethyl acetate 20:1) to yield the product as a yellow solid (4.63 g, 99%). TLC (cyclohexane / dichloromethane 4:1) R_f = 0.31; ^{1}H NMR ($CDCl_3$, 300 MHz): δ (ppm) = 7.58-7.46 (m, 2 H, 2 C**H$_{aromatic}$**C-C≡C), 7.42-7.28 (m, 3 H, 3 C**H$_{aromatic}$**), 6.97, 6.95 (2 s, 2 H, 2 C**H$_{aromatic}$**CO), 3.96 (dt, J = 6.4, 4.0 Hz, 4 H, 2 C**H$_2$**O), 1.96-1.71 (m, 4 H, 2 C**H$_2$**CH_3),), 1.09 (dt, J = 7.4, 2.7 Hz, 6 H, 2 C**H$_3$**), 0.26 (s, 9 H, 3 C**H$_3$**Si); ^{13}C NMR ($CDCl_3$, 75 MHz): δ (ppm) = 154.33, 153.63, 131.67, 128.44, 128.38, 123.58, 117.50, 117.21, 114.48, 113.93, 101.30, 100.15, 94.97, 85.98, 71.22, 71.18, 22.84, 10.67, 10.63, 0.08; FAB of $C_{25}H_{30}O_2Si$ (M+H$^+$ = 391.2); HRMS (FAB) of $C_{25}H_{30}O_2Si$ [M+H$^+$] calc. 390.2010, found 390.2011; IR (ATR) v = 2958.6, 2875.0, 2153.4, 1503.5, 1468.9, 1409.2, 1389.4, 1273.6, 1245.6, 1214.7, 1041.6, 1021.1, 889.1, 836.8, 752.4, 687.3, 627.0, 547.5, 526.5, 471.8, 384.5 cm^{-1}.

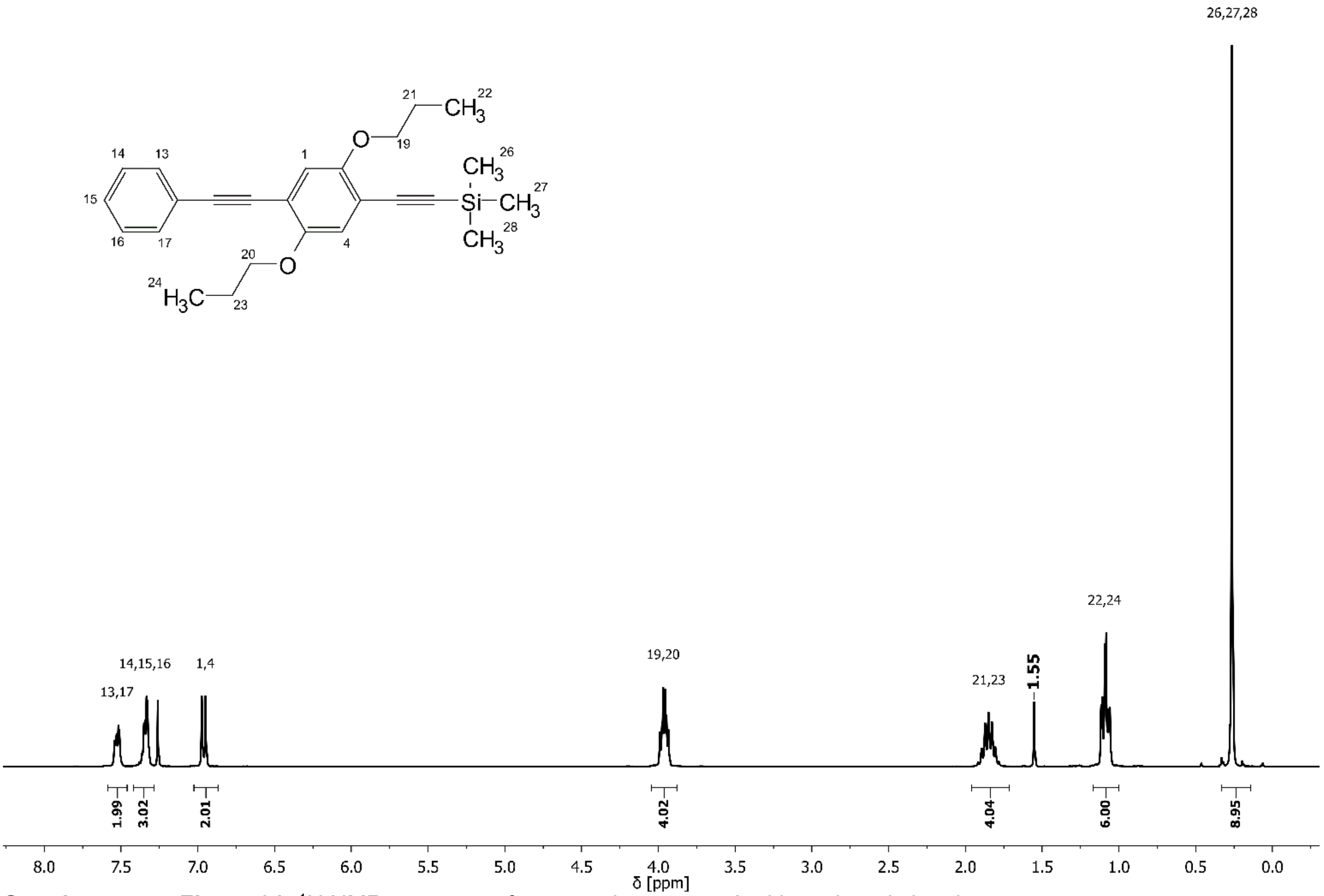

Supplementary Figure 31: ^{1}H NMR spectrum of protected monomer **2** with assigned signals.

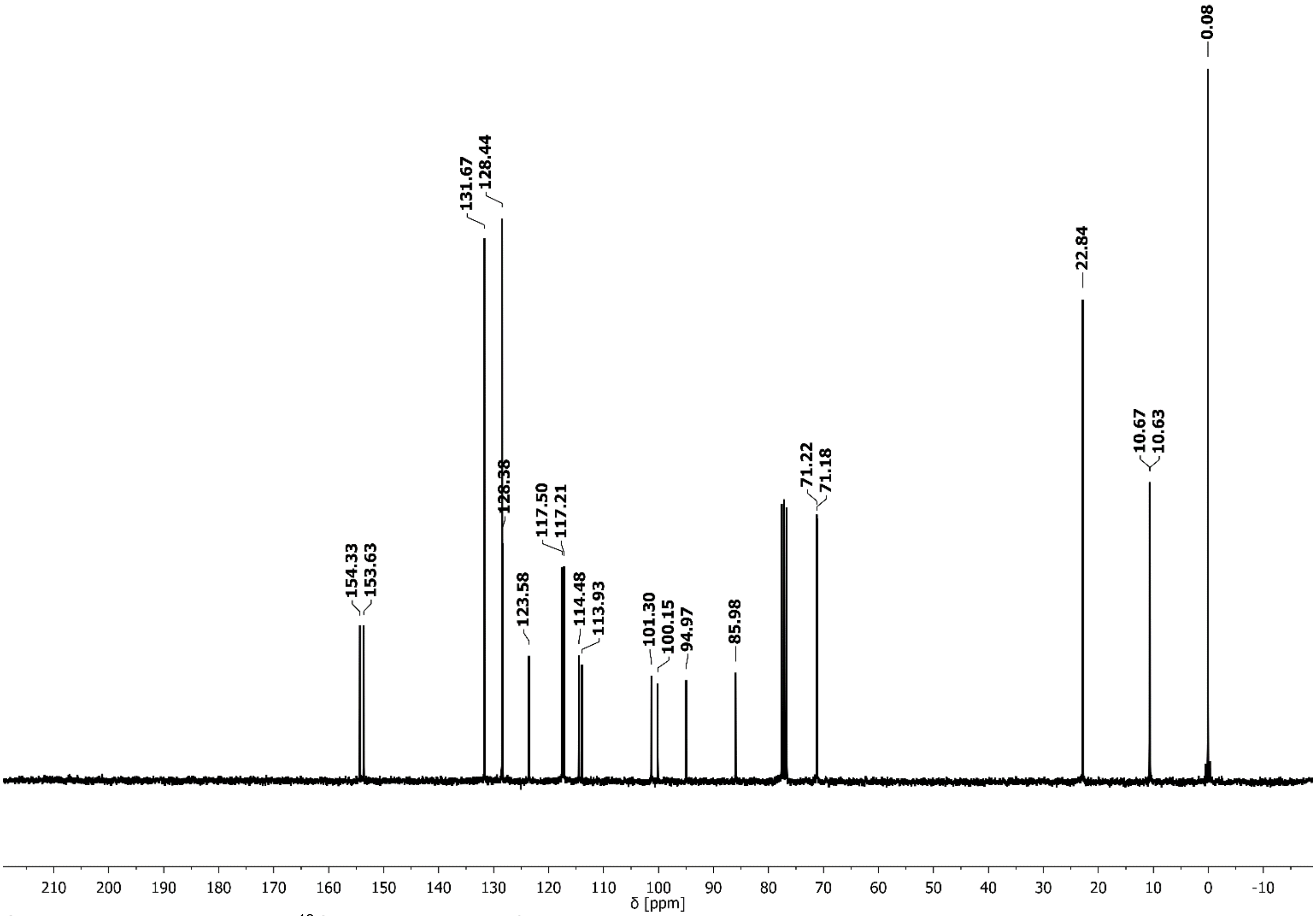

Supplementary Figure 32: ^{13}C NMR spectrum of protected monomer **2**.

Synthesis of 1-ethynyl-4-(phenylethynyl)-2,5-dipropoxybenzene **3**

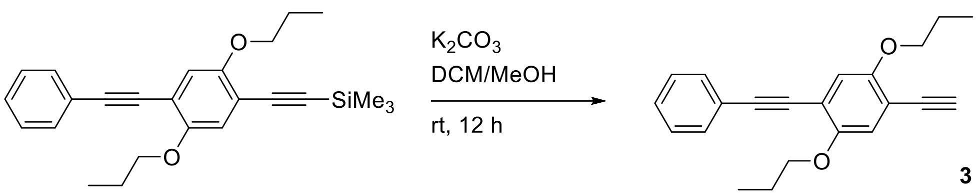

3

Trimethyl((4-(phenylethynyl)-2,5-dipropoxyphenyl)ethynyl)silane **2** (4.00 g, 10.2 mmol, 1.00 eq.) and two equivalents of potassium carbonate (2.83 g, 20.5 mmol) were placed into a Schlenk flask and degassed three times. Under continuous argon flow, 200 mL dry dichloromethane and 200 mL dry methanol were added. The reaction mixture was stirred overnight at room temperature under argon atmosphere and quenched with distilled water. The aqueous phase was extracted three times with dichloromethane, dried over Na_2SO_4, filtered and concentrated under reduced pressure. The residue was purified by flash silica column chromatography (cyclohexane / ethyl acetate 20:1) to yield the product as an orange solid (3.15 g, 97%). TLC (cyclohexane / dichloromethane 4:1) R_f = 0.22; ^{1}H NMR ($CDCl_3$, 300 MHz): *δ* (ppm) = 7.60-7.47 (m, 2 H, 2 C$\mathbf{H}_{\mathbf{aromatic}}$C-C≡C), 7.43-7.28 (m, 3 H, 3 C$\mathbf{H}_{\mathbf{aromatic}}$), 7.00, 6.98 (2 s, 2 H, 2 C$\mathbf{H}_{\mathbf{aromatic}}$CO), 3.98 (dt, *J* = 6.5, 4.3 Hz, 4 H, 2 C$\mathbf{H_2}$O), 3.35 (s, 1 H, 1 C≡C-**H**), 1.96-1.75 (m, 4 H, 2 C$\mathbf{H_2}$$CH_3$), 1.08 (dt, *J* = 9.8, 7.4 Hz, 6 H, 2 C$\mathbf{H_3}$); ^{13}C NMR ($CDCl_3$, 75 MHz): *δ* (ppm) = 154.33, 153.63, 131.72, 128.47, 123.55, 118.14, 117.18, 114.95, 112.81, 95.04, 85.83, 82.38, 80.15, 71.34, 71.29, 22.85, 22.72, 10.67, 10.59; FAB of $C_{22}H_{22}O_2$ (M+H$^+$ = 319.2); HRMS (FAB) of $C_{22}H_{22}O_2$ [M+H$^+$] calc. 318.1614, found 318.1614; IR (ATR) ν = 3259.6, 2960.2, 2936.7, 2876.7, 1593.3, 1501.4, 1469.4, 1440.3, 1389.9, 1275.5, 1212.1, 1065.9, 1042.0, 1013.4, 967.0, 911.1, 873.5, 857.7, 751.2, 687.3, 629.1, 527.1, 467.8, 387.4 cm^{-1}.

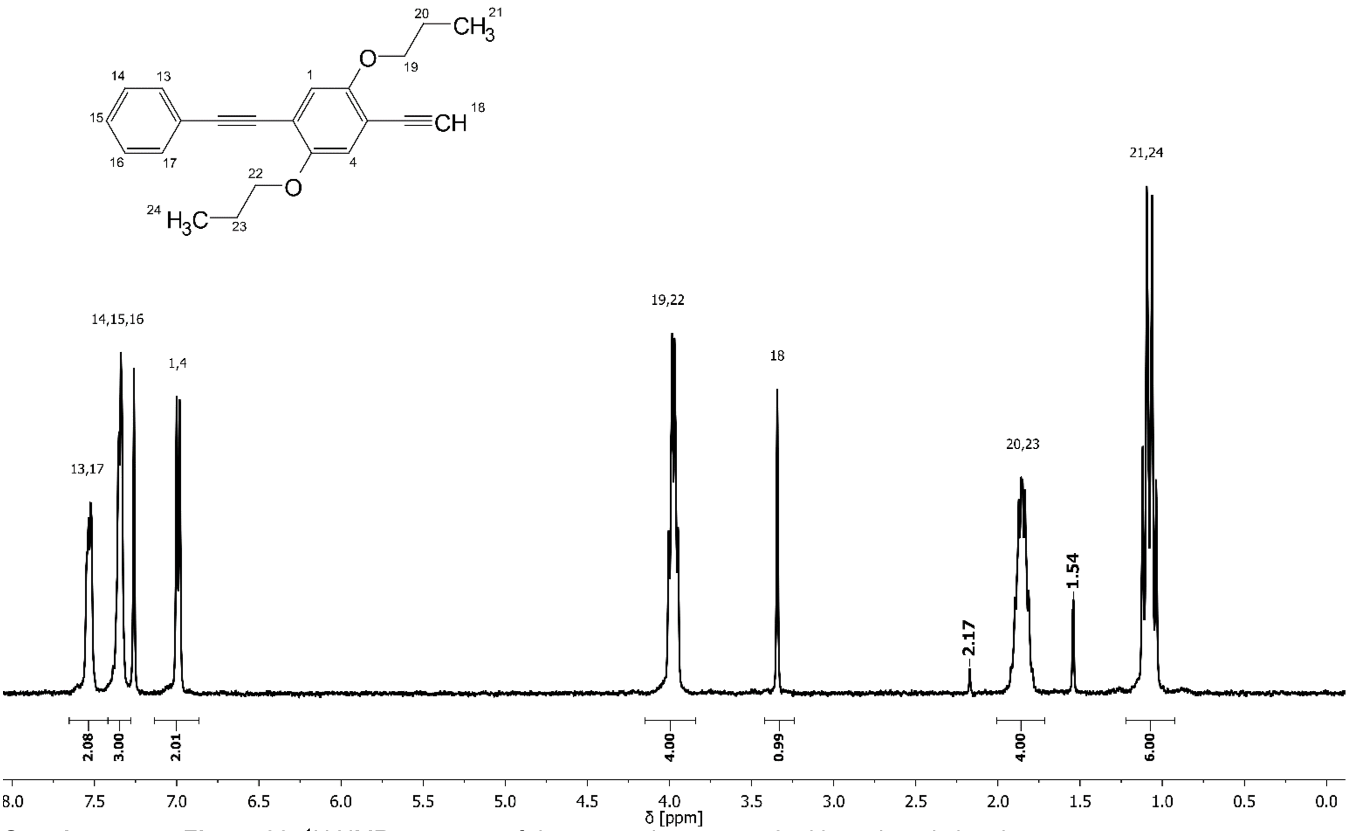

Supplementary Figure 33: ^{1}H NMR spectrum of deprotected monomer **3** with assigned signals.

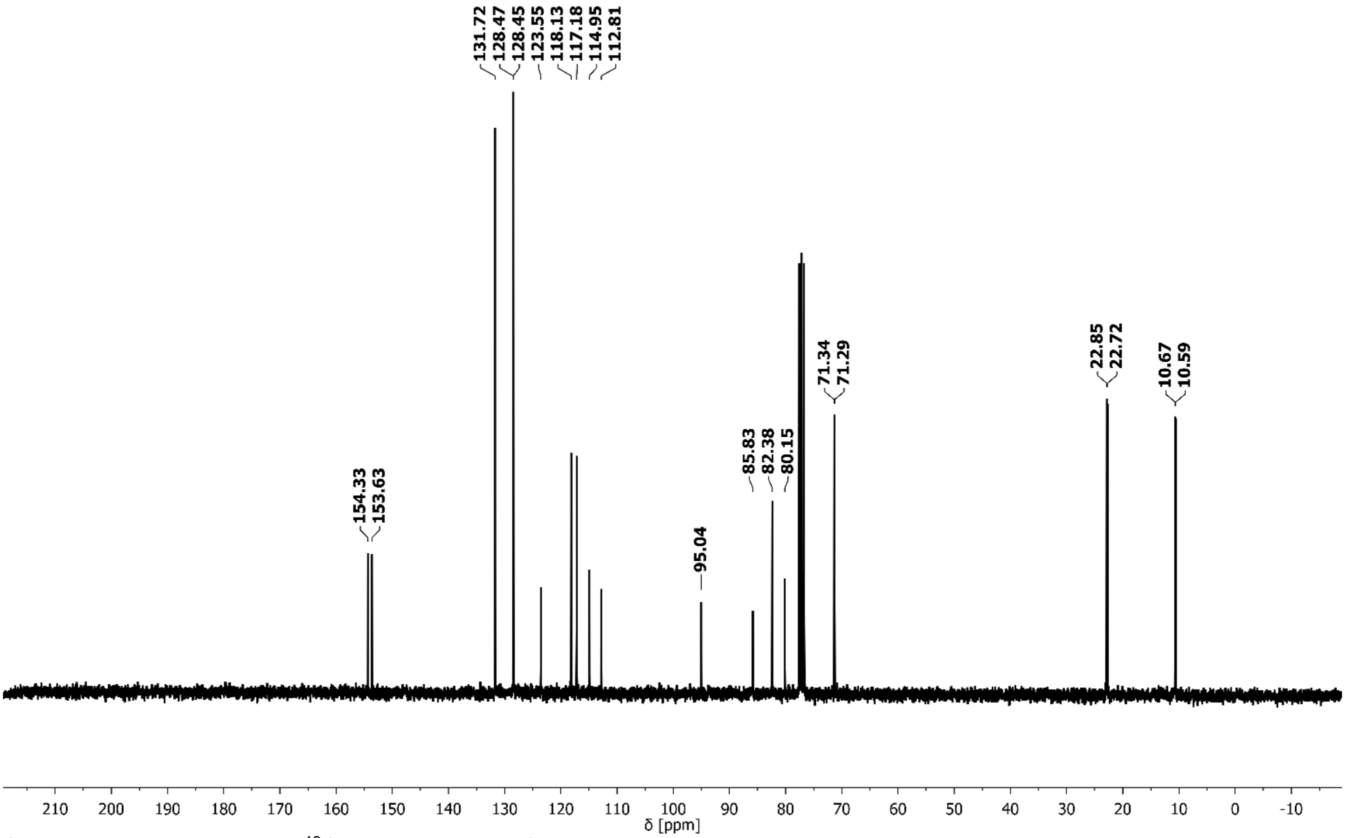

Supplementary Figure 34: ^{13}C NMR spectrum of deprotected monomer **3**.

Synthesis of trimethyl((4-((4-(phenylethynyl)-2,5-dipropoxyphenyl)ethynyl)-2,5-dipropoxyphenyl)ethynyl) silane **4**

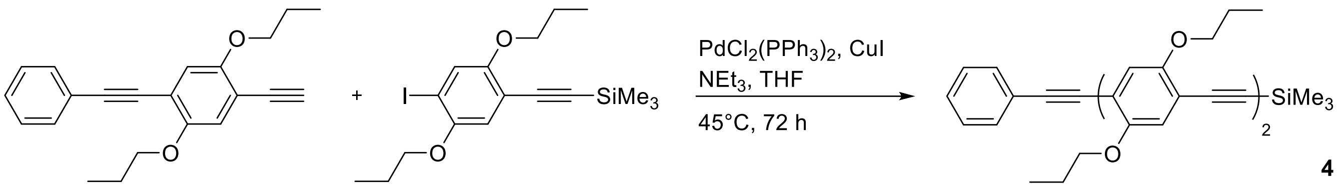

4

1,4-Bis(propyloxy)-2-iodo-5-trimethylsilylacetylenebenzene **1** (7.50 g, 18.0 mmol, 2.87 eq.), 5 mol% bis(triphenylphosphine) palladium(II) dichloride (220 mg, 314 µmol) and 5 mol% copper(I) iodide (59.8 mg, 314 µmol) were placed into a Schlenk flask and degassed three times. Under continuous argon flow, 100 mL dry THF and 8.71 mL dry triethylamine (6.36 g, 62.8 mmol, 10.0 eq.) were added and the mixture was stirred for 10 minutes. Subsequently, 1-ethynyl-4-(phenylethynyl)-2,5-dipropoxybenzene **3** (2.00 g, 6.28 mmol, 1.00 eq.) in 20 mL dry THF was added dropwise with a syringe. The reaction mixture was stirred for 72 h (3 d) at 45 °C, taken up in dichloromethane and washed with saturated NH_4Cl solution. The aqueous phase was extracted three times with dichloromethane. The combined organic layers were dried over Na_2SO_4, filtered and concentrated under reduced pressure. The residue was purified by silica column chromatography (cyclohexane / dichloromethane 3:1 → 3:2) and a flash silica column (cyclohexane / ethyl acetate 20:1) to yield the product as a yellow-orange solid (3.20 g, 84%). TLC (cyclohexane / dichloromethane 2:1) R_f = 0.35; ^{1}H NMR ($CDCl_3$, 300 MHz): δ (ppm) = 7.58-7.48 (m, 2 H, 2 C**H**$_{\mathbf{aromatic}}$C-C≡C), 7.40-7.30 (m, 3 H, 3 C**H**$_{\mathbf{aromatic}}$), 7.01 (s, 2 H, 2 C**H**$_{\mathbf{aromatic}}$CO), 6.97 (s, 1 H, 1 C**H**$_{\mathbf{aromatic}}$COC-C≡C-Si), 6.94 (s, 1 H, 1 C**H**$_{\mathbf{aromatic}}$C-C≡C-Si), 4.14–3.82 (m, 8 H, 4 C**H**$_{\mathbf{2}}$O), 1.99-1.71 (m, 8 H, 4 C**H**$_{\mathbf{2}}$$CH_3$), 1.20-0.94 (m, 12 H, 4 C**H**$_{\mathbf{3}}$), 0.26 (s, 9 H, 3 C**H**$_{\mathbf{3}}$Si); ^{13}C NMR ($CDCl_3$, 75 MHz): δ (ppm) = 154.26, 153.70, 153.57, 153.42, 131.63, 128.42, 128.34, 123.58, 117.56, 117.36, 117.28, 114.72, 114.37, 114.20, 113.89, 101.31, 100.18, 94.98, 91.62, 91.47, 86.09, 71.27, 71.24, 71.15, 71.08, 22.81, 22.79, 22.76, 10.63, 10.59, 0.04; FAB of $C_{39}H_{46}O_4Si$ (M+H$^+$ = 607.3); HRMS (FAB) of $C_{39}H_{46}O_4Si$ [M+H$^+$] calc. 606.3160, found 606.3161; IR (ATR) ν = 2961.3, 2873.4, 2150.4, 1596.0, 1506.0, 1466.0, 1420.3, 1384.1, 1271.6, 1248.3, 1205.1, 1060.8, 1010.7, 983.0, 892.2, 838.8, 754.7, 689.3, 637.4, 528.2 cm^{-1}.

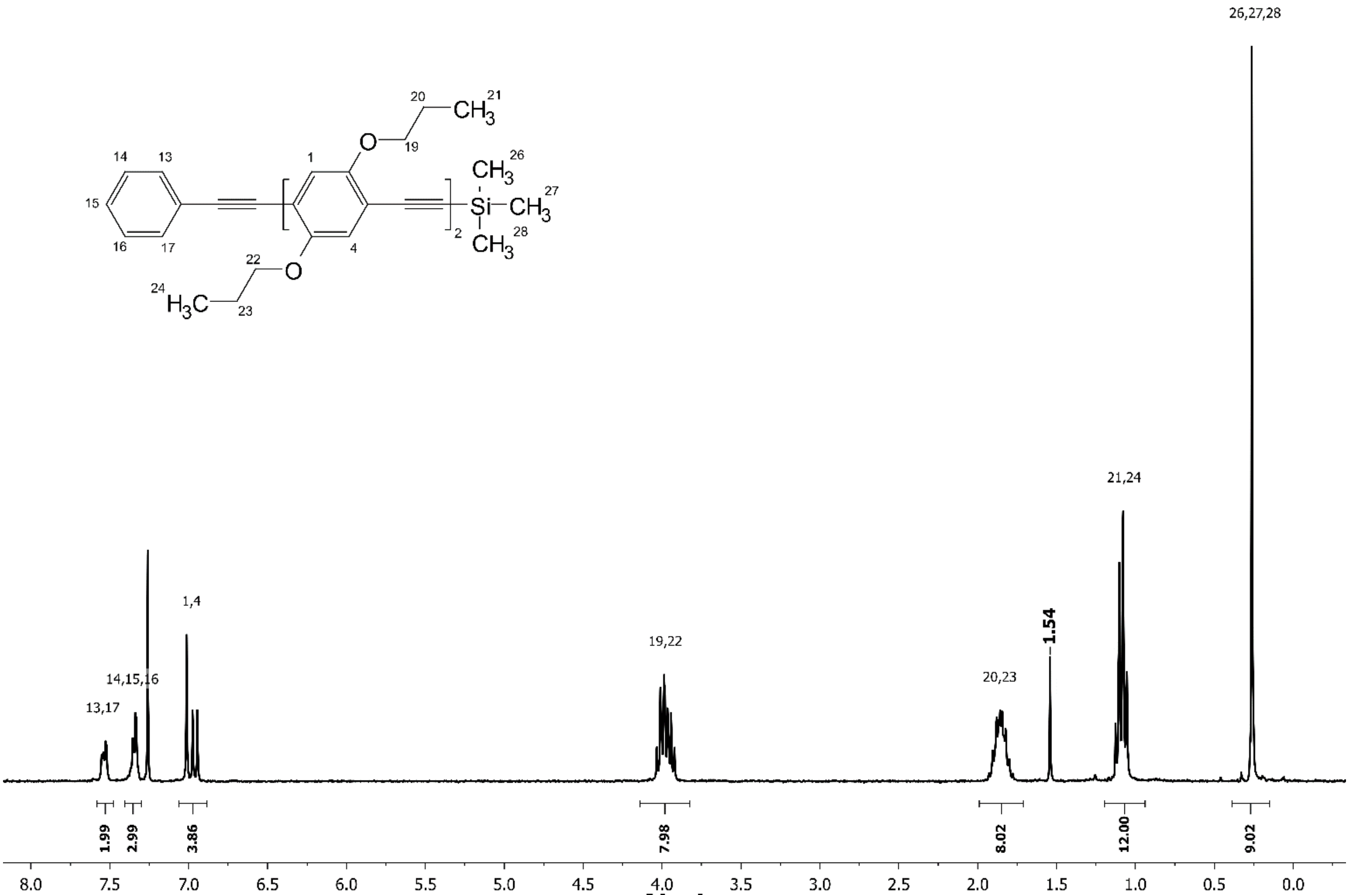

Supplementary Figure 35: ^{1}H NMR spectrum of monodisperse, protected dimer **4** with assigned signals.

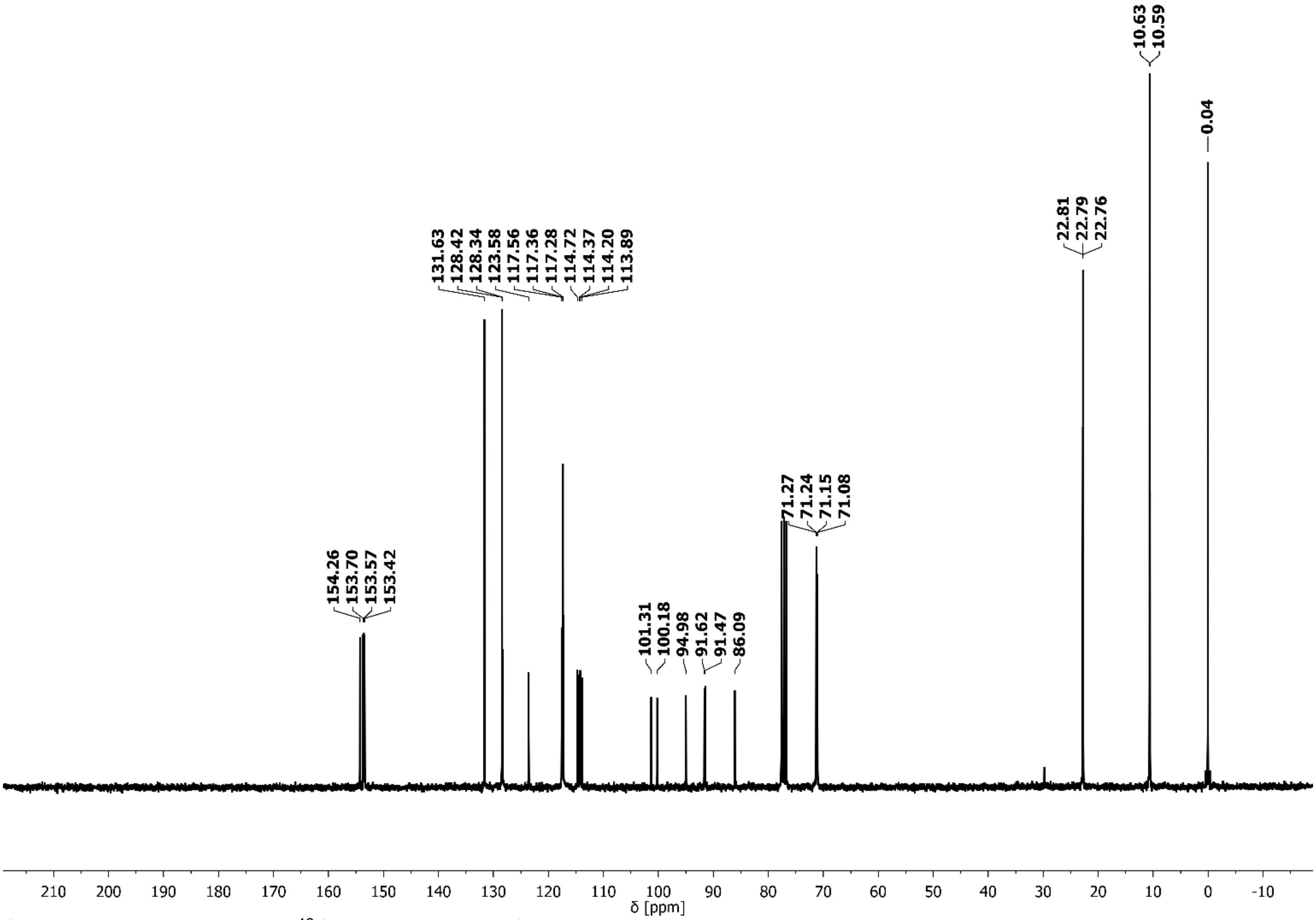

Supplementary Figure 36: ^{13}C NMR spectrum of monodisperse, protected dimer **4**.

Synthesis of 1-ethynyl-4-((4-(phenylethynyl)-2,5-dipropoxyphenyl)ethynyl)-2,5-dipropoxybenzene **5**

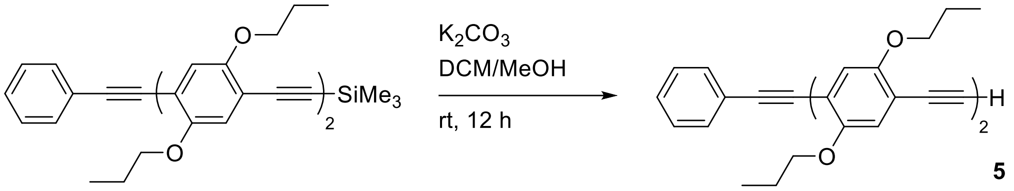

5

Compound **4** (3.00 g, 4.94 mmol, 1.00 eq.) and two equivalents of potassium carbonate (1.37 g, 9.89 mmol) were placed in a Schlenk flask and degassed three times. Under continuous argon flow, 150 mL dichloromethane and 150 mL methanol were added. The reaction mixture was stirred overnight at room temperature under argon atmosphere and quenched with distilled water. The aqueous phase was extracted three times with dichloromethane, dried over Na_2SO_4, filtered and concentrated under reduced pressure. The residue was purified by silica column chromatography (cyclohexane / ethyl acetate 20:1) to yield the product as a yellow solid (2.63 g, 100%). TLC (cyclohexane / dichloromethane 4:1) R_f = 0.33; ^{1}H NMR ($CDCl_3$, 300 MHz): δ (ppm) = 7.62-7.47 (m, 2 H, 2 C**H**$_{\textbf{aromatic}}$C-C≡C), 7.43-7.28 (m, 3 H, 3 C**H**$_{\textbf{aromatic}}$), 7.12-6.88 (m, 4 H, 4 C**H**$_{\textbf{aromatic}}$CO), 4.16-3.87 (m, 8 H, 4 C**H$_2$**O), 3.34 (s, 1 H, 1 C≡C-**H**), 1.99-1.72 (m, 8 H, 4 C**H$_2$**CH_3), 1.21-0.97 (m, 12 H, 4 C**H$_3$**); ^{13}C NMR ($CDCl_3$, 75 MHz): δ (ppm) = 154.28, 153.77, 153.67, 153.43, 131.68, 128.45, 128.38, 123.62, 118.24, 117.49, 117.38, 117.34, 115.19, 114.36, 112.79, 95.03, 91.67, 91.29, 86.10, 82.42, 80.17, 71.38, 71.35, 71.27, 71.21, 22.85, 22.81, 22.78, 22.67, 10.66, 10.55; FAB of $C_{36}H_{38}O_4$ (M+H$^+$ = 535.4); HRMS (FAB) of $C_{36}H_{38}O_4$ [M+H$^+$] calc. 534.2765, found 534.2768; IR (ATR) v = 3279.3, 2961.5, 2932.5, 2873.8, 1597.1, 1505.1, 1467.4, 1418.6, 1383.9, 1271.7, 1206.3, 1105.7, 1061.2, 1011.2, 982.5, 859.2, 755.1, 689.7, 660.6, 528.5 cm^{-1}.

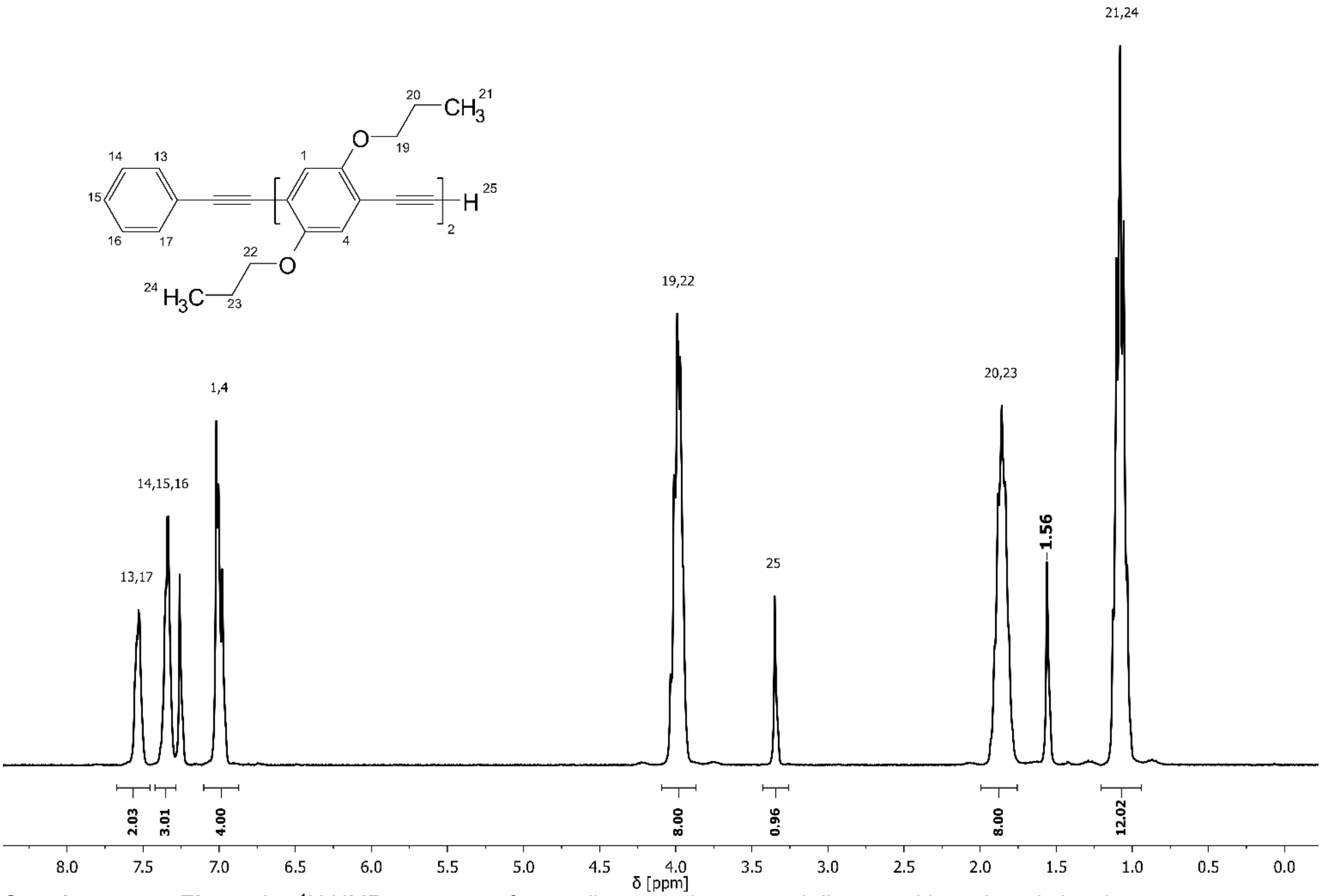

Supplementary Figure 37: ^{1}H NMR spectrum of monodisperse, deprotected dimer **5** with assigned signals.

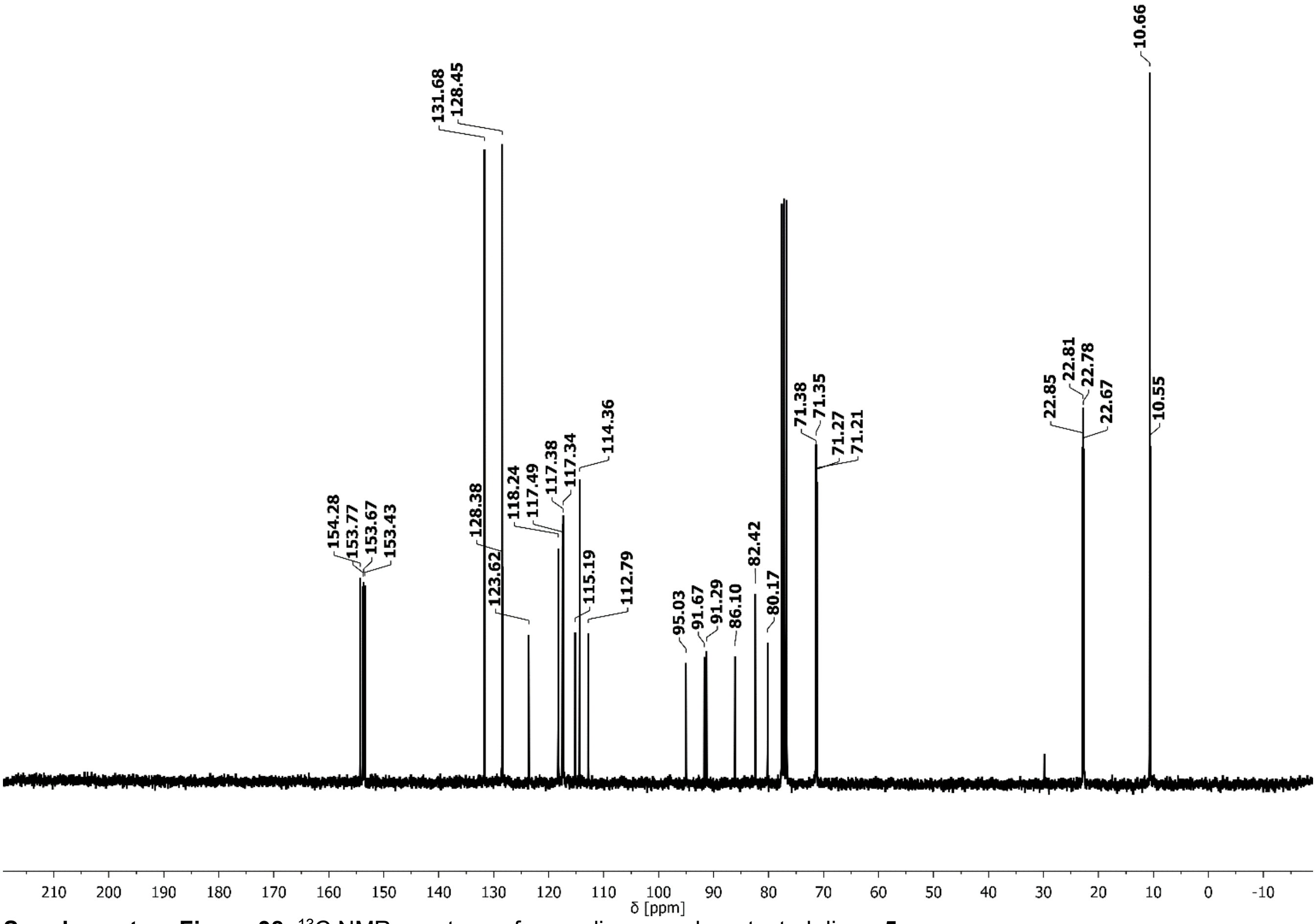

Supplementary Figure 38: ^{13}C NMR spectrum of monodisperse, deprotected dimer **5**.

Synthesis of trimethyl((4-((4-((4-(phenylethynyl)-2,5-dipropoxyphenyl)ethynyl)-2,5-dipropoxyphenyl)ethynyl)-2,5-dipropoxyphenyl)ethynyl)silane **6**

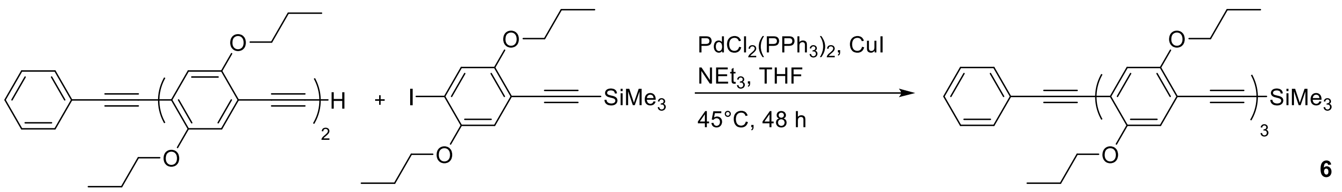

1,4-Bis(propyloxy)-2-iodo-5-trimethylsilylacetylenebenzene **1** (4.67 g, 11.2 mmol, 3.00 eq.), 5 mol% bis(triphenylphosphine) palladium(II) dichloride (131 mg, 187 µmol) and 5 mol% copper(I) iodide (35.6 mg, 187 µmol) were placed into a Schlenk flask. Under continuous argon flow, 130 mL dry THF and 5.20 mL dry triethylamine (3.78 g, 37.4 mmol, 10.0 eq.) were added and the mixture was stirred for 10 minutes. Subsequently, compound **5** (2.00 g, 3.74 mmol, 1.00 eq.) in 20 mL THF was added dropwise with a syringe. The reaction mixture was stirred for 48 h (2 d) at 45 °C, taken up in dichloromethane and washed with saturated NH_4Cl solution. The aqueous phase was extracted three times with dichloromethane. The combined organic layers were dried over Na_2SO_4, filtered and concentrated under reduced pressure. The residue was purified by silica column chromatography (cyclohexane / dichloromethane 2:1 → 1:1) and a flash silica column (cyclohexane / ethyl acetate 20:1) to yield the product as an orange solid (2.10 g, 68%). TLC (cyclohexane / dichloromethane 2:1) R_f = 0.16; ^{1}H NMR ($CDCl_3$, 300 MHz): δ (ppm) = 7.65-7.44 (m, 2 H, 2 C**H**$_{\mathbf{aromatic}}$C-C≡C), 7.43-7.28 (m, 3 H, 3 C**H**$_{\mathbf{aromatic}}$), 7.02 (s, 4 H, 4 C**H**$_{\mathbf{aromatic}}$CO), 6.98 (s, 1 H, 1 C**H**$_{\mathbf{aromatic}}$COC-C≡C-Si), 6.95 (s, 1 H, 1 C**H**$_{\mathbf{aromatic}}$C-C≡C-Si), 3.98 (ddt, *J* = 12.2, 10.2, 6.5 Hz, 12 H, 6 C$\mathbf{H_2}$O), 2.03-1.67 (m, 12 H, 6 C$\mathbf{H_2}$$CH_3$), 1.17-0.98 (m, 18 H, 6 C$\mathbf{H_3}$), 0.26 (s, 9 H, 3 C$\mathbf{H_3}$Si); ^{13}C NMR ($CDCl_3$, 75 MHz): δ (ppm) = 154.27, 153.72, 153.59, 153.54, 153.44, 131.68, 128.46, 128.38, 123.60, 117.54, 117.48, 117.34, 117.28, 114.70, 114.46, 114.42, 114.38, 114.17, 101.30, 100.25, 95.00, 91.66, 91.63, 91.55, 86.10, 71.30, 71.25, 71.19, 22.85, 22.78, 10.69, 0.08; FAB of $C_{53}H_{62}O_6Si$ (M+H$^+$ = 823.3); HRMS (FAB) of $C_{53}H_{62}O_6Si$ [M+H$^+$] calc. 822.4310, found 822.4309; IR (ATR) ν = 2961.7, 2873.6, 2146.8, 1510.8, 1470.2, 1420.1, 1389.2, 1274.1, 1212.3, 1042.8, 1024.3, 902.8, 837.5, 751.7, 687.8, 527.3 cm^{-1}.

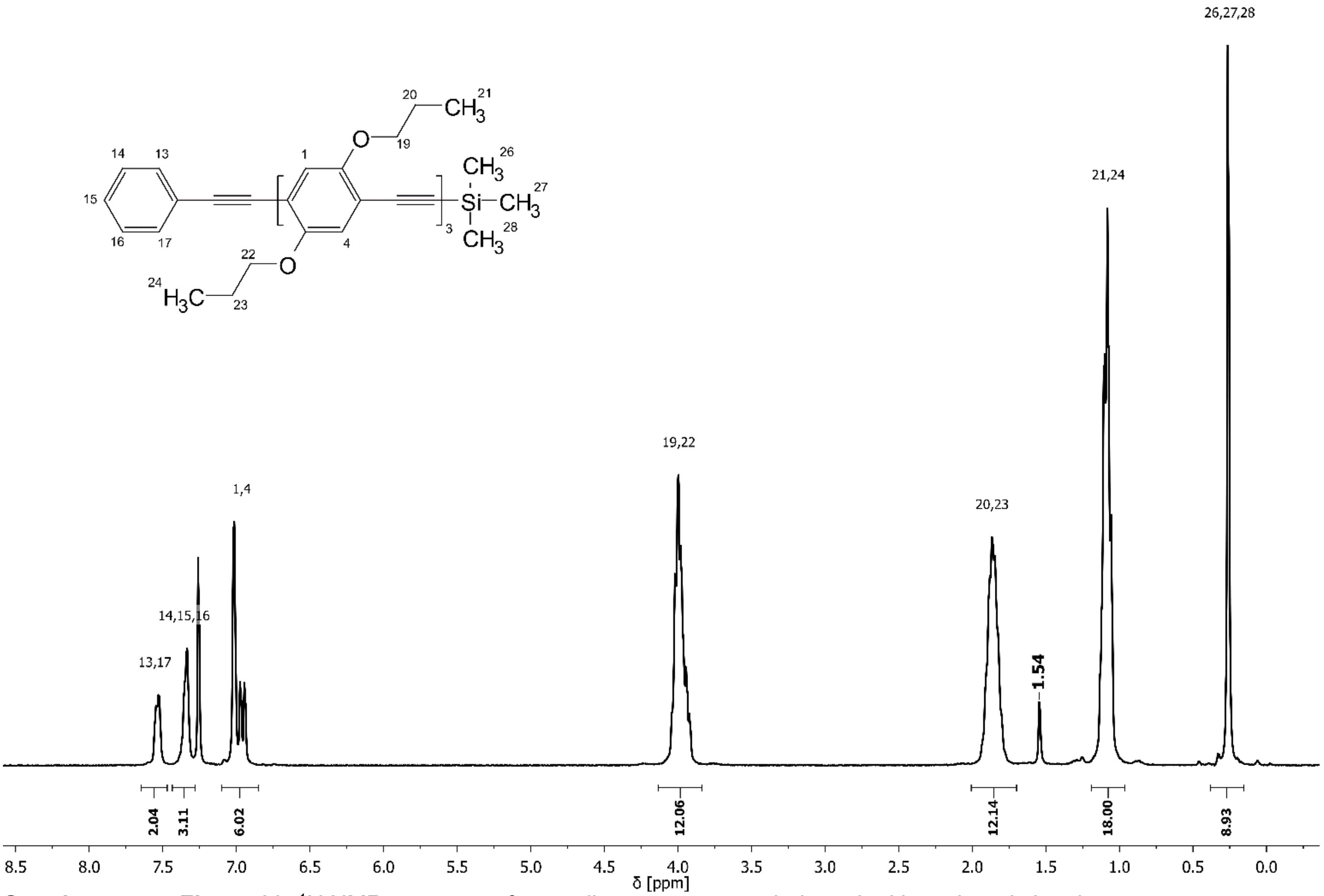

Supplementary Figure 39: ^{1}H NMR spectrum of monodisperse, protected trimer **6** with assigned signals.

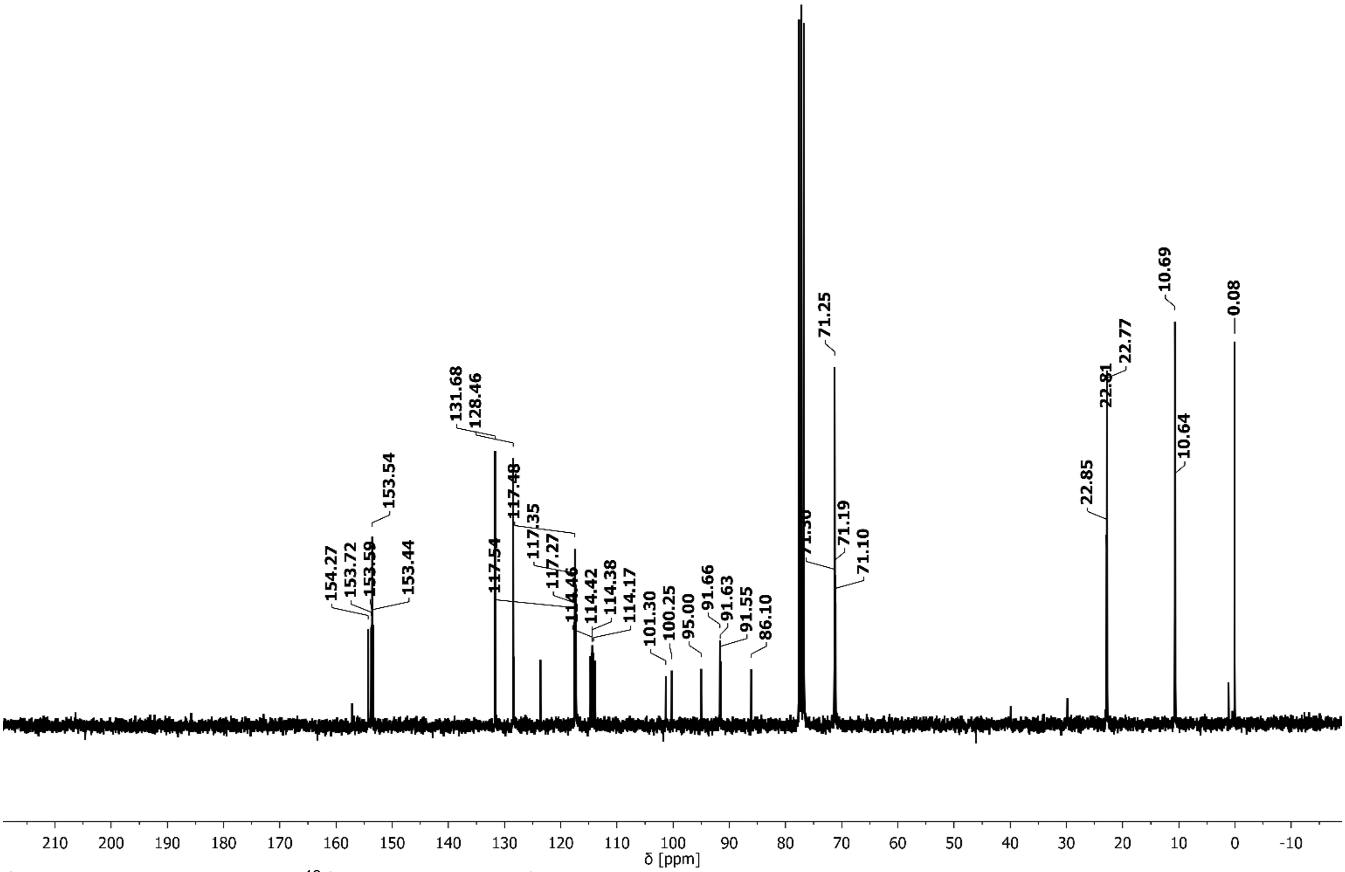

Supplementary Figure 40: ^{13}C NMR spectrum of monodisperse, protected trimer **6**.

Synthesis of 1-ethynyl-4-((4-((4-(phenylethynyl)-2,5-dipropoxyphenyl)ethynyl)-2,5-dipropoxyphenyl) ethynyl)-2,5-dipropoxybenzene **7**

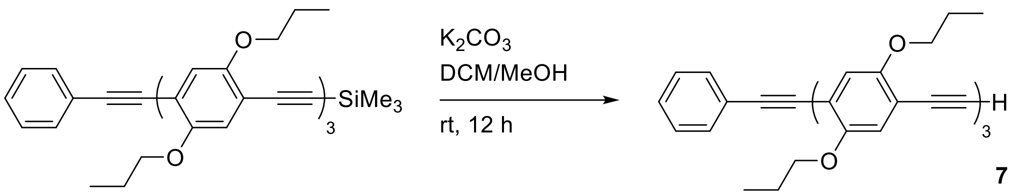

7

Compound **6** (1.90 g, 2.31 mmol, 1.00 eq.) and two equivalents of potassium carbonate (639 mg, 4.62 mmol) were added to 100 mL dichloromethane and 100 mL methanol. The reaction mixture was degassed with argon and stirred overnight at room temperature and quenched with distilled water. The aqueous phase was extracted three times with dichloromethane, dried over Na_2SO_4, filtered and concentrated under reduced pressure. The residue was purified by silica column chromatography (cyclohexane / ethyl acetate 20:1) to yield the product as a yellow solid (1.74 g, 98%). TLC (cyclohexane / dichloromethane 2:1) R_f = 0.14; ^{1}H NMR ($CDCl_3$, 300 MHz): δ (ppm) = 7.66-7.45 (m, 2 H, 2 C$\mathbf{H}_{\mathbf{aromatic}}$C-C≡C), 7.45-7.29 (m, 3 H, 3 C$\mathbf{H}_{\mathbf{aromatic}}$), 7.11-6.91 (m, 6 H, 6 C$\mathbf{H}_{\mathbf{aromatic}}$CO), 4.14-3.87 (m, 12 H, 6 C$\mathbf{H_2}$O), 3.35 (s, 1 H, 1 C≡C-**H**), 2.06-1.75 (m, 12 H, 6 C$\mathbf{H_2}$$CH_3$), 1.23-0.94 (m, 18 H, 6 C$\mathbf{H_3}$); ^{13}C NMR ($CDCl_3$, 75 MHz): δ (ppm) = 154.19, 153.69, 153.55, 153.35, 131.63, 128.42, 128.34, 123.56, 118.08, 117.45, 117.32, 117.20, 117.20, 115.06, 114.52, 114.36, 114.26, 114.16, 112.69, 95.14, 94.98, 91.68, 91.58, 91.33, 86.09, 82.46, 80.11, 71.25, 71.19, 71.14, 71.09, 22.80, 22.76, 22.73, 22.62, 10.62, 10.52; FAB of $C_{50}H_{54}O_6$ (M+H$^+$ = 751.6); HRMS (FAB) of $C_{50}H_{54}O_6$ [M+H$^+$] calc. 750.3915, found 750.3913; IR (ATR) v = 3288.0, 2958.7, 2932.8, 2873.3, 1595.7, 1508.1, 1469.9, 1423.0, 1385.9, 1272.5, 1204.2, 1062.8, 1041.1, 1020.9, 979.3, 906.5, 861.2, 769.9, 754.4, 687.7, 649.4, 528.2, 465.8 cm^{-1}.

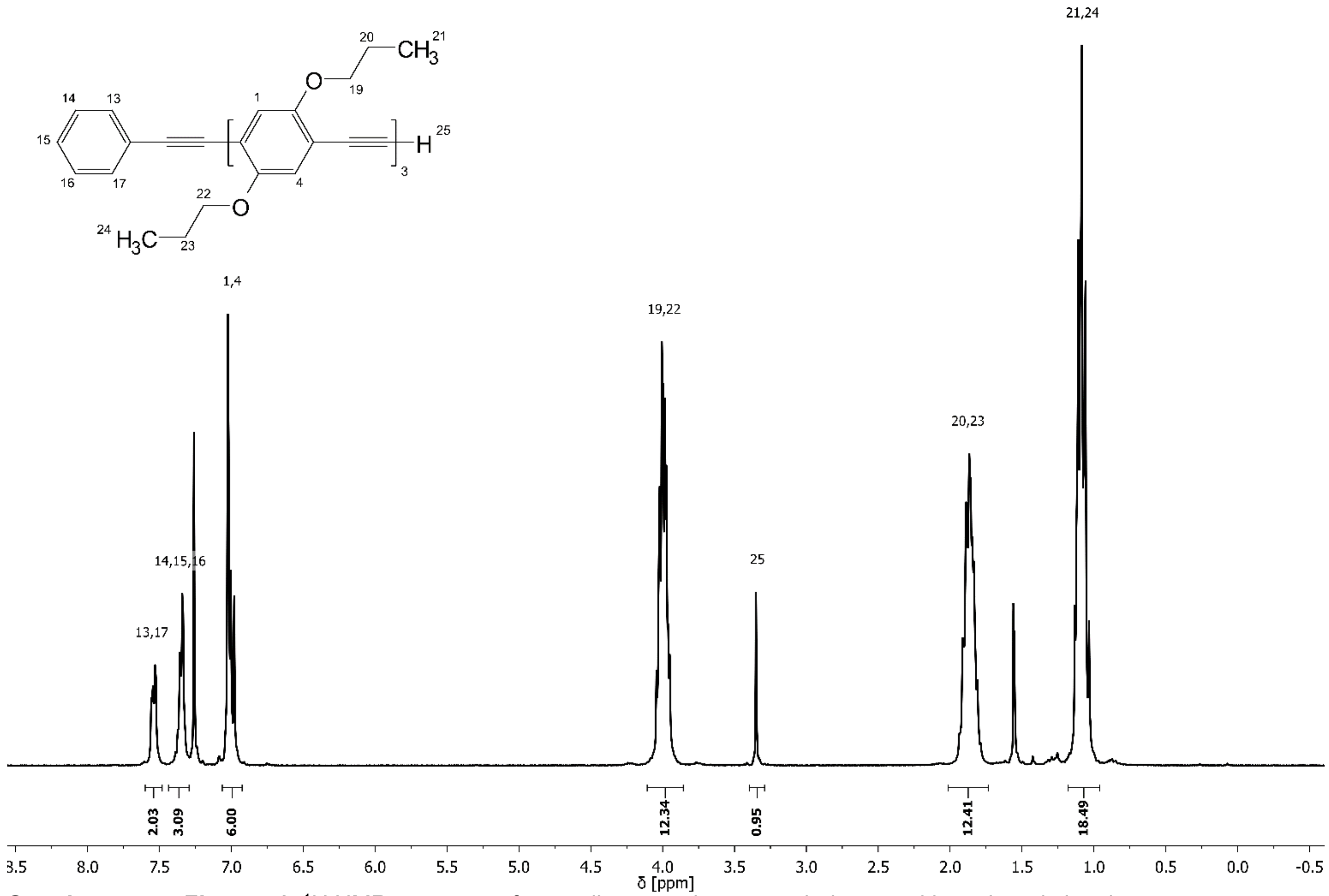

Supplementary Figure 41: ^{1}H NMR spectrum of monodisperse, deprotected trimer **7** with assigned signals.

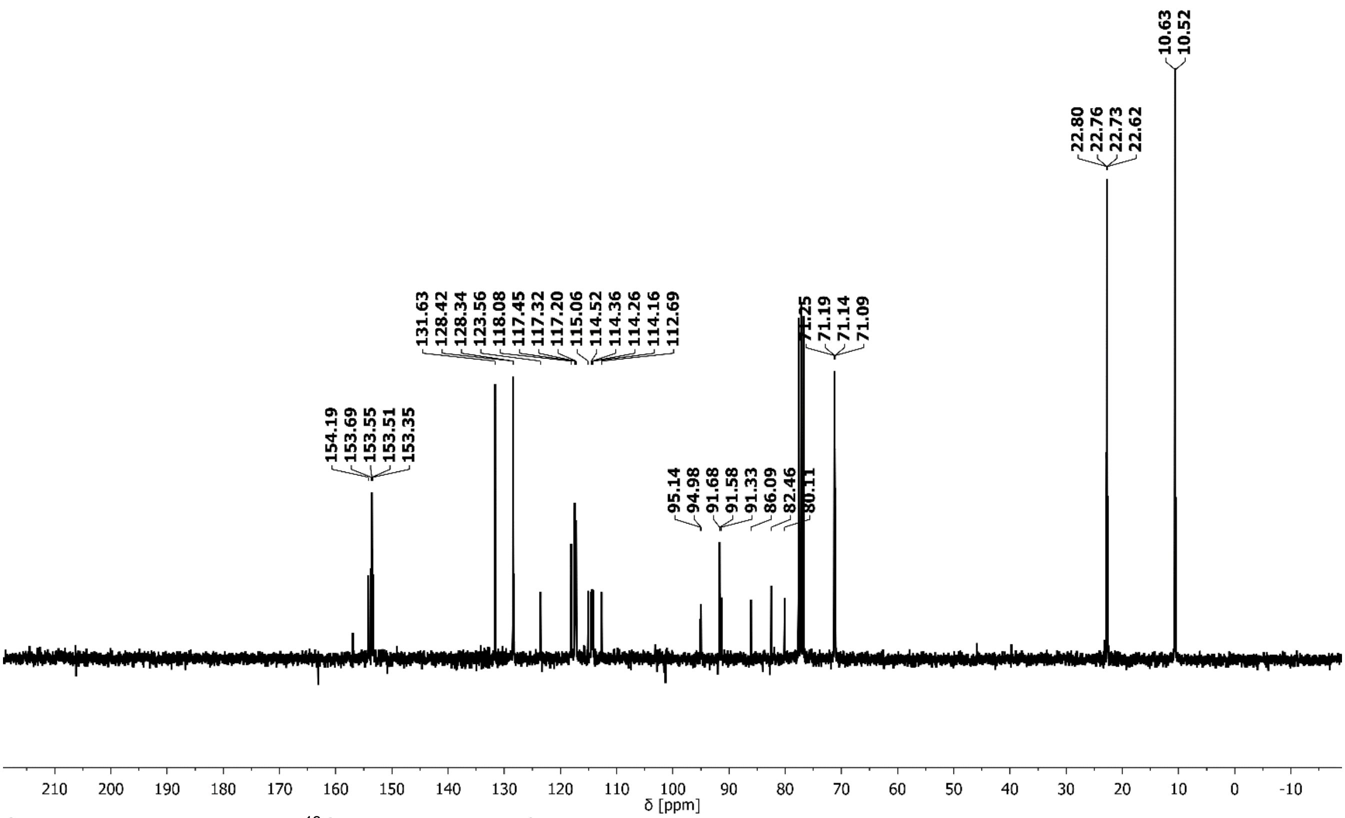

Supplementary Figure 42: ^{13}C NMR spectrum of monodisperse, deprotected trimer **7**.

Synthesis of trimethyl((4-((4-((4-((4-(phenylethynyl)-2,5-dipropoxyphenyl)ethynyl)-2,5-dipropoxyphenyl)ethynyl)-2,5-dipropoxyphenyl)ethynyl)-2,5-dipropoxyphenyl)ethynyl)silane **8**

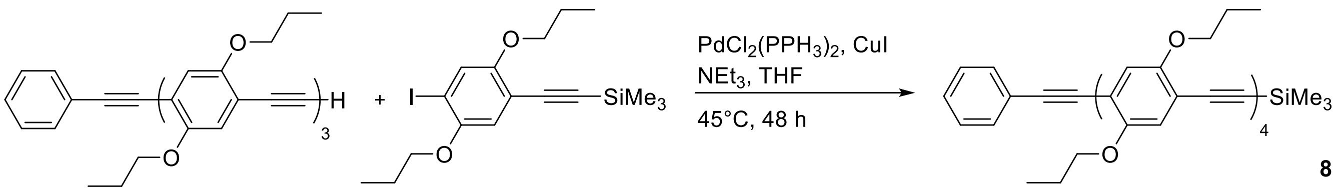

8

1,4-Bis(propyloxy)-2-iodo-5-trimethylsilylacetylenebenzene **1** (3.74 g, 9.00 mmol, 5.00 eq.), 5 mol% bis(triphenylphosphine) palladium(II) dichloride (63.1 mg, 90.0 µmol) and 5 mol% copper(I) iodide (17.1 mg, 90.0 µmol) were placed into a Schlenk flask and degassed three times. Under continuous argon flow, 70 mL dry THF and 2.49 mL dry triethylamine (1.82 g, 18.0 mmol, 10.0 eq.) were added and the mixture was stirred for 0.5 h. Subsequently, compound **7** (1.35 g, 1.80 mmol, 1.00 eq.) in 25 mL THF was added dropwise with a syringe under continuous argon flow. The reaction mixture was stirred for 48 h at 45 °C, taken up in dichloromethane and washed with saturated NH_4Cl solution. The aqueous phase was extracted three times with dichloromethane. The combined organic layers were dried over Na_2SO_4, filtered and concentrated under reduced pressure. The residue was purified by silica column chromatography (cyclohexane / dichloromethane 2:1→1:2) and recrystallisation from *n*-hexane to yield the product as a yellow solid (1.22 g, 65%). TLC (cyclohexane / dichloromethane 2:3) R_f = 0.34; ^{1}H NMR ($CDCl_3$, 300 MHz): δ (ppm) = 7.64–7.47 (m, 2 H, 2 C$\mathbf{H}_{\mathbf{aromatic}}$C-C≡C), 7.44–7.30 (m, 3 H, 3 C$\mathbf{H}_{\mathbf{aromatic}}$), 7.12–6.83 (m, 8 H, 8 C$\mathbf{H}_{\mathbf{aromatic}}$CO), 4.18–3.80 (m, 16 H, 8 C$\mathbf{H_2}$O), 1.97–1.74 (m, 16 H, 8 C$\mathbf{H_2}$$CH_3$), 1.20–0.97 (m, 24 H, 8 C$\mathbf{H_3}$), 0.26 (s, 9 H, 3 C$\mathbf{H_3}$Si); ^{13}C NMR ($CDCl_3$, 100 MHz): δ (ppm) = 154.29, 153.75, 153.62, 153.58, 153.46, 131.71, 128.47, 128.39, 123.63, 117.58, 117.52, 117.41, 117.38, 117.32, 114.73, 114.50, 114.47, 114.41, 114.19, 113.90, 101.31, 100.27, 95.02, 91.72, 91.70, 91.69, 91.66, 91.56, 86.12, 71.34, 71.28, 71.23, 71.13, 22.87, 22.82, 22.80, 22.78, 10.71, 10.69, 10.67, 10.65, 0.10; FAB of $C_{67}H_{78}O_8Si$ (M+H$^+$ = 1039.5); HRMS (FAB) of $C_{67}H_{78}O_8Si$ [M+H$^+$] calc. 1038.5466, found 1038.5463; IR (ATR) ν = 2962.11, 2934.63, 2874.83, 2148.77, 1943.89, 1595.96, 1493.76, 1468.98, 1421.24, 1383.35, 1272.49, 1248.71, 1205.75, 1105.78, 1061.34, 1043.86, 1010.75, 981.55, 839.86, 755.09, 689.75, 636.42, 579.20, 527.86, 465.76 cm^{-1}.

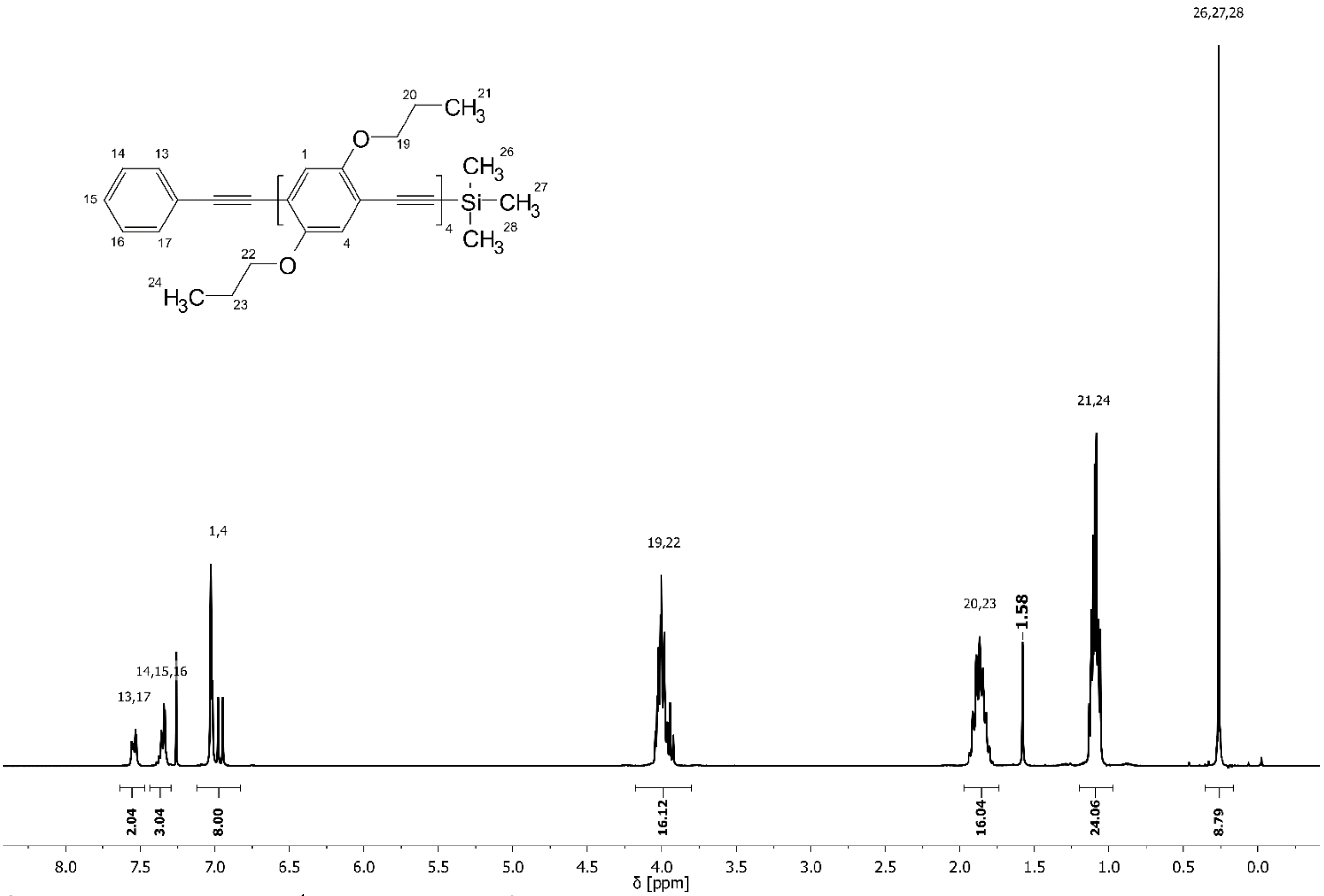

Supplementary Figure 43: ^1H NMR spectrum of monodisperse, protected tetramer **8** with assigned signals.

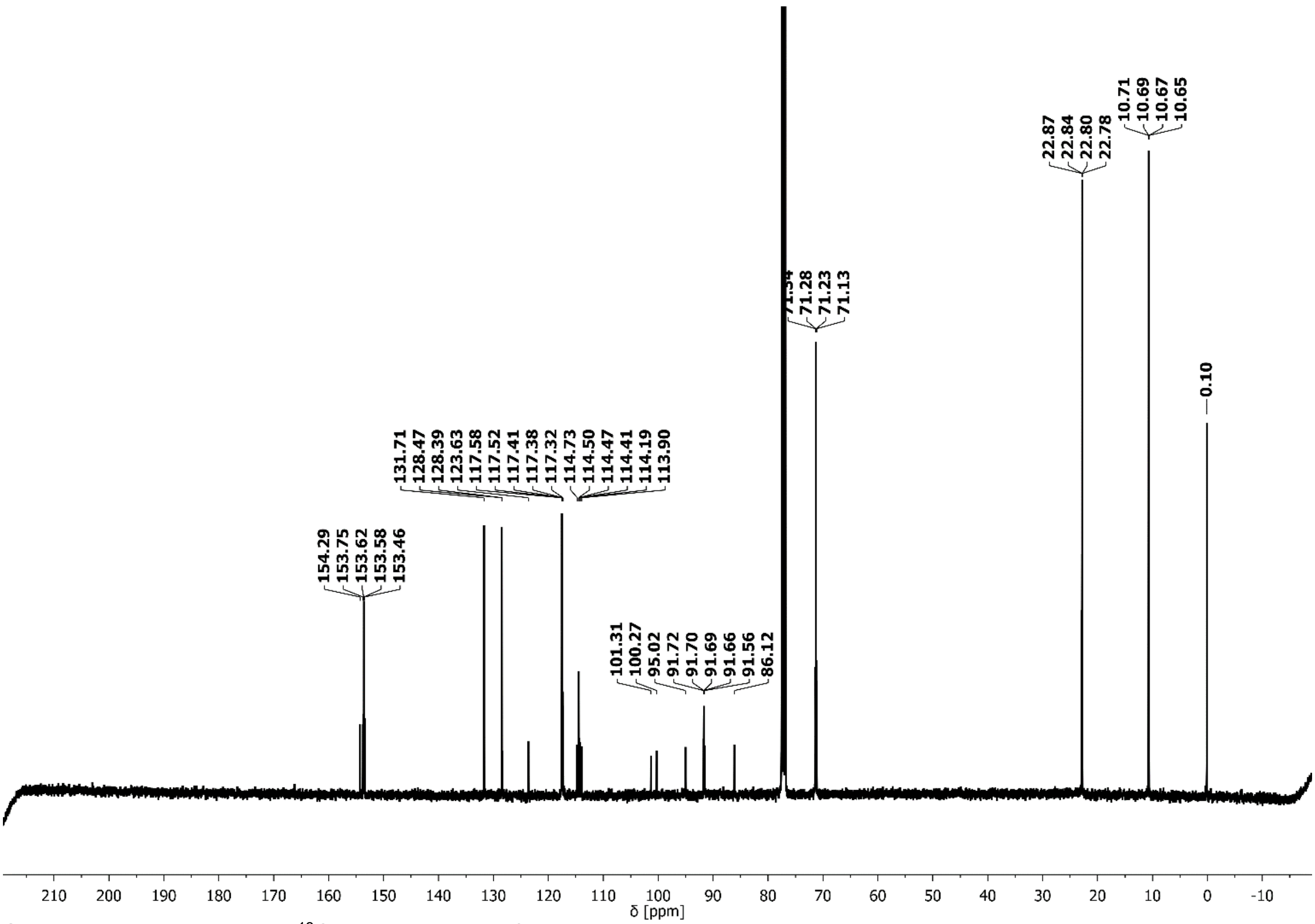

Supplementary Figure 44: ^{13}C NMR spectrum of monodisperse, protected tetramer **8**.

Synthesis of 1-ethynyl-4-((4-((4-((4-(phenylethynyl)-2,5-dipropoxyphenyl)ethynyl)-2,5-dipropoxyphenyl)ethynyl)-2,5-dipropoxyphenyl)ethynyl)-2,5-dipropoxybenzene **9**

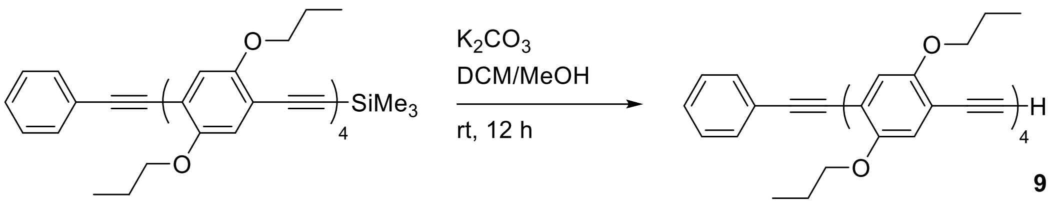

Compound **8** (739 mg, 0.711 mmol, 1.00 eq.) and two equivalents of potassium carbonate (197 mg, 1.42 mmol) were added to 40 mL dichloromethane and 40 mL methanol. The reaction mixture was degassed with argon and stirred overnight at room temperature and quenched with distilled water. The aqueous phase was extracted three times with dichloromethane, dried over Na_2SO_4, filtered and concentrated under reduced pressure. The residue was purified by recrystallisation from n-hexane to yield the product as a yellow-brown solid (682 mg, 99%). TLC (cyclohexane / dichloromethane 2:3) R_f = 0.31; ^{1}H NMR ($CDCl_3$, 300 MHz): δ (ppm) = 7.65–7.47 (m, 2 H, 2 C**H**$_{\mathbf{aromatic}}$C-C≡C), 7.47–7.29 (m, 3 H, 3 C**H**$_{\mathbf{aromatic}}$), 7.11–6.89 (m, 8 H, 8 C**H**$_{\mathbf{aromatic}}$CO), 4.11–3.88 (m, 16 H, 8 C**H**$_{\mathbf{2}}$O), 3.35 (s, 1 H, 1 C≡C-**H**), 2.01–1.66 (m, 16 H, 8 C**H**$_{\mathbf{2}}$$CH_3$), 1.23–0.92 (m, 24 H, 8 C**H**$_{\mathbf{3}}$); ^{13}C NMR ($CDCl_3$, 100 MHz): δ (ppm) = 154.24, 153.74, 153.60, 153.57, 153.40, 131.70, 128.47, 128.39, 123.61, 118.15, 117.51, 117.39, 117.31, 117.25, 115.13, 114.58, 114.48, 114.43, 114.30, 114.19, 112.70, 95.02, 91.75, 91.72, 91.67, 91.65, 91.34, 86.11, 82.44, 80.16, 77.36, 71.33, 71.28, 71.22, 71.16, 22.86, 22.81, 22.79, 22.67, 10.70, 10.68, 10.57; FAB of $C_{64}H_{70}O_8$ ($M+H^+$ = 967.4); HRMS (FAB) of $C_{64}H_{70}O_8$ [$M+H^+$] calc. 966.5071, found 966.5070; IR (ATR) ν = 3279.04, 2962.42, 2934.19, 2873.84, 2196.23, 2098.17, 1595.37, 1497.81, 1471.12, 1421.28, 1385.61, 1272.61, 1205.97, 1105.42, 1061.46, 1042.54, 1012.50, 982.24, 905.52, 857.47, 754.61, 689.37, 527.99, 462.05, 417.67 cm^{-1}.

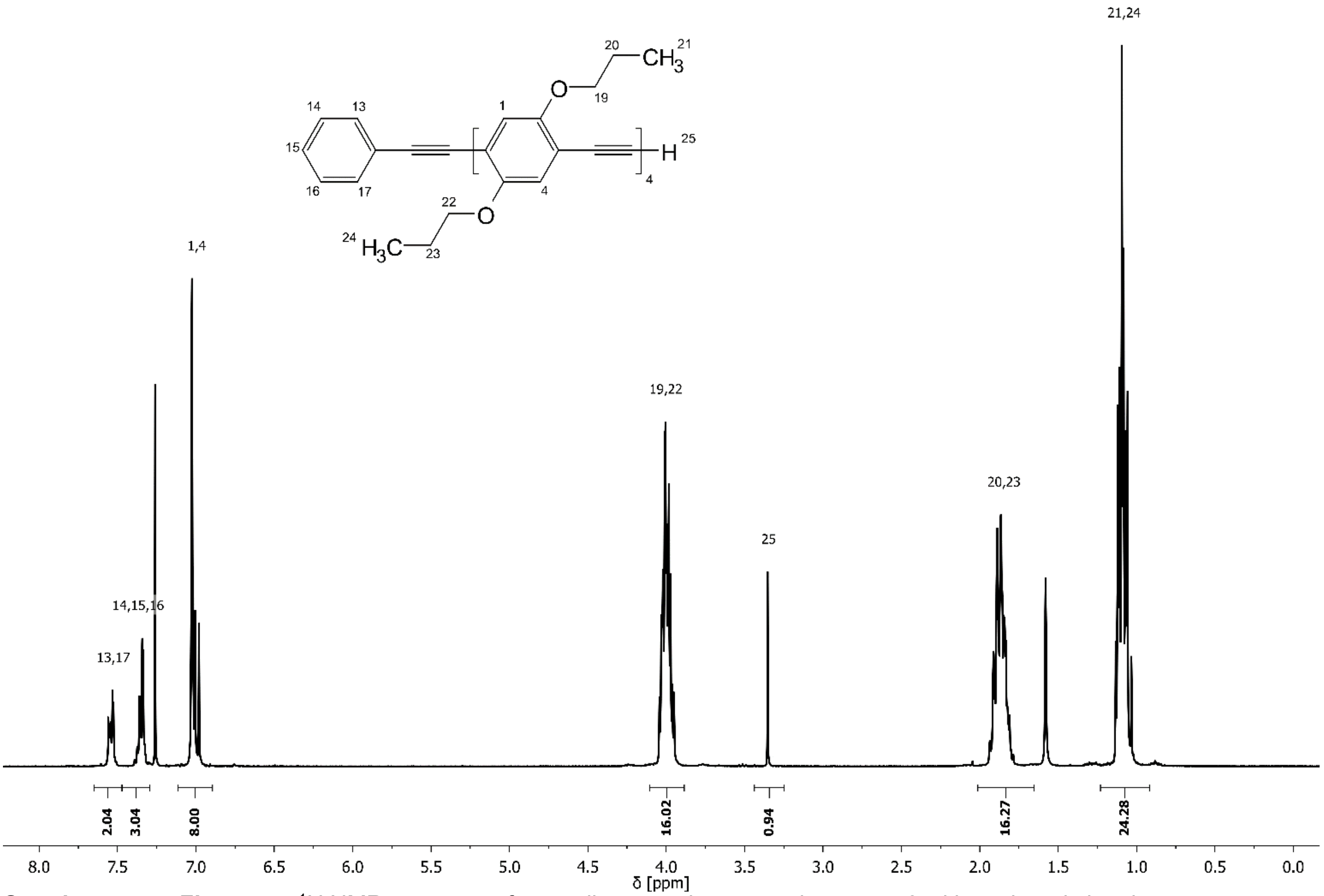

Supplementary Figure 45: ^{1}H NMR spectrum of monodisperse, deprotected tetramer **9** with assigned signals.

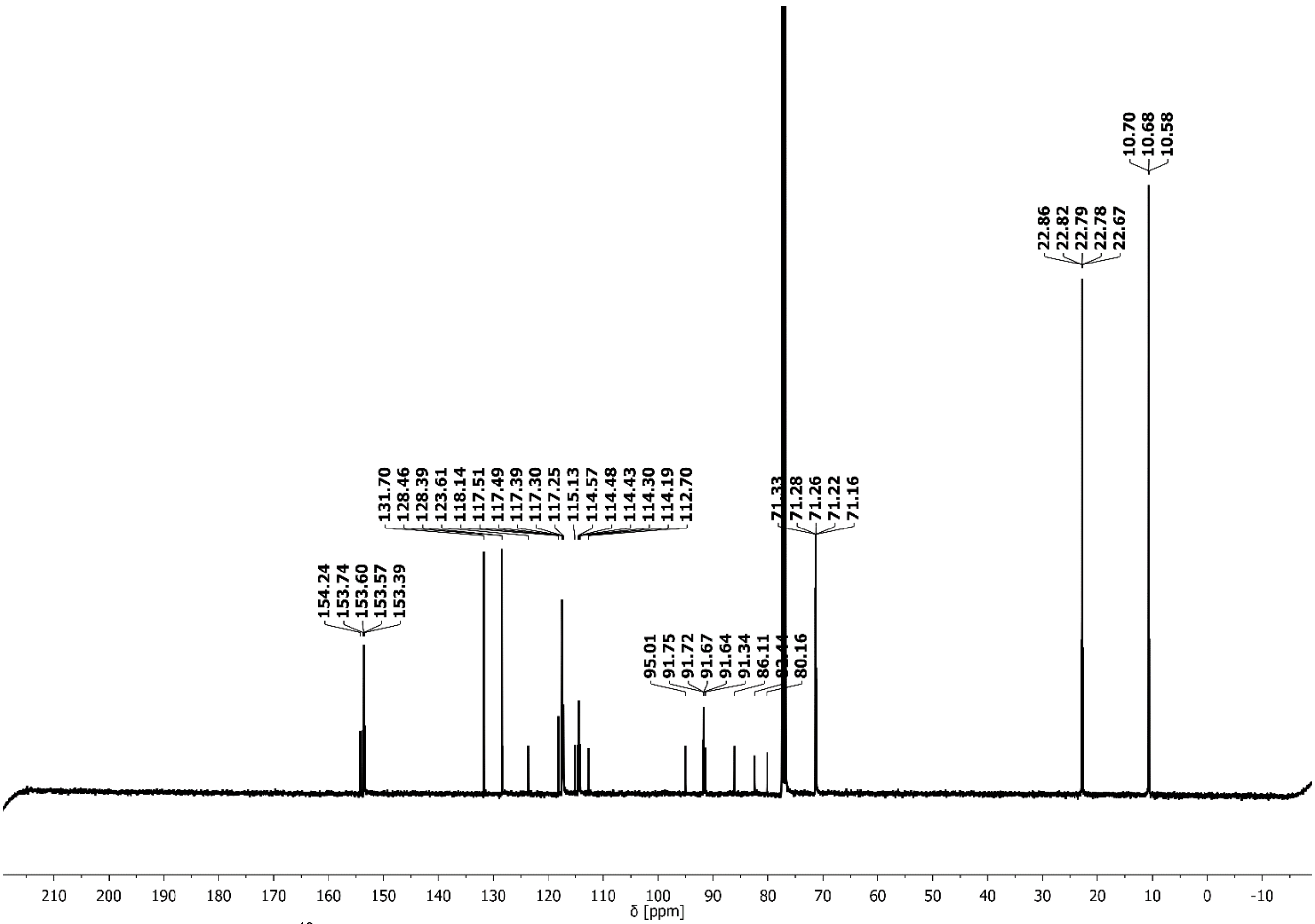

Supplementary Figure 46: ^{13}C NMR spectrum of monodisperse, deprotected tetramer **9**.

Synthesis of trimethyl((4-((4-((4-((4-((4-(phenylethynyl)-2,5-dipropoxyphenyl)ethynyl)-2,5-dipropoxyphenyl)ethynyl)-2,5-dipropoxyphenyl)ethynyl)-2,5-dipropoxyphenyl)ethynyl)-2,5-dipropoxyphenyl)ethynyl)silane **10**

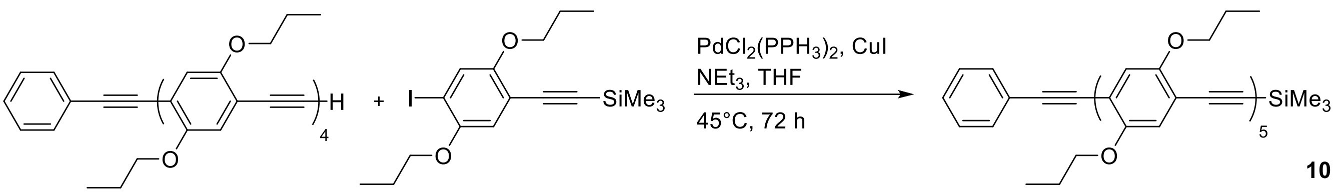

10

1,4-Bis(propyloxy)-2-iodo-5-trimethylsilylacetylenebenzene **1** (968 mg, 2.33 mmol, 5.00 eq.), 5 mol% bis(triphenylphosphine) palladium(II) dichloride (16.3 mg, 23.3 µmol) and 5 mol% copper(I) iodide (4.4 mg, 23.3 µmol) were placed into a Schlenk flask and degassed three times. Under continuous argon flow, 20 mL dry THF and 645 µL dry triethylamine (471 mg, 4.65 mmol, 10.0 eq.) were added and the mixture was stirred for 0.5 h. Subsequently, compound **9** (450 mg, 0.465 mmol, 1.00 eq.) in 15 mL THF was added dropwise with a syringe under continuous argon flow. The reaction mixture was stirred for 72 at 45 °C, taken up in dichloromethane and washed with saturated NH_4Cl solution. The aqueous phase was extracted three times with dichloromethane. The combined organic layers were dried over Na_2SO_4, filtered and concentrated under reduced pressure. The residue was purified by silica column chromatography (cyclohexane / dichloromethane 1:1→1:3) and recrystallisation from cyclohexane / ethyl acetate (10:1) to yield the product as a yellow solid (307 mg, 53%). TLC (dichloromethane / cyclohexane 2:1) R_f = 0.27; ^{1}H NMR ($CDCl_3$, 300 MHz): *δ* (ppm) = 7.58–7.50 (m, 2 H, 2 C$\mathbf{H}_{\mathbf{aromatic}}$C-C≡C), 7.42–7.31 (m, 3 H, 3 C$\mathbf{H}_{\mathbf{aromatic}}$), 7.07–6.91 (m, 10 H, 10 C$\mathbf{H}_{\mathbf{aromatic}}$CO), 4.09–3.79 (m, 20 H, 10 C$\mathbf{H_2}$O), 2.05–1.71 (m, 20 H, 10 C$\mathbf{H}_2$$CH_3$), 1.23–0.88 (m, 30 H, 10 C$\mathbf{H_3}$), 0.26 (s, 9 H, 3 C$\mathbf{H_3}$Si); ^{13}C NMR ($CDCl_3$, 100 MHz): *δ* (ppm) = 154.27, 153.72, 153.59, 153.56, 153.44, 131.68, 128.45, 128.38, 123.60, 117.55, 117.49, 117.37, 117.35, 117.28, 114.70, 114.45, 114.39, 114.17, 113.88, 101.30, 100.24, 95.00, 91.71, 91.55, 86.10, 77.48, 77.16, 76.84, 71.30, 71.25, 71.20, 71.10, 22.84, 22.80, 22.78, 10.67, 10.65, 10.63, 0.07; FAB of $C_{81}H_{94}O_{10}Si$ (M+H$^+$ = 1256.2); IR (ATR) ν = 2960.00, 2934.39, 2870.20, 2148.20, 1594.34, 1511.03, 1463.82, 1423.93, 1383.07, 1270.73, 1247.60, 1204.43, 1062.33, 1040.10, 1012.94, 982.26, 902.26, 860.46, 839.41, 751.72, 685.88, 626.99, 527.44, 468.07 cm^{-1}.

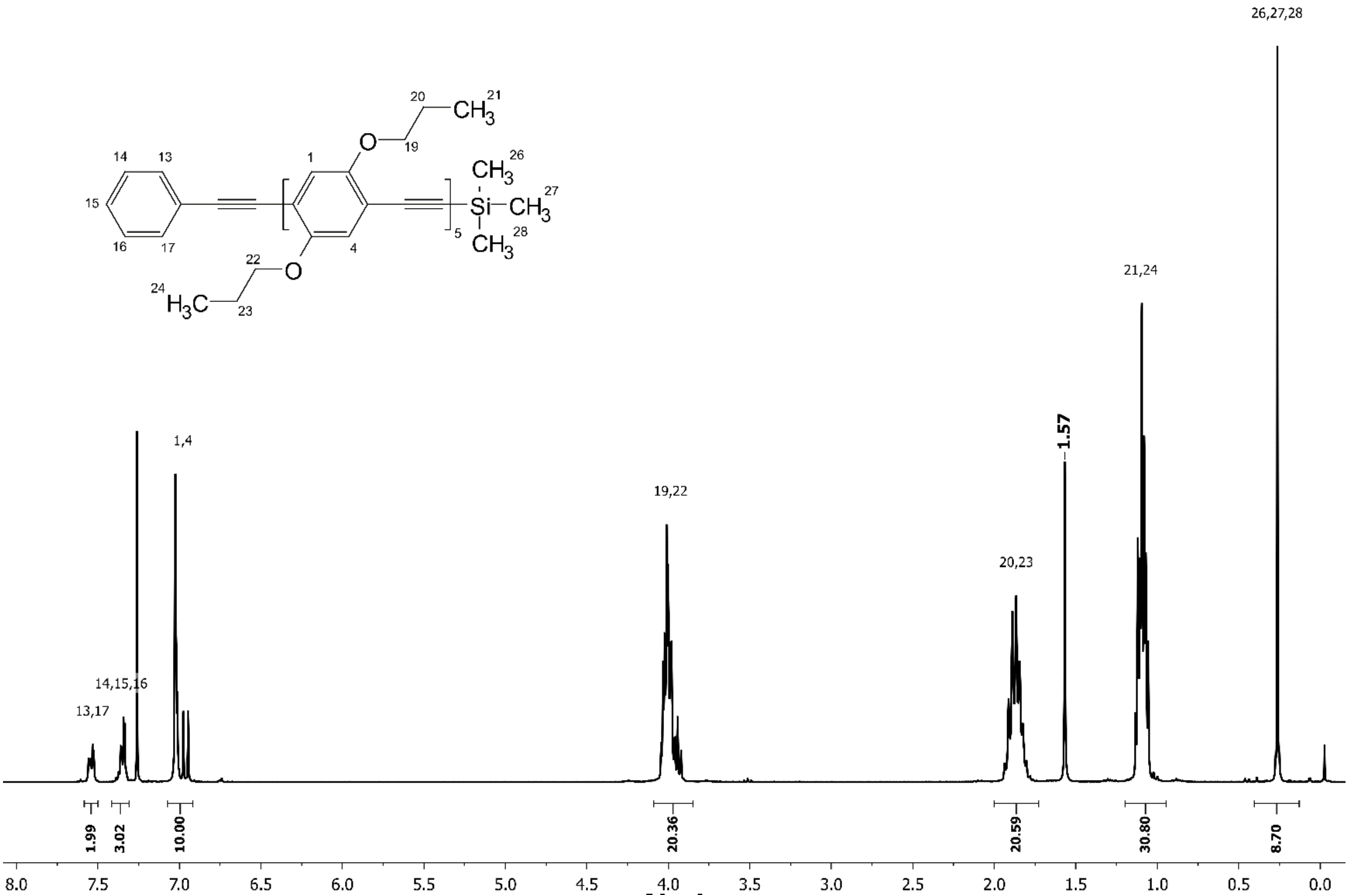

Supplementary Figure 47: ^{1}H NMR spectrum of monodisperse, protected pentamer **10** with assigned signals.

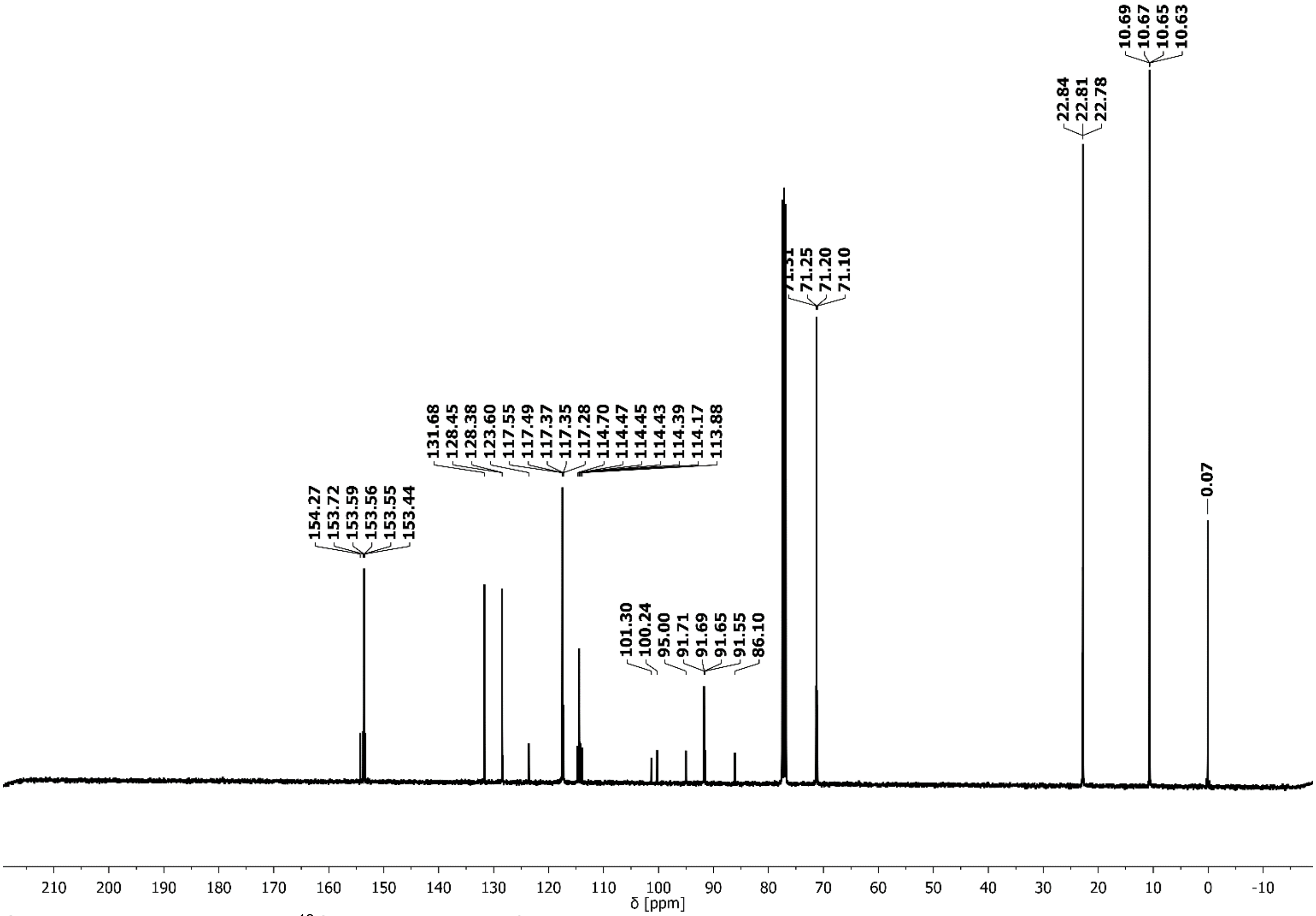

Supplementary Figure 48: ^{13}C NMR spectrum of monodisperse, protected pentamer **10**.

Synthesis of 1-ethynyl-4-((4-((4-((4-((4-(phenylethynyl)-2,5-dipropoxyphenyl)ethynyl)-2,5-dipropoxyphenyl)ethynyl)-2,5-dipropoxyphenyl)ethynyl)-2,5-dipropoxyphenyl)ethynyl)-2,5-dipropoxybenzene **11**

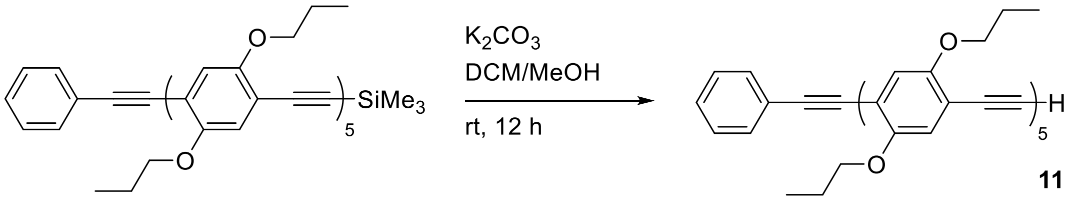

11

Compound **10** (125 mg, 99.5 µmol, 1.00 eq.) and two equivalents of potassium carbonate (27.5 mg, 0.199 mmol) were added to 12 mL dichloromethane and 6 mL methanol. The reaction mixture was degassed with argon and stirred overnight at room temperature and quenched with distilled water. The aqueous phase was extracted three times with dichloromethane, dried over Na_2SO_4, filtered and concentrated under reduced pressure. The residue was purified by silica column chromatography (dichloromethane / cyclohexane 3:1) to yield the product as a yellow solid (116 mg, 98%). TLC (dichloromethane / cyclohexane 2:1) R_f = 0.16; ^{1}H NMR ($CDCl_3$, 300 MHz): δ (ppm) = 7.63–7.47 (m, 2 H, 2 C**H**$_{aromatic}$C-C≡C), 7.43–7.32 (m, 3 H, 3 C**H**$_{aromatic}$), 7.11–6.91 (m, 10 H, 10 C**H**$_{aromatic}$CO), 4.15–3.87 (m, 20 H, 10 C**H**$_2$O), 3.35 (s, 1 H, 1 C≡C-**H**), 2.01-1.75 (m, 20 H, 10 C**H**$_2$CH$_3$), 1.19–0.92 (m, 30 H, 10 C**H**$_3$); ^{13}C NMR ($CDCl_3$, 100 MHz): δ (ppm) = 154.23, 153.74, 153.60, 153.58, 153.39, 131.69, 128.46, 128.38, 123.61, 118.15, 117.51, 117.39, 117.31, 117.25, 115.13, 114.58, 114.49, 114.44, 114.31, 114.19, 112.71, 95.01, 91.72, 91.67, 91.34, 86.11, 82.44, 80.16, 71.32, 71.27, 71.22, 71.16, 22.85, 22.79, 22.66, 10.67, 10.57; FAB of $C_{78}H_{86}O_{10}$ (M+H$^+$ = 1184.3); IR (ATR) v = 3288.46, 2959.22, 2933.08, 2873.56, 1595.35, 1511.17, 1470.04, 1424.66, 1385.03, 1273.01, 1204.34, 1104.57, 1062.42, 1039.80, 1019.72, 979.46, 905.22, 860.98, 768.48, 754.29, 688.02, 648.90, 527.53, 464.63 cm^{-1}.

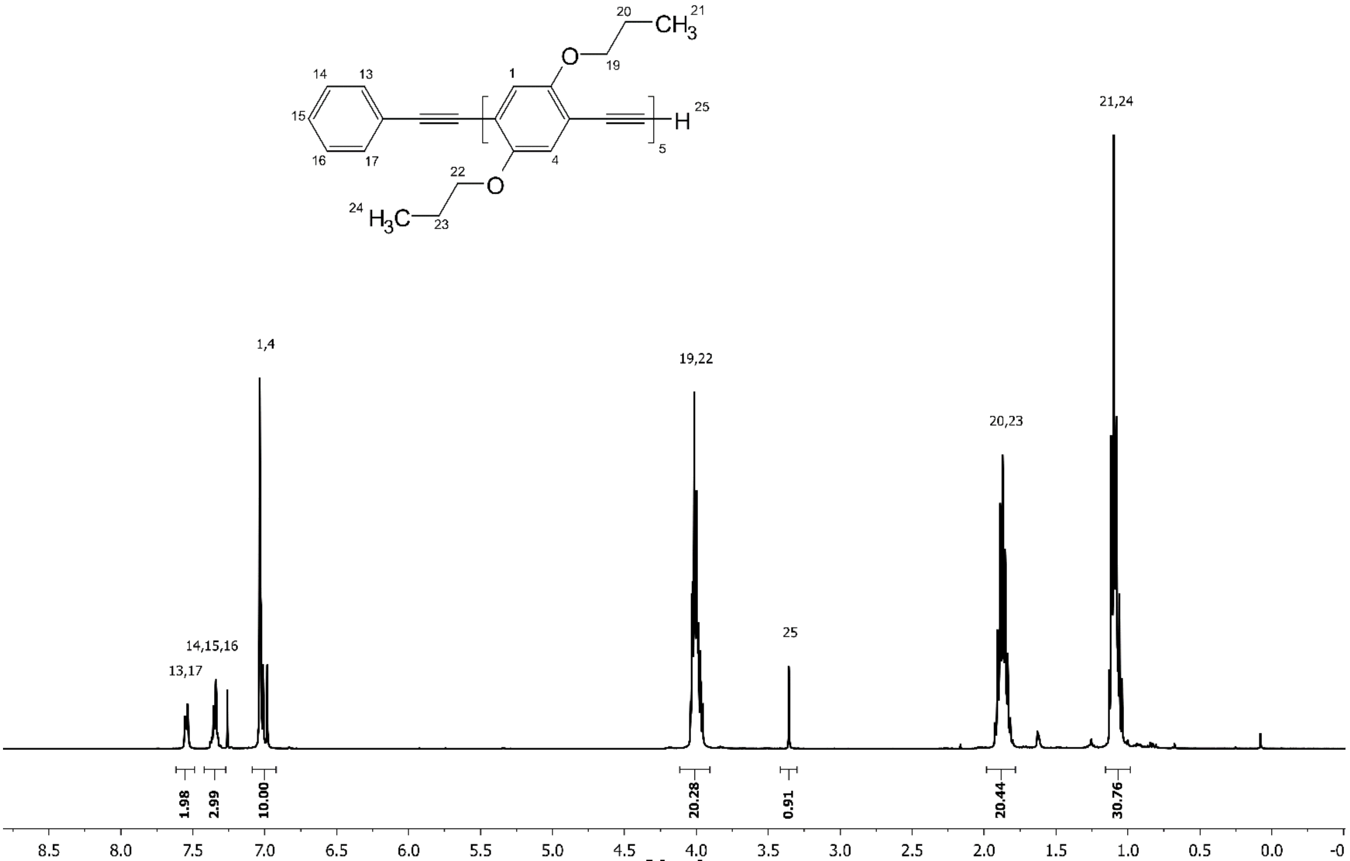

Supplementary Figure 49: ^{1}H NMR spectrum of monodisperse, deprotected pentamer **11** with assigned signals.

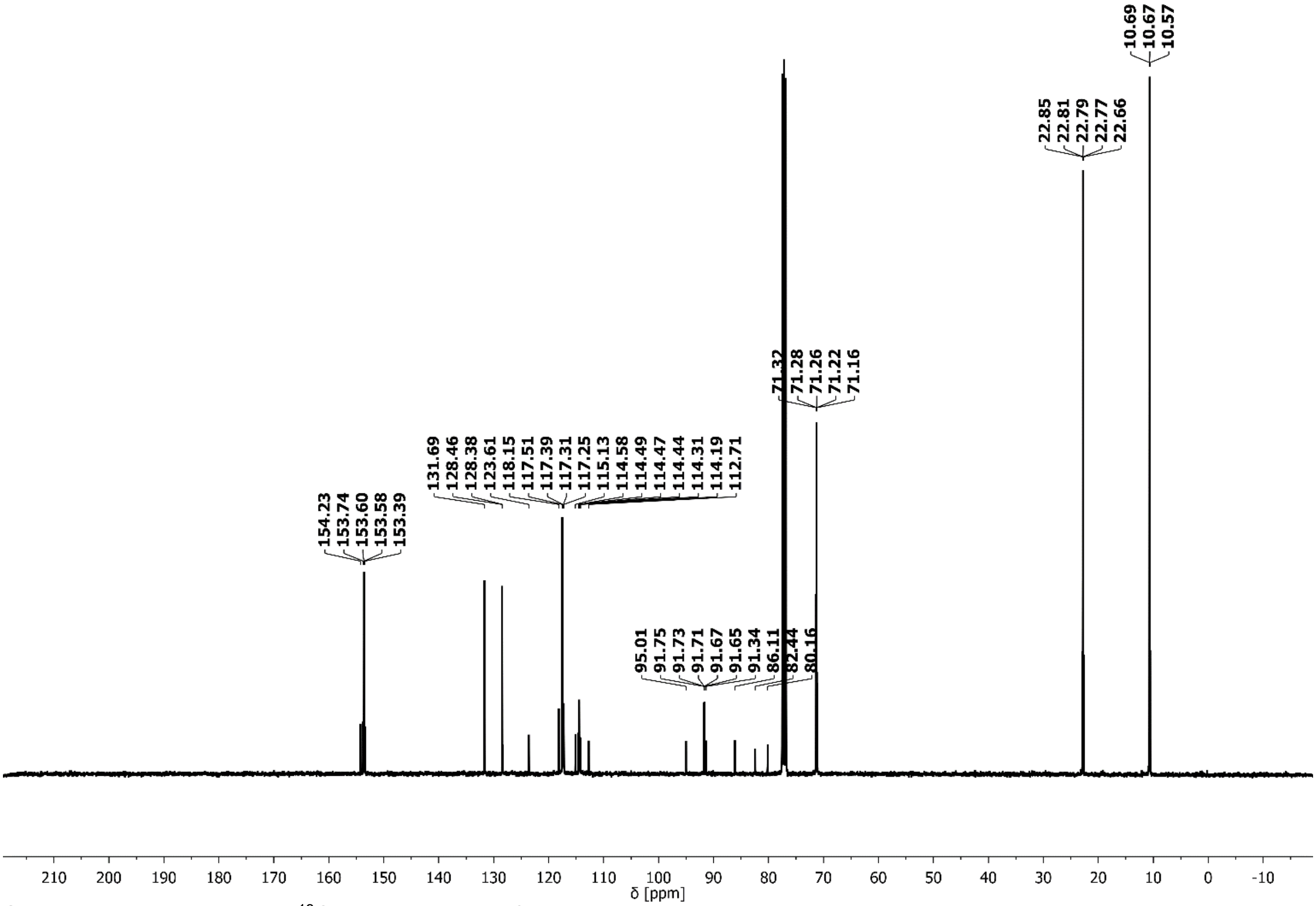

Supplementary Figure 50: ^{13}C NMR spectrum of monodisperse, deprotected pentamer **11**.

1.5 Solid Phase Organic Synthesis (SPOS) Approaches

The initial SPOS procedure was adapted from the solution approach. DMF was used as solvent and the deprotection was performed with tetra-*n*-butylammonium fluoride (TBAF) as depicted in Supplementary Figure 51.

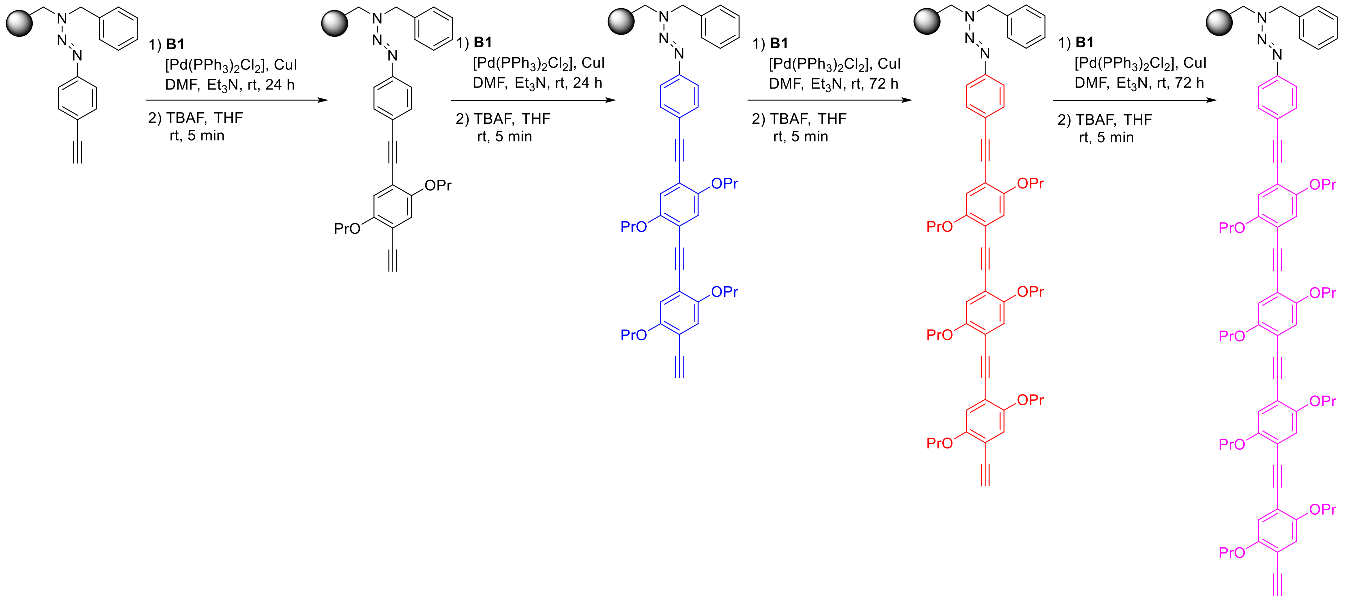

Supplementary Figure 51: Planned synthesis procedure towards the monodisperse tetramer via SPOS.

Since the tetramer was not obtained after cleavage, further attempts were not performed using these conditions. Further investigation revealed that the bis(triphenylphosphine)palladium(II)dichloride catalyst is not suitable for SPOS. Therefore, further optimisation approaches were performed with the palladium triphenylphosphine catalyst.

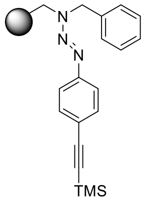

(E)-3-Benzyl-3-M-1-(4-((trimethylsilyl)ethynyl)phenyl)triaz-1-ene* (**I1**): Under argon atmosphere, 84.6 mg $Pd(PPh_3)_4$ (73.0 µmol, 12.0 mol%), 81.4 mg copper(I)iodide (0.427 mmol, 0.700 eq.), 800 mg (*E*)-3-benzyl-3-M*-1-(4-iodophenyl)triaz-1-ene (0.610 mmol, 0.763 mmol/g, 1.00 eq.) were placed in a 20 mL vial with 6 mL abs. DMF and 0.135 mL abs. triethylamine (0.976 mmol, 1.60 eq.). The mixture was swollen for 5 minutes, subsequently, 261 µL trimethylsilylacetylene (1.83 mmol, 3.00 eq.) were added and the mixture was shaken for 16 h at room temperature. The resin is filtered and was washed with25 mL DMF, cupral solution in DMF, DMF, MeOH, DMF, H_2O, MeOH, THF, MeOH, DMF, MeOH, dichloromethane (3×) and dried overnight at 70 °C. The product was obtained as dark brown resin. IR (ATR) ν = 3025, 2920, 1681, 1601, 1492, 1398, 1346, 1248, 1145, 1073, 1028, 905, 839, 755, 695, 537, 400 cm^{-1}. – *Load of the resin:* 0.701 mmol/g.

(E)-3-Benzyl-3-M-1-(4-ethynylphenyl)triaz-1-ene* (**I2**): 500 mg (*E*)-3-Benzyl-3-M*-1-(4-((trimethylsilyl)ethynyl)phenyl)-triaz-1-ene (**I1**, 0.351 mmol, 0.701 mmol/g, 1.00 eq.) were swollen in THF in a 10 mL filter syringe. Subsequently, 596 µL 1 M TBAF solution in THF (0.596 mmol, 1.70 eq.) were added and the mixture was shaken 1 h at room temperature. The resin was filtered and washed with 15 mL MeOH, DMF, H_2O, MeOH, THF, MeOH, DMF, MeOH, dichloromethane (3×) and dried overnight at 70 °C. The product was obtained as a dark brown resin. IR (ATR) v = 3025, 2917, 1601, 1492, 1449, 1398, 1320, 1144, 1075, 1028, 904, 841, 755, 695, 538, 415, 383 cm^{-1}. – *Load of the resin:* 0.738 mmol/g.

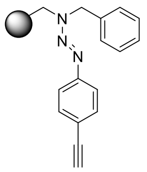

(E)-3-Benzyl-3-M-1-(4-((2,5-dipropoxy-4-((trimethylsilyl)ethynyl)phenyl)ethynyl)phenyl)-triaz-1-ene* (**I3**): Under argon atmosphere, 30.7 mg $Pd(PPh_3)_4$ (26.6 µmol, 12.0 mol%), 29.5 mg copper(I)iodide (0.155 mmol, 0.700 eq.), 300 mg (*E*)-3-benzyl-3-M*-1-(4-ethynylphenyl)triaz-1-ene (**I2**, 0.221 mmol, 0.738 mmol/g, 1.00 eq.), 277 mg **1** (0.664 mmol, 3.00 eq.) were suspended in 4 mL DMF und 153 µL abs. triethylamine (1.11 mmol, 5.00 eq.). The reaction mixture was shaken 24 h at room temperature. Subsequently, the resin was filtered and washed with 25 mL DMF, cupral solution. in DMF, DMF, MeOH, DMF, H_2O, MeOH, THF, MeOH, DMF, MeOH, dichloromethane (3×) and dried overnight at 70 °C. The product was obtained as orange resin. IR (ATR) v = 3025, 2919, 1601, 1492, 1450, 1248, 1200, 1145, 1064, 1028, 980, 839, 755, 695, 540, 455, 393 cm^{-1}. – *Load of the resin:* 0.608 mmol/g.

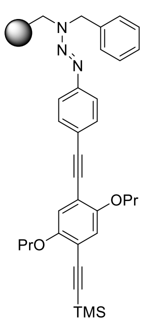

(E)-3-benzyl-3-M-1-(4-((4-ethynyl-2,5-dipropoxyphenyl)ethynyl)phenyl)triaz-1-ene* (**I4**): 200 mg **I4** (0.122 mmol, 0.608 mmol/g, 1.00 eq.) were swollen in 4 mL THF for 5 minutes in a filter syringe. Subsequently, 365 µL 1 M TBAF solution in THF (0.365 mmol, 1.70 eq.) were added and the mixture was shaken for 30 minutes at room temperature. The resin was filtered and washed with 10 mL MeOH, DMF, H_2O, MeOH, THF, MeOH, DMF, MeOH, dichloromethane (3×) and dried overnight at 70 °C. The product was obtained as light brown resin. IR (ATR) v = 3024, 2921, 1601, 1492, 1449, 1378, 1273, 1213, 1143, 1063, 1028, 981, 905, 840, 752, 695, 537, 431, 418, 391 cm^{-1}. – *Load of the resin:* 0.637 mmol/g.

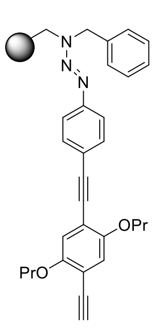

1-((4-azidophenyl)ethynyl)-4-ethynyl-2,5-dipropoxybenzene (**12**): 50.0 mg **I4** (31.8 µmol, 0.637 mmol/g, 1.00 eq.) was swollen in 1 mL dichloromethane. 42.2 µL Trimethylsilyl azide (0.318 mmol, 10.0 eq.) and 24.5 µL TFA (0.318 mmol, 10.0 eq.) were added and shaken for1 h at room temperature. The solid was filtered and washed with dichloromethane several times. Subsequently, the solvent of the filtrate was evaporated under reduced pressure and the crude product was purified by silica column chromatography (cyclohexane / dichloromethane 1:1). The product was obtained as yellow solid (6.80 mg, 18.9 µmol, 60% over five steps, starting from **I1**). TLC (cyclohexane / dichloromethane 1:1) R_f = 0.45; ^1H NMR (300 MHz, $CDCl_3$): δ (ppm) = 7.53-7.48 (m, 2 H, 2 C**H**$_{\mathbf{aromatic}}$), 7.03-6.99 (m, 2 H, 2 C**H**$_{\mathbf{aromatic}}$-C-N),6.98 (s, 2 H,

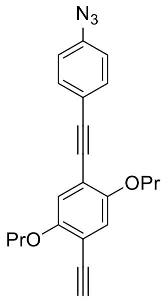

2 C**H**$_{aromatic}$CO), 3.99-3.95 (m, 4 H, 2 C**H$_2$**O), 3.55 (s, 1 H, 1 C≡C**H**), 1.90-1.80 (m, 4 H, 2 C**H$_2$**CH_3), 1.11-1.04 (m, 6 H, 2 C**H$_3$**); ^{13}C NMR (100 MHz, $CDCl_3$): δ (ppm) = 154.3, 153.6, 140.2, 133.2, 120.0, 119.2, 118.0, 117.0, 114.6, 112.8, 94.4, 86.2, 82.5, 80.1, 71.2, 22.7, 10.6; HRMS (FAB) of $C_{22}H_{21}O_2N_3$ [M+H$^+$] calc. 359.1634, found 359.1635; IR (ATR) ν = 3282, 2959, 2934, 2875, 2103, 1596, 1509, 1497, 1412, 1391, 1278, 1219, 1129, 1046, 1029, 973, 850, 831, 682, 664, 601, 556, 529 cm^{-1}.

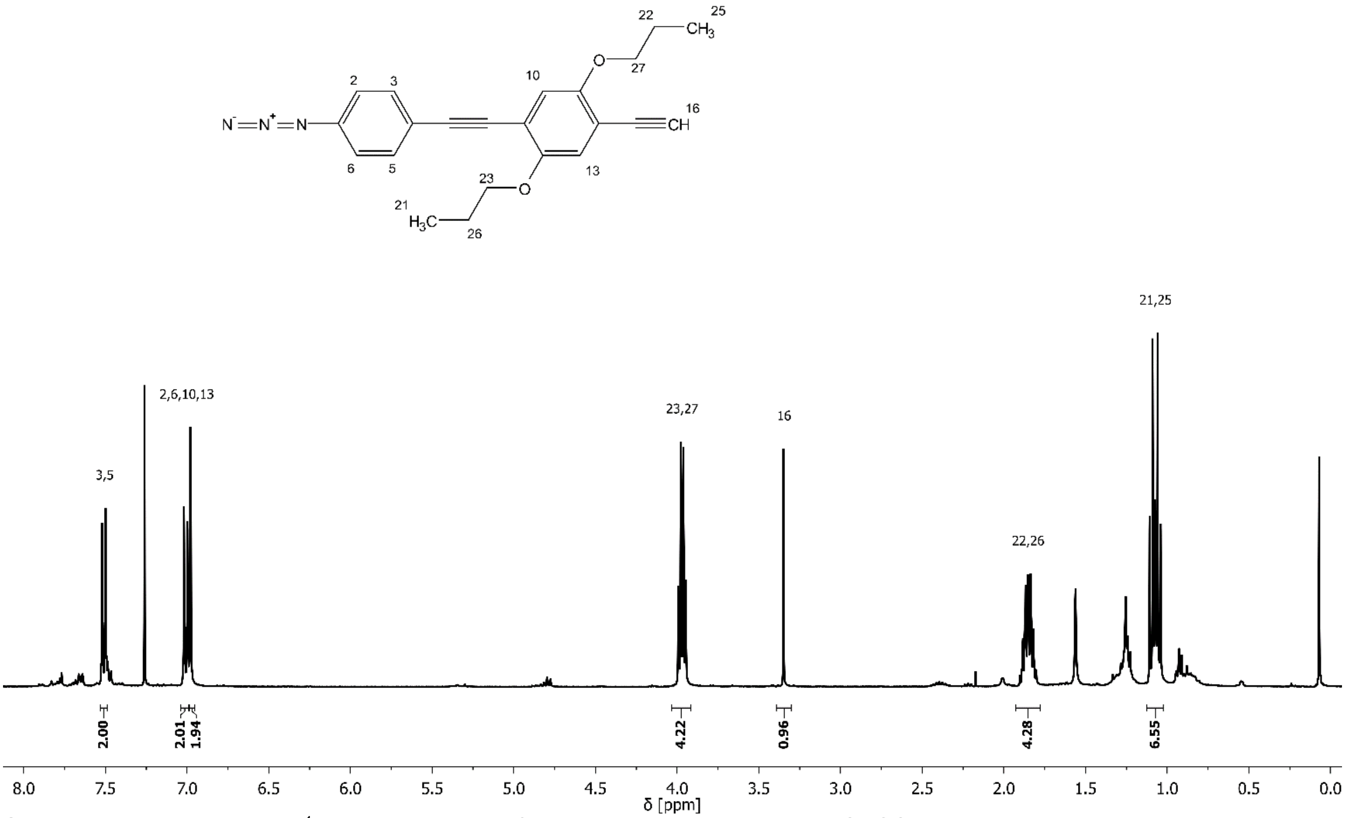

Supplementary Figure 52: ^{1}H NMR spectrum of the monomer **12** obtained by SPOS with assigned signals.

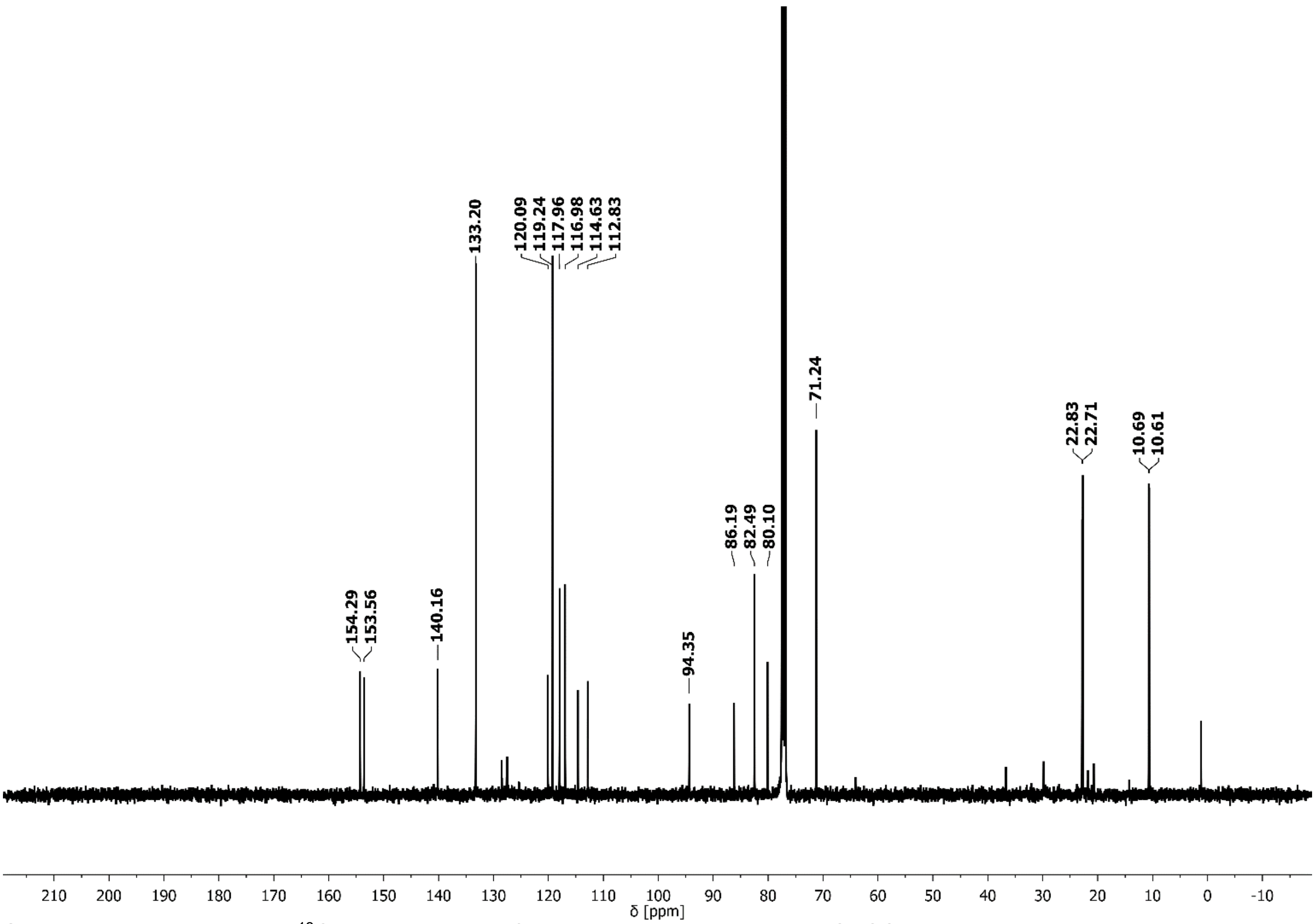

Supplementary Figure 53: ^{13}C NMR spectrum of the monomer **12** obtained by SPOS.

1.6 Oligomerization Approach

Synthesis of 1-ethynyl-4-iodo-2,5-dipropoxybenzene **13**

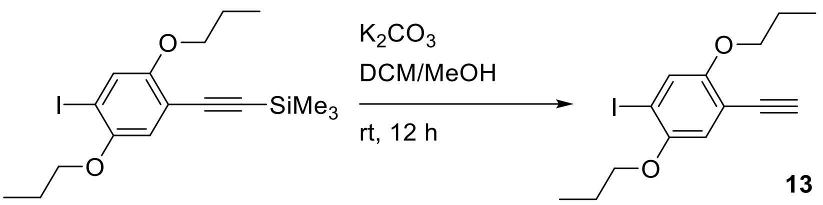

1,4-Bis(propyloxy)-2-iodo-5-trimethylsilylacetylenebenzene (1.00 g, 2.40 mmol, 1.00 eq.) and two equivalents of potassium carbonate (1.65 g, 4.81 mmol) were added to 50 mL dichloromethane and 50 mL methanol. The reaction mixture was degassed with argon and stirred overnight at room temperature and quenched with distilled water. The aqueous phase was extracted three times with dichloromethane, dried over Na_2SO_4, filtered and concentrated under reduced pressure to yield the product as a yellow solid (818 mg, 99%). TLC (cyclohexane / dichloromethane 4:1) R_f = 0.43; ^{1}H NMR ($CDCl_3$, 300 MHz): δ (ppm) = 7.29, 6.87 (2 s, 2 H, 2 C**H**$_{aromatic}$), 3.92 (dt, *J* = 8.6, 6.5 Hz, 4 H, 2 C**H$_2$**O), 3.30 (s, 1 H, 1 C≡C-**H**), 1.95–1.73 (m, 4 H, 2 C**H$_2$**CH_3), 1.07, 1.06 (2 t, *J* = 7.6 Hz, 6 H, 2 C**H$_3$**); ^{13}C NMR ($CDCl_3$, 75 MHz): δ (ppm) = 154.91, 151.78, 123.97, 116.82, 112.42, 88.42, 81.91, 79.76, 71.66, 71.43, 22.68, 22.64, 10.81, 10.54; FAB of $C_{14}H_{17}IO_2$ (M+H$^+$ = 345.1); HRMS (FAB) of $C_{14}H_{17}IO_2$ [M+H$^+$] calc. 344.0268, found 344.0266; IR (ATR) v = 3267.6, 2953.8, 2907.9, 2870.5, 1587.3, 1485.3, 1456.5, 1367.5, 1264.8, 1208.8, 1147.4, 1011.4, 907.2, 857.6, 818.7, 767.0, 722.2, 685.8, 658.7, 643.0, 441.9, 416.8 cm^{-1}.

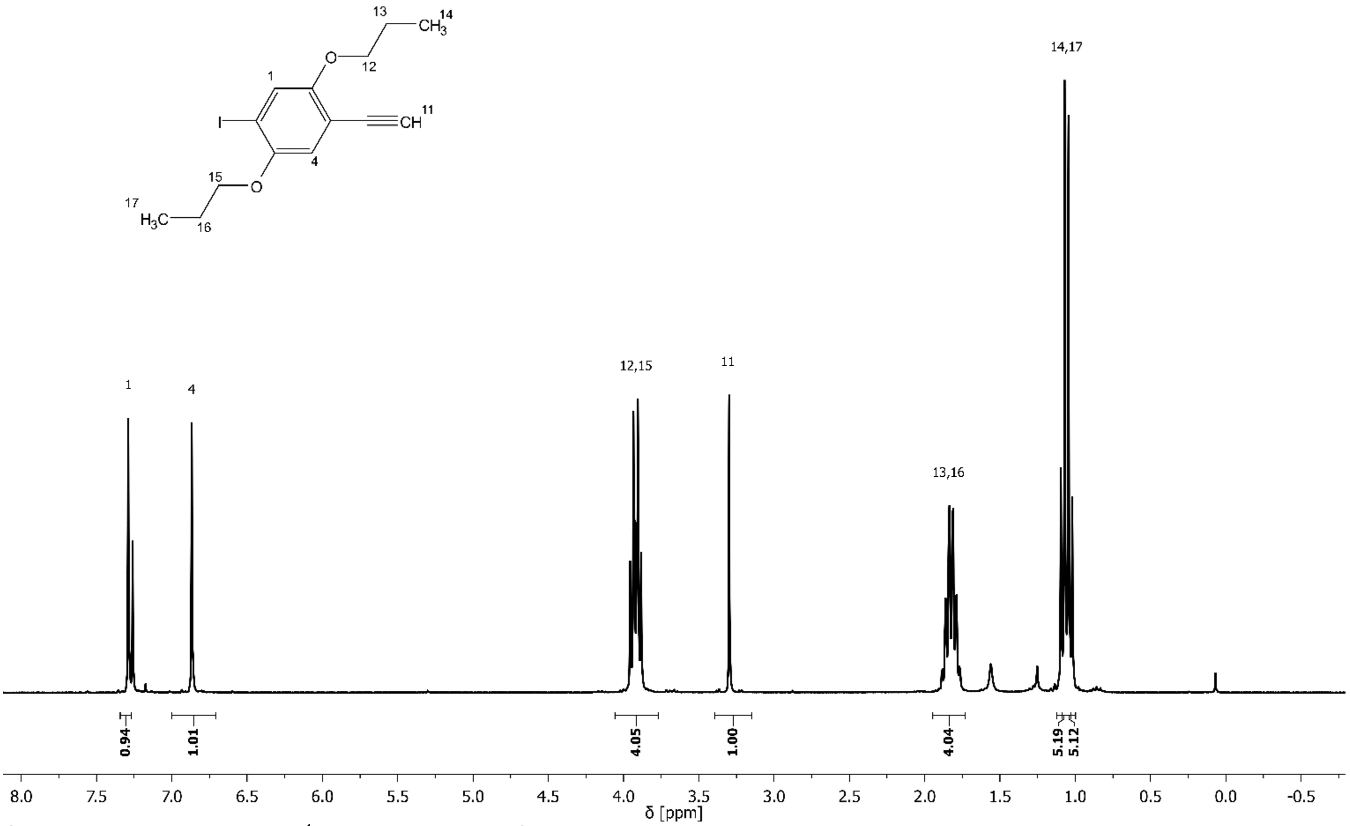

Supplementary Figure 54: ^{1}H NMR spectrum of the deprotected building block **13** with assigned signals.

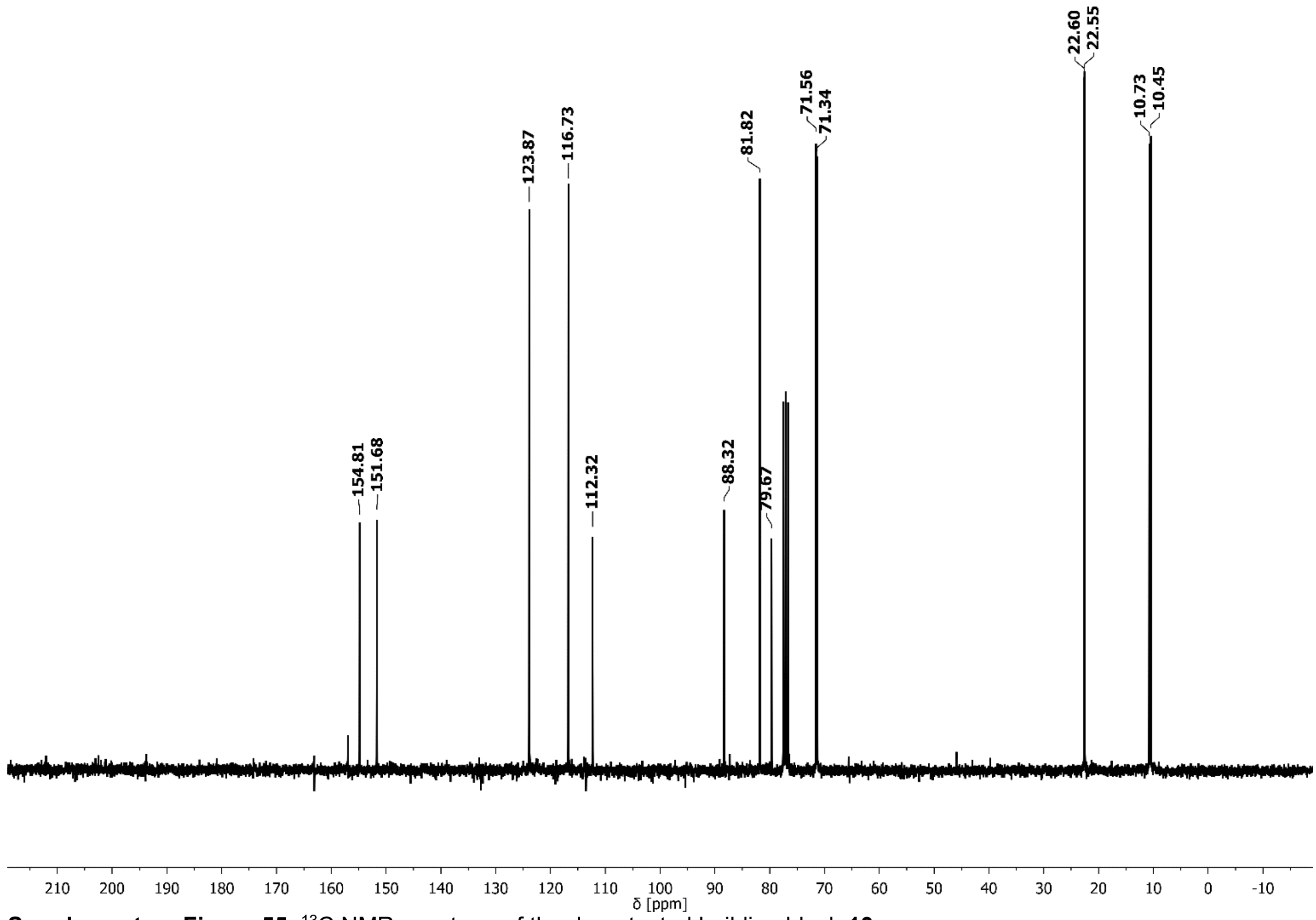

Supplementary Figure 55: ^{13}C NMR spectrum of the deprotected building block **13**.

The direct oligomerization approach

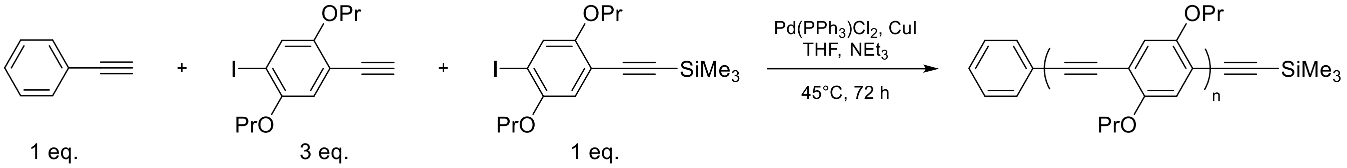

Phenylacetylene (106 µL, 98.9 mg, 0.968 mmol, 1.00 eq.), deprotected building block **13** (1.00 g, 2.91 mmol, 3.00 eq.) and building block **1** (403 mg, 0.968 mmol, 1.00 eq.) were introduced in a Sonogashira reaction with 5 mol% bis(triphenylphosphine) palladium(II)dichloride (34.0 mg, 48.4 µmol) and 5 mol% copper(I)iodide (9.2 mg, 48.4 µmol). They were placed into a Schlenk flask and degassed. Under continuous argon flow, 50 mL dry THF and 0.88 mL dry triethylamine (650 mg, 6.40 mmol, 10.0 eq.) were added and the mixture was stirred for 72 h at 45 °C. The reaction mixture was taken up in dichloromethane and washed with saturated NH_4Cl solution. The aqueous phase was extracted three times with dichloromethane. The combined organic layers were dried over Na_2SO_4, filtered and concentrated under reduced pressure. The crude product was analysed by SEC (compare Figure 2, b).

1.7 Synthesis of the Sequence-Defined Pentamer

Synthesis of ((2,5-diisopropoxy-4-((4-(phenylethynyl)-2,5-dipropoxyphenyl)ethynyl)phenyl)ethynyl) trimethylsilane **14**

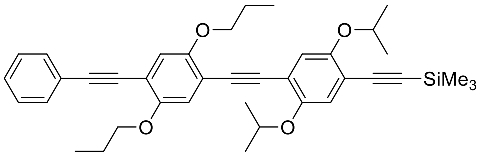

1,4-Bis(isopropyloxy)-2-iodo-5-trimethylsilylacetylenebenzene (5.88 g, 14.1 mmol, 3.00 eq.), 5 mol% bis(triphenylphosphine) palladium(II) dichloride (165 mg, 0.236 mmol) and 5 mol% copper(I) iodide (44.9 mg, 0.236 mmol) were placed into a Schlenk flask and degassed three times. Under continuous argon flow, 100 mL dry THF and 6.57 mL (4.77 g, 47.1 mmol, 10.0 eq.) dry triethylamine were added and the mixture was stirred for 10 minutes. Subsequently, 1-ethynyl-4-(phenylethynyl)-2,5-dipropoxybenzene **3** (1.50 g, 4.71 mmol, 1.00 eq.) in 50 mL THF was added dropwise with a syringe. The reaction mixture was stirred for 72 h at 45 °C, taken up in dichloromethane and washed with saturated NH_4Cl solution. The aqueous phase was extracted three times with dichloromethane. The combined organic layers were dried over Na_2SO_4, filtered and concentrated under reduced pressure. The residue was purified by silica column chromatography (cyclohexane / dichloromethane 3:1 → 3:2) and a flash silica column (cyclohexane / ethyl acetate 20:1) to yield the product as a yellow solid (1.84 g, 64%). TLC (cyclohexane / dichloromethane 2:1) R_f = 0.31; ^{1}H NMR ($CDCl_3$, 300 MHz): δ (ppm) =7.61–7.46 (m, 2 H, 2 C**H**$_{\mathbf{aromatic}}$C-C≡C), 7.43–7.29 (m, 3 H, 3 C**H**$_{\mathbf{aromatic}}$), 7.08–6.91 (m, 4 H, 4 C**H**$_{\mathbf{aromatic}}$CO),

4.50 (dhept, *J* = 18.4, 6.2 Hz, 2 H, 2 C**H(**$CH_3)_2$), 4.00 (dt, *J* = 6.5, 4.7 Hz, 4 H, 2 C**H₂**O), 1.86 (dp, *J* = 6.9, 1.9 Hz, 4 H, 2 C$\mathbf{H_2}$$CH_3$), 1.36 (dd, *J* = 7.1, 6.0 Hz, 12 H, 4 C$\mathbf{H_3}$CH), 1.10, 1.08 (2 t, *J* = 7.2 Hz, 6 H, 2 C$\mathbf{H_3}$$CH_2$), 0.26 (s, 9 H, 3 C$\mathbf{H_3}$Si); ^{13}C NMR ($CDCl_3$, 75 MHz): *δ* (ppm) = 153.72, 153.61, 152.94, 131.68, 128.44, 123.60, 121.87, 121.10, 117.45, 117.26, 116.58, 116.15, 114.25, 101.62, 100.05, 95.00, 91.66, 91.40, 86.09, 73.54, 73.13, 71.26, 22.84, 22.34, 10.71, 0.07; FAB of $C_{39}H_{46}O_4Si$ ($M+H^+$ = 607.3); HRMS (FAB) of $C_{39}H_{46}O_4Si$ [$M+H^+$] calc. 606.3165, found 606.3164; IR (ATR) ν = 2965.3, 2932.5, 2875.7, 2150.2, 1595.6, 1505.1, 1488.3, 1418.1, 1382.8, 1330.7, 1271.0, 1249.1, 1204.2, 1137.2, 1105.3, 1060.6, 1010.0, 962.1, 889.0, 839.6, 756.8, 690.7, 637.6, 527.7, 466.2 cm^{-1}.

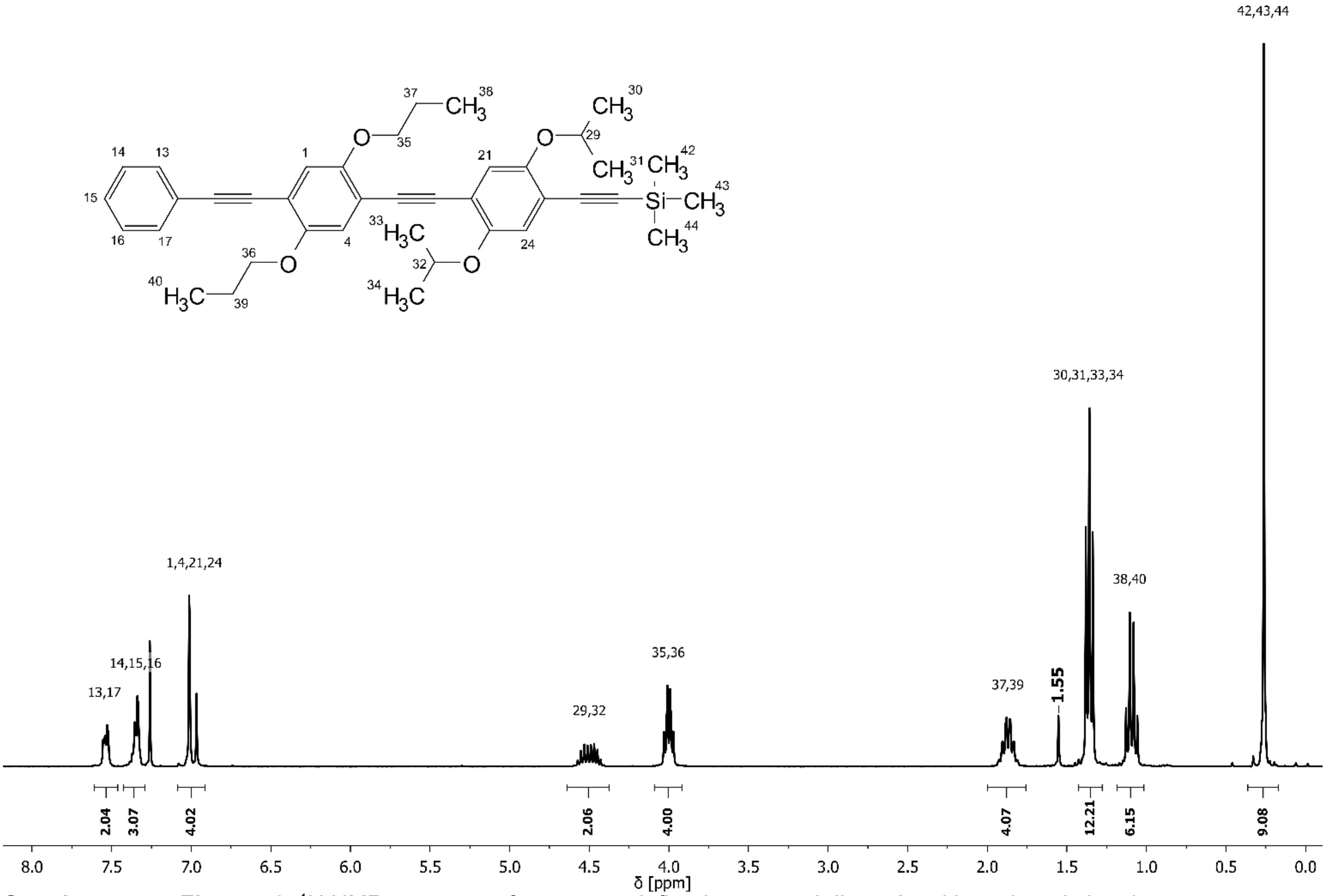

Supplementary Figure 56: ^{1}H NMR spectrum of sequence-defined, protected dimer **14** with assigned signals.

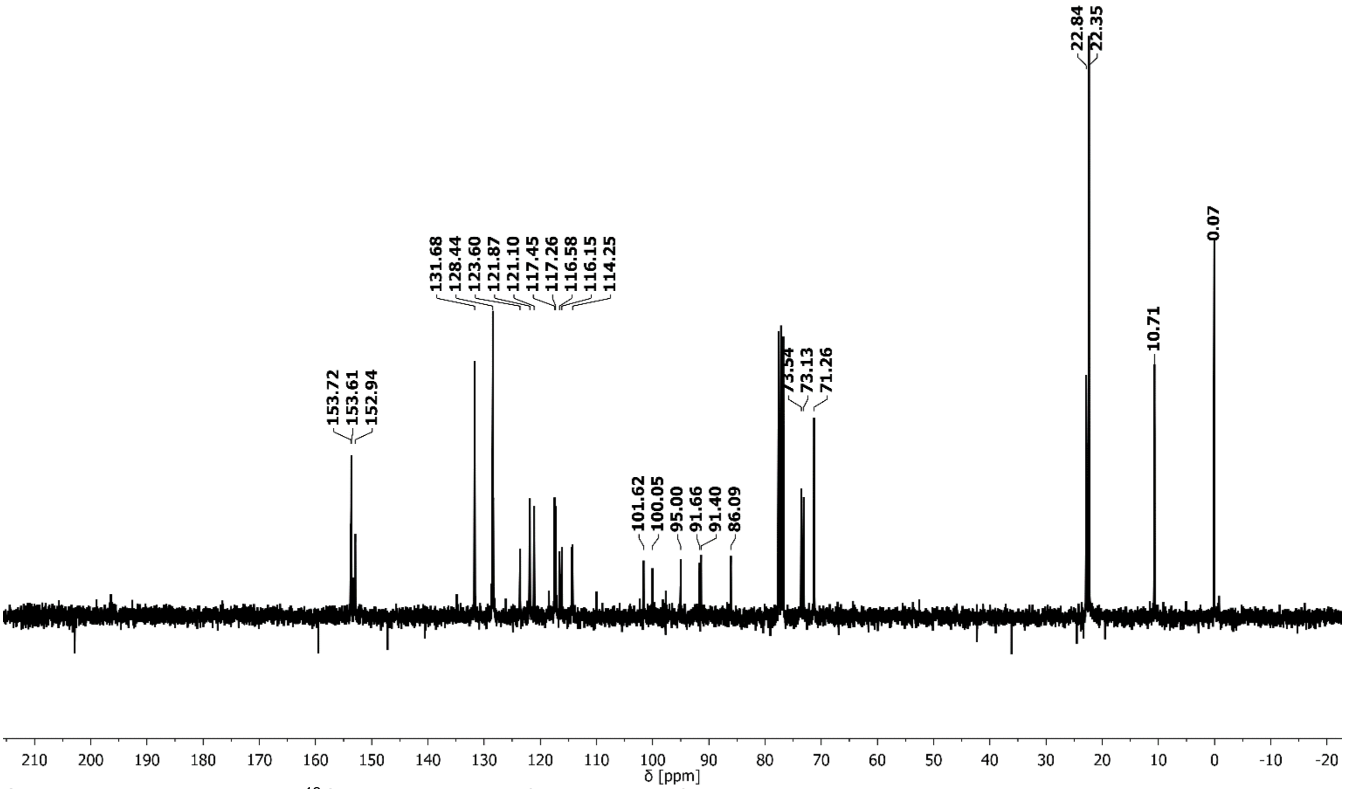

Supplementary Figure 57: ^{13}C NMR spectrum of sequence-defined, protected dimer **14**.

Synthesis of 1-ethynyl-2,5-diisopropoxy-4-((4-(phenylethynyl)-2,5-dipropoxyphenyl)ethynyl)benzene **15**

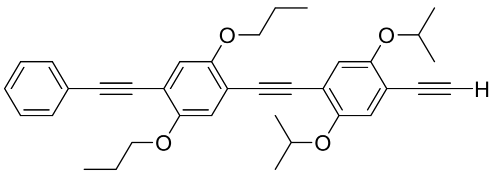

Compound **14** (800 mg, 1.32 mmol, 1.00 eq.) and two equivalents of potassium carbonate (365 mg, 2.64 mmol) were placed in a Schlenk flask and degassed three times. Under continuous argon flow 40 mL dichloromethane and 40 mL methanol were added. The reaction mixture was stirred overnight at room temperature under argon atmosphere and quenched with distilled water. The aqueous phase was extracted three times with dichloromethane, dried over Na_2SO_4, filtered and concentrated under reduced pressure. The residue was purified by silica column chromatography (cyclohexane / ethyl acetate 20:1) to yield the product as an orange solid (688 mg, 98%). TLC (cyclohexane / dichloromethane 2:1) R_f = 0.28; ^{1}H NMR ($CDCl_3$, 300 MHz): δ (ppm) = 7.60–7.46 (m, 2 H, 2 C**H**$_{aromatic}$C-C≡C), 7.46–7.30 (m, 3 H, 3 C**H**$_{aromatic}$), 7.11–6.91 (m, 4 H, 4 C**H**$_{aromatic}$CO), 4.66–4.41 (m, 2 H, 2 C**H(**$CH_3)_2$)), 4.11–3.90 (m, 4 H, 2 C**H₂**O), 3.32 (s, 1 H, 1 C≡C-**H**), 2.00–1.77 (m, 4 H, 4 C**H**$_2$$CH_3$), 1.37 (dd, *J* = 6.1, 2.2 Hz, 12 H, 4 C**H**$_3$CH), 1.23–0.97 (m,6 H, 2 C**H**$_3$$CH_2$), ^{13}C NMR ($CDCl_3$, 75 MHz): δ (ppm) = 153.47, 153.37, 153.30, 152.30, 131.35, 128.26, 128.19, 123.34, 121.30, 120.13, 117.05, 116.87, 116.61, 114.34, 114.05, 113.98, 94.86, 91.42, 91.35, 86.04, 82.51, 80.11, 72.78, 72.54, 70.85, 70.82, 22.58, 22.55, 22.03, 21.94, 10.47, 10.42, FAB of $C_{36}H_{38}O_4$ (M+H$^+$ = 535.3); HRMS (FAB) of $C_{36}H_{38}O_4$ [M+H$^+$] calc. 534.2770, found 534.2771; IR (ATR) ν = 3285.7, 2970.9, 2932.2, 2874.6, 1596.1, 1505.2, 1487.3, 1417.5, 1383.0, 1330.5, 1271.3, 1203.5, 1135.2, 1105.6, 1060.5, 1011.7, 957.6, 862.6, 754.6, 689.4, 664.0, 611.7, 527.8, 459.1 cm^{-1}.

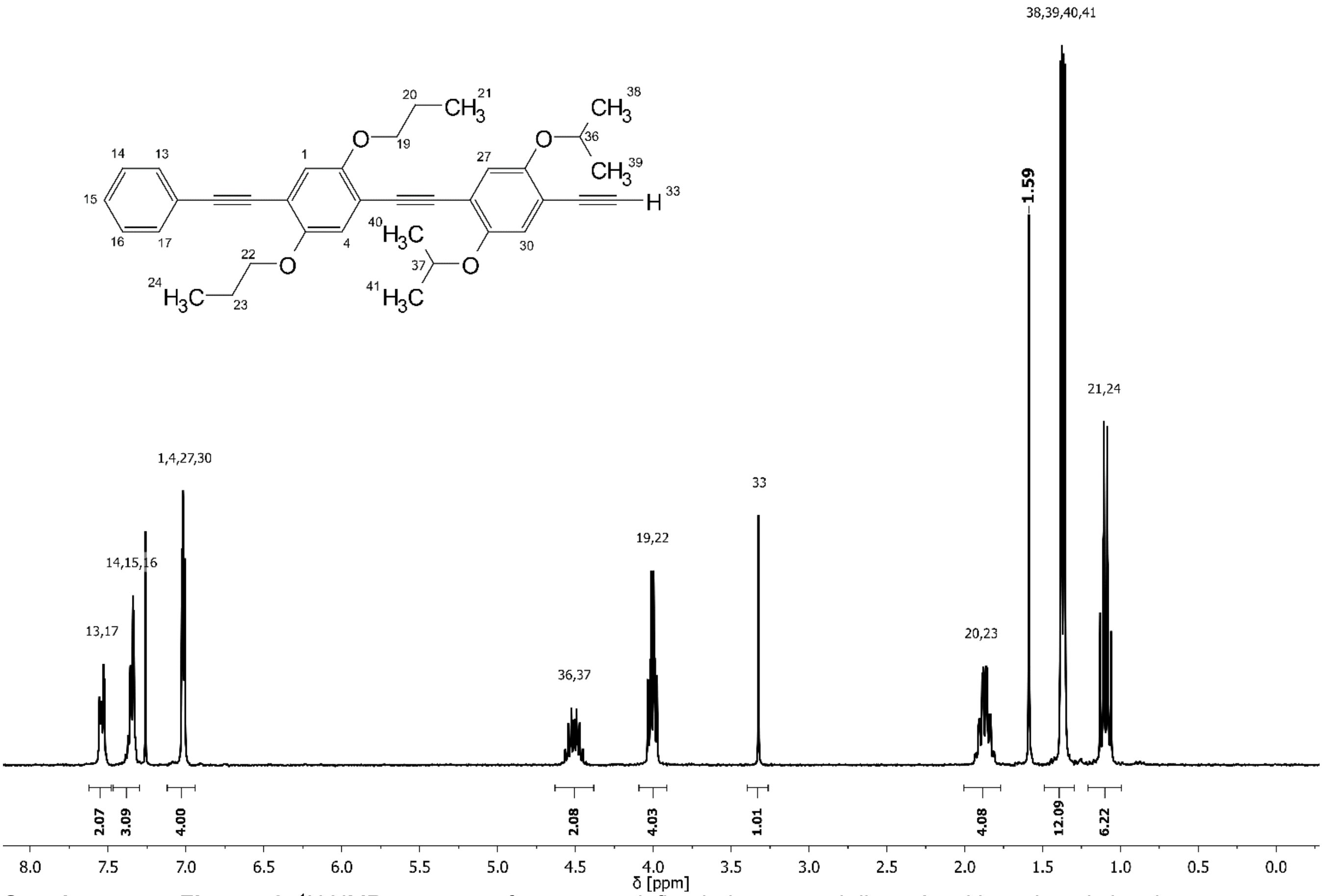

Supplementary Figure 58: ^{1}H NMR spectrum of sequence-defined, deprotected dimer **15** with assigned signals.

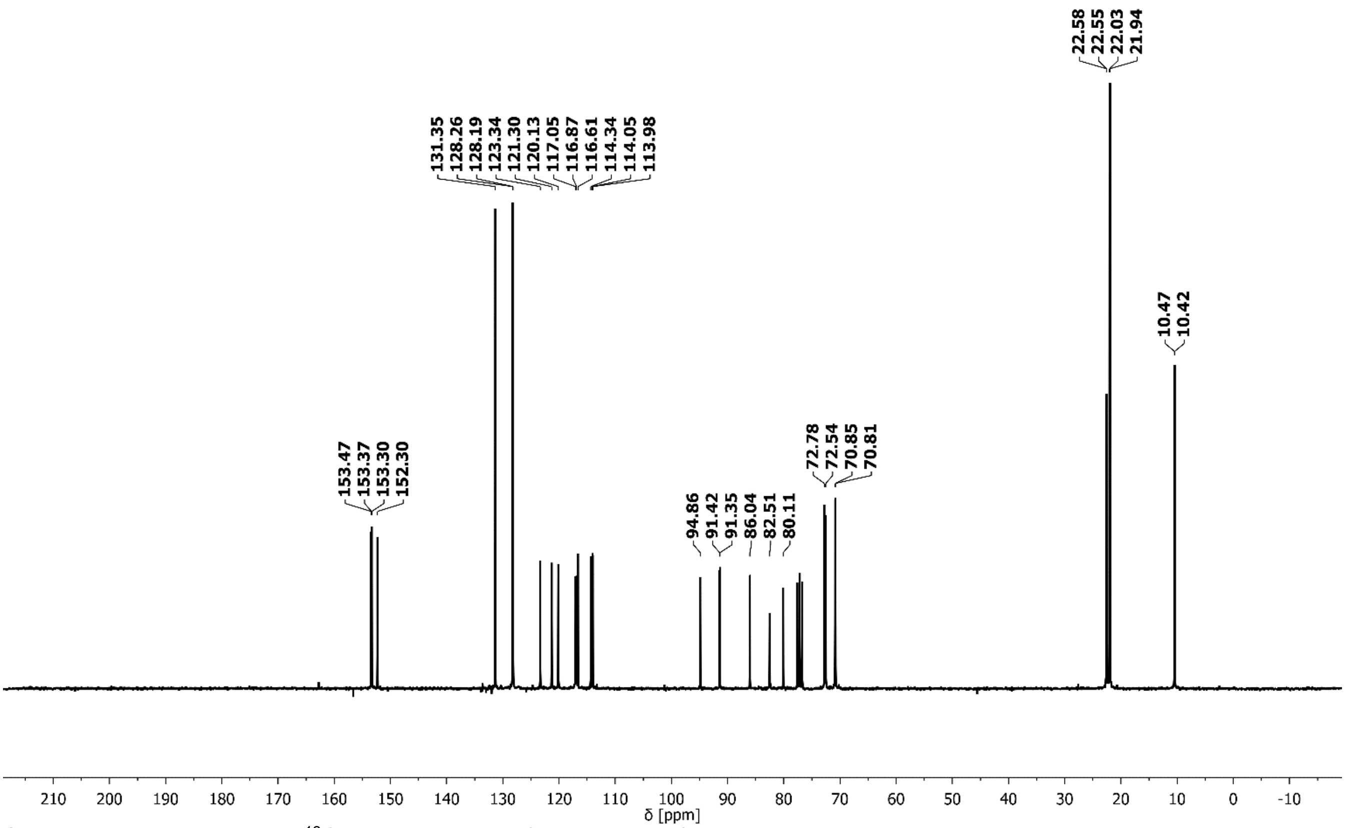

Supplementary Figure 59: ^{13}C NMR spectrum of sequence-defined, deprotected dimer **15**.

Synthesis of ((2,5-bis(cyclohexyloxy)-4-((2,5-diisopropoxy-4-((4-(phenylethynyl)-2,5-dipropoxyphenyl) ethynyl)phenyl)ethynyl)phenyl)ethynyl)trimethylsilane **16**

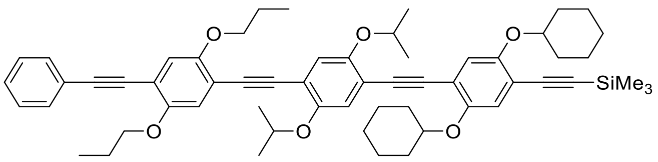

1,4-Bis(cyclohexyloxy)-2-iodo-5-trimethylsilylacetylenebenzene (6.76 g, 14.4 mmol, 5.00 eq.), 10 mol% bis(triphenylphosphine) palladium(II) dichloride (202 mg, 288 µmol) and 2.5 mol% copper(I) iodide (13.7 mg, 72.0 µmol) were placed into a Schlenk flask and degassed three times. Under continuous argon flow, 60 mL dry THF and 3.99 mL dry triethylamine (2.91 g, 28.8 mmol, 10.0 eq.) were added and the mixture was stirred for 10 minutes. Subsequently, compound **15** (1.54 g, 2.88 mmol, 1.00 eq.) in 50 mL THF was added dropwise with a syringe. The reaction mixture was stirred for 72 h at 45 °C, taken up in dichloromethane and washed with saturated NH_4Cl solution. The aqueous phase was extracted three times with dichloromethane. The combined organic layers were dried over Na_2SO_4, filtered and concentrated under reduced pressure. The residue was purified by silica column chromatography (dichloromethane / cyclohexane 2:1) and a further silica column (cyclohexane / ethyl acetate 20:1 → 15:1) to yield the product as a yellow solid (1.40 g, 54%). TLC (cyclohexane / dichloromethane 2:1) R_f = 0.17; ^{1}H NMR ($CDCl_3$, 300 MHz): *δ* (ppm) = 7.58–7.51 (m, 2 H, 2 C**H**$_{\textbf{aromatic}}$C-C≡C), 7.41–7.30 (m, 3 H, 3 C**H**$_{\textbf{aromatic}}$), 7.08–6.99 (m, 5 H, 5 C**H**$_{\textbf{aromatic}}$CO), 6.97 (s, 1 H, 1 C**H**$_{\textbf{aromatic}}$C-C≡C-Si), 4.66–4.44 (m, 2 H, 2 C**H(**$CH_3)_2$)), 4.37–4.17 (m, 2 H, 2 C**H**CH_2), 4.10-3.91 (m, 4 H, 2 C**H**$_{\textbf{2}}$O), 2.02-1.75 (m, 12 H, 2 C**H**$_{\textbf{2}}$$CH_3$, 4 C**H**$_{\textbf{equatorial}}$CHO, 4 C**H**$_{\textbf{equatorial}}$$CH_2$CHO), 1.75–1.56 (m, 6 H, 4 C**H**$_{\textbf{axial}}$CHO, 2 C**H**$_{\textbf{equatorial}}$$CH_2CH_2$CHO), 1.46–1.31 (m, 18 H, 4 C**H**$_{\textbf{3}}$CH, 4 C**H**$_{\textbf{axial}}$$CH_2$CHO, 2 C**H**$_{\textbf{axial}}$$CH_2CH_2$CHO), 1.10 (dt, *J* = 7.4, 4.9 Hz, 6 H, 2 C**H**$_{\textbf{3}}$$CH_2$), 0.26 (s, 9 H, 3 C**H**$_{\textbf{3}}$Si), ^{13}C NMR ($CDCl_3$, 100 MHz): *δ* (ppm) = 153.71, 153.60, 153.30, 152.87, 152.85, 152.65, 131.68, 128.45, 128.38, 123.58, 121.57, 121.19, 121.04, 120.91, 117.41, 117.23, 116.52, 116.30, 116.24, 116.03, 114.39, 114.20, 101.66, 99.93, 95.00, 91.81, 91.62, 91.53, 91.41, 86.09, 77.89, 77.37, 73.19, 73.14, 71.27, 71.23, 31.90, 31.77, 25.84, 25.82, 23.60, 23.35, 22.85, 22.81, 22.39, 22.36, 10.74, 10.70, 0.08; FAB of $C_{59}H_{70}O_6Si$ (M+H$^+$ = 903.6); HRMS (FAB) of $C_{59}H_{70}O_6Si$ [M+H$^+$] calc. 902.4942, found 902.4943; IR (ATR) v = 2931.5, 2856.4, 2149.7, 1595.6, 1485.6, 1414.3, 1383.9, 1270.8, 1248.4, 1197.6, 1106.1, 1040.2, 1015.8, 963.2, 887.7, 839.1, 755.1, 689.2, 646.8, 524.7, 460.8 cm^{-1}.

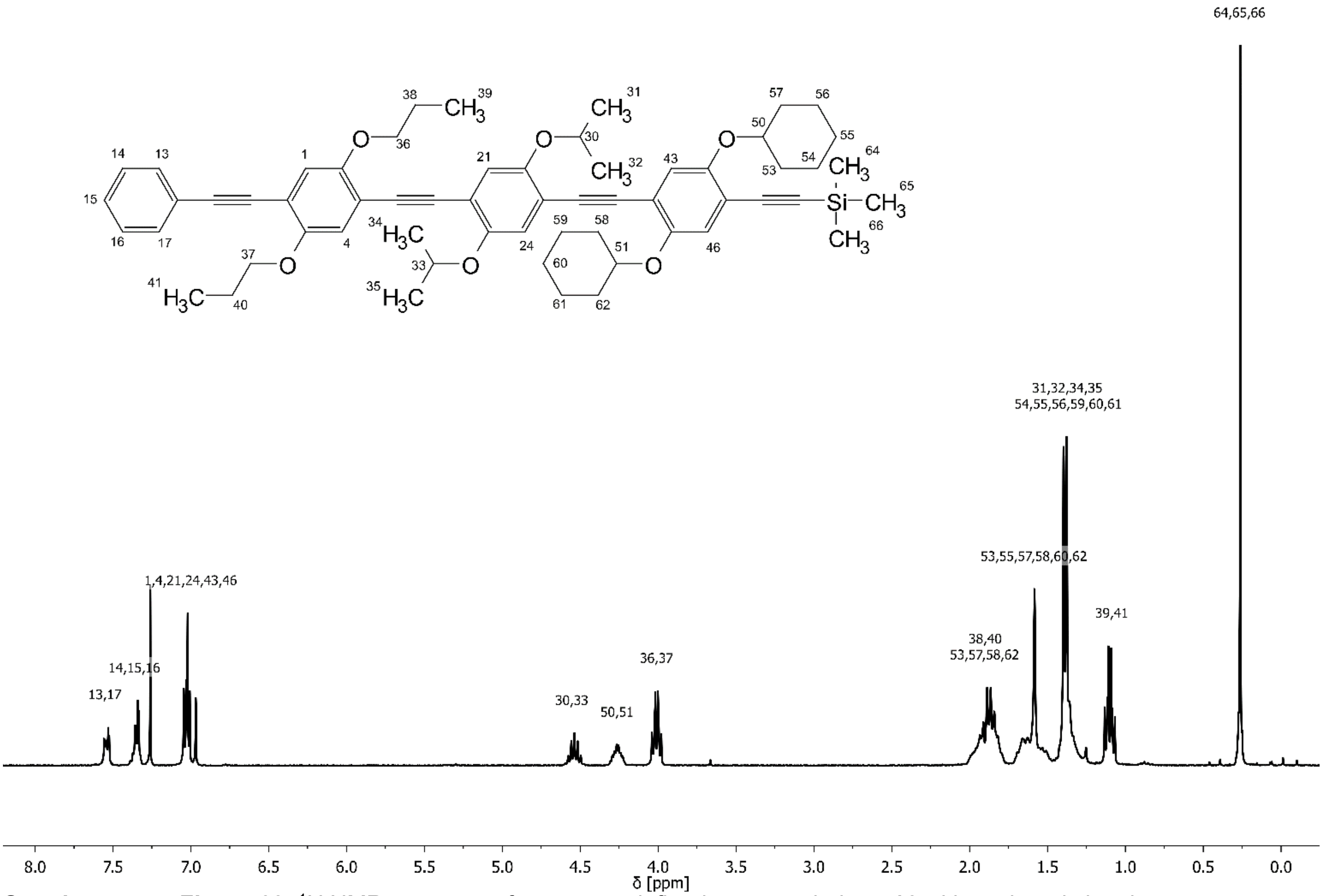

Supplementary Figure 60: ^{1}H NMR spectrum of sequence-defined, protected trimer **16** with assigned signals.

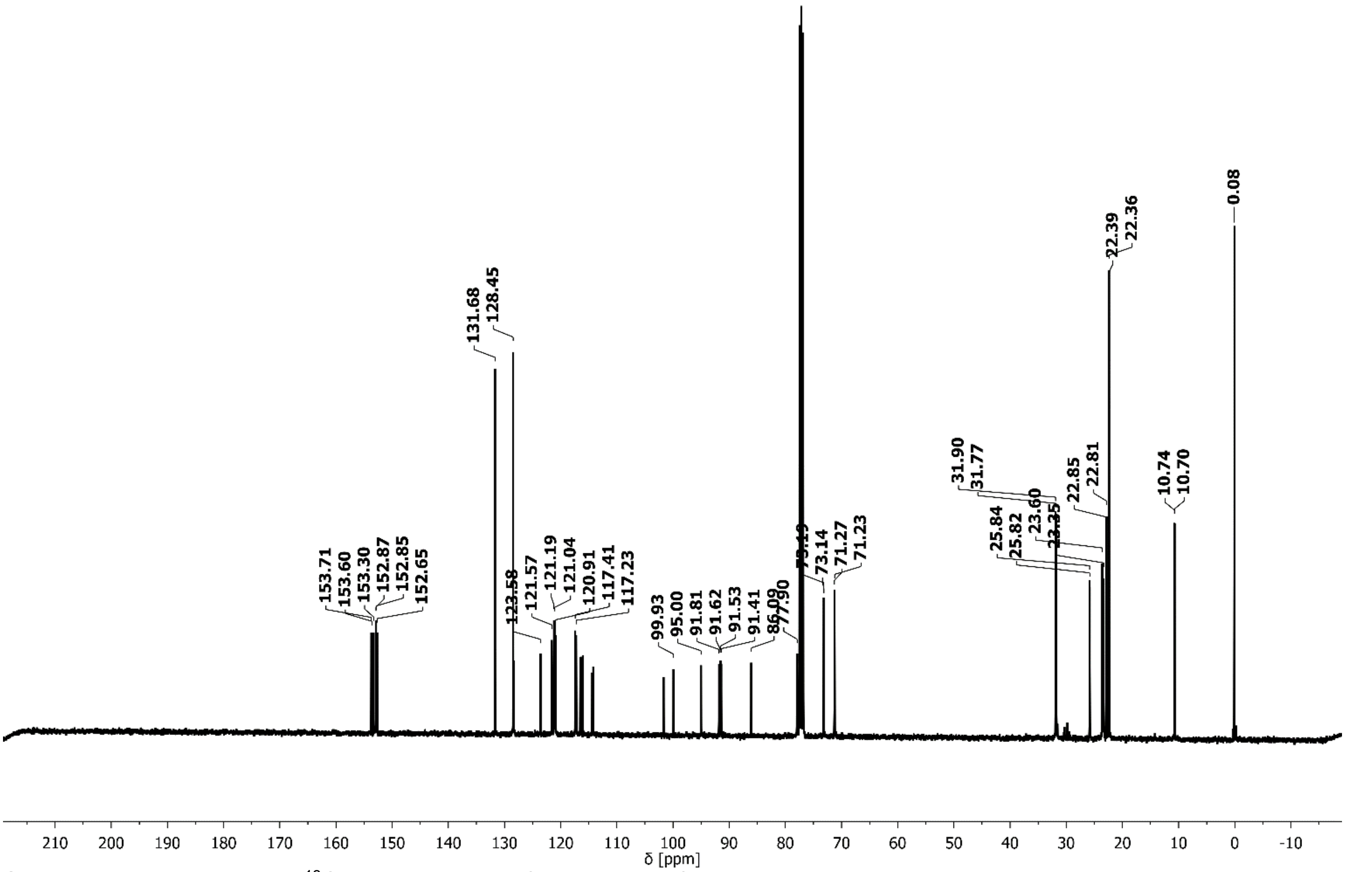

Supplementary Figure 61: ^{13}C NMR spectrum of sequence-defined, protected trimer **16**.

Synthesis of sequence-defined, deprotected trimer **17**

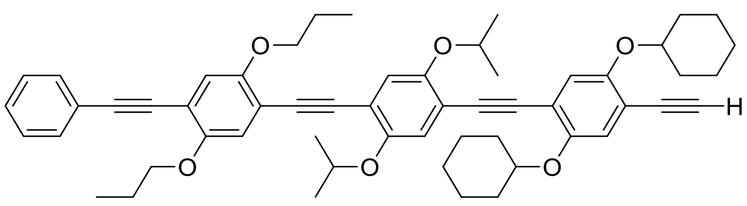

Compound **16** (1.35 g, 1.49 mmol, 1.00 eq.) and two equivalents of potassium carbonate (413 mg, 2.99 mmol) were placed in a Schlenk flask and degassed three times. Under continuous argon flow 70 mL dichloromethane and 70 mL methanol were added. The reaction mixture was stirred overnight at room temperature under argon atmosphere and quenched with distilled water. The aqueous phase was extracted three times with dichloromethane, dried over Na_2SO_4, filtered and concentrated under reduced pressure. The residue was purified by silica column chromatography (cyclohexane / ethyl acetate 8:1) to yield the product as an orange solid (1.22 g, 98%). TLC (cyclohexane / dichloromethane 2:1) R_f = 0.29; ^{1}H NMR ($CDCl_3$, 300 MHz): δ (ppm) = 7.59–7.49 (m, 2 H, 2 C**H**$_{\mathbf{aromatic}}$C-C≡C), 7.43–7.30 (m, 3 H, 3 C**H**$_{\mathbf{aromatic}}$), 7.10–6.97 (m, 6 H, 6 C**H**$_{\mathbf{aromatic}}$CO), 4.65–4.45 (m, 2 H, 2 C**H(**$CH_3)_2$)), 4.35–4.14 (m, 2 H, 2 C**H**CH_2), 4.10–3.92 (m, 4 H, 2 C**H₂**O), 3.32 (s, 1 H, 1 C≡C-**H**), 2.10–1.75 (m, 12 H, 2 C**H₂**CH_3, 4 C**H**$_{\mathbf{equatorial}}$CHO, 4 C**H**$_{\mathbf{equatorial}}$$CH_2$CHO), 1.73–1.48 (m, 6 H, 4 C**H**$_{\mathbf{axial}}$CHO, 2 C**H**$_{\mathbf{equatorial}}$$CH_2CH_2$CHO), 1.47–1.23 (m, 18 H, 4 C**H₃**CH, 4 C**H**$_{\mathbf{axial}}$$CH_2$CHO, 2 C**H**$_{\mathbf{axial}}$$CH_2CH_2$CHO), 1.10 (dt, J = 7.4, 4.8 Hz, 6 H, 2 C**H₃**CH_2), ^{13}C NMR ($CDCl_3$, 100 MHz): δ (ppm) = 153.73, 153.63, 153.38, 152.91, 152.39, 131.70, 128.47, 128.40, 123.60, 121.55, 121.25, 121.07, 120.46, 117.46, 117.26, 116.90, 116.35, 116.25, 114.47, 114.39, 114.25, 95.02, 91.79, 91.57, 91.47, 86.10, 82.22, 80.44, 78.03, 77.36, 73.24, 73.19, 71.30, 71.27, 31.91, 25.82, 25.76, 23.75, 23.61, 22.86, 22.83, 22.41, 22.38, 10.75, 10.71; FAB of $C_{56}H_{62}O_6$ (M+H$^+$ = 831.5); HRMS (FAB) of $C_{56}H_{62}O_6$ [M+H$^+$] calc. 830.4546, found 830.4547; IR (ATR) ν = 3279.97, 2931.27, 2856.32, 1596.03, 1485.92, 1450.30, 1415.15, 1383.81, 1370.35, 1270.94, 1197.47, 1137.72, 1105.86, 1040.21, 1015.97, 962.78, 886.50, 859.45, 754.92, 689.36, 645.52, 527.72, 457.80 cm^{-1}.

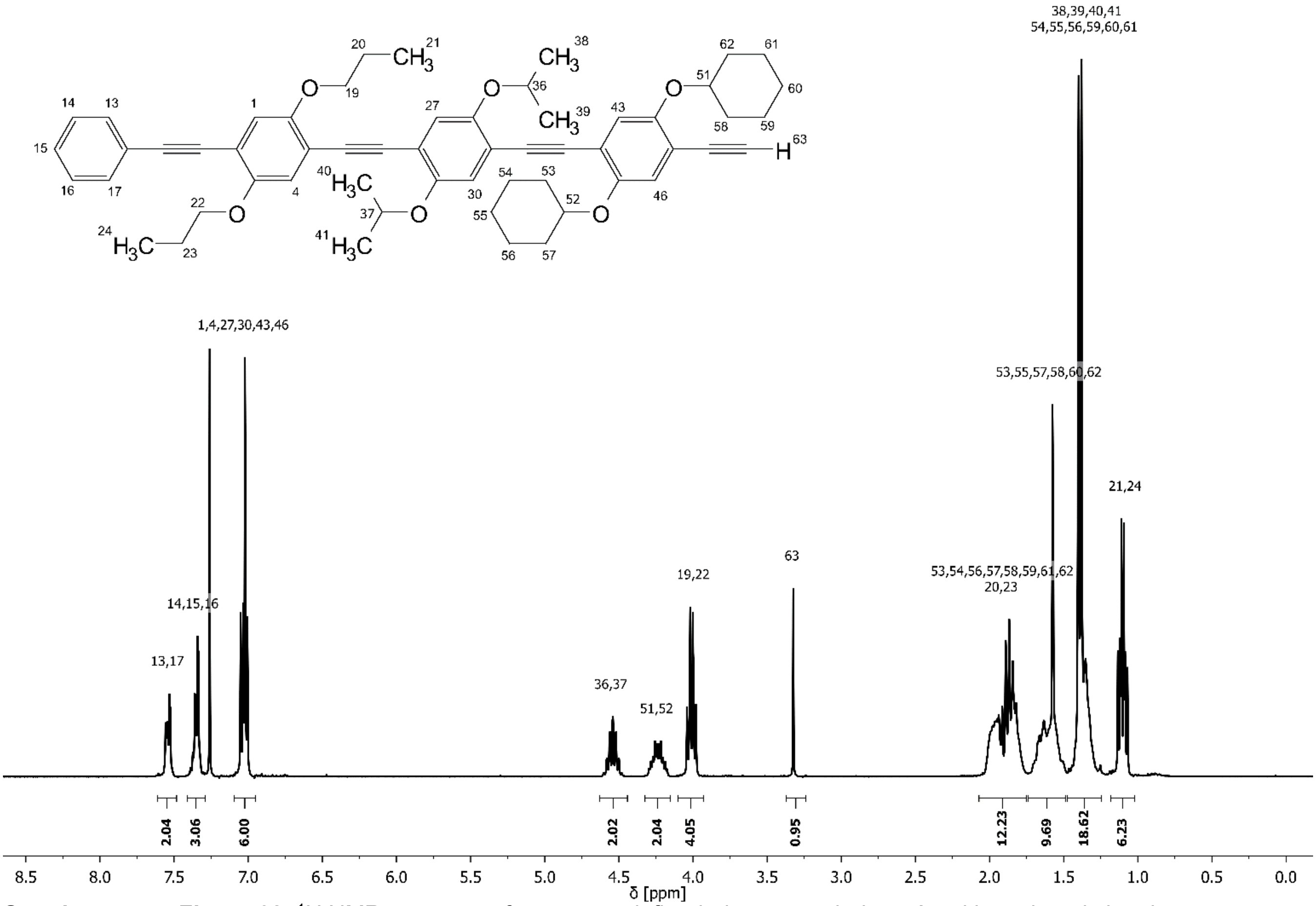

Supplementary Figure 62: ^{1}H NMR spectrum of sequence-defined, deprotected trimer **17** with assigned signals.

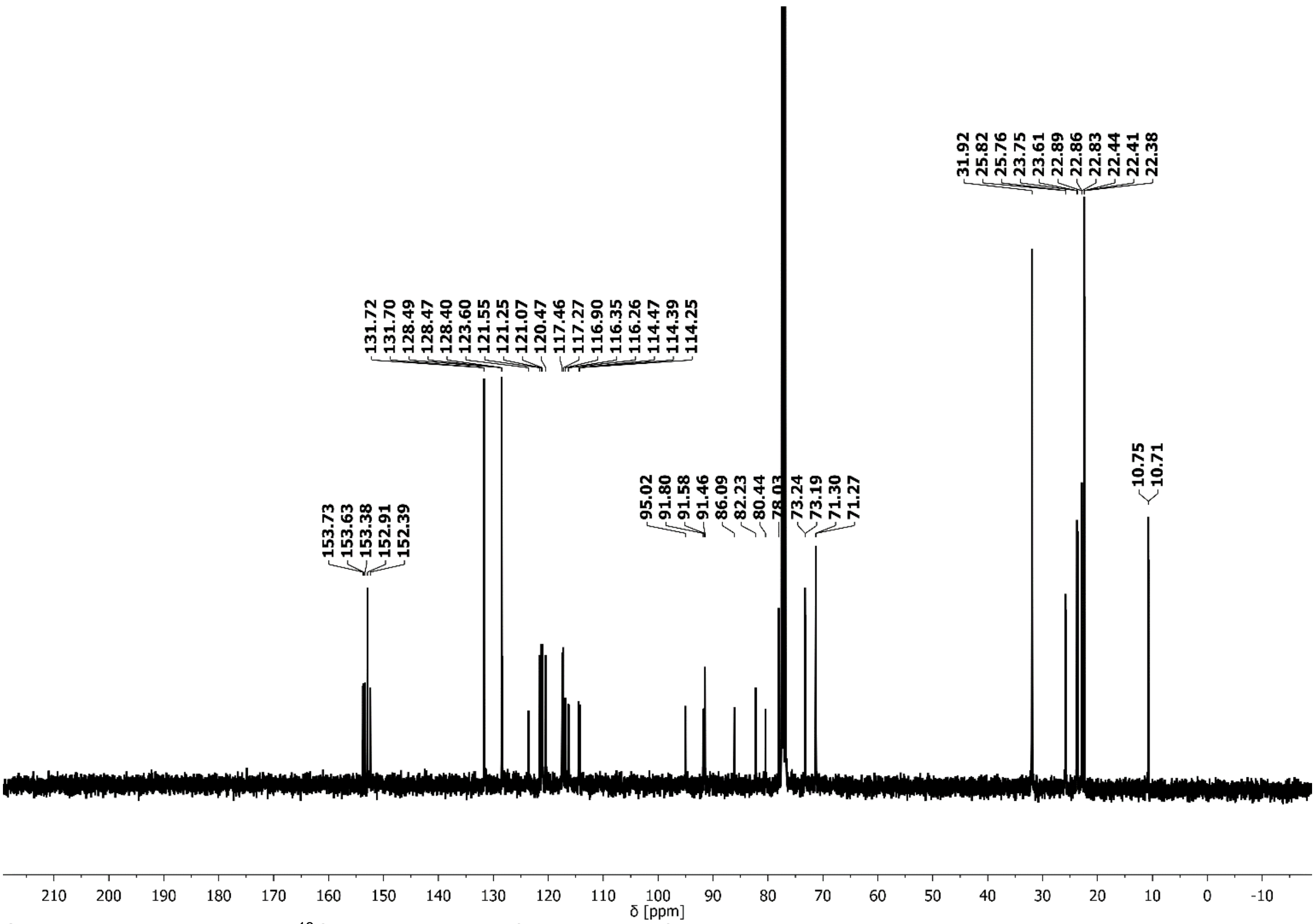

Supplementary Figure 63: ^{13}C NMR spectrum of sequence-defined, deprotected trimer **17.**

Synthesis of ((4-((2,5-bis(cyclohexyloxy)-4-((2,5-diisopropoxy-4-((4-(phenylethynyl)-2,5-dipropoxyphenyl)ethynyl)phenyl)ethynyl)phenyl)ethynyl)-2,5-dimethoxyphenyl)ethynyl)trimethylsilane **18**

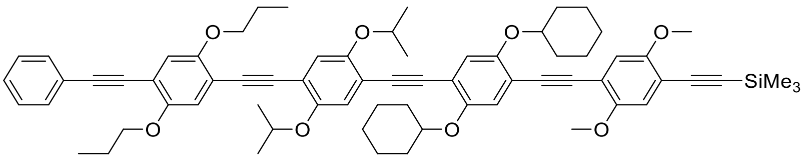

1,4-Bis(methoxy)-2-iodo-5-trimethylsilylacetylenebenzene (2.60 g, 7.22 mmol, 5.00 eq.), 10 mol% bis(triphenylphosphine) palladium(II) dichloride (101 mg, 0.144 mmol) and 2.5 mol% copper(I) iodide (6.9 mg, 36.1 µmol) were placed into a Schlenk flask and degassed three times. Under continuous argon flow, 60 mL dry THF and 2.00 mL dry triethylamine (1.46 g, 14.4 mmol, 10.0 eq.) were added and the mixture was stirred for 10 minutes. Subsequently, compound **17** (1.20 g, 1.44 mmol, 1.00 eq.) in 40 mL THF was added dropwise with a syringe. The reaction mixture was stirred for 72 h at 45 °C, taken up in dichloromethane and washed with saturated NH_4Cl solution. The aqueous phase was extracted three times with dichloromethane. The combined organic layers were dried over Na_2SO_4, filtered and concentrated under reduced pressure. The residue was purified by silica column chromatography (dichloromethane / cyclohexane 1:1 → 9:1) to yield the product as a yellow solid (568 mg, 37%). TLC (dichloromethane / cyclohexane 3:1) R_f = 0.15; ^{1}H NMR ($CDCl_3$, 300 MHz): *δ* (ppm) = 7.58–7.49 (m, 2 H, 2 C$\mathbf{H}_{\mathbf{aromatic}}$C-C≡C), 7.39–7.31 (m, 3 H, 3 C$\mathbf{H}_{\mathbf{aromatic}}$), 7.11–6.90 (m, 8 H, 8 C$\mathbf{H}_{\mathbf{aromatic}}$CO), 4.55 (hept, *J* = 6.1 Hz, 2 H, 2 C**H**$(CH_3)_2$)), 4.38–4.23 (m, 2 H, 2 C**H**CH_2), 4.08–3.95 (m, 4 H, 2 C$\mathbf{H_2}$O), 3.88, 3.86 (s, 6 H, 2 C$\mathbf{H_3}$O), 2.07–1.79 (m, 12 H, 2 C$\mathbf{H_2}$$CH_3$, 4 C$\mathbf{H}_{\mathbf{equatorial}}$CHO, 4 C$\mathbf{H}_{\mathbf{equatorial}}$$CH_2$CHO), 1.77–1.48 (m, 6 H, 4 C$\mathbf{H}_{\mathbf{axial}}$CHO, 2 C$\mathbf{H}_{\mathbf{equatorial}}$$CH_2CH_2$CHO), 1.48–1.27 (m, 18 H, 4 C$\mathbf{H_3}$CH, 4 C$\mathbf{H}_{\mathbf{axial}}$$CH_2$CHO, 2 C$\mathbf{H}_{\mathbf{axial}}$$CH_2CH_2$CHO), 1.10 (dt, *J* = 7.4, 4.5 Hz, 6 H, 2 C$\mathbf{H_3}$$CH_2$), 0.28 (s, 9 H, 3 C$\mathbf{H_3}$Si); ^{13}C NMR ($CDCl_3$, 100 MHz): *δ* (ppm) = 154.43, 153.80, 153.74, 153.63, 152.91, 152.89, 152.81, 152.66, 131.70, 128.46, 128.39, 123.61, 121.24, 121.08, 121.00, 120.72, 117.47, 117.28, 116.47, 116.36, 116.27, 116.24, 115.98, 115.80, 114.43, 114.24, 114.12, 113.18, 101.10, 100.58, 95.01, 92.24, 91.83, 91.77, 91.68, 91.43, 91.07, 86.11, 77.95, 77.91, 73.22, 73.18, 71.31, 71.27, 56.52, 56.49, 31.95, 31.86, 25.90, 25.85, 23.64, 23.53, 22.86, 22.83, 22.43, 22.39, 10.75, 10.71, 0.16; FAB of $C_{69}H_{78}O_8Si$ (M+H$^+$ = 1064.3); IR (ATR) ν = 2931.1, 2855.7, 2148.8, 1717.1, 1595.9, 1505.5, 1486.7, 1464.4, 1415.4, 1384.5, 1272.2, 1248.7, 1207.1, 1106.3, 1038.6, 1017.0, 963.5, 856.5, 840.5, 755.4, 690.1, 633.0, 527.4, 457.4 cm^{-1}.

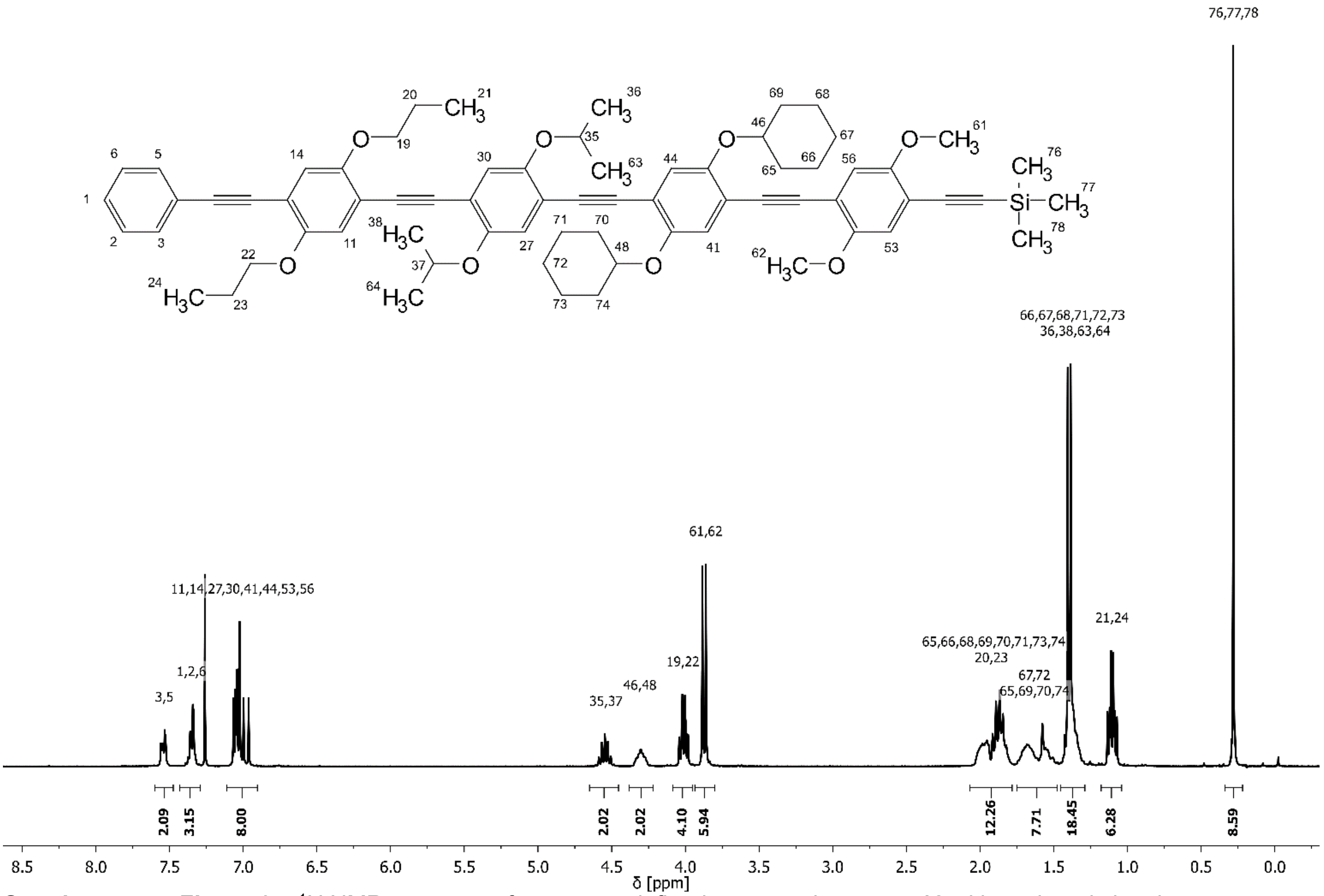

Supplementary Figure 64: ^{1}H NMR spectrum of sequence-defined, protected tetramer **18** with assigned signals.

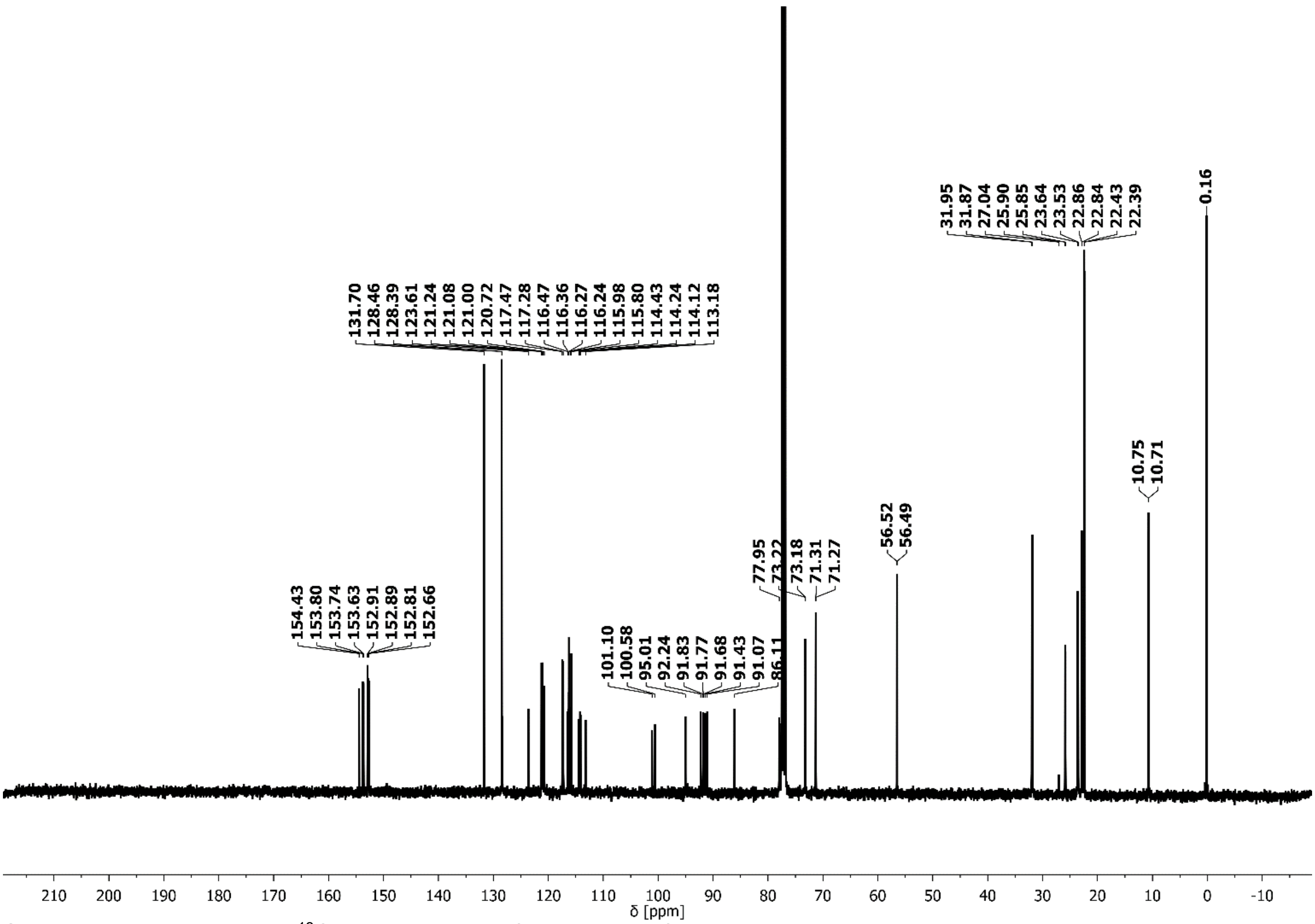

Supplementary Figure 65: ^{13}C NMR spectrum of sequence-defined, protected tetramer **18**.

Synthesis of sequence-defined, deprotected tetramer **19**

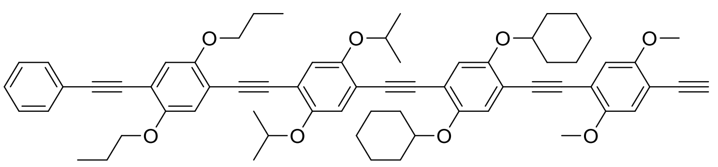

Compound **18** (500 mg, 0.470 mmol, 1.00 eq.) and two equivalents of potassium carbonate (130 mg, 0.940 mmol) were placed in a Schlenk flask and degassed three times. Under continuous argon flow 25 mL dichloromethane and 25 mL methanol were added. The reaction mixture was stirred overnight at room temperature under argon atmosphere and quenched with distilled water. The aqueous phase was extracted three times with dichloromethane, dried over Na_2SO_4, filtered and concentrated under reduced pressure. The residue was purified by silica column chromatography (dichloromethane / cyclohexane 12:1) to yield the product as a yellow solid (397 mg, 85%). TLC (dichloromethane / cyclohexane 3:1) R_f = 0.11; ^{1}H NMR ($CDCl_3$, 300 MHz): δ (ppm) = 7.60–7.48 (m, 2 H, 2 C**H**$_{\mathbf{aromatic}}$C-C≡C), 7.42–7.29 (m, 3 H, 3 C**H**$_{\mathbf{aromatic}}$), 7.12–6.95 (m, 8 H, 8 C**H**$_{\mathbf{aromatic}}$CO), 4.55 (hept, *J* = 6.1 Hz, 2 H, 2 C**H(**$CH_3)_2$)), 4.40–4.22 (m, 2 H, 2 C**H**CH_2), 4.08–3.94 (m, 4 H, 2 C**H$_2$**O), 3.89, 3.88 (2 s, 6 H, 2 C**H$_3$**O), 3.42 (s, 1 H, 1 C≡C-**H**), 2.09–1.77 (m, 12 H, 2 C**H$_2$**CH_3, 4 C**H**$_{\mathbf{equatorial}}$CHO, 4 C**H**$_{\mathbf{equatorial}}$$CH_2$CHO), 1.77–1.48 (m, 6 H, 4 C**H**$_{\mathbf{axial}}$CHO, 2 C**H**$_{\mathbf{equatorial}}$$CH_2CH_2$CHO), 1.48–1.27 (m, 18 H, 4 C**H**$_3$CH, 4 C**H**$_{\mathbf{axial}}$$CH_2$CHO, 2 C**H**$_{\mathbf{axial}}$$CH_2CH_2$CHO), 1.10 (dt, *J* = 7.4, 4.5 Hz, 6 H, 2 C**H$_3$**CH_2); ^{13}C NMR ($CDCl_3$, 100 MHz): δ (ppm) = 154.65, 153.83, 153.75, 153.64, 152.90, 152.84, 152.66, 131.71, 128.47, 128.40, 123.61, 121.23, 121.09, 120.96, 120.78, 117.47, 117.28, 116.58, 116.37, 116.34, 116.30, 115.87, 115.66, 114.57, 114.42, 114.25, 111.93, 95.02, 92.35, 91.83, 91.75, 91.72, 91.45, 90.83, 86.11, 82.75, 80.10, 77.98, 77.88, 73.22, 73.19, 71.32, 71.28, 56.54, 56.49, 31.96, 31.87, 25.90, 25.85, 23.64, 23.53, 22.87, 22.84, 22.43, 22.39, 10.76, 10.71; ESI-MS of $C_{66}H_{70}O_8$ (M+H$^+$ = 991.51); IR (ATR) ν = 3277.4, 2930.3, 2855.9, 2209.2, 2104.2, 1716.0, 1596.1, 1504.9, 1486.5, 1463.9, 1415.1, 1384.4, 1272.3, 1204.5, 1138.1, 1106.1, 1038.0, 1016.5, 962.4, 861.0, 754.2, 689.9, 644.5, 527.9, 458.0 cm^{-1}.

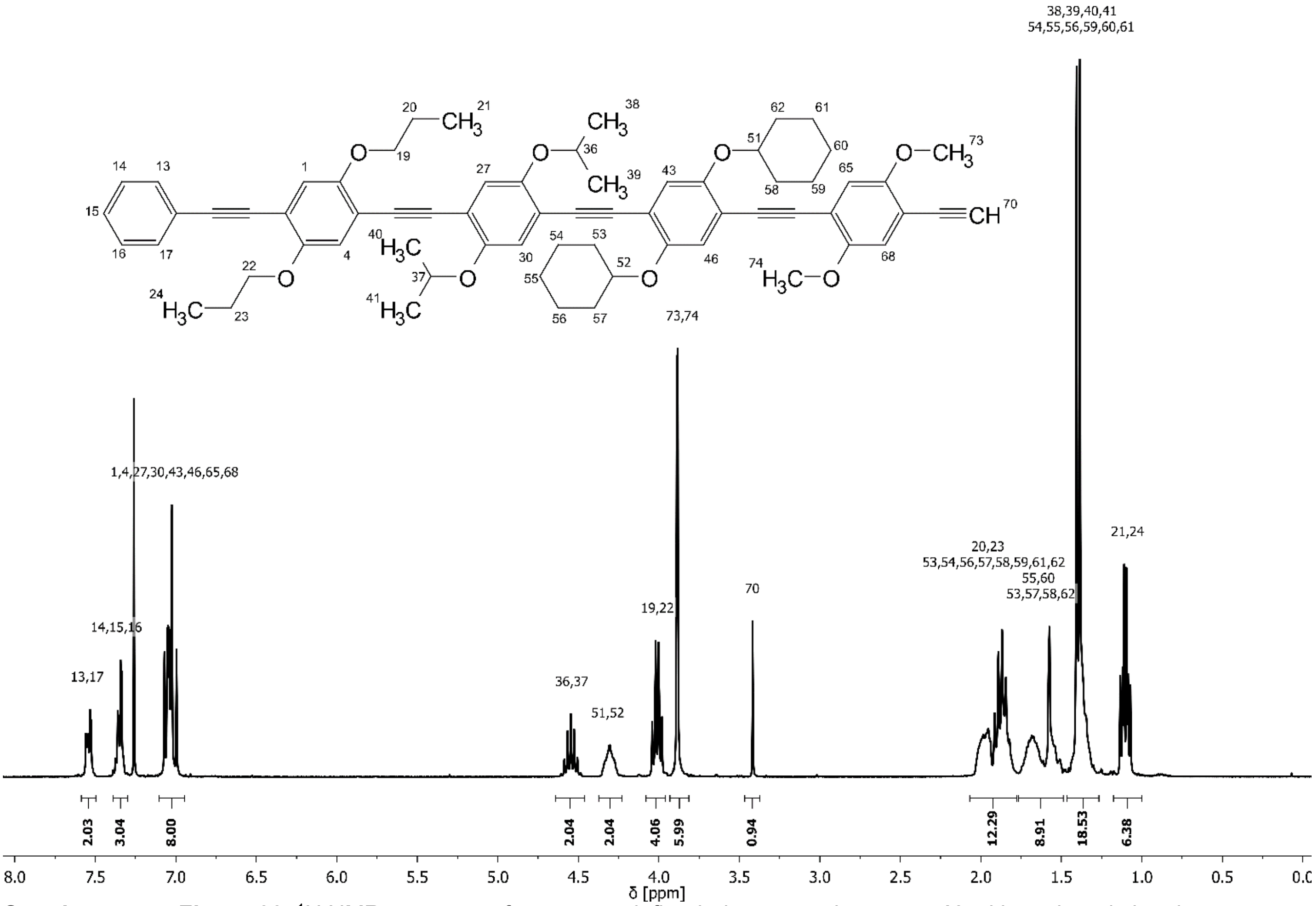

Supplementary Figure 66: ^{1}H NMR spectrum of sequence-defined, deprotected tetramer **19** with assigned signals.

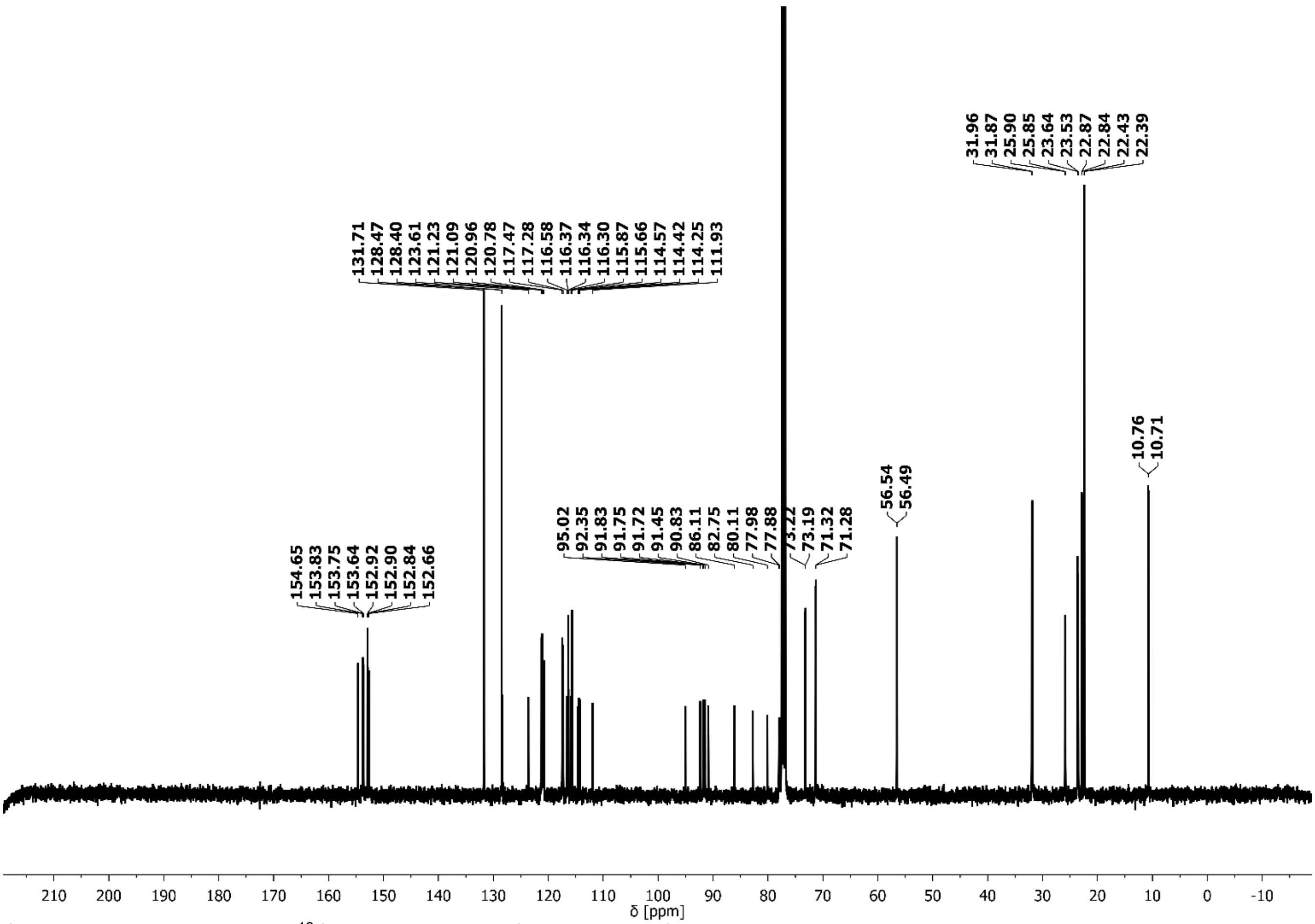

Supplementary Figure 67: ^{13}C NMR spectrum of sequence-defined, deprotected tetramer **19**.

Synthesis of ((4-((4-((2,5-bis(cyclohexyloxy)-4-((2,5-diisopropoxy-4-((4-(phenylethynyl)-2,5-dipropoxyphenyl)ethynyl)phenyl)ethynyl)phenyl)ethynyl)-2,5-dimethoxyphenyl)ethynyl)-2,5-bis(octyloxy)phenyl)ethynyl)trimethylsilane **20**

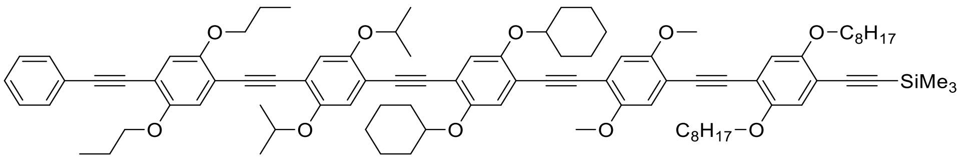

1,4-Bis(octyloxy)-2-iodo-5-trimethylsilylacetylenebenzene (841 mg, 1.51 mmol, 5.00 eq.), 10 mol% bis(triphenylphosphine) palladium(II) dichloride (21.2 mg, 30.3 µmol) and 2.5 mol% copper(I) iodide (1.4 mg, 7.56 µmol) were placed into a Schlenk flask and degassed three times. Under continuous argon flow, 20 mL dry THF and 420 µL dry triethylamine (306 mg, 3.03 mmol, 10.0 eq.) were added and the mixture was stirred for 10 minutes. Subsequently, compound **19** (300 mg, 0.303 mmol, 1.00 eq.) in 20 mL THF was added dropwise with a syringe. The reaction mixture was stirred for 72 h at 45 °C, taken up in dichloromethane and washed with saturated NH_4Cl solution. The aqueous phase was extracted three times with dichloromethane. The combined organic layers were dried over Na_2SO_4, filtered and concentrated under reduced pressure. The residue was purified by silica column chromatography (dichloromethane / cyclohexane 1:1 → 49:1) to yield the product as a yellow solid (140 mg, 33%). TLC (dichloromethane / cyclohexane 3:1) R_f = 0.25; ^{1}H NMR ($CDCl_3$, 300 MHz): δ (ppm) = 7.59–7.47 (m, 2 H, 2 C**H**$_{\mathbf{aromatic}}$C-C≡C), 7.40–7.29 (m, 3 H, 3 C**H**$_{\mathbf{aromatic}}$), 7.21–6.85 (m, 10 H, 10 C**H**$_{\mathbf{aromatic}}$CO), 4.55 (hept, *J* = 6.1 Hz, 2 H, 2 C**H(**$CH_3)_2$)), 4.38–4.22 (m, 2 H, 2 C**H**CH_2), 4.09–3.93 (m, 8 H, 4 C**H₂**O), 3.90, 3.89 (s, 6 H, 2 C**H₃**O), 2.13–1.77 (m, 16 H, 4 C**H₂**CH_3, 4 C**H**$_{\mathbf{equatorial}}$CHO, 4 C**H**$_{\mathbf{equatorial}}$$CH_2$CHO), 1.75–1.61 (m, 4 H, 4 C**H**$_{\mathbf{axial}}$CHO), 1.60–1.45 (m, 6 H, 2 C**H**$_{\mathbf{equatorial}}$$CH_2CH_2$CHO, 2 C**H**$_2CH_2CH_2$O), 1.45–1.18 (m, 34 H, 4 C**H₃**CH, 4 C**H**$_{\mathbf{axial}}$$CH_2$CHO, 2 C**H**$_{\mathbf{axial}}$$CH_2CH_2$CHO, 8 C**H₂**$(CH_2)_n$), 1.10 (dt, *J* = 7.4, 4.4 Hz, 6 H, 2 C**H₃**CH_2), 0.88 (dt, *J* = 10.0, 6.8 Hz, 6 H, 2 C**H₃**$(CH_2)_n$), 0.26 (s, 9 H, 3 C**H₃**Si); ^{13}C NMR ($CDCl_3$, 125 MHz): δ (ppm) = 154.25, 153.94, 153.90, 153.71, 153.62, 153.60, 152.89, 152.86, 152.77, 152.64, 131.69, 128.46, 128.39, 123.59, 121.21, 121.04, 120.97, 120.70, 117.40, 117.25, 117.21, 116.92, 116.41, 116.34, 116.24, 116.01, 115.66, 114.38, 114.26, 114.19, 113.86, 113.79, 113.63, 101.29, 100.33, 95.01, 92.29, 91.83, 91.78, 91.67, 91.45, 91.42, 91.22, 86.09, 77.94, 77.88, 73.20, 73.16, 71.27, 71.23, 69.65, 69.56, 56.50, 56.44, 31.99, 31.97, 31.95, 31.87, 29.55, 29.53, 29.51, 29.47, 29.45, 29.43, 26.19, 26.07, 25.91, 25.85, 23.65, 23.55, 22.86, 22.83, 22.82, 22.80, 22.43, 22.39, 14.25, 14.23, 10.77, 10.72, 0.11; ESI-MS of $C_{93}H_{114}O_{10}Si$ (M+H$^+$ = 1419.82); IR (ATR) ν = 2926.5, 2853.8, 2149.1, 1595.5, 1486.5, 1465.2, 1413.9, 1383.3, 1272.7, 1208.4, 1106.7, 1038.5, 963.4, 840.8, 754.9, 689.2, 628.7, 461 cm^{-1}.

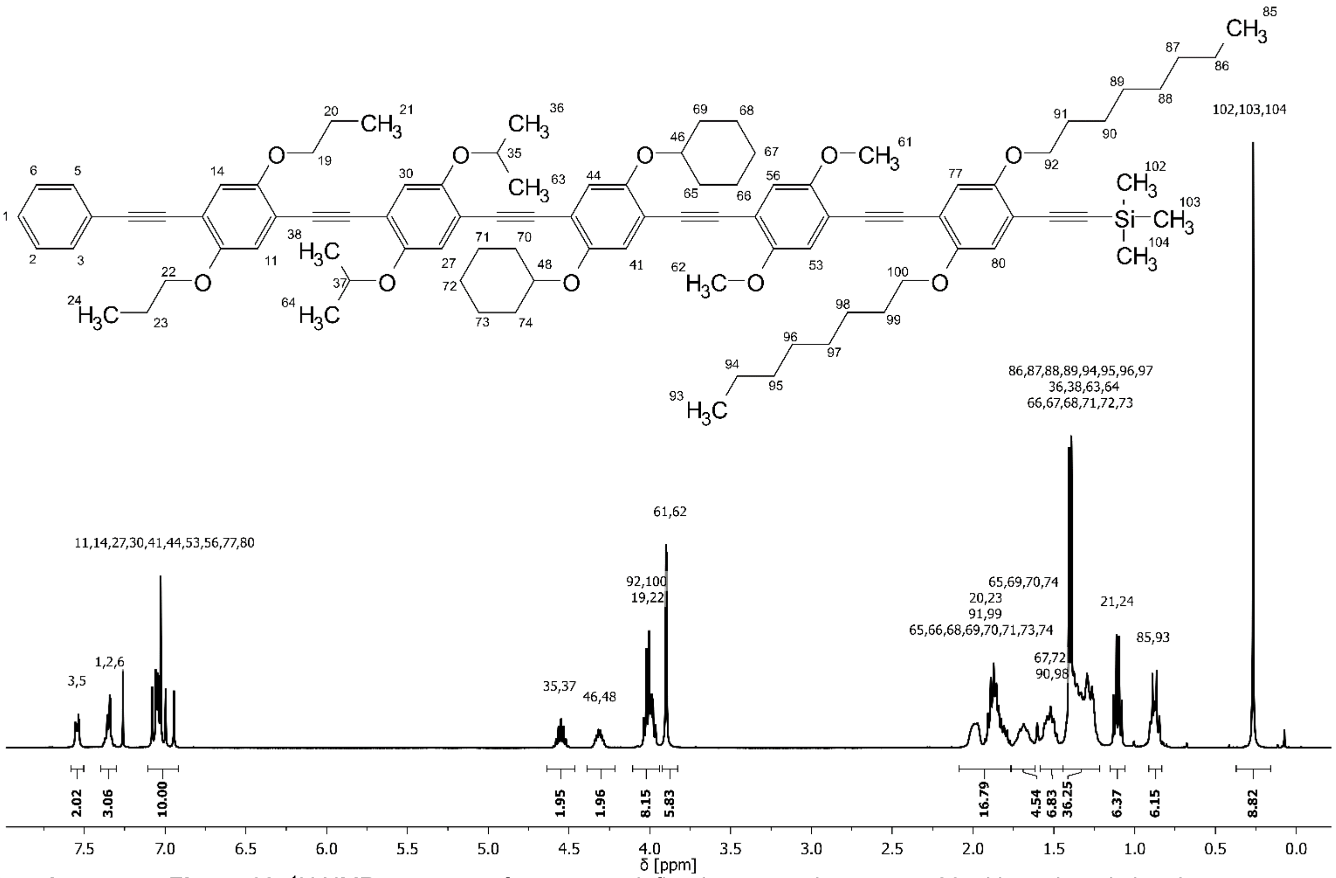

Supplementary Figure 68: ^{1}H NMR spectrum of sequence-defined, protected pentamer **20** with assigned signals.

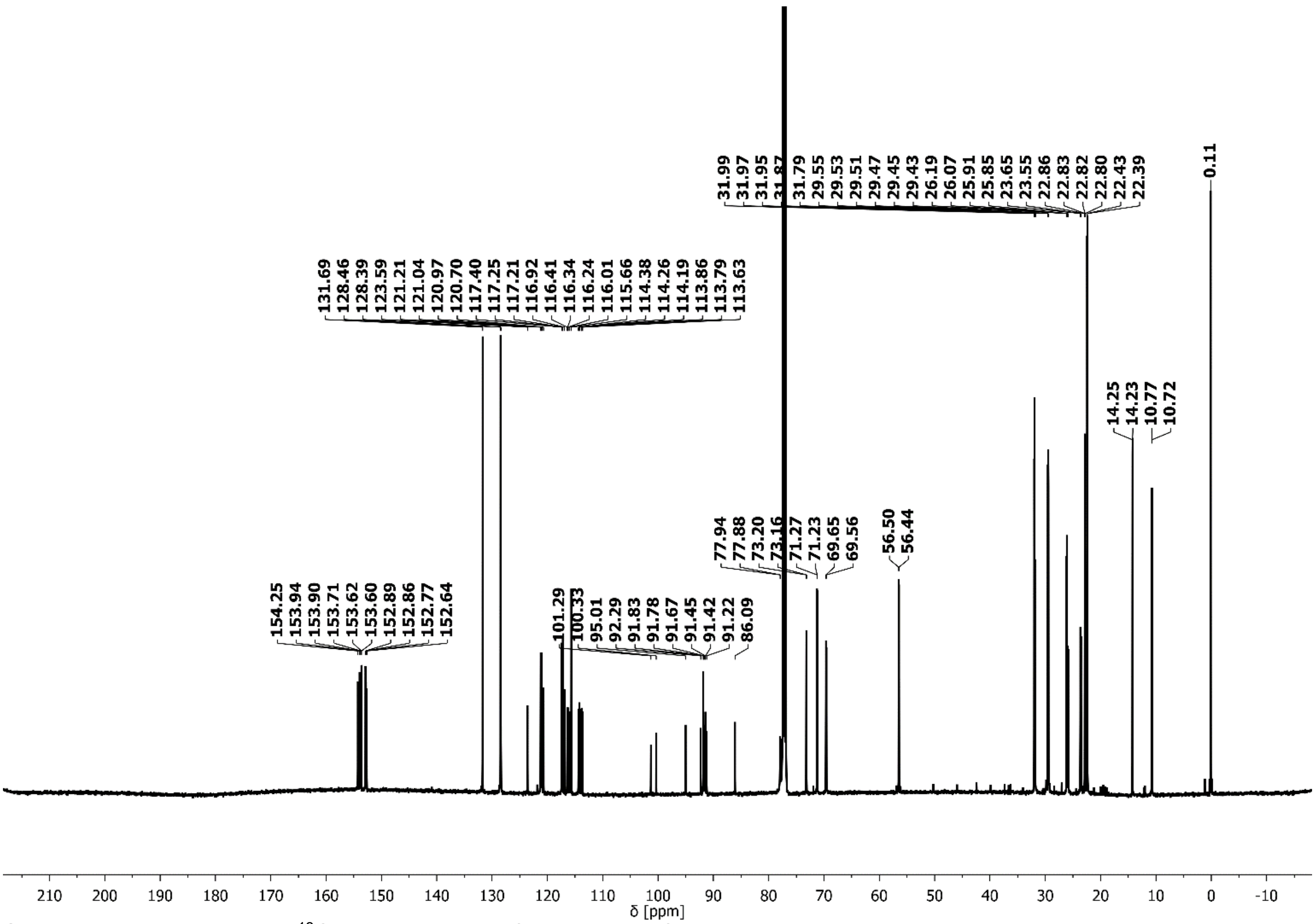

Supplementary Figure 69: ^{13}C NMR spectrum of sequence-defined, protected pentamer **20**.

Synthesis of sequence-defined, deprotected pentamer **21**

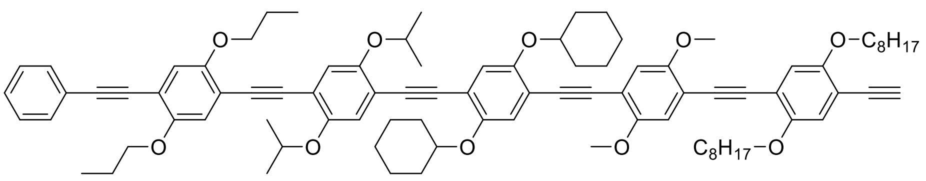

Compound **20** (80.0 mg, 56.3 µmol, 1.00 eq) and two equivalents of potassium carbonate (15.6 mg, 0.113 mmol) were placed in a Schlenk flask and degassed three times. Under continuous argon flow 8 mL dichloromethane and 4 mL methanol were added. The reaction mixture was stirred overnight at room temperature under argon atmosphere and quenched with distilled water. The aqueous phase was extracted three times with dichloromethane, dried over Na_2SO_4, filtered and concentrated under reduced pressure. The residue was purified by silica column chromatography (dichloromethane / cyclohexane 4:1→8:1) to yield the product as a yellow solid (73.6 mg, 97%). TLC (dichloromethane / cyclohexane 3:1) R_f = 0.19; ^{1}H NMR ($CDCl_3$, 500 MHz): δ (ppm) =7.54 (dd, J = 7.6, 2.0 Hz, 2 H, 2 C**H**$_{aromatic}$C-C≡C), 7.43–7.28 (m, 3 H, 3 C**H**$_{aromatic}$), 7.14–6.90 (m, 10 H, 10 C**H**$_{aromatic}$CO), 4.55 (hept, J = 6.1 Hz, 2 H, 2 C**H(**CH_3)$_2$)), 4.40-4.22 (m, 2 H, 2 C**H**CH_2), 4.01 (dt, J = 7.9, 6.4 Hz, 8 H, 4 C**H₂**O), 3.90 (s, 6 H, 2 C**H₃**O), 3.35 (s, 1 H, 1 C≡C-**H**), 2.07–1.95 (m, 4 H, 4 C**H**$_{equatorial}$CHO), 1.85 (ddp, J = 21.5, 14.4, 6.9 Hz, 12 H, 4 C**H**$_{equatorial}$$CH_2$CHO, 2 C**H**$_2$$CH_2$O, 2 C**H**$_2$$CH_3$), 1.76–1.62 (m, 4 H, 2 C**H**$_2$$CH_2$$CH_2$O), 1.58–1.46 (m, 6 H, 4 C**H**$_{axial}$CHO, 2 C**H**$_{equatorial}$$CH_2$$CH_2$CHO), 1.41–1.19 (m, 34 H, 4 C**H**$_3$CH, 4 C**H**$_{axial}$$CH_2$CHO, 2 C**H**$_{axial}$$CH_2$$CH_2$CHO, 8 C**H**$_2$), 1.11, 1.10 (2 t, J = 7.3 Hz, 6 H, 2 C**H**$_3$$CH_2$), 0.88 (dt, J = 13.0, 6.8 Hz, 6 H, 2 C**H**$_3$(CH_2)$_n$); ^{13}C NMR ($CDCl_3$, 125 MHz): δ (ppm) = 154.23, 153.97, 153.91, 153.71, 153.60, 152.88, 152.86, 152.78, 152.64, 131.69, 128.46, 128.39, 123.59, 121.20, 121.04, 120.96, 120.70, 117.80, 117.40, 117.21, 116.94, 116.43, 116.33, 116.24, 115.99, 115.69, 115.68, 114.68, 114.38, 114.19, 113.88, 113.54, 112.75, 95.01, 92.32, 91.83, 91.77, 91.68, 91.61, 91.50, 91.43, 91.19, 86.09, 82.53, 80.17, 77.94, 77.87, 73.20, 73.16, 71.27, 71.23, 69.73, 69.72, 56.51, 56.45, 31.97, 31.94, 31.87, 29.53, 29.45, 29.42, 29.37, 29.29, 26.07, 26.06, 25.91, 25.85, 23.65, 23.54, 22.86, 22.83, 22.80, 22.43, 22.39, 14.26, 14.23, 10.77, 10.72; ESI-MS of $C_{90}H_{106}O_{10}$ (M+H$^+$ = 1347.78); IR (ATR) ν = 3276.5, 2925.8, 2853.4, 1596.0, 1486.5, 1465.2, 1413.2, 1383.2, 1272.4, 1208.0, 1106.4, 1038.1, 962.9, 860.9, 754.5, 689.4, 528.1 cm^{-1}.

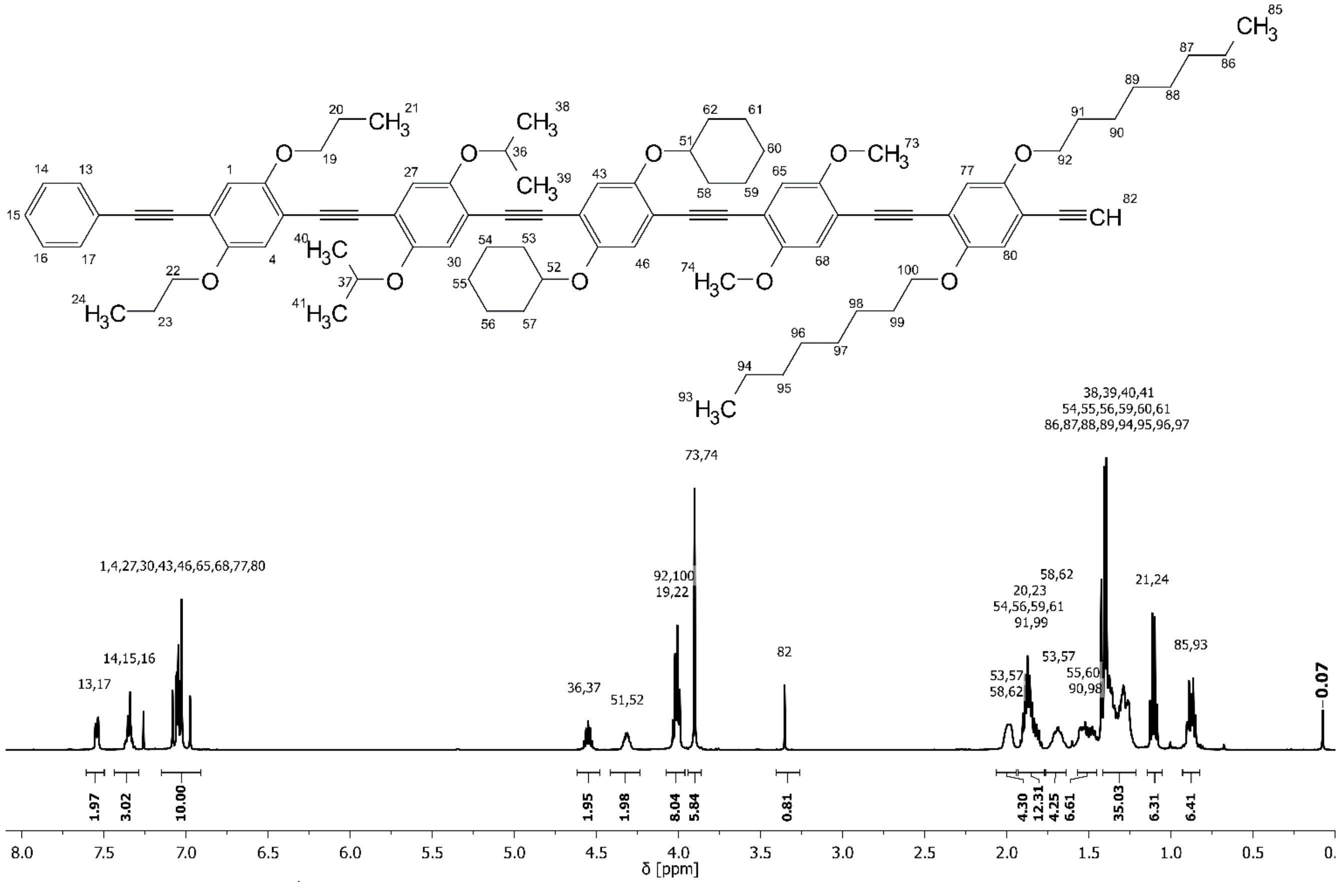

Supplementary Figure 70: ^{1}H NMR spectrum of sequence-defined, deprotected pentamer **21** with assigned signals.

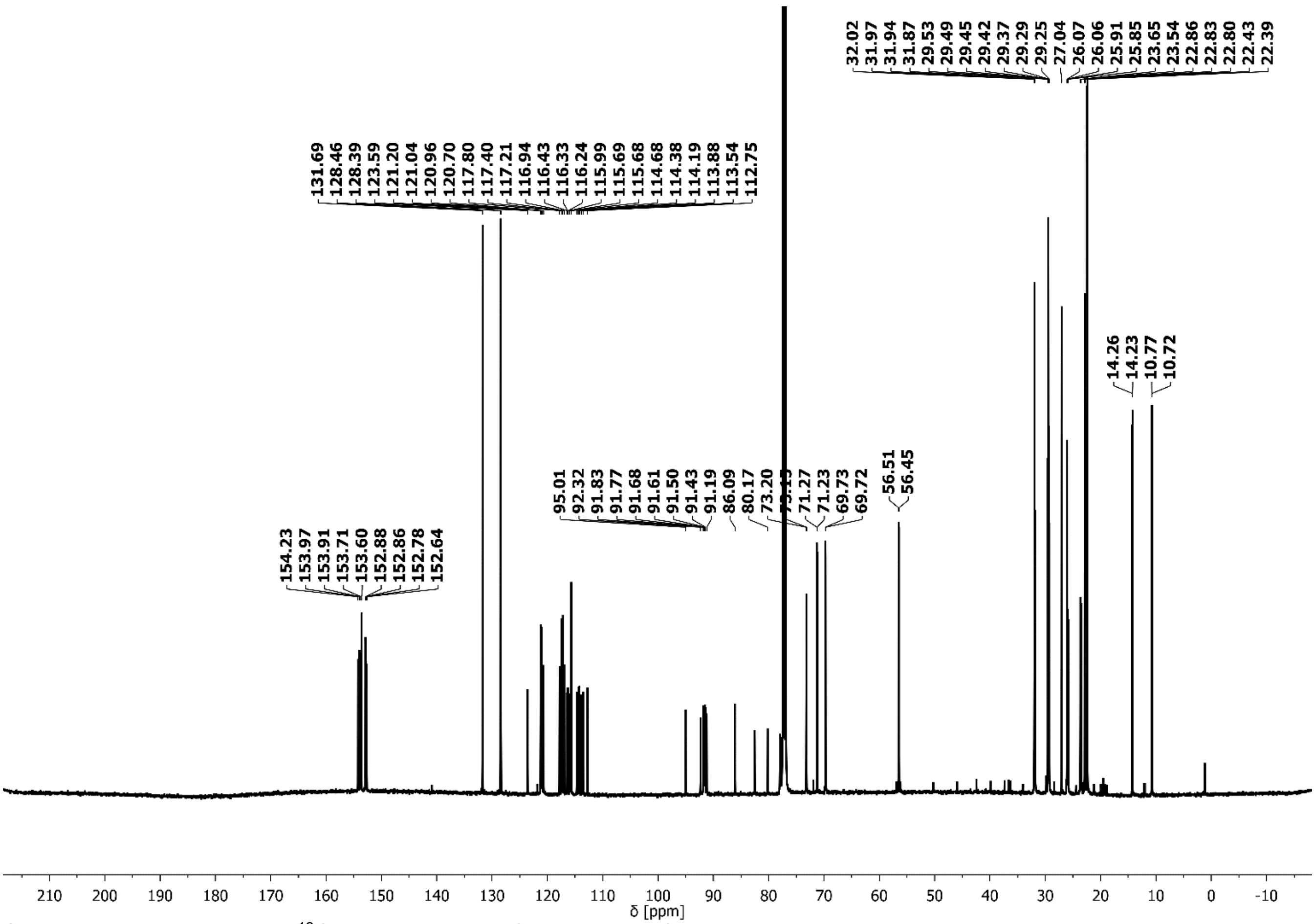

Supplementary Figure 71: ^{13}C NMR spectrum of sequence-defined, deprotected pentamer **21**.

1.8 Synthesis of Sequence-Defined Trimers with one Fluorene Unit

Synthesis of trimethyl((7-(phenylethynyl)-*9H*-fluoren-2-yl)ethynyl)silane **26**

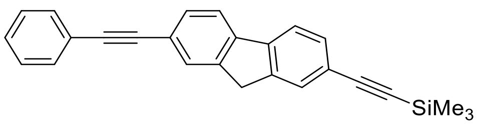

7-Iodo-2-trimethylsilylacetylene-9*H*-fluorene **22** (551 mg, 1.42 mmol, 1.00 eq.), 2.5 mol% bis(triphenylphosphine)palladium(II) dichloride (25.0 mg, 0.0356 mmol) and 5 mol% copper(I)iodide (13.4 mg, 0.0704 mmol) were placed into a Schlenk flask and degassed. Under continuous argon flow, 20 mL dry THF and 1.93 mL dry triethylamine (1.42 g. 14.3 mmol, 10.0 eq.) were added and the mixture was stirred for 10 minutes. Subsequently, 0.46 mL phenylacetylene (430 mg, 4.21 mmol, 3.00 eq.) was added dropwise with a syringe. The reaction mixture was stirred for 48 h at room temperature, taken up in dichloromethane and washed with saturated NH_4Cl solution. The aqueous phase was extracted three times with dichloromethane. The combined organic layers were dried over Na_2SO_4, filtered and concentrated under reduced pressure. The residue was purified by silica column chromatography (cyclohexane / dichloromethane 4:1 and cyclohexane / ethyl acetate 20:1) to yield the product as white yellow solid (468 mg, 92%); TLC (cyclohexane / dichloromethane 4:1) R_f = 0.61; ^{1}H NMR (300 MHz, $CDCl_3$): δ (ppm) = 7.74–7.66 (m, 4 H, 2 C$\mathbf{H}_{\mathbf{aromatic}}$CHC-C≡C, 2 C$\mathbf{H}_{\mathbf{aromatic}}$C-C≡C), 7.57–7.50 (m, 4 H, 2 C$\mathbf{H}_{\mathbf{aromatic}}$CH, 2 C$\mathbf{H}_{\mathbf{aromatic}}$ benzene end unit), 7.36–7.35 (m, 3 H, 3 C$\mathbf{H}_{\mathbf{aromatic}}$ benzene end unit), 3.89 (s, 2 H, CC$\mathbf{H_2}$C), 0.27 (s, 9 H, 3 C$\mathbf{H_3}$Si); ^{13}C NMR (75 MHz, $CDCl_3$): δ (ppm) = 143.70, 143.49, 141.52, 141.29, 131.73, 131.16, 130.79, 128.71, 128.51, 128.36, 128.30, 123,51, 121.95, 121.72, 120.25, 120.07, 105.87, 94.59, 90.19, 89.91, 36.65, 0.19; HRMS (FAB) of $C_{26}H_{22}Si[M+H^+]$ calc. 362.1487, found 362.1485; IR (ATR) v = 3056.4, 2955.5, 2896.2, 2180.1, 2141.4, 1595.4, 1490.4, 1464.5, 1551.7, 1415.7, 1397.5, 1279.3, 1246.2, 1217.2, 1196.6, 1135.9, 1069.9, 1024.6, 929.8, 836.7, 820.9, 750.0. 689.3, 653.5, 616.7, 556.7, 537.2, 518.6, 456.0, 420.0 cm^{-1}.

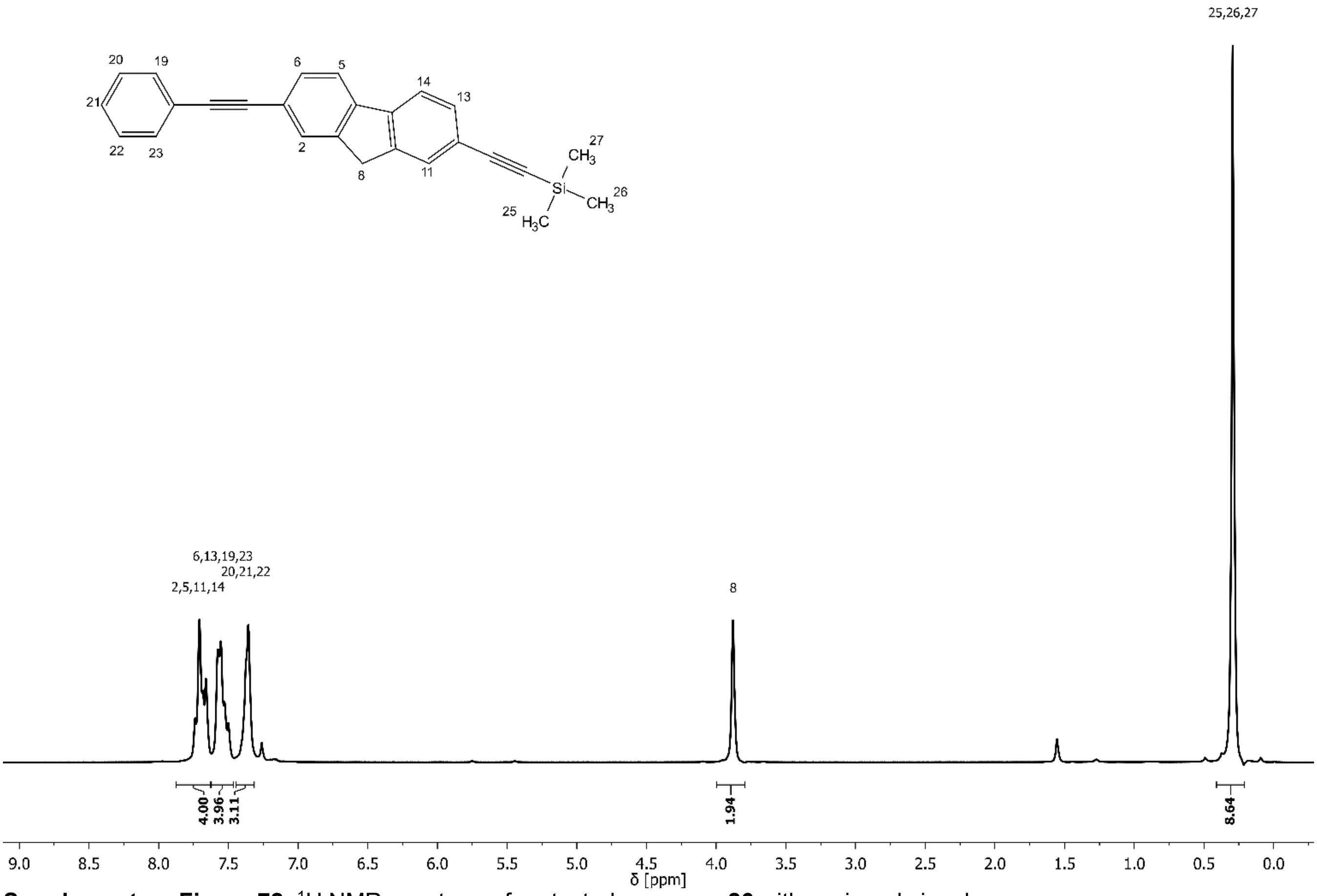

Supplementary Figure 72: ^{1}H NMR spectrum of protected monomer **26** with assigned signals.

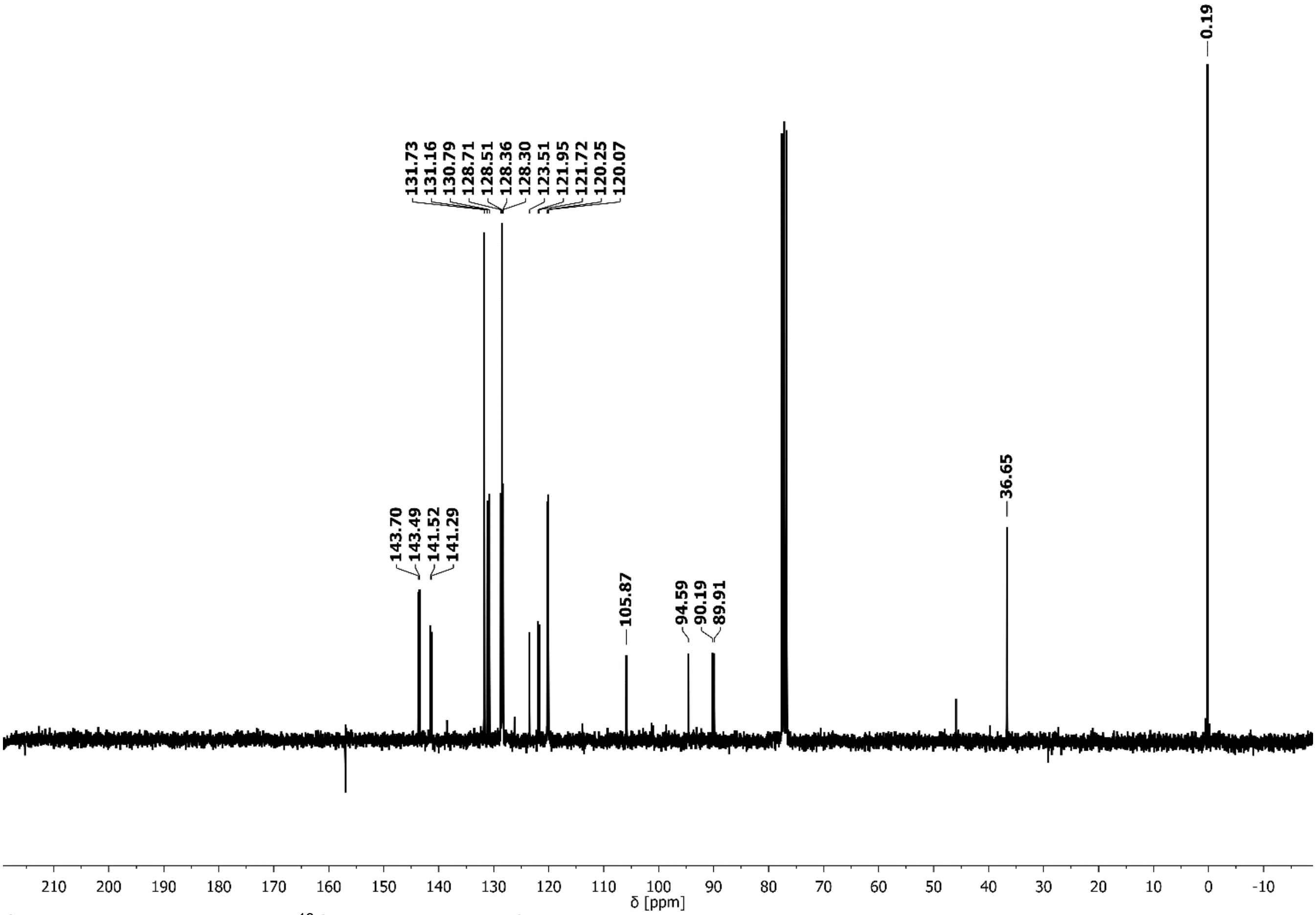

Supplementary Figure 73: ^{13}C NMR spectrum of protected monomer **26**.

Synthesis of 2-ethynyl-7-(phenylethynyl)-*9H*-fluorene **27**

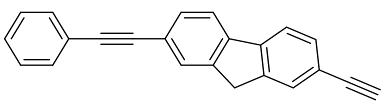

Compound **26** (470 mg, 1.30 mmol, 1.00 eq.) and two equivalents of potassium carbonate (360 mg, 2.60 mmol) were placed into a Schlenk flask and degassed three times. Under continuous argon flow 25 mL dry dichloromethane and 25 ml dry methanol were added. The reaction mixture was stirred overnight at room temperature under argon atmosphere and quenched with distilled water. The aqueous phase was extracted three times with dichloromethane, dried over Na_2SO_4, filtered and concentrated under reduced pressure. The residue was purified by flash silica column chromatography (cyclohexane / ethyl acetate 20:1) to yield the product as a light yellow solid (368 mg, 98%); TLC (cyclohexane / dichloromethane 4:1) R_f = 0.44; ^{1}H NMR (300 MHz, $CDCl_3$): δ (ppm) = 7.75–7.65 (m, 4 H, 2 C$\mathbf{H}_{\mathbf{aromatic}}$CHC-C≡C, 2 C$\mathbf{H}_{\mathbf{aromatic}}$C-C≡C), 7.58–7.52 (m, 4 H, 2 C$\mathbf{H}_{\mathbf{aromatic}}$CH, 2 C$\mathbf{H}_{\mathbf{aromatic}}$ benzene end unit), 7.41–7.36 (m, 3 H, 3 C$\mathbf{H}_{\mathbf{aromatic}}$ benzene end unit), 3.89 (s, 2 H, CC$\mathbf{H}_2$C), 3.14 (s, 1 H, 1 C≡C-**H**); ^{13}C NMR (75 MHz, $CDCl_3$): δ (ppm) = 143.68, 143.58, 141.83, 141.16, 131.29, 130.80, 128.86, 128.51, 128.37, 128.32, 123.49, 122.07, 120.64, 120.29, 120.14, 90.15, 89.96, 84.39, 77.48, 36.65; HRMS (FAB) of $C_{23}H_{24}[M+H^+]$ calc. 290.1096, found 290.1097; IR (ATR) ν = 3304.3, 2923.4, 2101.5, 1670.7, 1488.0, 1462.2, 1441.1, 1415.2, 1392.2, 1328.8, 1278.6, 1175.2, 1070.7, 1002.0, 958.2, 939.4, 917.7, 863.8, 849.7, 822.3, 754.6, 692.6, 643.0, 609.31, 594.32, 553.0, 525.0, 488.2, 453.3, 423.3 cm^{-1}.

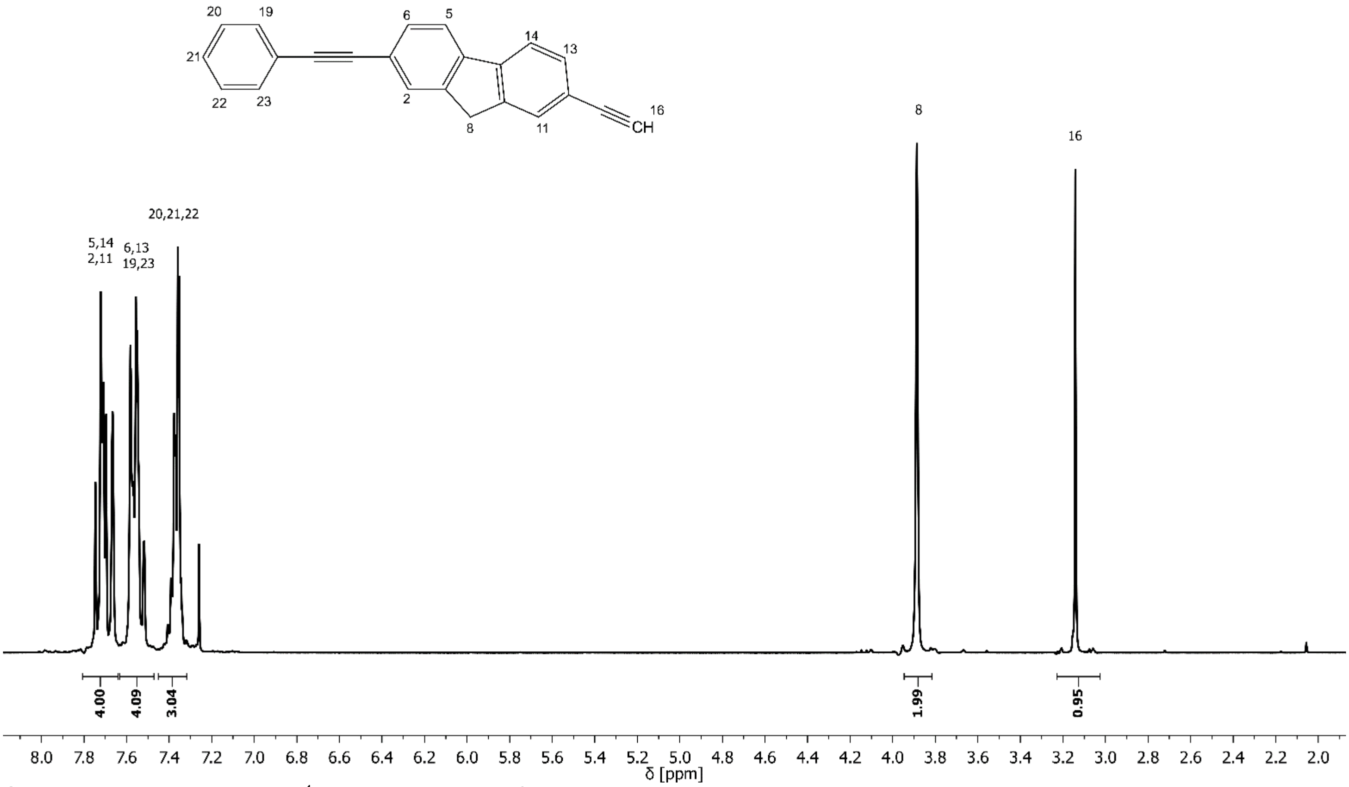

Supplementary Figure 74: ^{1}H NMR spectrum of deprotected monomer **27** with assigned signals.

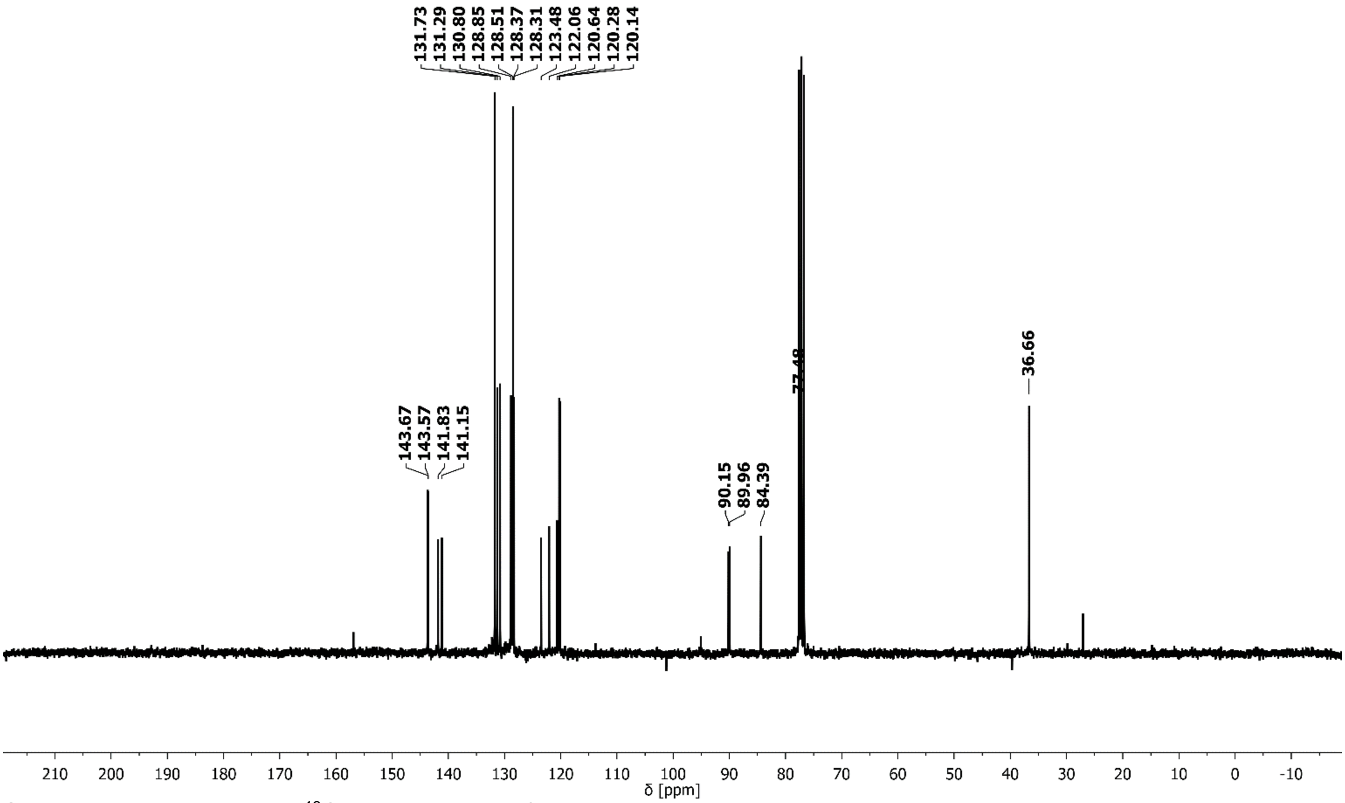

Supplementary Figure 75: ^{13}C NMR spectrum of deprotected monomer **27**.

Synthesis of trimethyl((4-((7-(phenylethynyl)-*9H*-fluoren-2-yl)ethynyl)-2,5-dipropoxyphenyl)ethynyl) silane **28**

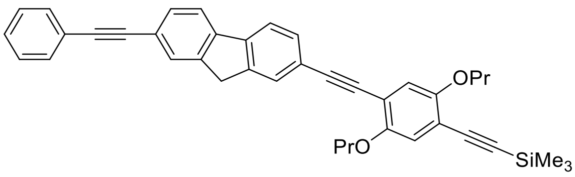

Compound **27** (360 mg, 1.23 mmol, 1.00 eq.), 5 mol% bis(triphenylphosphine)palladium(II)dichloride (45.6 mg, 0.0650 mmol), 5 mol% copper(I)iodide (12.4 mg, 0.0651 mmol) and 1,4-Bis(propyloxy)-2-iodo-5-trimethylsilylacetylenebenzene **1** (1.54 g, 3.69 mmol, 3.00 eq.) were placed into a Schlenk flask and degassed. Under continuous argon flow, 50 mL dry THF and 1.7 mL dry triethylamine (1.24 g. 12.3 mmol, 10.0 eq.) were added and the mixture was stirred for 72 h at 45 °C. The reaction mixture was taken up in dichloromethane and washed with saturated NH_4Cl solution. The aqueous phase was extracted three times with dichloromethane. The combined organic layers were dried over Na_2SO_4, filtered and concentrated under reduced pressure. The residue was purified by silica column chromatography twice (cyclohexane / dichloromethane 4:1 → 2:1 and cyclohexane / ethyl acetate 20:1) to yield the product as yellow solid (421 mg, 60%); TLC (cyclohexane / dichloromethane 2:1) R_f = 0.45; ^{1}H NMR (400 MHz, $CDCl_3$): δ (ppm) = 7.76–7.70 (m, 4 H, 2 C**H**$_{\mathbf{aromatic}}$CHC-C≡C, 2 C**H**$_{\mathbf{aromatic}}$C-C≡C), 7.58–7.55 (m, 4 H, 2 C**H**$_{\mathbf{aromatic}}$CH, 2 C**H**$_{\mathbf{aromatic}}$ benzene end unit), 7.40–7.33 (m, 3 H, 3 C**H**$_{\mathbf{aromatic}}$ benzene end unit), 7.00, 6.97 (2 s, 2 H, 2 C**H**$_{\mathbf{aromatic}}$CO), 4.09 – 3.95 (m, 4 H, 2 C**H**$_{\mathbf{2}}$O), 3.92 (s, 2 H, CC**H**$_{\mathbf{2}}$C), 1.95–1.80 (m, 4 H, 2 C**H**$_{\mathbf{2}}$$CH_3$), 1.15–1.07 (m, 6 H, 2 C**H**$_{\mathbf{3}}$), 0.28 (s, 9 H, 3 C**H**$_{\mathbf{3}}$Si); ^{13}C NMR (100 MHz, $CDCl_3$): δ (ppm) = 154.33, 153.58, 143.67, 143.64, 141.34, 131.71, 130.80, 128.50, 128.29, 128.21, 120.21, 117.41, 117.09, 101.30, 100.25, 95.76, 90.21, 89.91, 86.45, 77.16, 71.20, 71.16, 39.69, 22.87, 22.85, 10.73, 10.67, 0.10; HRMS (FAB) of $C_{40}H_{38}O_2Si$[M+H$^+$] calc. 578.2641, found 578.2643; IR (ATR) ν = 2960.6. 2872.4, 2149.2, 1594.9, 1502.4, 1471.9, 1441.8, 1406.9, 1388.5, 1274.7, 1247.7, 1215.7, 1195.2, 1218.3, 1045.2, 1025.5, 925.1, 871.3, 838.5, 822.1, 751.4, 689.9, 631.7, 551.9, 524.3, 475.7, 422.4. cm^{-1}.

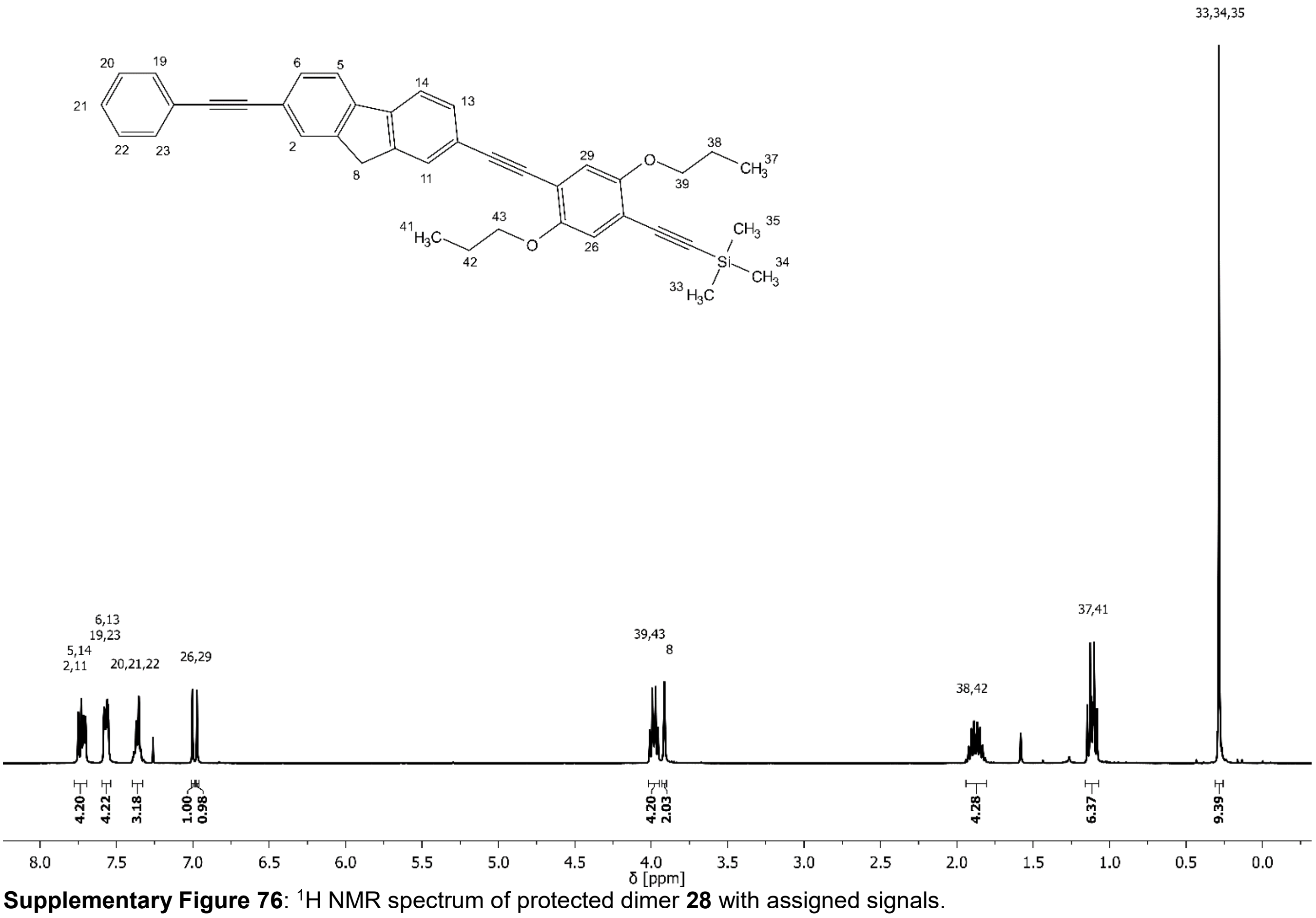

Supplementary Figure 76: ^{1}H NMR spectrum of protected dimer **28** with assigned signals.

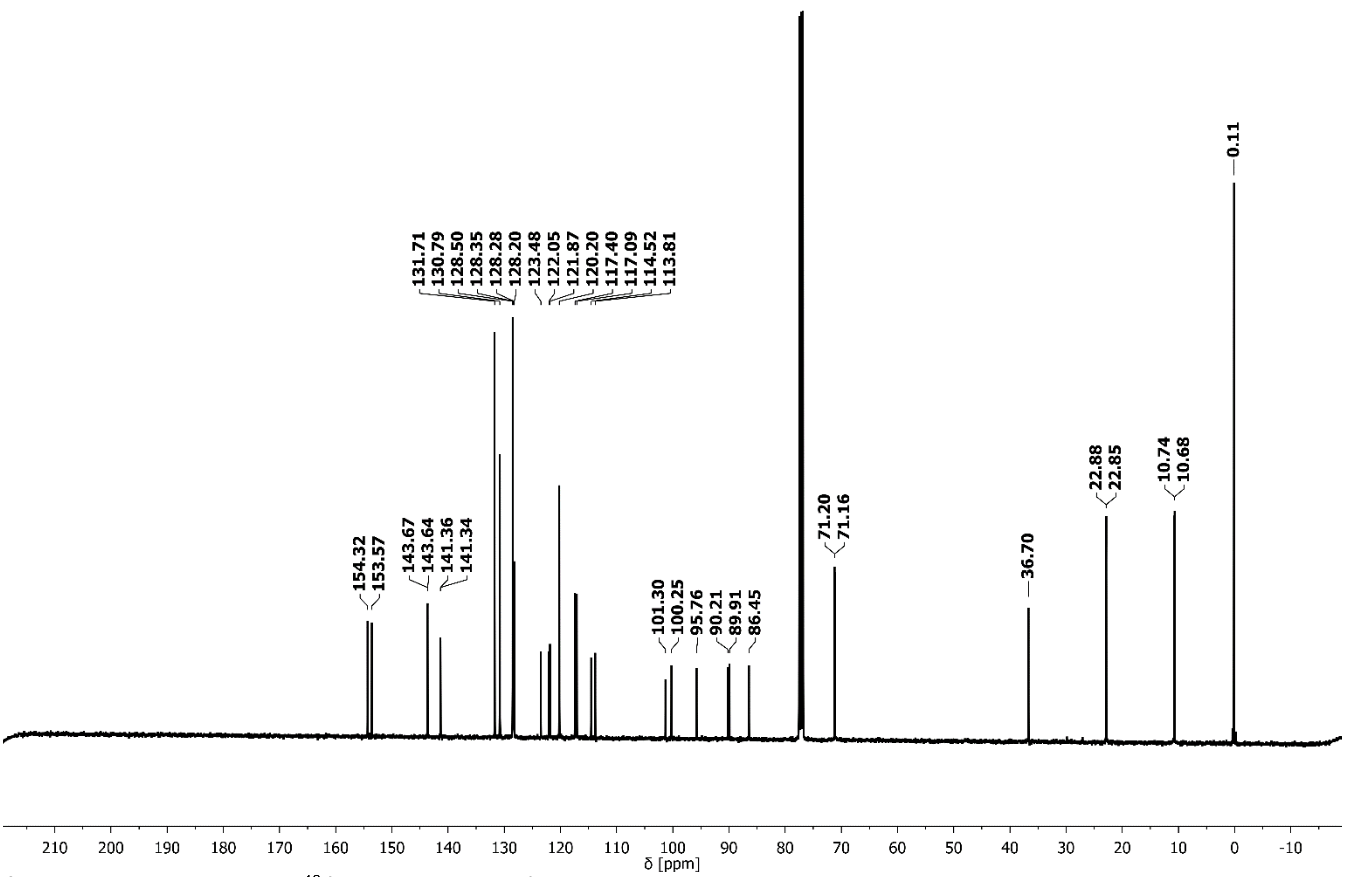

Supplementary Figure 77: ^{13}C NMR spectrum of protected dimer **28**.

Synthesis of 2-((4-ethynyl-2,5-dipropoxyphenyl)ethynyl)-7-(phenylethynyl)-*9H*-fluorene **29**

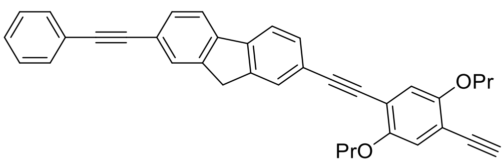

Compound **28** (377 mg, 0.651 mmol, 1.00 eq.) and two equivalents of potassium carbonate (180 mg, 1.30 mmol) were placed into a Schlenk flask and degassed three times. Under continuous argon flow 20 mL dry dichloromethane and 20 ml dry methanol were added. The reaction mixture was stirred overnight at room temperature under argon atmosphere and quenched with distilled water. The aqueous phase was extracted three times with dichloromethane, dried over Na_2SO_4, filtered and concentrated under reduced pressure. The product was obtained as a yellow solid (315 mg, 95%); TLC (cyclohexane / dichloromethane 2:1) R_f = 0.40; ^{1}H NMR (400 MHz, $CDCl_3$): δ (ppm) = 7.76–7.71 (m, 4 H, 2 C**H**$_{\mathbf{aromatic}}$CHC-C≡C, 2 C**H**$_{\mathbf{aromatic}}$C-C≡C),7.58–7.55 (m, 4 H, 2 C**H**$_{\mathbf{aromatic}}$CH, 2 C**H**$_{\mathbf{aromatic}}$ benzene end unit),7.39–7.34 (m, 3 H, 3 C**H**$_{\mathbf{aromatic}}$ benzene end unit), 7.02, 6.99 (2 s, 2 H, 2 C**H**$_{\mathbf{aromatic}}$CO), 4.02–3.97 (m, 4 H, 2 C**H**$_\mathbf{2}$O), 3.93 (s, 2 H, CC**H**$_\mathbf{2}$C), 3.35 (s, 1 H, 1 C≡C-**H**), 1.95–1.80 (m, 4 H, C**H**$_\mathbf{2}$$CH_3$), 1.14–1.05 (m, 6 H, 2 C**H**$_\mathbf{3}$); ^{13}C NMR (100 MHz, $CDCl_3$): δ (ppm) = 154.31, 153.57, 143.70, 143.67, 141.45, 131.73, 130.83, 128.51, 128.37, 128.31, 128.25, 121.93, 120.23, 118.02, 117.04, 95.81, 90.21, 89.94, 86.28, 82.43, 80.18, 71.29, 71.25, 36.72, 22.87, 22.72, 10.72, 10.62; HRMS (FAB) of$C_{37}H_{30}O_2$[M+H$^+$] calc. 507.2324, found 507.2325; IR (ATR) v = 3298.3, 3283.9, 2958.1, 2908.0, 2871.3, 2202.9, 1595.4, 1500.3, 1472.1, 1416.6, 1389.6, 1274.9, 1213.4, 1193.2, 1125.9, 1041.0, 1020.1, 937.4, 906.9, 861.5, 824.6, 775.0, 756.7, 715.1, 689.2, 651.1, 621.2, 604.7, 523.5, 482.2, 442.8, 428.9 cm^{-1}.

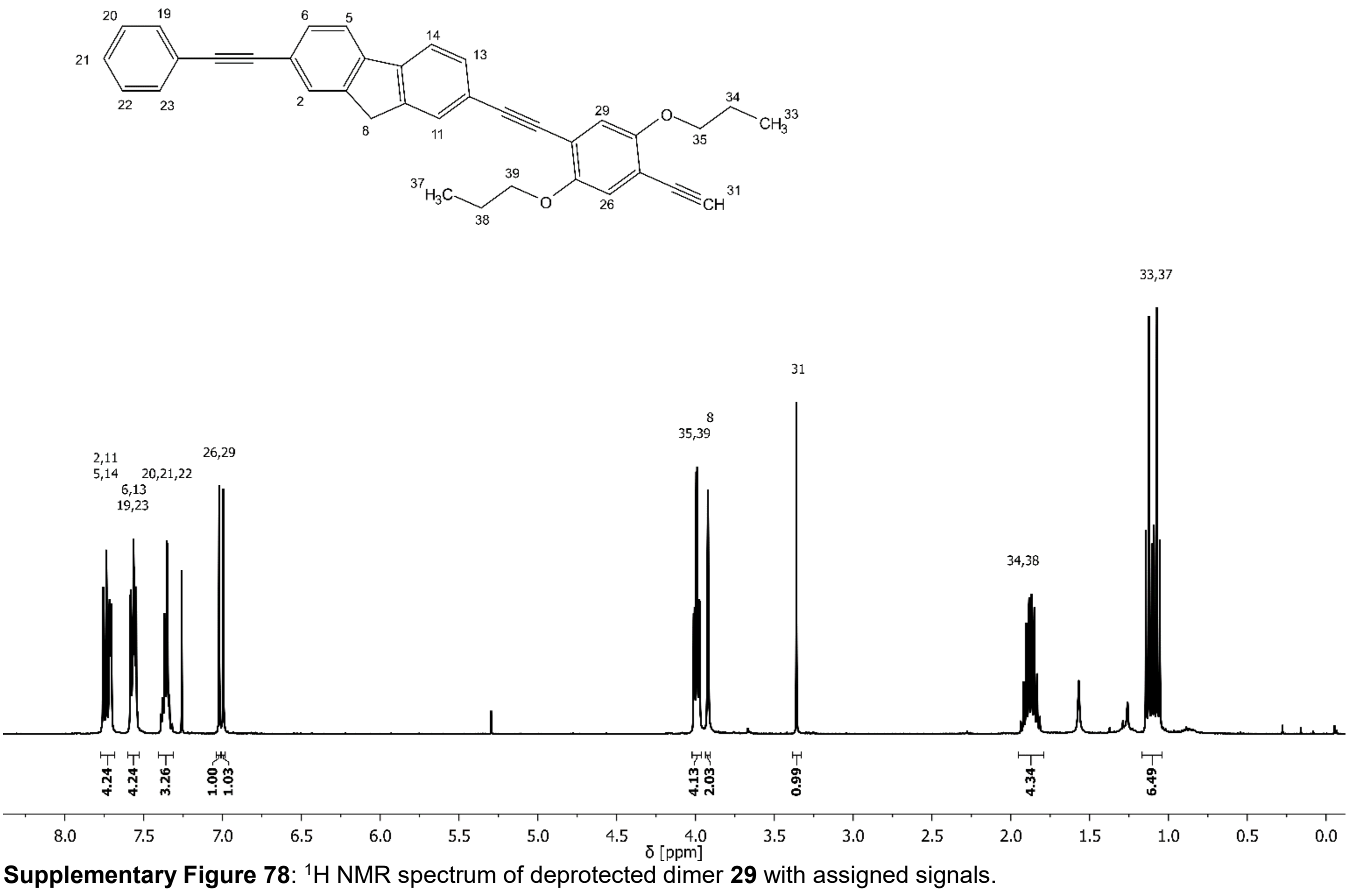

Supplementary Figure 78: ^{1}H NMR spectrum of deprotected dimer **29** with assigned signals.

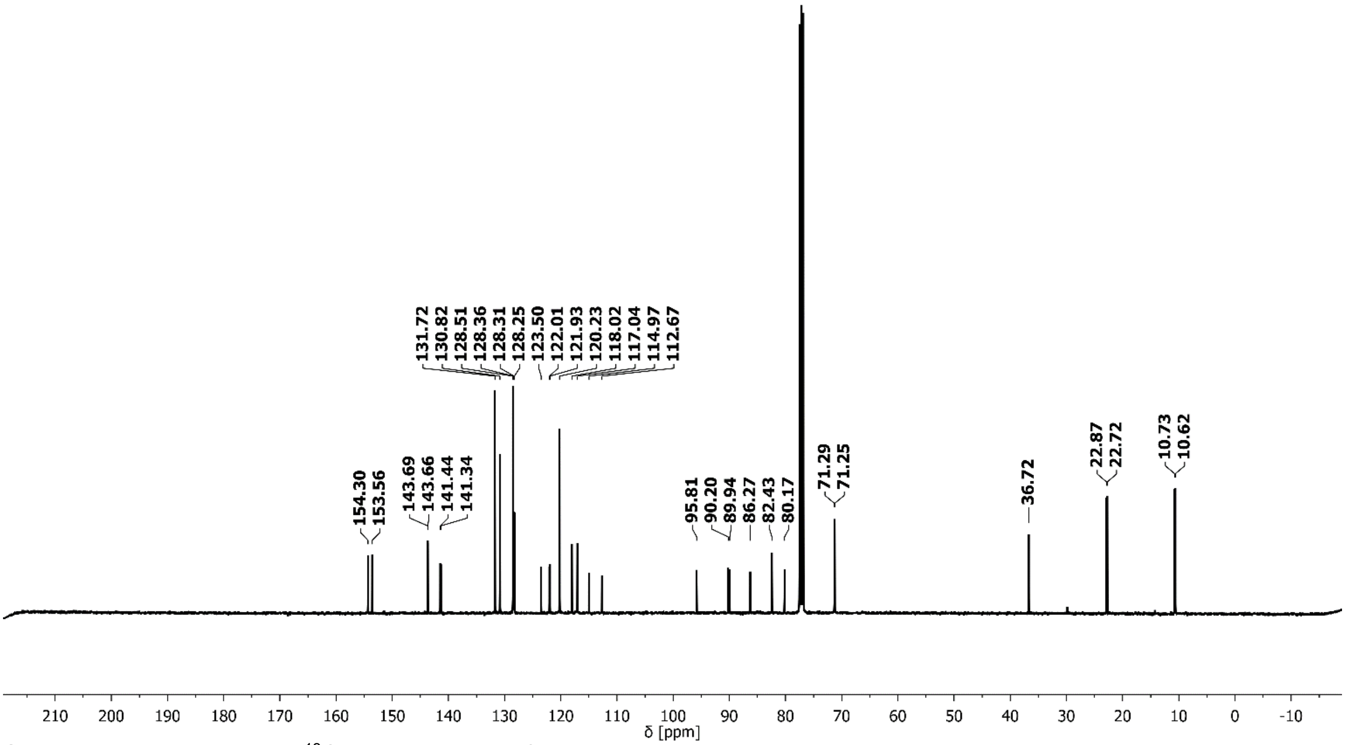

Supplementary Figure 79: ^{13}C NMR spectrum of deprotected dimer **29**.

Synthesis of trimethyl((7-((4-(phenylethynyl)-2,5-dipropoxyphenyl)ethynyl)-*9H*-fluoren-2-yl)ethynyl) silane **30**

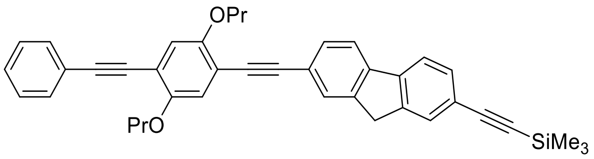

Compound **3** (414 mg, 1.30 mmol, 1.00 eq.), 5 mol% bis(triphenylphosphine)palladium(II)dichloride (45.6 mg, 65.0 µmol), 5 mol% copper(I)iodide (12.4 mg, 65.0 µmol) and 7-Iodo-2-trimethylsilylacetylene-9*H*-fluorene **22** (1.51 g, 3.90 mmol, 3.00 eq.) were placed into a Schlenk flask and degassed. Under continuous argon flow, 50 mL dry THF and 1.8 mL dry triethylamine (1.32 g. 13.0 mmol, 10.0 eq.) were added and the mixture was stirred for 72 h at 45 °C. The reaction mixture was taken up in dichloromethane and washed with saturated NH_4Cl solution. The aqueous phase was extracted three times with dichloromethane. The combined organic layers were dried over Na_2SO_4, filtered and concentrated under reduced pressure. The residue was purified by silica column chromatography twice (cyclohexane / dichloromethane 4:1 → 2:1 and cyclohexane / ethyl acetate 20:1) to yield the product as lightly yellow solid (482 mg, 64%); TLC (cyclohexane / dichloromethane 2:1) R_f = 0.45; ^{1}H NMR (400 MHz, $CDCl_3$): δ (ppm) = 7.75–7.66 (m, 4 H, 2 C**H**$_{\textbf{aromatic}}$CHC-C≡C, 2 C**H**$_{\textbf{aromatic}}$C-C≡C),7.59–7.49 (m, 4 H, 2 C**H**$_{\textbf{aromatic}}$CH, 2 C**H**$_{\textbf{aromatic}}$ benzene end unit),7.40–7.26 (m, 3 H, 3 C**H**$_{\textbf{aromatic}}$ benzene end unit), 7.05, 7.04 (2 s, 2 H, 2 C**H**$_{\textbf{aromatic}}$CO), 4.04–4.00 (m, 4 H, C**H**$_\textbf{2}$O), 3.89 (s, 2 H, CC**H**$_\textbf{2}$C), 1.97–1.84 (m, 4 H, 2 C**H**$_\textbf{2}$$CH_3$), 1.16–1.10 (m, 6 H, 2 C**H**$_\textbf{3}$), 0.28 (s, 9 H, 3 C**H**$_\textbf{3}$Si); ^{13}C NMR (100 MHz, $CDCl_3$): δ (ppm) = 153.78, 153.74, 143.69, 143.50, 141.30, 131.71, 130.79, 128.69, 128.47, 128.21, 120.26, 120.07, 117.12, 105.86, 95.70, 95.01, 94.59, 86.55, 86.10, 71.26, 36.66, 22.89, 10.75, 10.73, 0.17; HRMS (FAB) of $C_{40}H_{38}O_2Si$[M+H$^+$] calc. 578.2641, found 578.2639; IR (ATR) ν = 2960.3, 2934.6, 2875.2, 2149.4, 1595.6, 1530.0, 1505.3, 1489.2, 1464.9, 1443.4, 1411.7, 1388.2, 1280.5, 1247.4, 1214.5, 1120.6, 1066.7, 1021.2, 985.5, 930.0, 855.3, 839.6, 813.2, 755.5, 701.7, 687.9, 656.9, 610.0, 551.4, 524.1, 457.2, 426.1 cm^{-1}.

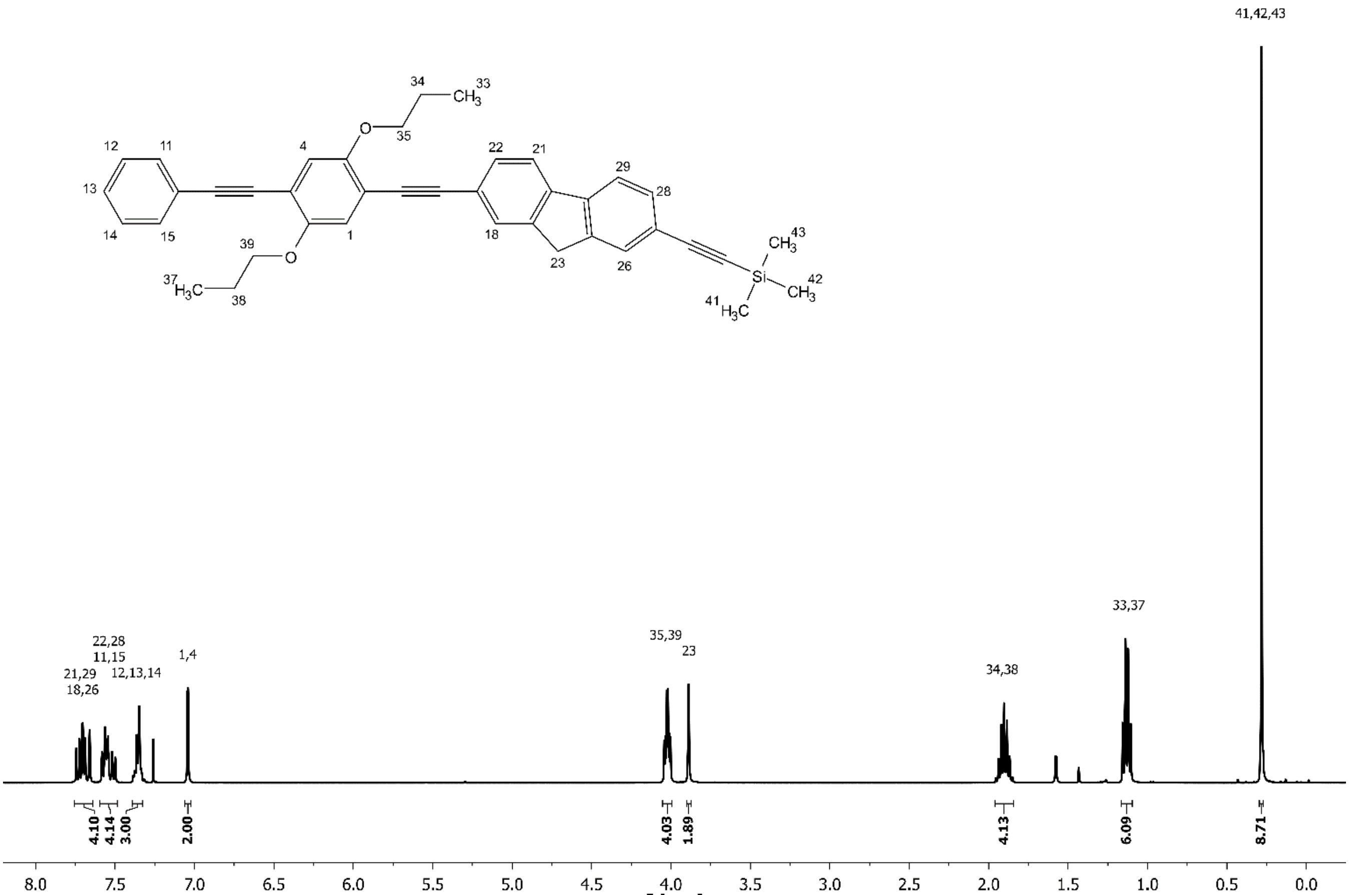

Supplementary Figure 80: ^{1}H NMR spectrum of protected dimer **30** with assigned signals.

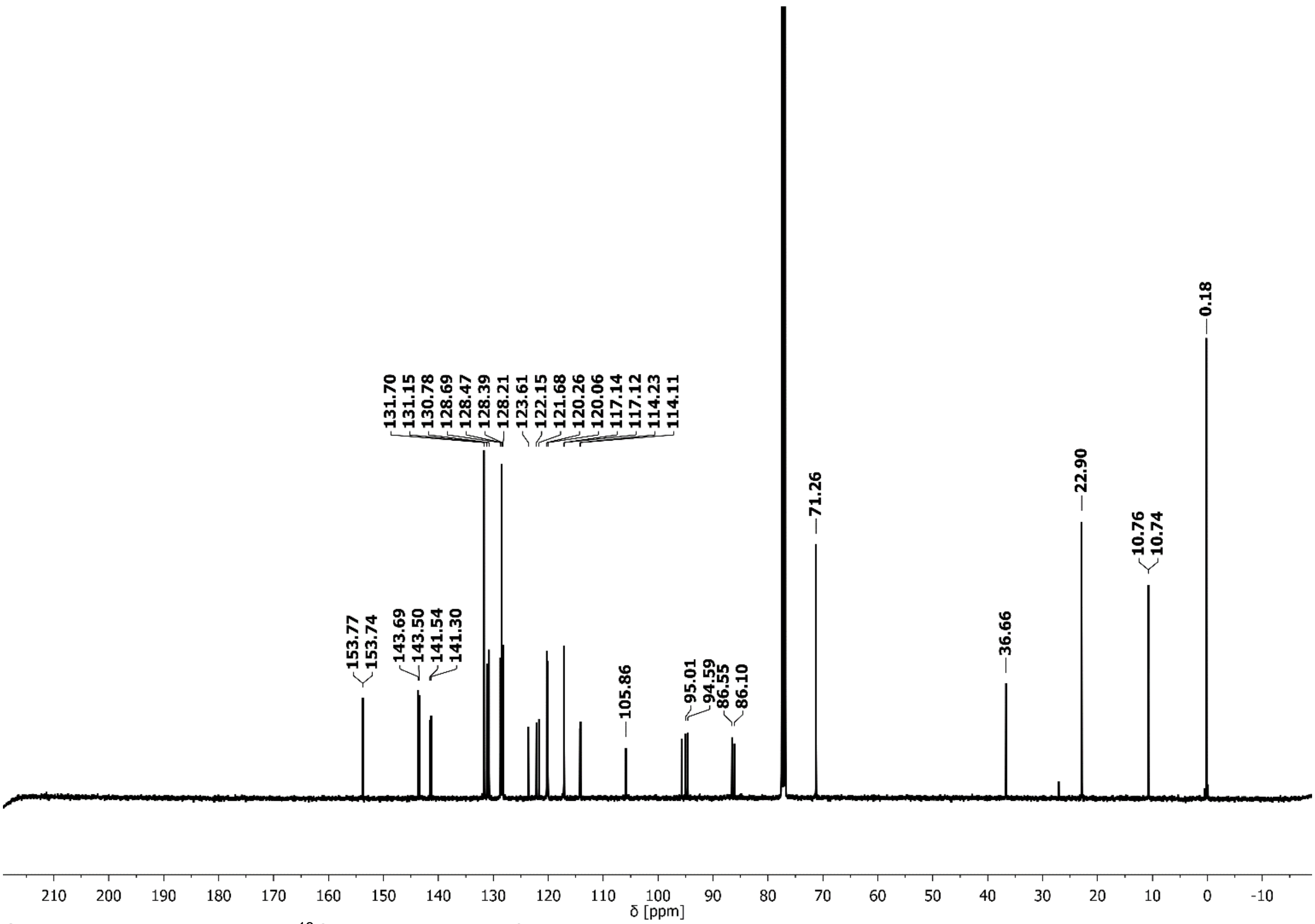

Supplementary Figure 81: ^{13}C NMR spectrum of protected dimer **30**.

Synthesis of 2-ethynyl-7-((4-(phenylethynyl)-2,5-dipropoxyphenyl)ethynyl)-*9H*-fluorene **31**

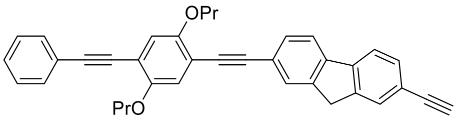

Compound **30** (443 mg, 0.765 mmol, 1.00 eq.) and two equivalents of potassium carbonate (212 mg, 1.53 mmol) were placed into a Schlenk flask and degassed three times. Under continuous argon flow 20 mL dry dichloromethane and 20 ml dry methanol were added. The reaction mixture was stirred overnight at room temperature under argon atmosphere and quenched with distilled water. The aqueous phase was extracted three times with dichloromethane, dried over Na_2SO_4, filtered and concentrated under reduced pressure. The product was obtained as an orange solid (360 mg, 93%); TLC (cyclohexane / dichloromethane 2:1) R_f = 0.40; ^{1}H NMR (400 MHz, $CDCl_3$): δ (ppm) = 7.76–7.68 (m, 4 H, 2 C**H**$_{\mathbf{aromatic}}$CHC-C≡C, 2 C**H**$_{\mathbf{aromatic}}$C-C≡C),7.59–7.52 (m, 4 H, 2 C**H**$_{\mathbf{aromatic}}$CH, 2 C**H**$_{\mathbf{aromatic}}$ benzene end unit),7.39–7.33 (m, 3 H, 3 C**H**$_{\mathbf{aromatic}}$ benzene end unit), 7.05, 7.04 (2 s, 2 H, 2 C**H**$_{\mathbf{aromatic}}$CO), 7.04 (s, 2 H, 2 C**H**$_{\mathbf{aromatic}}$CO), 4.14–4.05 (m, 4 H, 2 C**H₂**O), 3.91 (s, 2 H, CC**H₂**C), 3.14 (s, 1 H, 1 C≡CH) 1.97–1.83 (m, 4 H, 2 C**H₂**CH₃), 1.26–1.16 (m, 6 H, 2 CH₃); ^{13}C NMR (100 MHz, $CDCl_3$): δ (ppm) = 153.79, 153.76, 143.67, 143.59, 141.86, 141.17, 131.71, 131.30, 130.80, 128.85, 128.47, 128.39, 128.23, 123.63, 122.29, 120.62, 120.30, 120.14, 117.17, 114.23, 114.17, 95.64, 95.02, 86.61, 86.11, 84.39, 71.28, 71.27, 36.68, 22.89, 10.74, 10.72; HRMS (FAB) of $C_{37}H_{30}O_2$[M+H$^+$] calc. 506.2246, found 506.2244; IR (ATR) ν = 3287.8, 2962.7, 2933.6, 2903.4, 2874.1, 2101.8, 1709.6, 1595.2, 1538.3, 1505.0, 1489.7, 1469.9, 1444.1, 1410.0, 1389.7, 1269.6, 1212.6, 1071.4, 1040.8, 1019.5, 939.8, 921.7, 907.0, 860.8, 823.6, 759.8, 691.9, 642.9, 614.9, 554.0, 528.4, 463.7, 423.2 cm^{-1}.

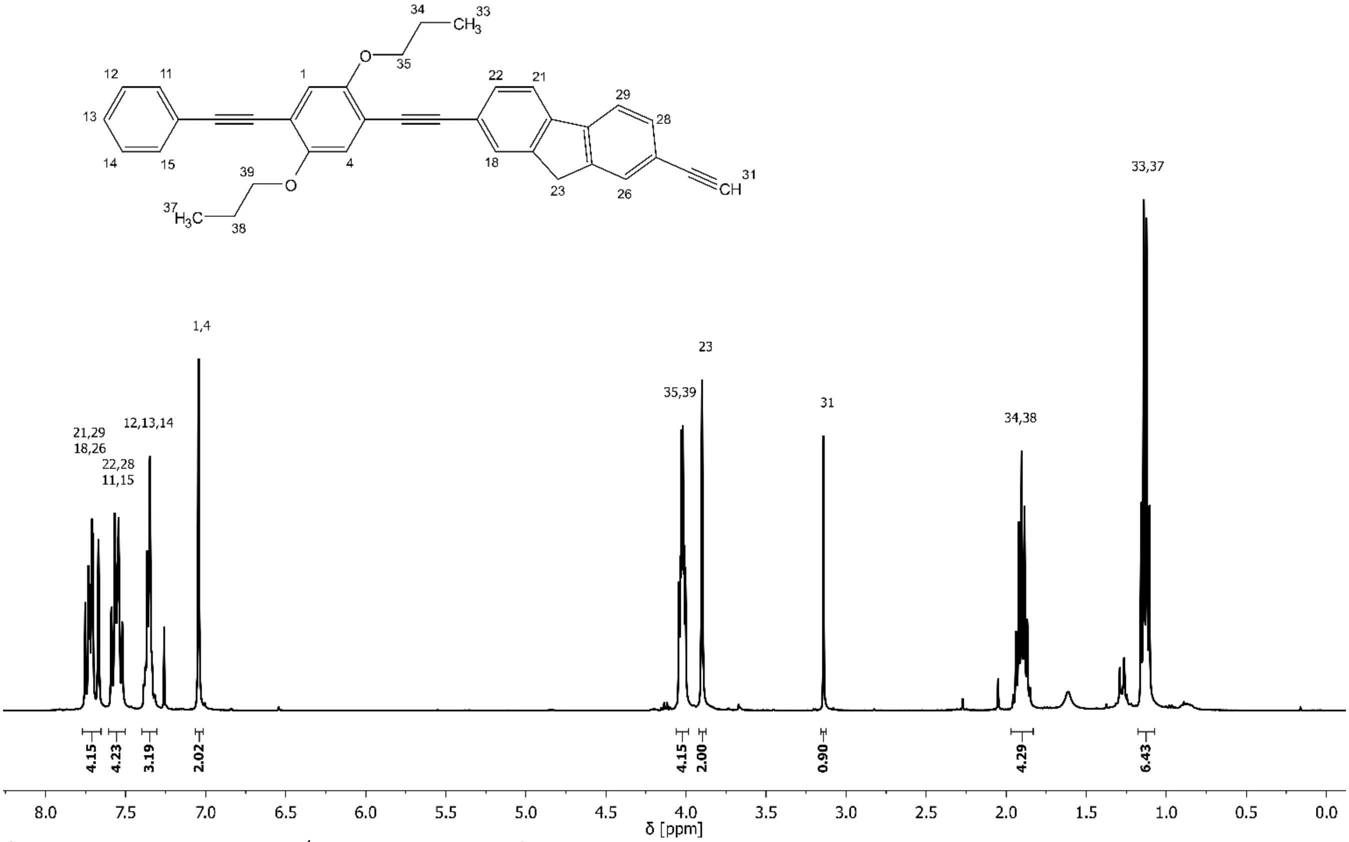

Supplementary Figure 82: ^{1}H NMR spectrum of deprotected dimer **31** with assigned signals.

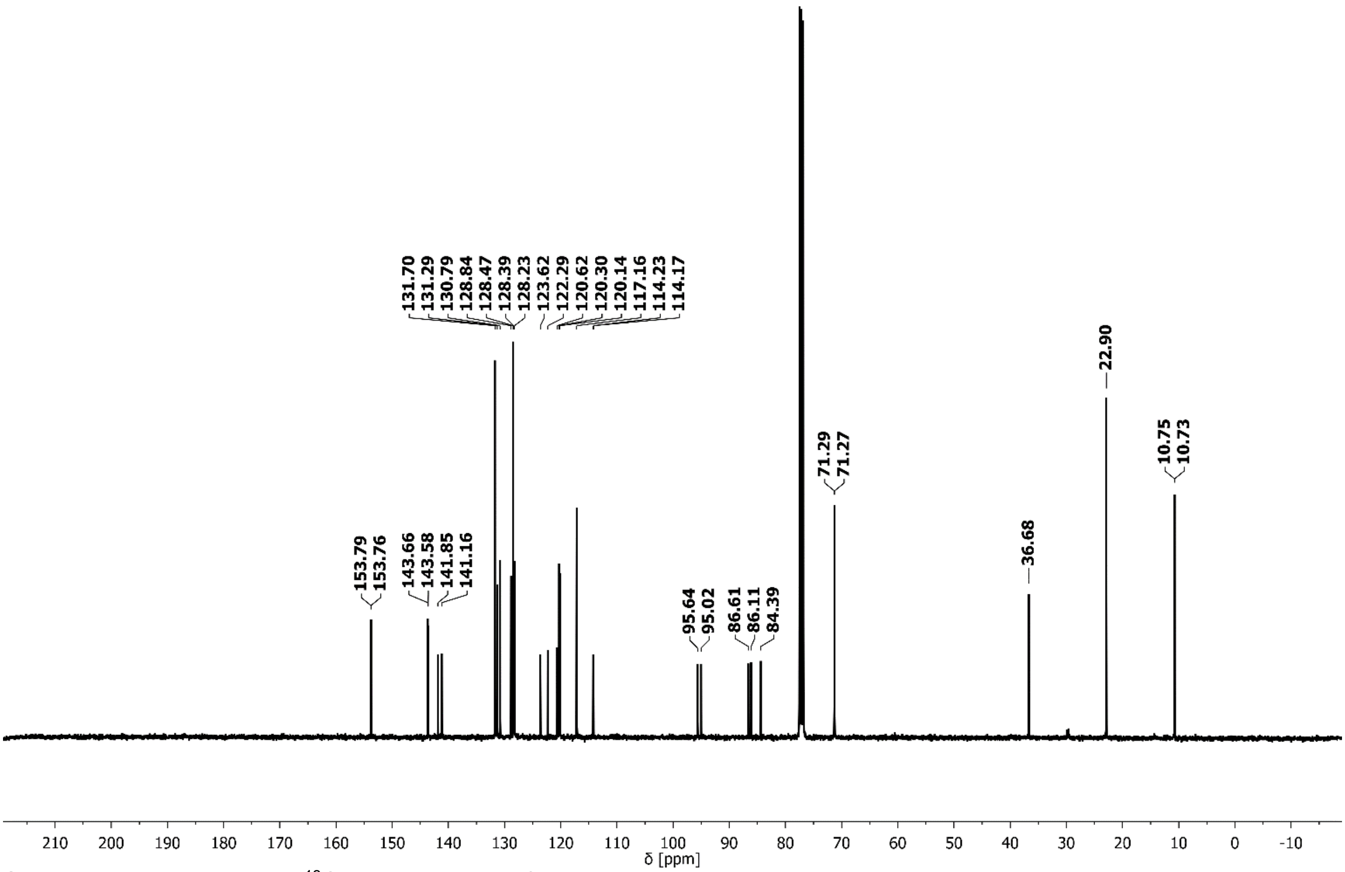

Supplementary Figure 83: ^{13}C NMR spectrum of deprotected dimer **31**.

Synthesis of trimethyl((4-((4-((7-(phenylethynyl)-*9H*-fluoren-2-yl)ethynyl)-2,5-dipropoxyphenyl)ethynyl)-2,5-dipropoxyphenyl)ethynyl)silane **23**

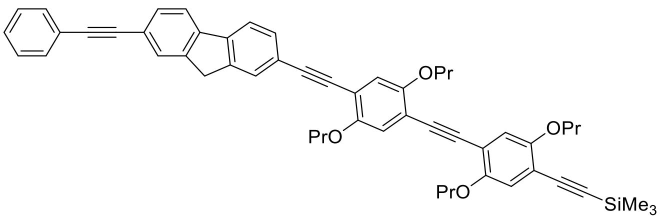

Compound **29** (288 mg, 0.577 mmol, 1.00 eq.), 5 mol% bis(triphenylphosphine)palladium(II)dichloride (19.9 mg, 28.4 µmol), 5 mol% copper(I)iodide (5.4 mg, 28.4 µmol) and 1,4-bis(propyloxy)-2-iodo-5-trimethylsilylacetylenebenzene **1** (709 mg, 1.70 mmol, 3.00 eq.) were placed into a Schlenk flask and degassed. Under continuous argon flow, 50 mL dry THF and 0.79 mL dry triethylamine (577 mg. 5.70 mmol, 10.0 eq.) were added and the mixture was stirred for 72 h at 45 °C. The reaction mixture was taken up in dichloromethane and washed with saturated NH_4Cl solution. The aqueous phase was extracted three times with dichloromethane. The combined organic layers were dried over Na_2SO_4, filtered and concentrated under reduced pressure. The residue was purified by silica column chromatography twice (cyclohexane / dichloromethane 2:1 → 3:2 and cyclohexane / ethyl acetate 20:1) to yield the product as yellow solid (188 mg, 41%); TLC (cyclohexane / dichloromethane 2:1) R_f = 0.20; ^{1}H NMR (400 MHz, $CDCl_3$): δ (ppm) = 7.80–7.67 (m, 4 H, 2 C**H**$_{\mathbf{aromatic}}$CHC-C≡C, 2 C**H**$_{\mathbf{aromatic}}$C-C≡C), 7.64–7.52 (m, 4 H, 2 C**H**$_{\mathbf{aromatic}}$CH, 2 C**H**$_{\mathbf{aromatic}}$ benzene end unit), 7.43–7.32 (m, 3 H, 3 C**H**$_{\mathbf{aromatic}}$ benzene end unit), 7.11 – 6.92 (m, 4 H, 4 C**H**$_{\mathbf{aromatic}}$CO), 4.10–3.84 (m, 10 H, 4 C**H**$_{\mathbf{2}}$O, CC**H**$_{\mathbf{2}}$C), 1.99–1.76 (m, 8 H, 4 C**H**$_{\mathbf{2}}$$CH_3$), 1.22–1.01 (m, 12 H, 4 C**H**$_{\mathbf{3}}$), 0.28 (s, 9 H, 3 C**H**$_{\mathbf{3}}$Si); ^{13}C NMR (100 MHz, $CDCl_3$): δ (ppm) = 154.29, 153.71, 153.62, 153.45, 143.70, 143.67, 141.38, 131.73, 130.82, 128.52, 128.37, 128.32, 128.24, 123.50, 120.23, 117.55, 117.35, 117.24, 101.31, 100.28, 95.97, 91.67, 91.56, 90.21, 89.98, 86.59, 77.33, 71.27, 71.20, 71.12, 36.73, 22.88, 22.84, 22.81, 10.74, 10.70, 10.66, 0.11;HRMS (FAB) of $C_{36}H_{38}O_4$[M+H$^+$] calc. 794.379, found 794.3793; IR (ATR) ν = 2961.4, 2874.2, 2148.8, 1596.3, 1494.2, 1465.8, 1418.8, 1385.3, 1273.8, 1247.9, 1206.9, 1061.5, 1042.8, 1017.0, 984.4, 938.0, 921.9, 883.0, 856.4, 839.3, 820.6, 753.3, 689.9, 645.3, 621.3, 588.4, 551.4, 524.4, 491.4, 423.5 cm^{-1}.

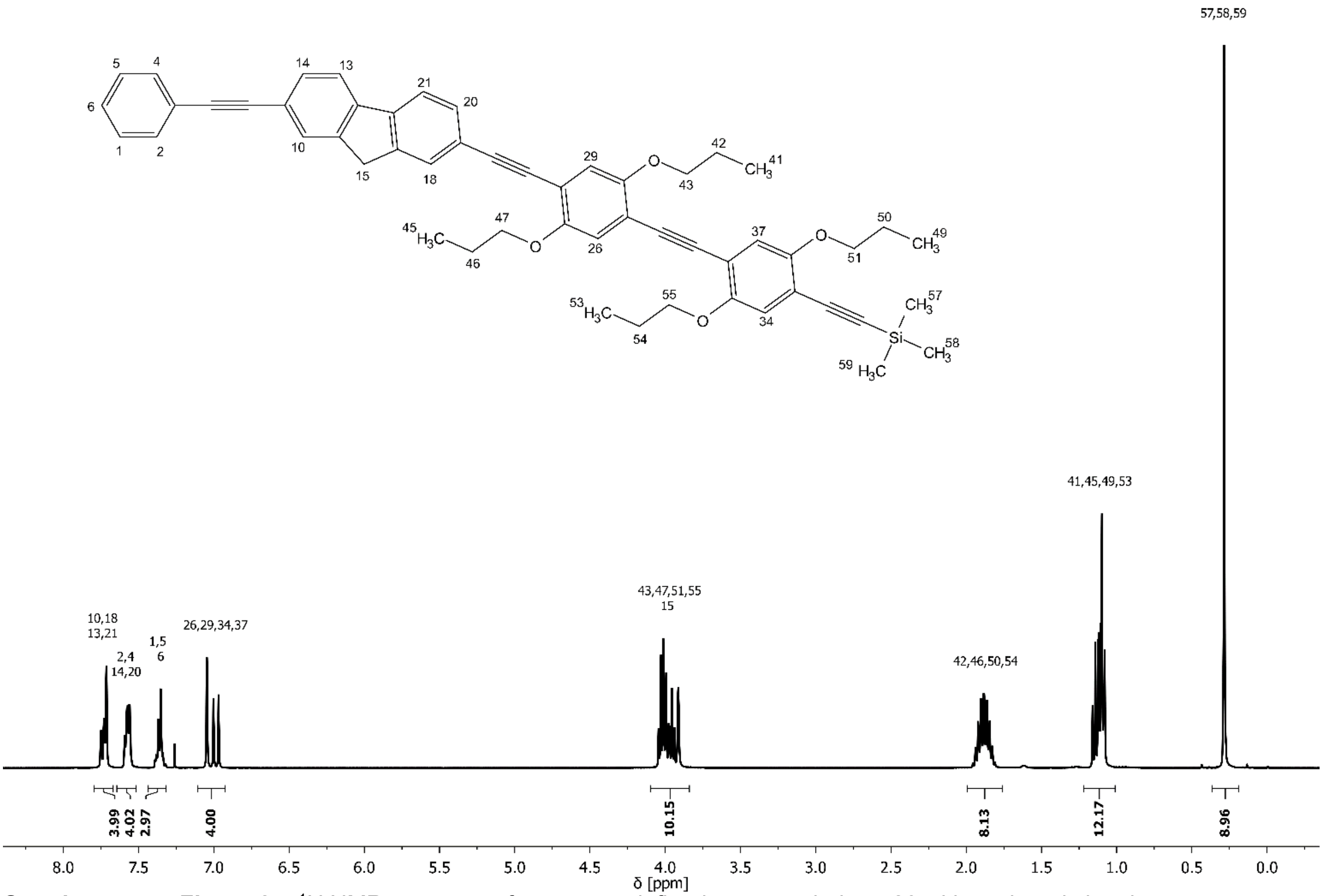

Supplementary Figure 84: ^{1}H NMR spectrum of sequence-defined, protected trimer **23** with assigned signals.

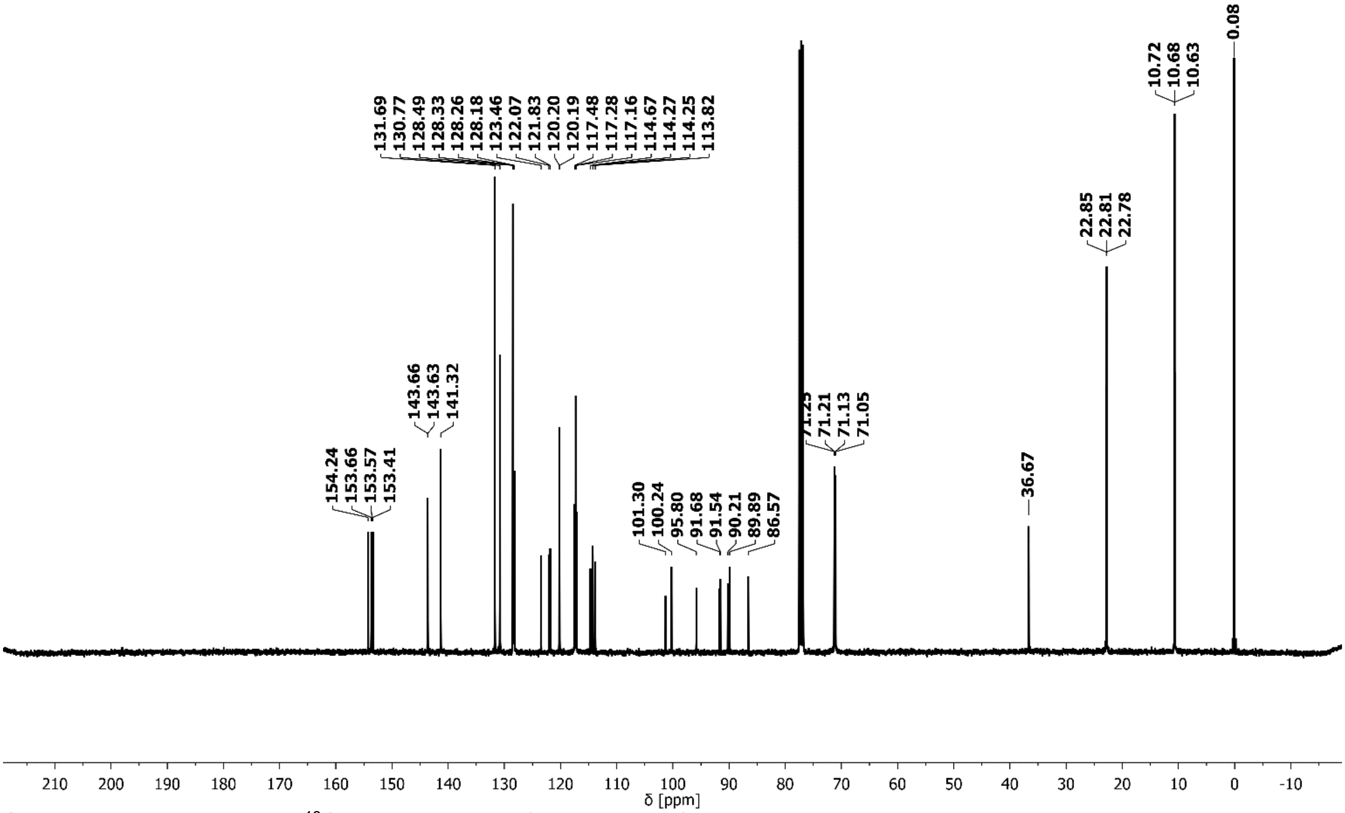

Supplementary Figure 85: ^{13}C NMR spectrum of sequence-defined, protected trimer **23**.

Synthesis of trimethyl((4-((7-((4-(phenylethynyl)-2,5-dipropoxyphenyl)ethynyl)-*9H*-fluoren-2-yl)ethynyl)-2,5-dipropoxyphenyl)ethynyl)silane **24**

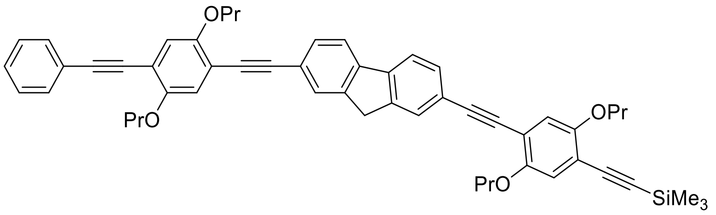

Compound **31** (324 mg, 0.640 mmol, 1.00 eq.), 5 mol% bis(triphenylphosphine)palladium(II)dichloride (22.5 mg, 32.1 µmol), 5 mol% copper(I)iodide (6.1 mg, 32.1 µmol) and 1,4-bis(propyloxy)-2-iodo-5-trimethylsilylacetylenebenzene **1** (799 mg, 1.92 mmol, 3.00 eq.) were placed into a Schlenk flask and degassed. Under continuous argon flow, 50 mL dry THF and 0.88 mL dry triethylamine (650 mg. 6.40 mmol, 10.0 eq.) were added and the mixture was stirred for 72 h at 45 °C. The reaction mixture was taken up in dichloromethane and washed with saturated NH_4Cl solution. The aqueous phase was extracted three times with dichloromethane. The combined organic layers were dried over Na_2SO_4, filtered and concentrated under reduced pressure. The residue was purified by silica column chromatography twice (cyclohexane / dichloromethane 2:1 → 3:2 and cyclohexane / ethyl acetate 20:1) to yield the product as yellow solid (319 mg, 63%); TLC (cyclohexane / dichloromethane 2:1) R_f = 0.20; ^{1}H NMR (400 MHz, $CDCl_3$): δ (ppm) = 7.82–7.67 (m, 4 H, 2 C**H**$_{\mathbf{aromatic}}$CHC-C≡C, 2 C**H**$_{\mathbf{aromatic}}$C-C≡C), 7.63–7.48 (m, 4 H, 2 C**H**$_{\mathbf{aromatic}}$CH, 2 C**H**$_{\mathbf{aromatic}}$ benzene end unit), 7.42–7.29 (m, 3 H, 3 C**H**$_{\mathbf{aromatic}}$ benzene end unit), 7.12–6.89 (m, 4 H, 4 C**H**$_{\mathbf{aromatic}}$CO), 4.12 – 3.86 (m, 10 H; 4 C**H$_2$**O, CC**H$_2$**C), 2.02 – 1.76 (m, 8 H, C**H$_2$**CH_3), 1.22 – 0.99 (m, 12 H, 4 C**H$_3$**), 0.28 (s, 9 H, 3 C**H$_3$**Si); ^{13}C NMR (100 MHz, $CDCl_3$): δ (ppm) = 154.34, 153.79, 153.75, 153.59, 143.68, 141.41, 141.38, 131.71, 130.82, 128.48, 128.23, 123.62, 120.22, 117.42, 117.15, 117.13, 117.10, 114.52, 114.25, 114.11, 113.82, 101.29, 100.26, 95.77, 95.74, 95.01, 86.57, 86.47, 86.10, 71.27, 71.22, 71.17, 36.74. 27.05, 22.89, 10.75, 10.74, 10.68, 0.11; HRMS (FAB) of $C_{36}H_{38}O_4$[M+H$^+$] calc. 794.3791, found 794.3792; IR (ATR) v = 2964.9, 2875.0, 2151.8, 1595.6, 1501.6, 1471.0, 1409.5, 1387.7, 1273.0, 1247.9, 1215.7, 1046.3, 1026.2, 977.2, 937.0, 920.8, 855.5, 841.5, 812.6, 754.8, 745.0, 683.8, 626.9, 580.6, 552.1, 526.4, 458.6, 417.0 cm^{-1}.

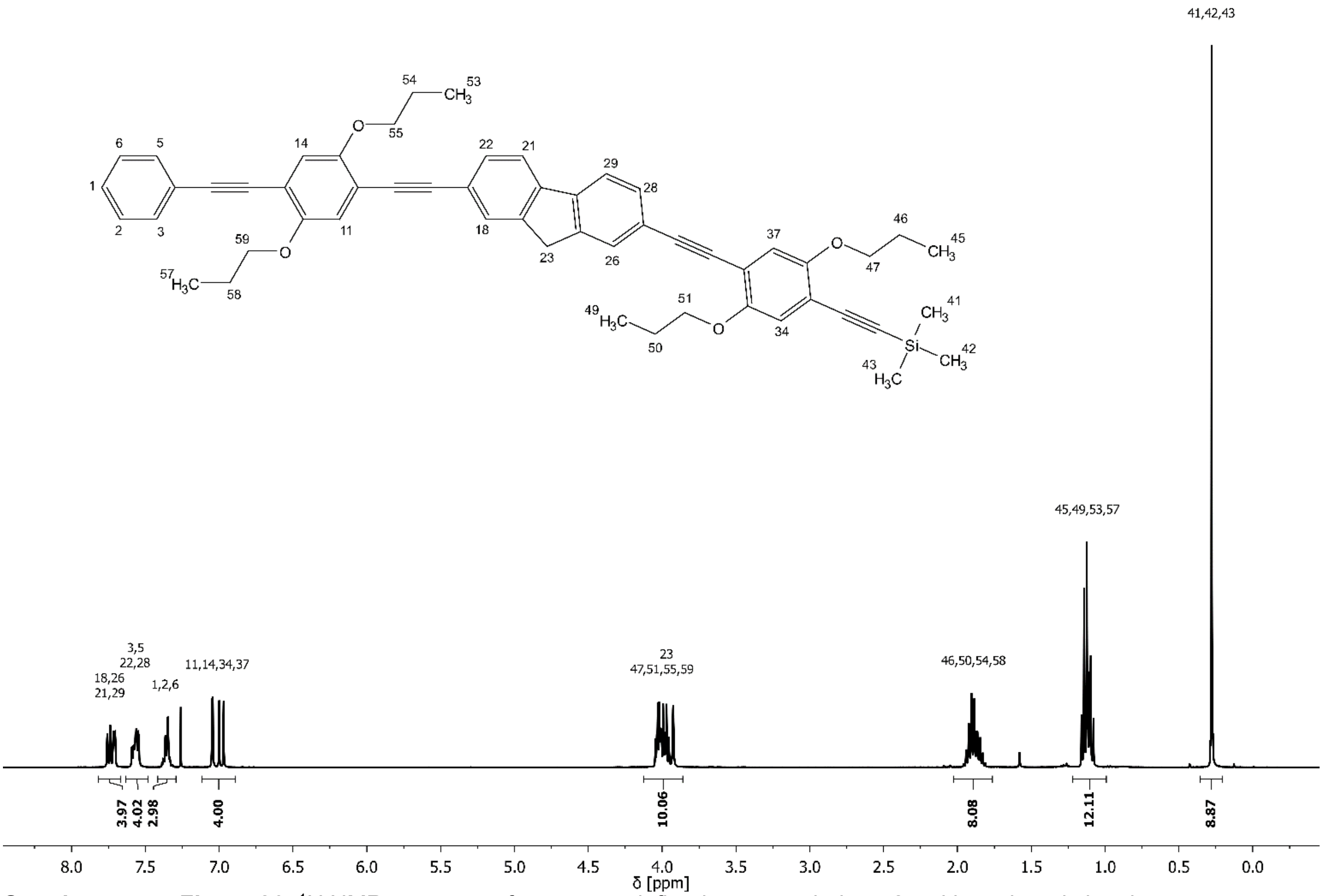

Supplementary Figure 86: ^{1}H NMR spectrum of sequence-defined, protected trimer **24** with assigned signals.

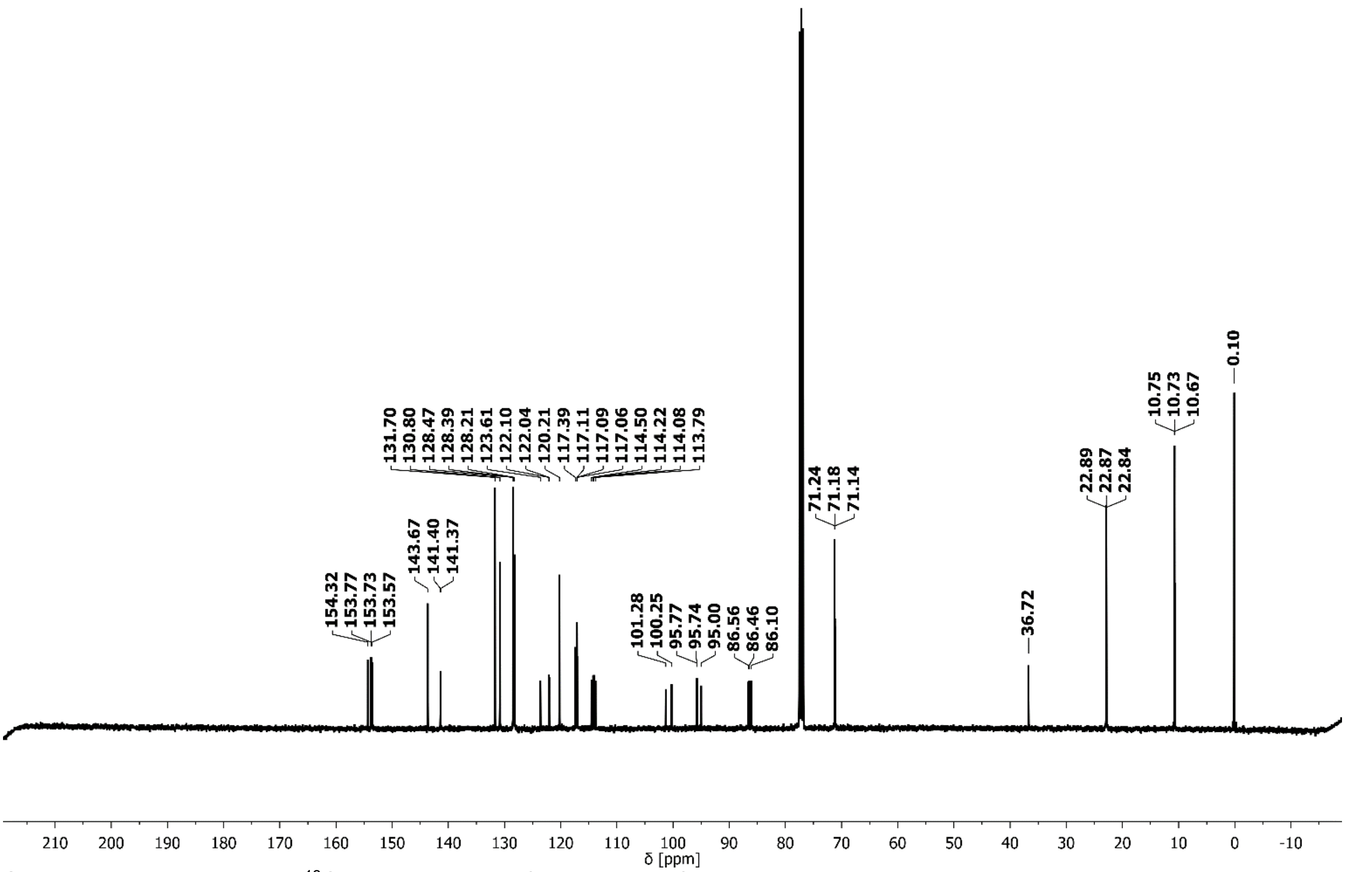

Supplementary Figure 87: ^{13}C NMR spectrum of sequence-defined, protected trimer **24**.

Synthesis of trimethyl((7-((4-((4-(phenylethynyl)-2,5-dipropoxyphenyl)ethynyl)-2,5-dipropoxyphenyl) ethynyl)-*9H*-fluoren-2-yl)ethynyl)silane **25**

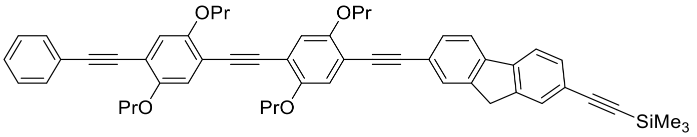

Compound **5** (450 mg, 0.842 mmol, 1.00 eq.), 5 mol% bis(triphenylphosphine)palladium(II)dichloride (29.5 mg, 42.0 µmol), 5 mol% copper(I)iodide (8.0 mg, 42.0 µmol) and 9-Iodo-2-trimethylsilylacetylene-9*H*-fluorene **22** (981 mg, 2.52 mmol, 3.00 eq.) were placed into a Schlenk flask and degassed. Under continuous argon flow, 50 mL dry THF and 1.17 mL dry triethylamine (852 mg. 8.42 mmol, 10.0 eq.) were added and the mixture was stirred for 72 h at 45 °C. The reaction mixture was taken up in dichloromethane and washed with saturated NH_4Cl solution. The aqueous phase was extracted three times with dichloromethane. The combined organic layers were dried over Na_2SO_4, filtered and concentrated under reduced pressure. The residue was purified by silica column chromatography twice (cyclohexane / dichloromethane 2:1 → 3:2 and cyclohexane / ethyl acetate 20:1) to yield the product as yellow solid (426 mg, 64%). TLC (cyclohexane/dichloromethane 2:1) R_f = 0.20; ^{1}H NMR (400 MHz, $CDCl_3$): δ (ppm) = 7.76–7.64 (m, 4 H, 2 C**H**$_{\textbf{aromatic}}$CHC-C≡C, 2 C**H**$_{\textbf{aromatic}}$C-C≡C), 7.63–7.55 (m, 3 H, 1 C**H**$_{\textbf{aromatic}}$CH, 2 C**H**$_{\textbf{aromatic}}$ benzene end unit), 7.52 (d, *J* = 7.9 Hz, 1 H, 1 C**H**$_{\textbf{aromatic}}$C-C≡C-Si), 7.42–7.30 (m, 3 H, 3 C**H**$_{\textbf{aromatic}}$ benzene end unit), 7.10–7.03 (m, 4 H, 4 C**H**$_{\textbf{aromatic}}$CO), 4.12–3.93 (m, 8 H; 4 C**H$_2$**O), 3.88 (s, 2 H, CC**H$_2$**C), 1.92 (p, *J* = 7.1 Hz, 8 H, C**H$_2$**CH_3), 1.24 – 1.01 (m, 12 H, 4 C**H$_3$**), 0.31 (s, 9 H, 3 C**H$_3$**Si); ^{13}C NMR (100 MHz, $CDCl_3$): δ (ppm) = 153.74, 153.62, 143.70, 143.51, 141.55, 141.31, 131.71, 130.80, 128.70, 128.47, 128.22, 120.27, 120.07, 117.38, 117.35, 117.29, 114.43, 114.40, 114.25, 114.18, 105.86, 95.76, 95.02, 94.60, 91.66, 91.64, 86.60, 86.12, 71.32, 71.21, 36.66, 31.73, 22.88, 22.87, 22.83, 22.80, 10.72, 0.17; HRMS (FAB) of $C_{36}H_{38}O_4$[M+H$^+$] calc 794.3791, found 794.3794; IR (ATR) ν = 2960.1, 2872.6, 2141.9, 1594.9, 1500.2, 1464.7, 1407.9, 1388.8, 1276.7, 1250.3, 1218.7, 1043.5, 1013.8, 974.1, 929.0, 854.7, 821.1, 753.8, 688.1, 665.6, 619.2, 555.9, 528.5, 426.6 cm^{-1}.

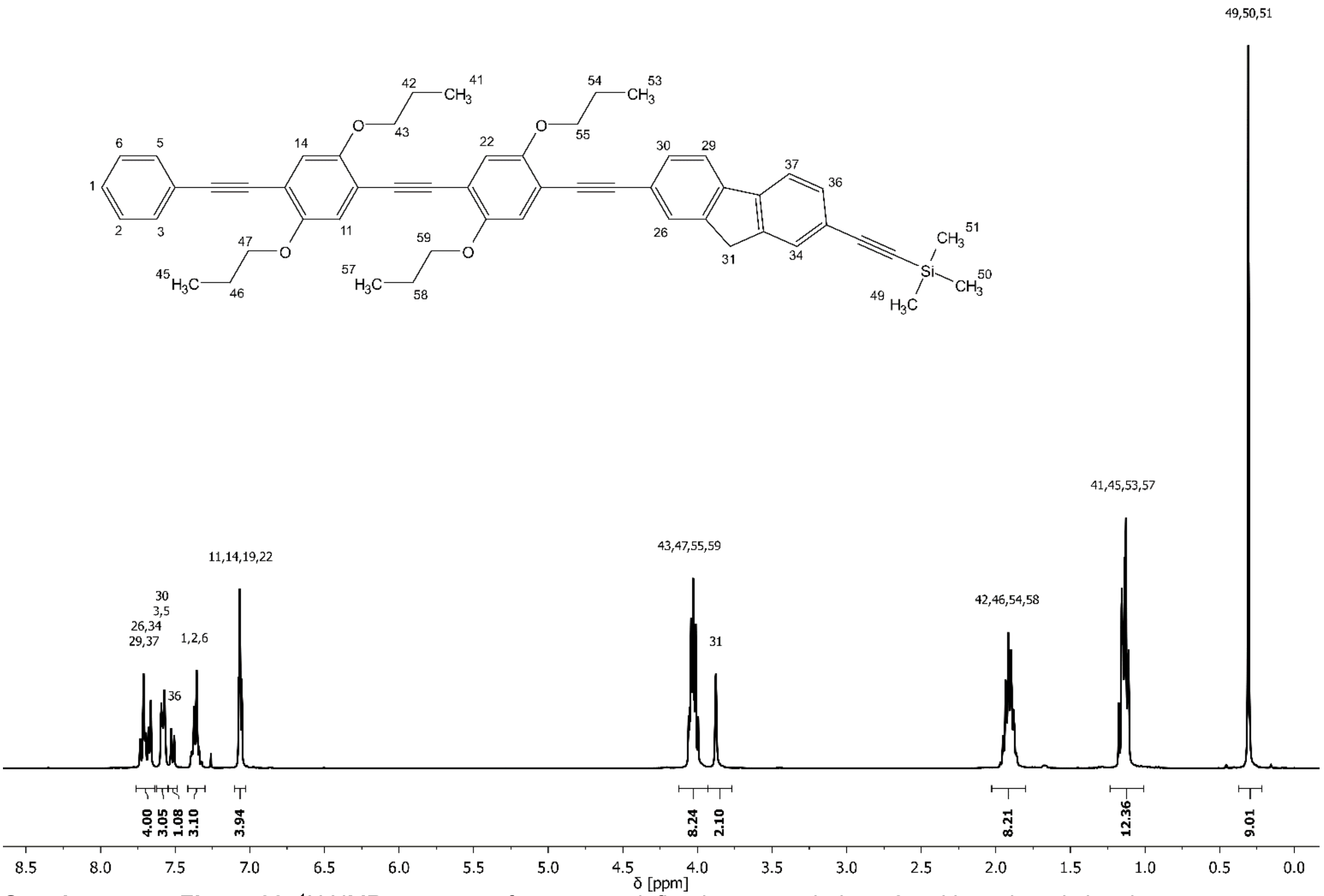

Supplementary Figure 88: ^{1}H NMR spectrum of sequence-defined, protected trimer **25** with assigned signals.

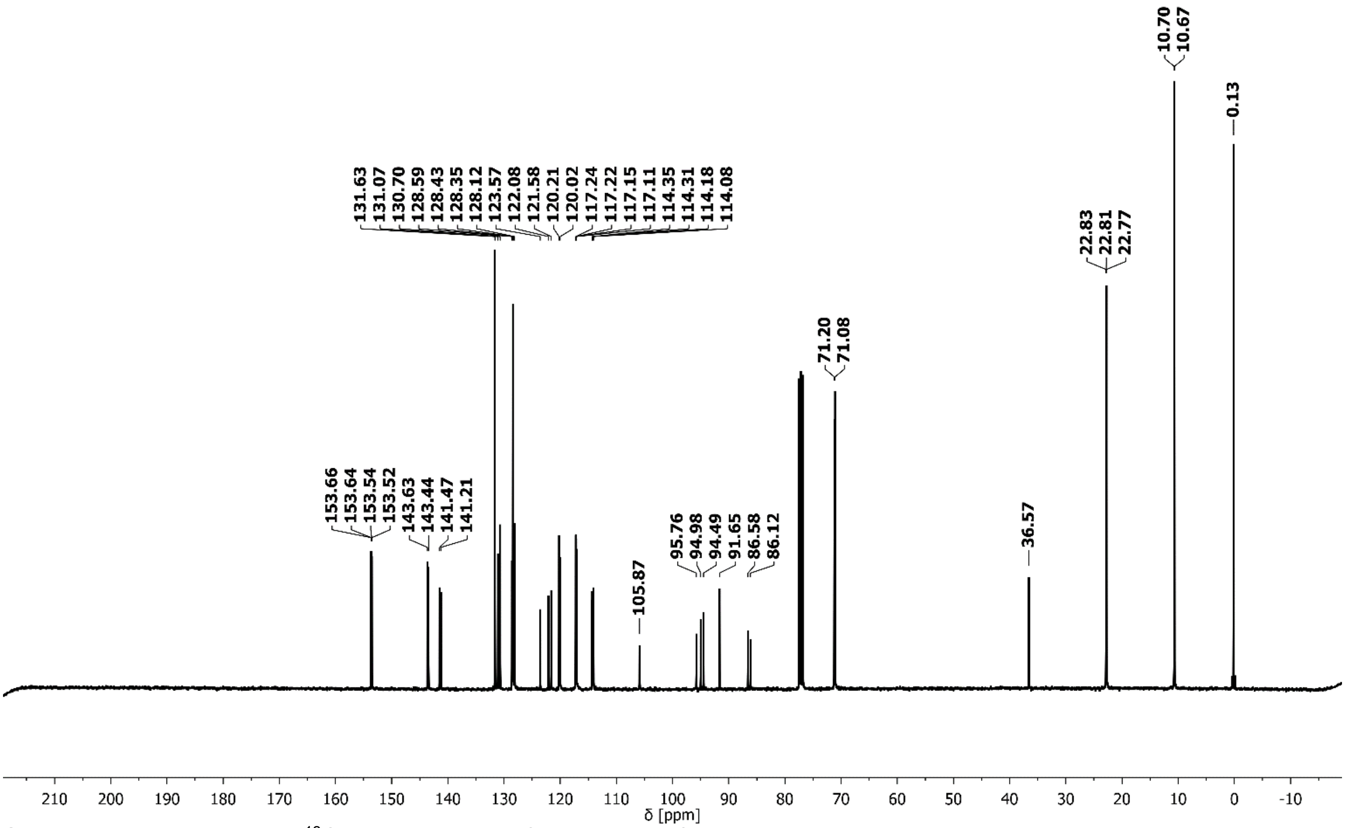

Supplementary Figure 89: ^{13}C NMR spectrum of sequence-defined, protected trimer **25**.

2. Overview of overall yields

Supplementary Table 1. Overall yields in comparison.

Reaction step	Monodisperse pentamer	Sequence-defined pentamer	SPOS	Sequence-defined fluorene position 1	Sequence-defined fluorene position 2	Sequence-defined fluorene position 3
1st	99% (**2**)	99% (**2**)		92% (**26**)	99% (**2**)	99% (**2**)
2nd	97% (**3**)	97% (**3**)	60% (**12**)	98% (**27**)	97% (**3**)	97% (**3**)
3rd	84% (**4**)	64% (**14**)		60% (**28**)	64% (**30**)	84% (**4**)
4th	100% (**5**)	98% (**15**)		95% (**29**)	93% (**31**)	100% (**5**)
5th	68% (**6**)	54% (**16**)		41% (**23**)	63% (**24**)	64% (**25**)
6th	98% (**7**)	98% (**17**)				
7th	65% (**8**)	37% (**18**)				
8th	99% (**9**)	85% (**19**)				
9th	53% (**10**)	33% (**20**)				
10th	98% (**11**)	97% (**21**)				
**Overall yield**	**18% for 5mer**	**3.2% for 5mer**	**60% for 1mer**	**21% for 3mer**	**36% for 3mer**	**52% for 3mer**

3. SEC Traces

3.1 Monodisperse Oligomers

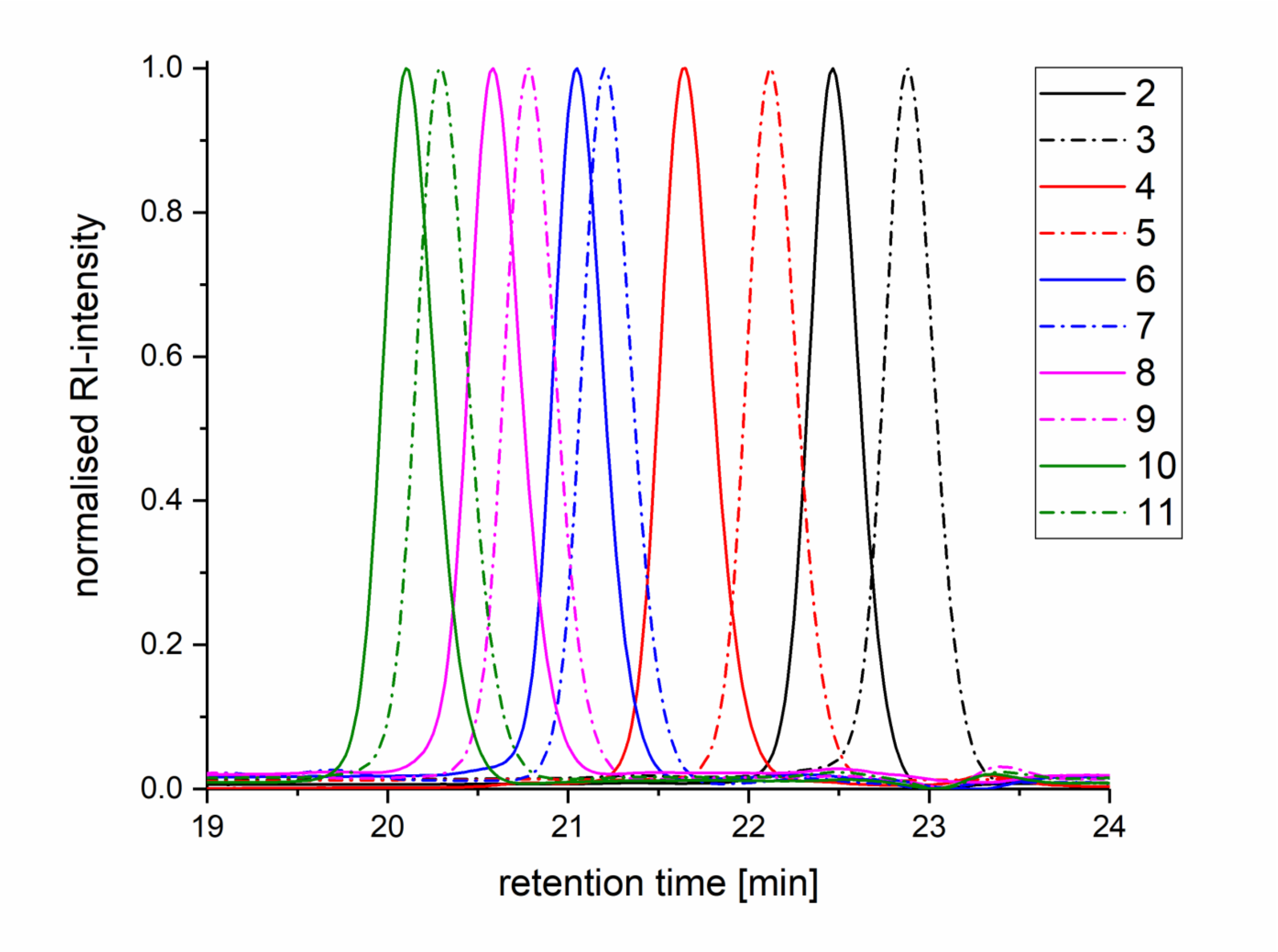

Supplementary Figure 90: SEC traces of the monodisperse oligomers **2-11**.

3.2 Sequence-Defined Trimers with one Fluorene Unit

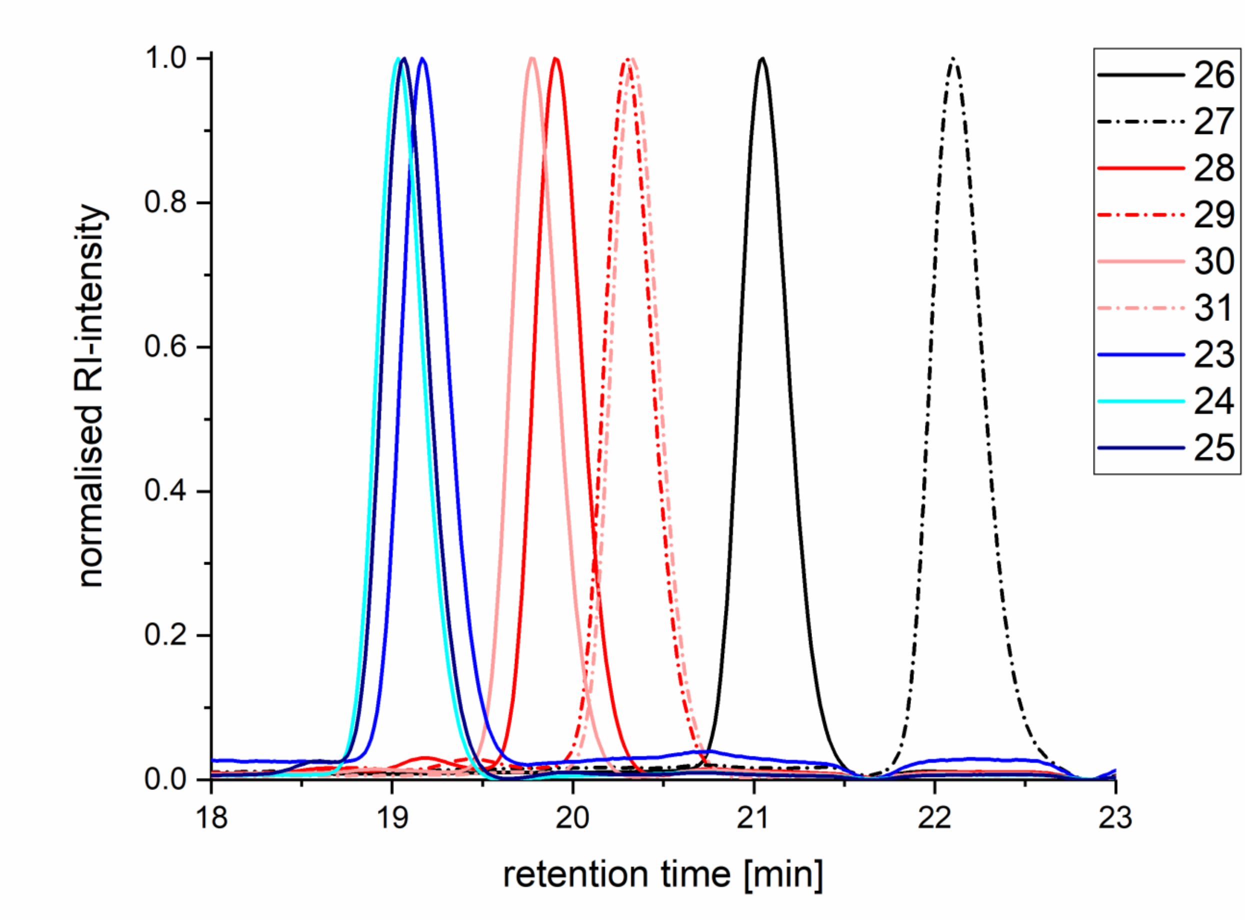

Supplementary Figure 91: SEC traces of the sequence-defined oligomers with fluorene units.

4. Optical properties

4.1 Sequence-Defined Oligomers

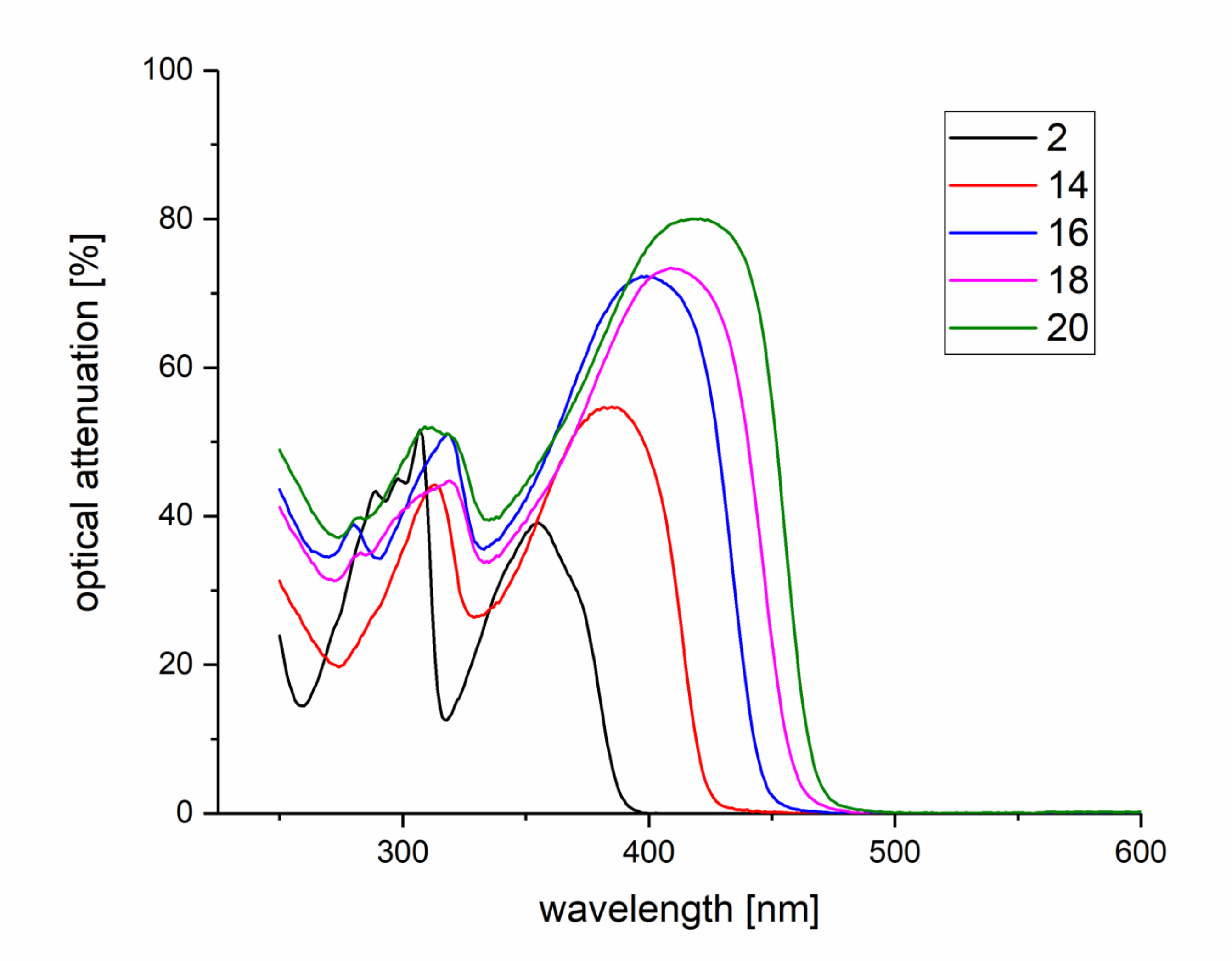

Supplementary Figure 92: Absorption spectra of the respective sequence-defined oligomers **2**-**20**.

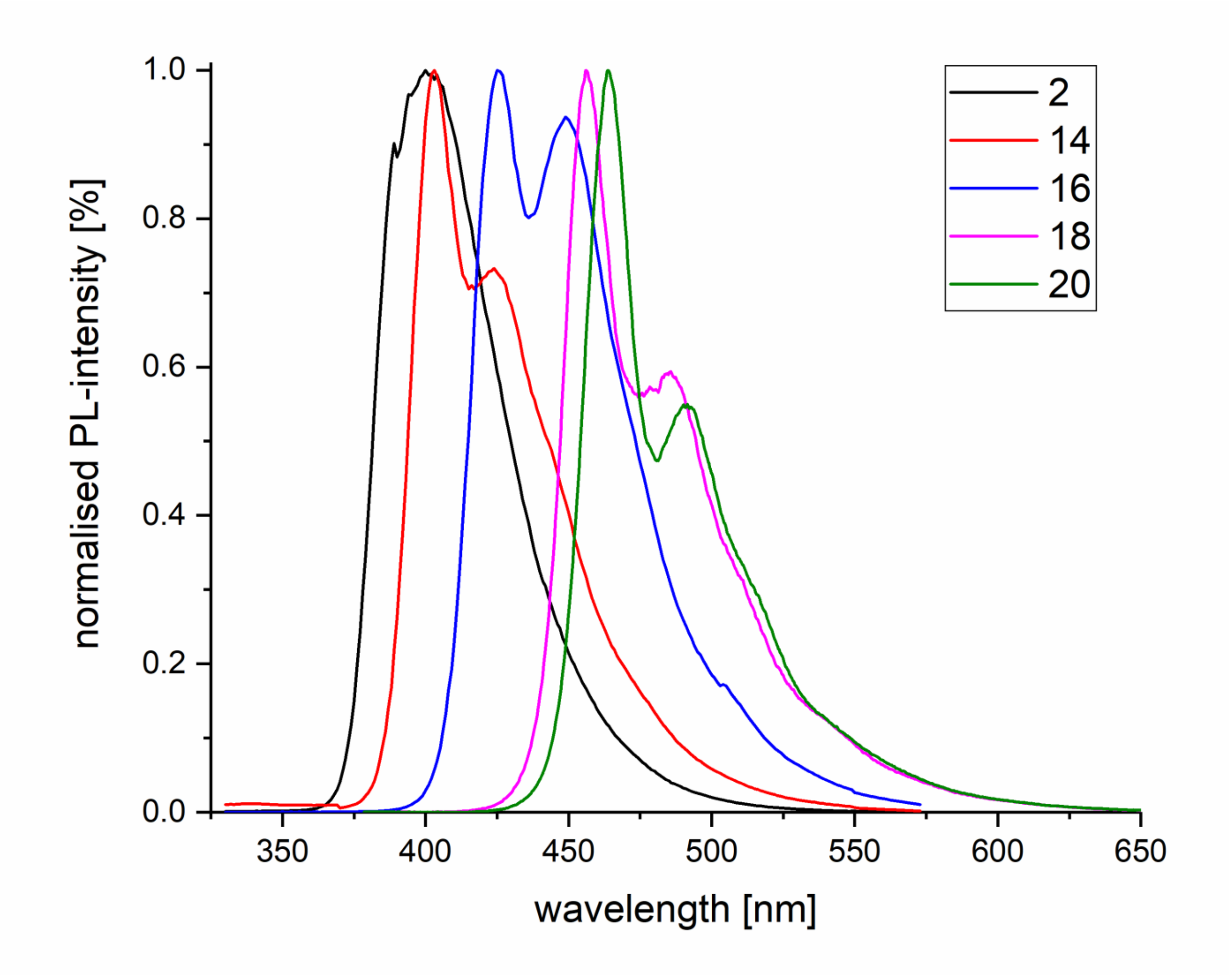

Supplementary Figure 93: Photoluminescence spectra of the respective sequence-defined oligomers **2**-**20**.

4.2 Sequence-Defined Trimers with one Fluorene Unit

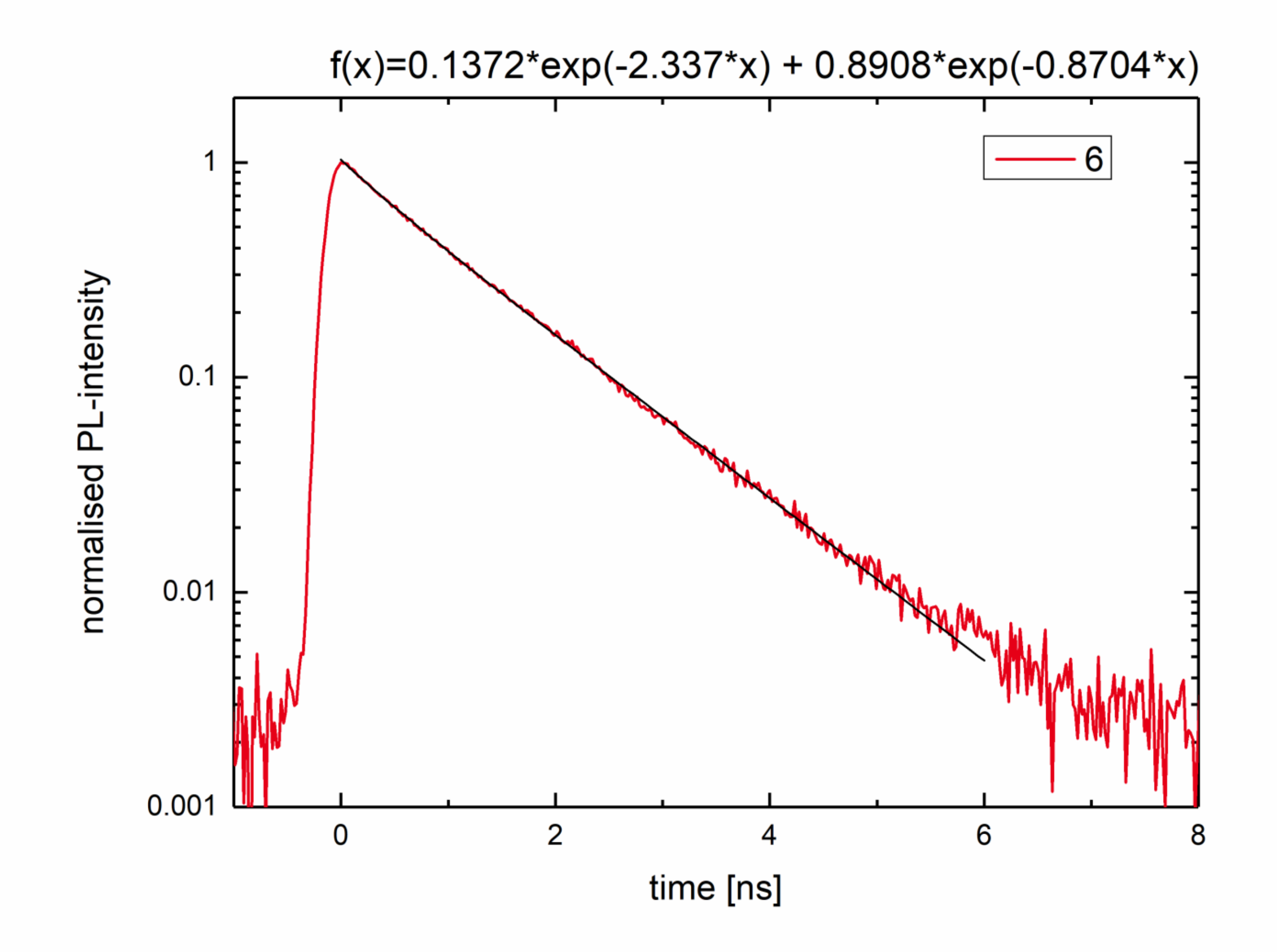

Supplementary Figure 94: Time-resolved photoluminescence spectra of the monodisperse trimer **6**.

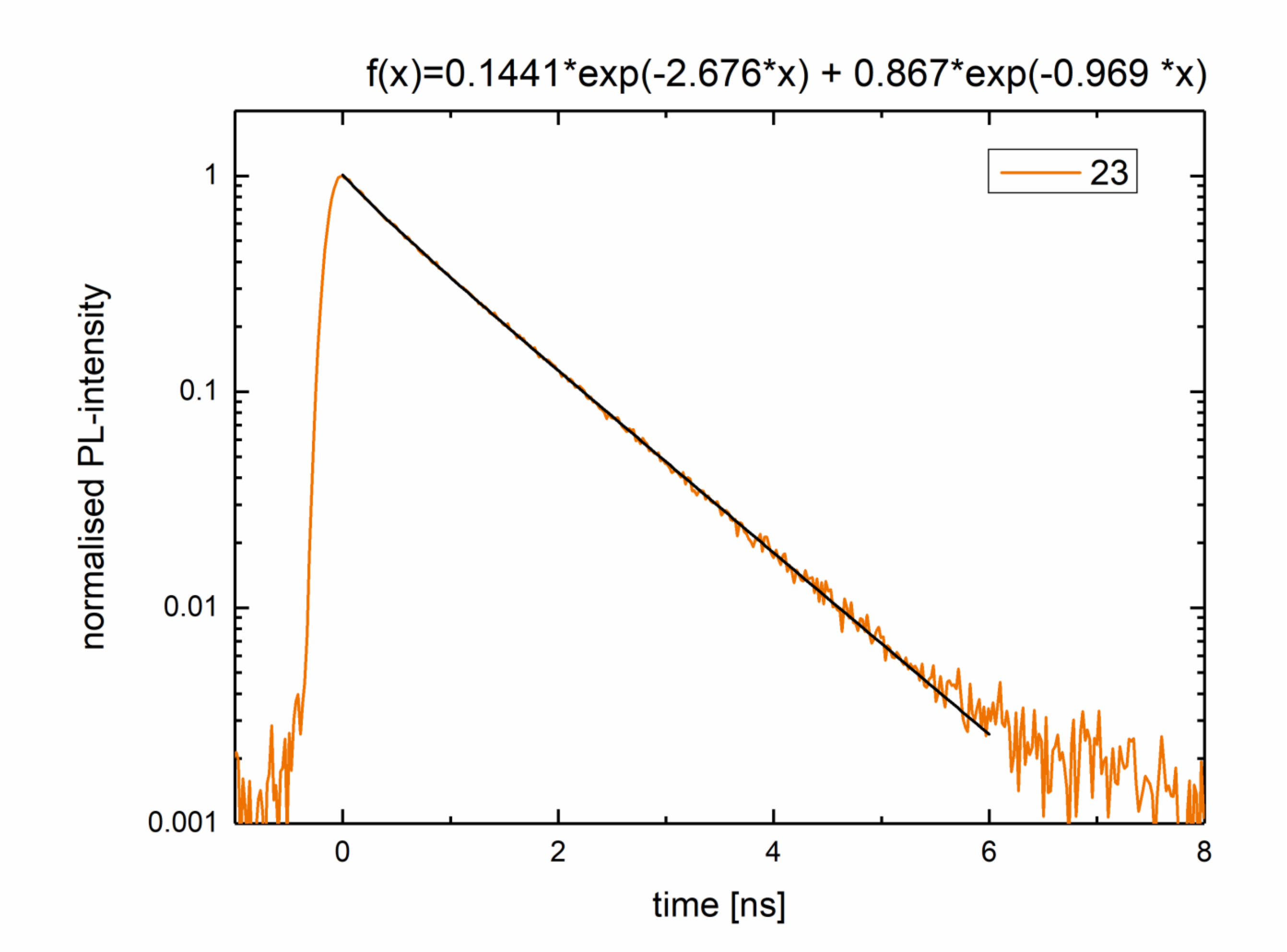

Supplementary Figure 95: Time-resolved photoluminescence spectra of the sequence-defined trimer **23**.

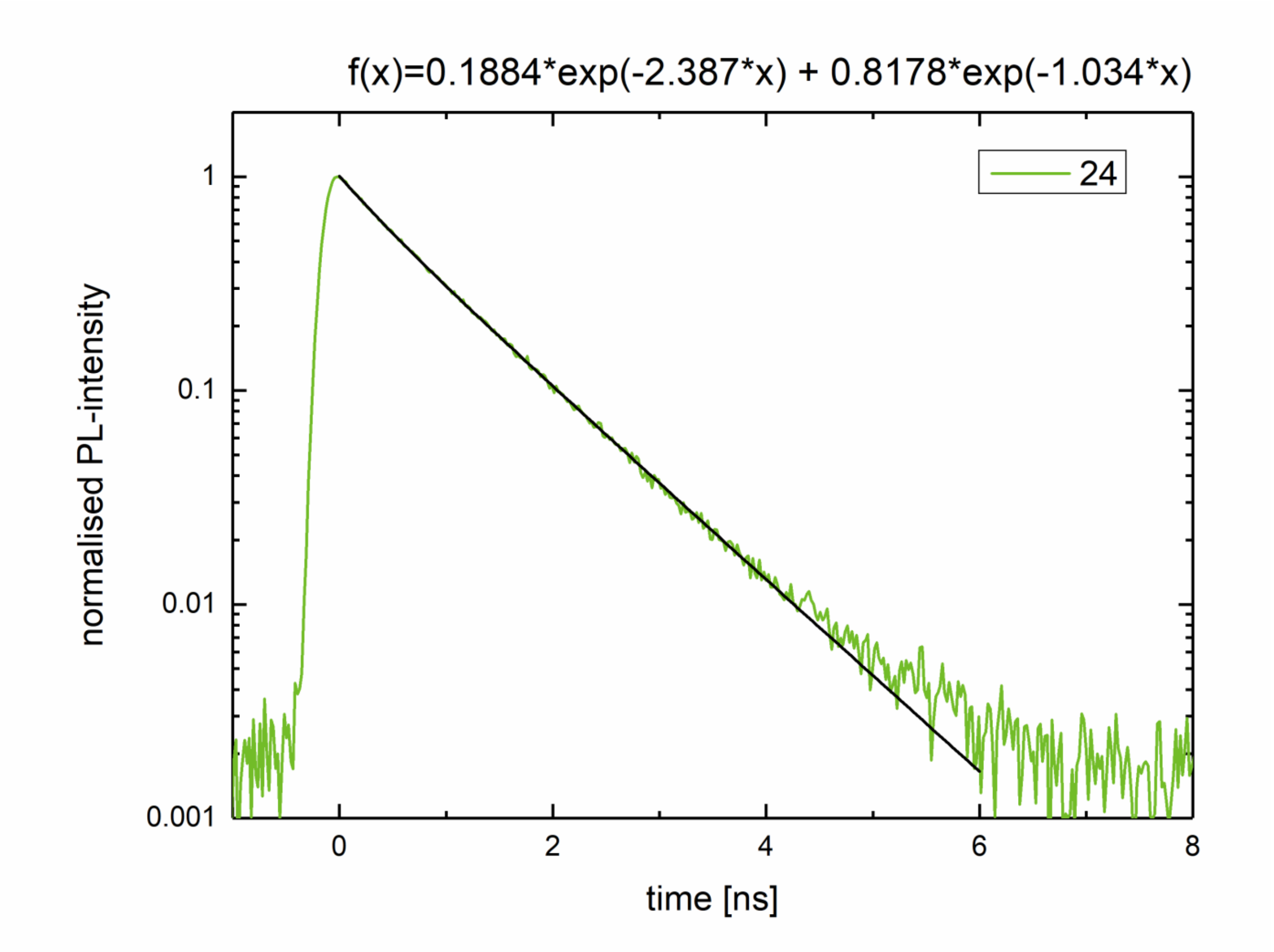

Supplementary Figure 96: Time-resolved photoluminescence spectra of the sequence-defined trimer **24**.

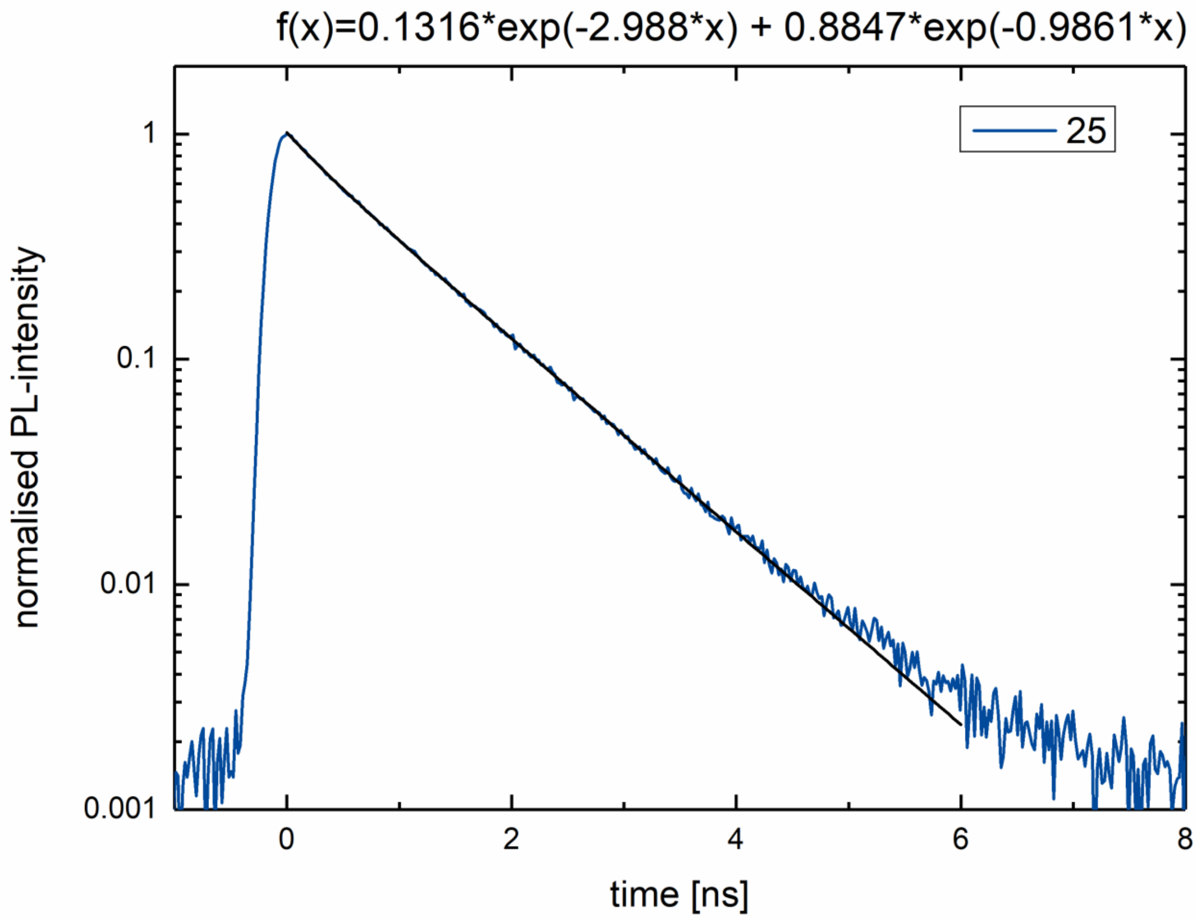

Supplementary Figure 97: Time-resolved photoluminescence spectra of the sequence-defined trimer **25**.

Supplementary Table 2: Photoluminescence Quantum Yield (PLQY), excitation wavelength of405 nm.

6	23	24	25
88.5%	88.7%	89.3%	83.3%

References

[1] J. C. de Mello, H. F. Wittmann, R. H. Friend, *Adv. Mater.* **1997**, *9*, 230–232.
[2] D. O. Faulkner, J. J. McDowell, A. J. Price, D. D. Perovic, N. P. Kherani, G. A. Ozin, *Laser & Photon. Rev.* **2012**, *6*, 802-806.
[3] H. Meier, D. Ickenroth, U. Stalmach, K. Koynov, A. Bahtiar, C. Bubeck, *Eur. J. Org. Chem.* **2001**, *2001*, 4431-4443.
[4] S.-B. Ko, A.-N. Cho, M.-J. Kim, C.-R. Lee, N.-G. Park, *Dyes. Pigm.* **2012**, *94*, 88-98.
[5] J.-F. Morin, T. Sasaki, Y. Shirai, J. M. Guerrero, J. M. Tour, *J. Org. Chem.* **2007**, *72*, 9481-9494.
